# Community Structure and Toxicity Potential of Cyanobacteria during Summer and Winter in a Temperate-Zone Lake Susceptible to Phytoplankton Blooms

**DOI:** 10.3390/toxins16080357

**Published:** 2024-08-14

**Authors:** Łukasz Wejnerowski, Tamara Dulić, Sultana Akter, Arnoldo Font-Nájera, Michał Rybak, Oskar Kamiński, Anna Czerepska, Marcin Krzysztof Dziuba, Tomasz Jurczak, Jussi Meriluoto, Joanna Mankiewicz-Boczek, Mikołaj Kokociński

**Affiliations:** 1 Department of Hydrobiology, Institute of Environmental Biology, Faculty of Biology, Adam Mickiewicz University, Uniwersytetu Poznańskiego 6, 61-614 Poznań, Poland; oskkam@st.amu.edu.pl (O.K.); anncze7@st.amu.edu.pl (A.C.); mikolaj.kokocinski@amu.edu.pl (M.K.); 2 Biochemistry and Cell Biology, Faculty of Science and Engineering, Åbo Akademi University, Tykistökatu 6A, 20520 Turku, Finland; tdulic@abo.fi; 3 Biotechnology, Department of Life Technologies, Faculty of Technology, University of Turku, 20520 Turku, Finland; suakte@utu.fi; 4 European Regional Centre for Ecohydrology of the Polish Academy of Sciences, Tylna 3, 90-364 Łódź, Poland; a.font-najera@erce.unesco.lodz.pl; 5 Department of Water Protection, Institute of Environmental Biology, Faculty of Biology, Adam Mickiewicz University, Uniwersytetu Poznańskiego 6, 61-614 Poznań, Poland; m.rybak@amu.edu.pl; 6 Department of Ecology and Evolutionary Biology, University of Michigan, Ann Arbor, MI 48109, USA; marcind@umich.edu; 7 UNESCO Chair on Ecohydrology and Applied Ecology, Faculty of Biology and Environmental Protection, University of Lodz, Banacha 12/16, 90-237 Lodz, Poland; tomasz.jurczak@biol.uni.lodz.pl (T.J.); joanna.mankiewicz@biol.uni.lodz.pl (J.M.-B.)

**Keywords:** cyanotoxins, cyanobacterial blooms, cyanotoxin immunoassays, high-performance liquid chromatography–mass spectrometry, toxigenicity

## Abstract

Cyanobacterial blooms are increasingly common during winters, especially when they are mild. The goal of this study was to determine the summer and winter phytoplankton community structure, cyanotoxin presence, and toxigenicity in a eutrophic lake susceptible to cyanobacterial blooms throughout the year, using classical microscopy, an analysis of toxic cyanometabolites, and an analysis of genes involved in biosynthesis of cyanotoxins. We also assessed whether cyanobacterial diversity in the studied lake has changed compared to what was reported in previous reports conducted several years ago. Moreover, the bloom-forming cyanobacterial strains were isolated from the lake and screened for cyanotoxin presence and toxigenicity. Cyanobacteria were the main component of the phytoplankton community in both sampling times, and, in particular, Oscillatoriales were predominant in both summer (*Planktothrix*/*Limnothrix*) and winter (*Limnothrix*) sampling. Compared to the winter community, the summer community was denser; richer in species; and contained alien and invasive Nostocales, including *Sphaerospermopsis aphanizomenoides*, *Raphidiopsis raciborskii*, and *Raphidiopsis mediterranea*. In both sampling times, the blooms contained toxigenic species with genetic determinants for the production of cylindrospermopsin and microcystins. Toxicological screening revealed the presence of microcystins in the lake in summer but no cyanotoxins in the winter period of sampling. However, several cyanobacterial strains isolated from the lake during winter and summer produced anabaenopeptins and microcystins. This study indicates that summer and winter blooms of cyanobacteria in the temperate zone can differ in biomass, structure, and toxicity, and that the toxic hazards associated with cyanobacterial blooms may potentially exist during winter.

## 1. Introduction

Excessive nutrient input into aquatic ecosystems, together with climate change, accelerates their eutrophication and makes them more susceptible to cyanobacterial blooms—it is a phenomenon that constitutes a global problem. A recent estimate [1] showed that eutrophication, just in lakes, will increase by 25–200% by the middle of the 21st century and double or quadruple by the end of the century as a result of the changing climate. The more eutrophic the lake ecosystems, the more incidences of bloom-forming cyanobacteria in lakes and, consequently, the greater the challenge from the aquatic environment-management perspective.

The blooming of cyanobacteria carries numerous negative consequences for the ecosystem, from light limitation [2] and alterations of food-web structure [3] to biodiversity loss [4]. Among the characteristics of cyanobacteria that determine their harmfulness (e.g., ability to form surface scums [5] and low nutritional value to consumers [6]), the ability of these prokaryotes to synthesize and secrete a variety of toxic metabolites [7,8] is probably the most important. According to the National Rivers Authority [9], 25%–75% of cyanobacterial blooms can be toxic, and they may cause the mortality of aquatic organisms and pose a threat to other users of water resources [10,11]. Moreover, toxic cyanobacterial blooms, as well as the release of toxic cyanometabolites in water, e.g., during bloom collapse, can impair the recreational and economic functions of water bodies and disqualify the lake/reservoir as a drinking water resource (e.g., see [12]). At the same time, preventing toxic cyanobacterial blooms and mitigating existing problems can come at a high cost [13,14].

Considering the response of bloom-forming cyanobacteria to climate change, one can conclude that aquatic ecosystems face a future with more toxic cyanobacterial biomass if projections regarding increases in temperature and concentrations of greenhouse gasses in the atmosphere are realized by the end of the 21st century [15]. For example, an increase in temperature, apart from intensifying the growth of cyanobacteria [16], was found to increase the production of toxic metabolites for some cyanobacteria (e.g., nodularin for *Nodularia*, [17], saxitoxin for *Aphanizomenon*, [18], or microcystins for *Planktothrix*, [19]). Other toxic cyanobacteria, e.g., *Microcystis aeruginosa*, can also exhibit similar responses when a temperature rise is accompanied by extra nutrient input [20]. Apart from increasing toxin production, temperature elevation can favor toxic genotypes and, consequently, promote their dominance in the cyanobacterial community [21].

Blooms also like it cold—there are increasing reports of cyanobacterial bloom-forming taxa and even active blooms in wintertime, even under the ice cover, and they are often formed by the same bloom-forming and potentially toxic species as in summertime [22,23,24,25,26,27,28]. The presence of toxic cyanometabolites in winter blooms (anatoxin-a [29] and microcystins [25,30,31]) shows that cyanobacterial blooms can be “cold and toxic”. Synthesis of cyanotoxins can also aid cyanobacteria in acclimation to a lower water temperature, as shown by Stark et al. [32] for microcystin-producing *Microcystis aeruginosa* and its non-toxigenic mutant strain. Moreover, toxic cyanometabolites, i.e., microcystins, were found even in cyanobacteria living in extremely cold glacial ecosystems [33,34]. In addition, winter populations of cyanobacteria can exert toxic effects on other species in the aquatic environment [31,35].

The main objective of the present study was to determine the community structure, toxicity, and toxigenicity of the summer and winter blooming of cyanobacteria and to compare whether cyanobacterial diversity in the studied lake has changed in relation to previous reports conducted several years ago. Therefore, we sampled a shallow, eutrophic lake, Lubosińskie Lake, in summer and winter, analyzed the structure of cyanobacterial communities, and studied the presence of cyanotoxins in the lake and in the isolated strains of cyanobacteria. The strain toxigenicity and real capability to produce toxigenic cyanometabolites were not previously investigated in cyanobacteria from this lake. Our study provides special insight into the toxicity of summer and winter cyanobacterial blooms and the potential threats associated with ongoing climate change.

## 2. Results

### 2.1. Physicochemical Parameters and Phytoplankton Structure in Lubosińskie Lake

The surface-water temperature of Lubosińskie Lake was 22.6 °C on 2 September 2019 and 4.2 °C on 11 February 2020, while water transparency was 0.2 m and 0.4 m, respectively. There was no ice cover on the lake during winter sampling. Values of the remaining measured parameters of surface water during summer and winter sampling, respectively, were as follows: 8.8 and 8.5 (pH), 616 and 711 µS/cm (conductivity), 2.23 and 1.6 mg N/L (total nitrogen), 0.126 and 0.435 mg/L (N-NO_3_), 0.004 and 0.012 mg/L (N-NO_2_), 2.1 and 1.15 mg/L (Kjeldahl nitrogen), 0.215 and 0.24 mg P/L (total phosphorus), and 0.081 and 0.137 mg P/L (total reactive phosphorus).

**Figure 1:**
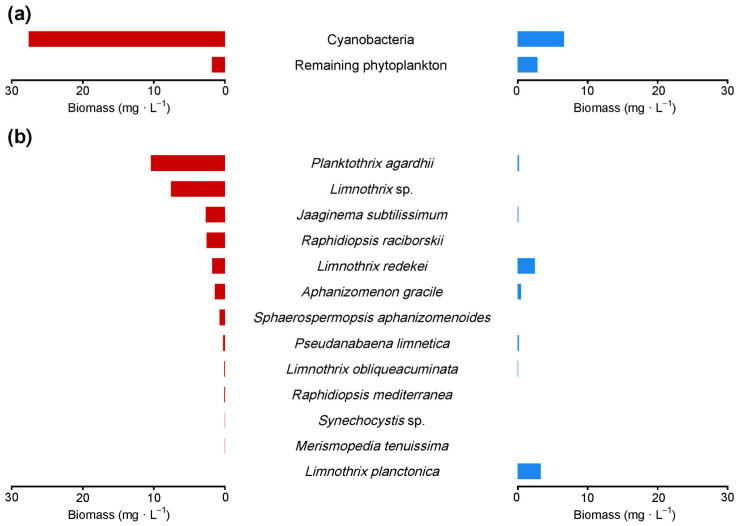
The proportion of cyanobacteria in the phytoplankton (**a**) and quantitative structure of cyanobacterial communities (**b**) of the Lubosińskie Lake during summer (left panel) and winter (right panel). No bar means that a given taxon was absent.

The phytoplankton biomass was 29.4 mg/L on 2 September 2019 and 9.4 mg/L on 11 February 2020. Cyanobacteria constituted ~94% of summer and ~70% of winter phytoplankton (Figure 1a). Apart from cyanobacteria, summer phytoplankton contained members of Bacillariophyceae (two taxa), Chlorophyta (five taxa), Cryptophyceae (four taxa), Dinophyceae (one taxon), and Haptophyceae (one taxon). In winter, Bacillariophyceae were represented by two taxa, Chlorophytes by one taxon, Cryptophyceae by three taxa, Dinophyceae by two taxa, and there were no representatives of Haptophyceae. Numerical values of phytoplankton taxa biomass and species or genus authority are outlined in Supplementary Materials S1.

**Figure 2:**
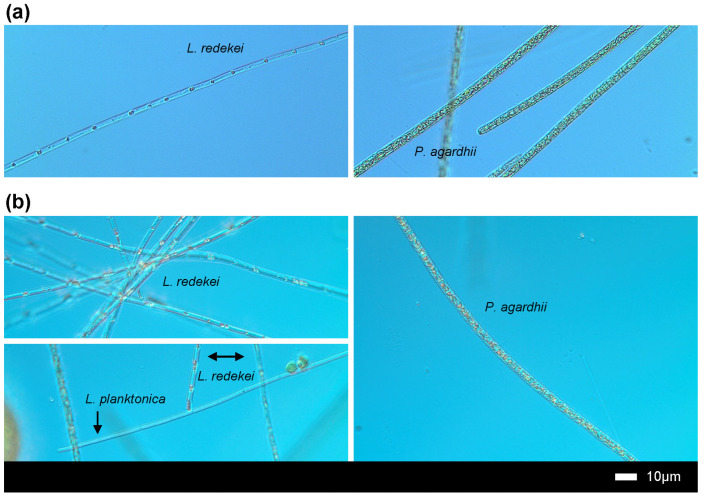
Micrographs of *Limnothrix* and *Planktothrix* specimens from summer (**a**) and winter (**b**) samples, conducted using light microscope with Nomarski interference contrast.

The summer cyanobacterial bloom was dominated (~61% of total biomass) by *Planktothrix agardhii* and *Limnothrix* sp. (10.39 and 7.58 mg/L, respectively); there were also four subdominants (*Jaaginema subtilissimum*, *Raphidiopsis raciborskii*, *Limnothrix redekei*, and *Aphanizomenon gracile*) of biomass ranging from 2.69 to 1.39 mg/L; and six taxa (*Sphaerospermopsis aphanizomenoides*, *Pseudanabaena limnetica*, *Limnothrix obliqueacuminata*, *Raphidiopsis mediterranea*, *Synechocystis* sp., and *Merismopedia tenuissima*) of biomass less than 0.8 mg/L (Figure 1b, left panel). Two *Limnothrix* species dominated the cyanobacterial biomass in winter (*L. planctonica*, 3.27 mg/L; and *L. redekei*, 2.41 mg/L) (Figure 1b, right panel) (Figure 2), while the biomass of other taxa (*A. gracile*, *P. limnetica*, *P. agardhii*, *J. subtilissimum*, and *L. obliqueacuminata*) was lower than 0.5 mg/L.

### 2.2. Overview of Cyanobacteria Strains Isolated from the Lake during Summer and Winter

**Figure 3:**
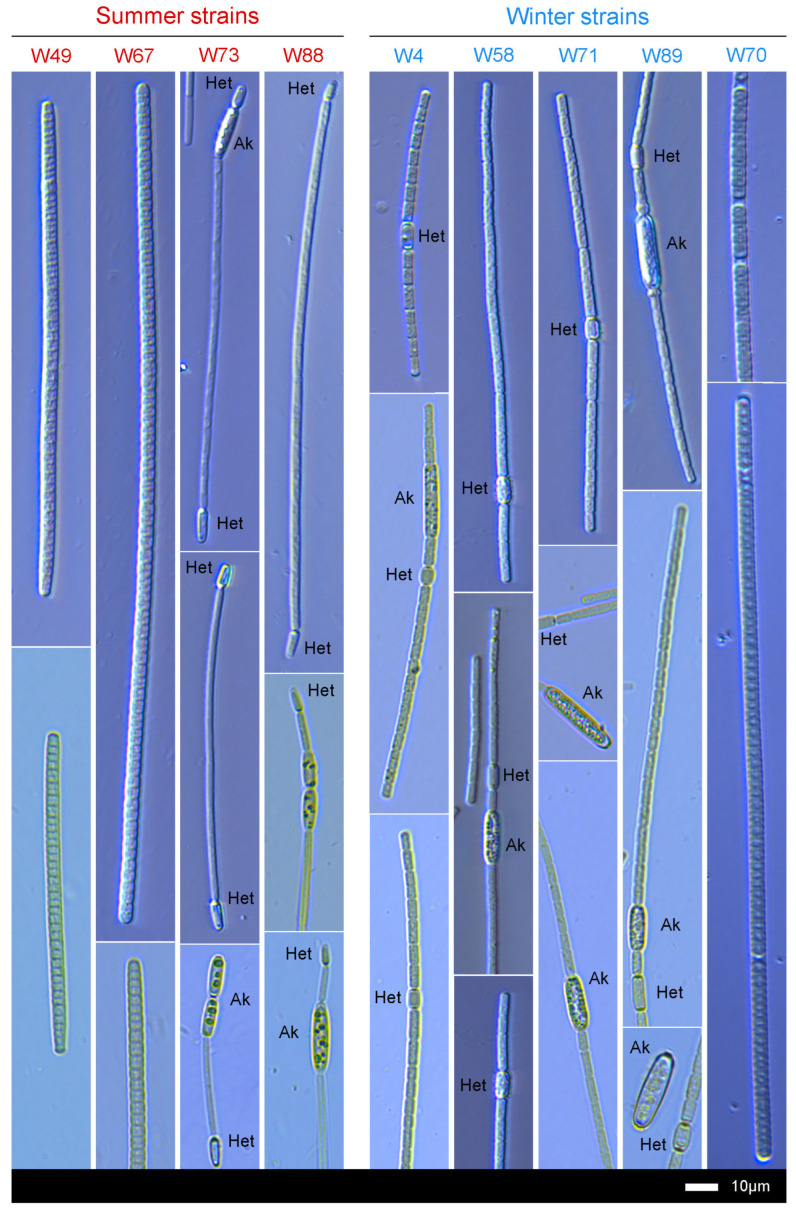
Micrographs of cyanobacterial strains isolated from summer and winter phytoplankton of Lubosińskie Lake, conducted using light microscope with integrated modulation contrast.

Microscopic inspection of successfully growing strains showed that the summer set of strains comprised *P. agardhii* (W49 and W67 strains) and *R. raciborskii* (W73 and W88 strains), whereas the winter set included four isolates of *A. gracile* (W4, W58, W71, and W89) and one *P. agardhii* (W70) (Figure 3). Specimens of each strain possessed key characteristic morphological features of the species to which they were classified: (1) *A. gracile*: trichomes solitary and free-floating, vegetative cells 2.4–3.4 µm wide and 3.5–7.4 µm long in the middle of trichomes, apical cells non-hyaline and capitate at the end, barrel-shaped and intercalary heterocytes (3.4–4.7 µm wide and 5.7–8.9 µm long), akinetes (3.6–7.2 µm wide and 16.9–26.1 µm long), cylindrical, possessing at poles often cup-shaped sheath formations opened towards the neighboring vegetative cells; (2) *P. agardhii*: trichomes mostly solitary and free-floating sometimes with fine sheaths, vegetative cells 3.7–5.2 µm wide and 1.6–3.8 µm long, apical cells bluntly conical or sometimes capitate; (3) *R. raciborskii*: free-floating trichomes, vegetative cells 1.9–3.4 µm wide and 7.1–15.9 µm long, heterocytes 2.5–3.8 µm wide and 5.7–11.4 µm long, drop-like, terminal and present at one or two ends of a trichome, akinetes of 3.5–5.7 µm width and 11.8–24.1 µm length, cylindrical-oval and adjacent to the heterocytes or slightly distant from these cells.

**Figure 4:**
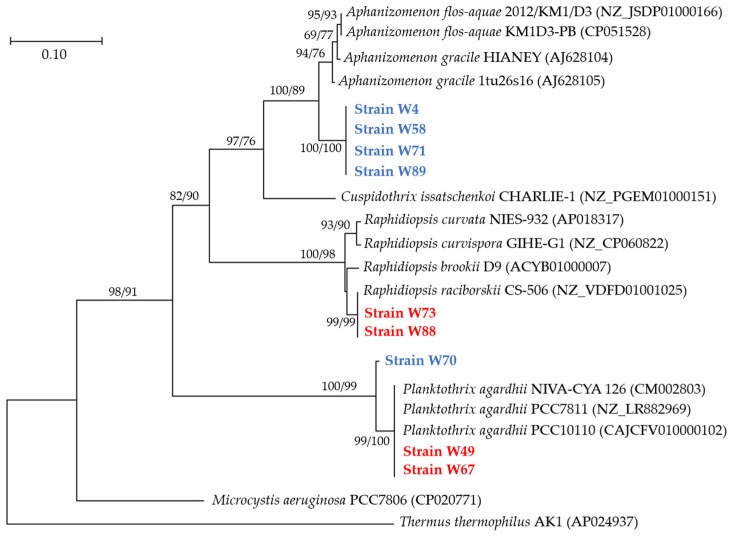
Phylogenetic reconstruction based on the *rpo*B gene for the isolated cyanobacterial strains from the Lubosińskie Lake. The tree was constructed using the NJ topology and the numbers associated with the nodes represent the bootstrap support values of NJ and ML analyses, respectively. Only bootstrap supports ≥ 50% were reported. The bar above the tree represents nucleotide substitutions per position. Accession numbers were given for *rpo*B sequences obtained from GenBank. The sequences of *Microcystis aeruginosa* PCC7806 and *Thermus thermophilus* AK1 were used as outgroups to cluster the representative strains in the phylum Cyanobacteria. Red and blue colors indicate summer and winter strains, respectively.

Phylogenetic analyses based on *rpo*B gene sequencing showed that sequences of W4, W58, W71, and W89 strains clustered together and exhibited a short phylogenetic distance to reference sequences of *A. gracile* and *A. flos-aquae* from GenBank (Figure 4). Considering that specimens in each of these four strains had neither hyaline terminal/subterminal cells nor the tendency to aggregate into fascicles, these strains were finally determined to be *A. gracile*. Sequences of W49 and W67 strains from summer phytoplankton and the sequence of W70 strain from winter clustered together with the reference sequences of *P. agardhii*. The remaining two sequences (W73 and W88 strains) clustered together with sequences of *Raphidiopsis*, and they had slightly shorter phylogenetic distance to *R. raciborskii* than to *R. curvata*, *R. curvispora*, or *R. brookii*. Pairwise distance matrix between cyanobacterial strains isolated from summer and winter phytoplankton of Lubosińskie Lake and comparative sequences from GenBank using the Kimura2-parameter model is outlined in Supplementary Materials S2.

### 2.3. Cyanometabolites and Toxigenicity Genes

**Figure 5:**
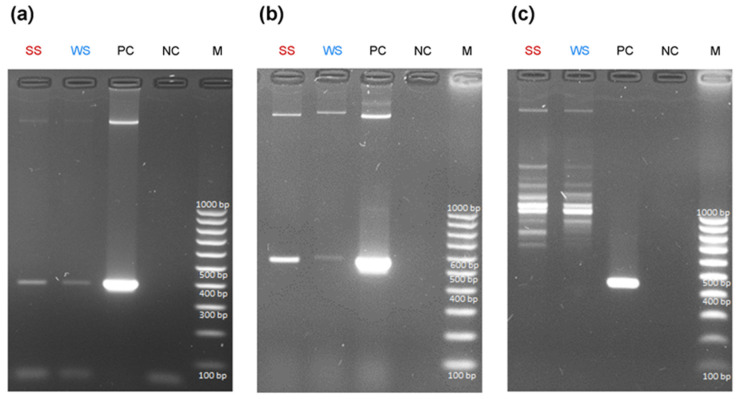
Qualitative amplification of toxigenic genes in summer and winter samples from Lubosińskie Lake: (**a**) *mcy*E (405 bp)—potential for production of MCs; (**b**) *cyr*J (578 bp)—potential for production of CYN; and (**c**) *ana*F (467 bp)—potential for production of ATX-a. SS in red color—summer lake-water sample, WS in blue color—winter lake-water sample, PC—positive control (*mcy*E, *Microcystis aeruginosa* PCC7806; *cyr*J, *Raphidiopsis raciborskii* CS505 Australia; *ana*F, *Cuspidothrix issatschenkoi* NIVA-CYA 711); NC—negative control; M—DNA size marker.

The toxicological screening of water from Lubosińskie Lake using HPLC-DAD revealed the presence of dmMC-RR (10.58 µg/L), MC-YR (1.14 µg/L), and dmMC-LR (1.94 µg/L) in the summer, whereas none of the examined microcystin forms was detected in winter. Summer and winter lake water did not contain APs and STXs, according to the ELISA immunoassay. The results from the HPLC-DAD chromatography and ELISA immunoassay for lake-water samples are presented in Supplementary Materials S3. The toxigenicity assessment showed the presence of *mcy*E and *cyr*J genes both in summer and winter samples, while *ana*F was found in neither (Figure 5).

**Figure 6:**
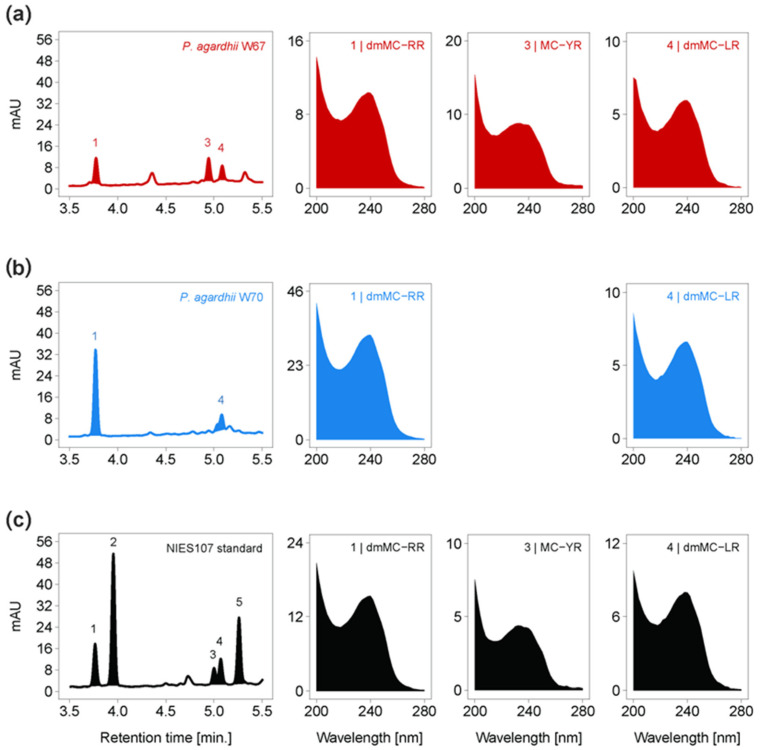
Visualized HPLC-DAD chromatograms and compound spectra of dmMC-RR (1, RT = 3.776 min), MC-YR (3, RT = 4.945 min), and dmMC-LR (4, RT = 5.086 min) in summer *P. agardhii* strain W67 (**a**); dmMC-RR (1, RT = 3.771 min) and dmMC-LR (4, RT = 5.081 min) in winter *P. agardhii* strain W70 (**b**); and dmMC-RR (1, RT = 3.766 min), MC-RR (2, RT = 3.955 min), MC-YR (3, RT = 5.001 min), dmMC-LR (4, RT = 5.071 min), and MC-LR (5, RT = 5.260 min) in the standard NIES107 (**c**). Summer and winter MCs-producing strains are marked, respectively, in red and blue.

**Figure 7:**
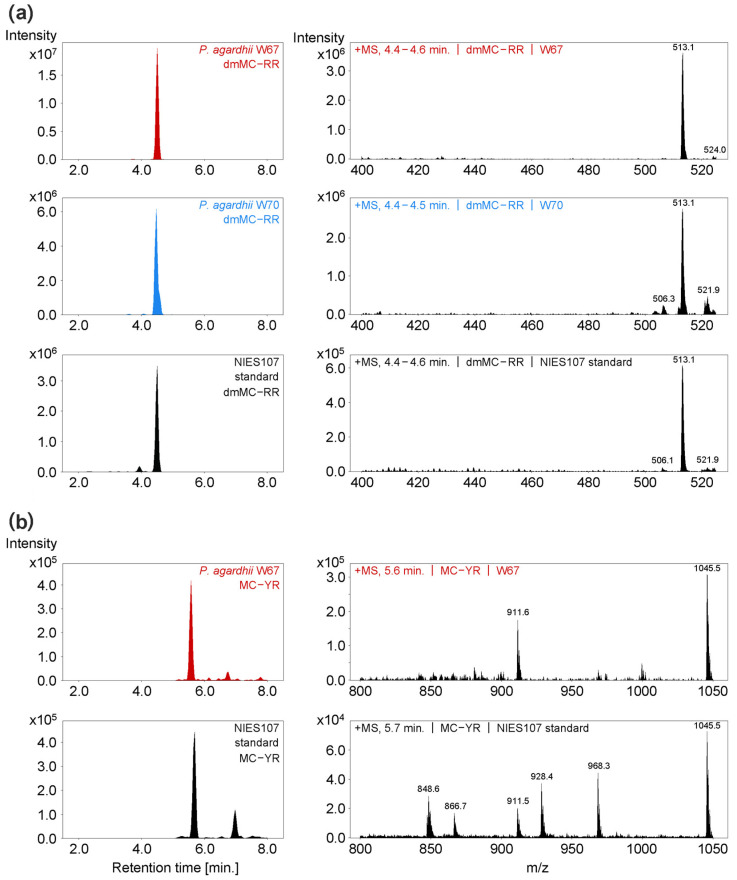
Visualized chromatograms and MS scan spectra of (**a**) dmMC-RR in the summer *P. agardhii* W67 (RT = 4.5 min), winter *P. agardhii* W70 (RT = 4.5 min), and NIES107 standard (RT = 4.5 min); and (**b**) MC-YR in *P. agardhii* W67 (RT = 5.6 min) and NIES107 standard (RT = 5.7 min). Summer and winter MCs-producing strains are marked, respectively, in red and blue.

The results of the toxicological screening of strains are summarized in Table 1. APs were detected in all examined strains of *A. gracile* and *P. agardhii*—the concentration of APs was above the detection limit, 2 µg/L, in each case (Supplementary Materials S4). Strains of *R. raciborskii* were free of APs. The ELISA immunoassay also showed that *P. agardhii* W67 and *A. gracile* W4 and W89 strains contained anatoxin-a, and the concentrations were very close to the lower detection limit. Saxitoxins were not detected by ELISA in the summer or in the winter set of strains. The noncompetitive immunoassay (TRFIA) revealed *P. agardhii* from summer (W67) and winter (W70) to be positive for MCs/NOD, while the test gave negative results in the case of other examined strains (Supplementary Materials S5). The HPLC-DAD showed the presence of dmMC-RR and dmMC-LR in summer and winter isolates of *P. agardhii* (W67 and W70, respectively), and MC-YR in summer strain W67 (Figure 6). Other forms of microcystins were not detected in these strains by this chromatographic method (Supplementary Materials S6). The remaining strains were free of all examined MC forms based on HPLC-DAD. The LC-MS technique confirmed the presence of demethylated forms of MC-RR in the W67 and W70 strains, and MC-YR in W67 (Figure 7), and the absence of other investigated MC forms in these strains (Supplementary Materials S7). Cylindrospermopsin, anatoxin-a, and nodularin were not detected in any of the screened strains by HPLC-DAD and LC-MS (Supplementary Materials S6 and S7, respectively). Screening of strains for genes involved in production of the selected cyanotoxins revealed the presence of *mcy*E gene (potential for synthesis of microcystins) in *P. agardhii* W67 and W70 strains (Supplementary Materials S8). The *cyr*J (gene marker for cylindrospermopsin synthesis) and *ana*F (gene marker for anatoxin-a synthesis) were not detected in any of the examined cyanobacterial strains isolated from the lake.

## 3. Discussion

Cyanobacterial blooms in eutrophic north temperate lakes are a phenomenon that occurs frequently in summer and is becoming increasingly common in winter. Lubosińskie Lake has also experienced cyanobacterial blooms in recent decades due to its eutrophic nature [25,30,31,36]. High chlorophyll-*a* levels, sometimes exceeding 100 µg/L in both summer and winter [25], indicate the high productivity of this lake—conditions suitable for large cyanobacteria blooms [37]. In 2019/2020, Lubosińskie Lake continued to be “bloom friendly” for cyanobacteria, as these organisms were a dominant fraction of the phytoplankton in summer and winter. In previous studies (2006/2008), clear dominance of cyanobacteria (*Planktothrix agardhii*) was also observed in both summer and winter. The potential for the synthesis and actual concentration of microcystins was described, without distinguishing other metabolites of cyanobacteria [25,30].

The present study shows that cyanobacterial community in Lubosińskie Lake had a higher biomass and was richer in bloom-forming and potentially toxic taxa in summer than in winter. In both periods, the surface water was dominated by oscillatorialean cyanobacteria: *Planktothrix* and *Limnothrix* in summer and *Limnothrix* in winter. Members of these genera, especially *P. agardhii* and *L. redekei*, are frequently dominant in eutrophic lakes of temperate zones and often compete with each other (e.g., see [22]). The winter population of cyanobacteria near the surface was clearly dominated by *Limnothrix redekei* and *Limnothrix planctonica* in February 2020, while the biomass of *P. agardhii* decreased significantly. The outcompeting of *P. agardhii* populations by *Limnothrix* sp. was previously reported in other shallow lakes and explained by the ability of *Limnothrix* to grow faster than *P. agardhii* at lower temperatures and limited light supply [22,38,39,40]. Interestingly, over a decade ago, previous studies reported that cyanobacterial blooms in Lubosińskie Lake were dominated by *P. agardhii* during all consecutive years of monitoring from 2006 to 2008 [30], while in contrast, *Limnothrix* spp. were reported to occur but not dominate the phytoplankton communities [25]. A shift in the cyanobacterial community with higher dominance of *Limnothrix* spp. over *P. agardhii* was described for the first time for winter in 2017 [31], dynamics that were also observed for the later winter in 2020 in the present study. More frequent monitoring of this particular lake in the near future, in terms of phytoplankton composition and physical and chemical changes in the water would help to explain the reasons for the observed shifts in species dominance between the seasons.

Summer conditions supported relatively high biomass of several nostocalean cyanobacteria, especially alien and invasive species such as *S. aphanizomenoides*, *R. raciborskii*, and *R. mediterranea*. These species have subcosmopolitan distribution and often constitute a substantial element of summer phytoplankton in eutrophic lakes [41,42,43,44,45,46]. However, the strains of these invasive species were not able to endure winter in Lubosińskie Lake, although it was mild, with a water temperature of approximately 4 °C. The same species were also absent in the lake in the winter of 2017, when ice cover was present [31]. These results indicate the rarity of ecotypes of the species that would be able to actively overwinter in the studied lake, while such ecotypes exist in other temperate lakes of Central Europe (e.g., [47]), and also in lakes on other continents (e.g., [48,49]), and can maintain relatively high biomass during mild winters. To our knowledge, there are no reports of active overwintering of *R. raciborskii* under ice or at temperatures near 0 °C, as some chroococcalean, nostocalean, and oscillatorialean cyanobacteria are capable of that [50]. *R. raciborskii* is a common component of summer–autumn phytoplankton community in numerous temperate lakes (e.g., [43]). This raises the question of what the secrets of the success of *R. raciborskii* and other expansive species are in the already invaded waters of the temperate zone. The collapse of populations of this species in temperate lakes of Central Europe under harsh winter conditions and their subsequent renewal in summer suggest winter dormancy. Investing in the formation of akinetes [51,52] could be an effective strategy of this cyanobacterium in order to persist in the invaded waters. This could be an important reason for the stability of *R. raciborskii* populations in the temperate zone.

Both the summer and winter blooms consisted of potentially toxic cyanobacteria. Toxicological screening revealed the presence of hepatotoxins–microcystins (variants: MC-YR and demethylated forms of MC-RR and MC-LR) in lake water, but only during summer. Other studied cyanometabolites (anabaenopeptins, cylindrospermopsin, microcystins, and saxitoxins) were not detected in the lake during summer or winter. However, molecular analyses revealed the presence of *mcy*E gene, the marker for synthesis of microcystins, and the *cyr*J gene, which is associated with CYN production, in both summer and winter samples. The presence of the *mcy*E and *cyr*J genes suggests that potentially toxigenic MC- and CYN-producing cyanobacteria can survive in the winter community. In the case of MCs, this is also confirmed by the toxicological screening of cyanobacterial strains isolated from the lake, as dmMC-LR and dmMC-RR were detected in the winter isolate of *P. agardhii*. Despite the detection of MC-producing genotypes in winter, the microcystins themself were undetected at that time in Lubosińskie Lake, which may suggest that MC-producing genotypes accounted for a relatively low share in the cyanobacteria community. Considering that the biosynthesis of toxic cyanometabolites is regulated at the level of gene expression, which can vary throughout the season and decrease with decreasing temperatures (e.g., microcystins, [53]), the low efficiency of biosynthesis of a particular toxin could also explain its absence in the lake during winter. In the previous studies, microcystins were detected throughout the years 2006 to 2008 in Lubosińskie Lake [25,30]; however, their concentrations during winter seasons were lower than in the summer, e.g., on average, 17.7 μg/L and 46.9 μg/L, respectively. Moreover, the potential toxigenicity of cyanobacterial blooms dictated by the gene *mcy*E was already observed to persist throughout the entire winter of 2007/2008 and summer of 2008 [30], similar to how it was observed for the present study. Overall, it is evident that the summer cyanobacterial bloom had a greater potential to contaminate lake water with toxic cyanometabolites, while the winter cyanobacterial bloom had a hidden potential for toxicity.

**Figure 8:**
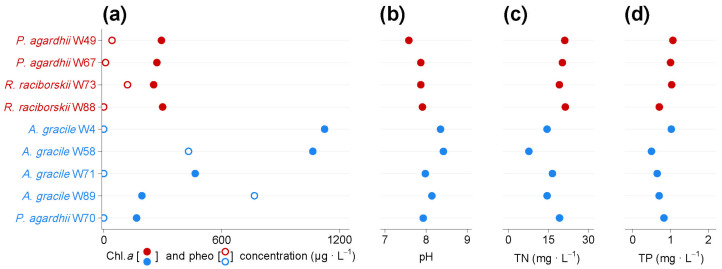
Preliminary data obtained from strain culturing about chlorophyll-*a* (filled circles) and pheophytin (empty circles) concentration (**a**), pH (**b**), total nitrogen concentration (**c**), and total phosphorus concentration (**d**) in 40-day-old cyanobacterial cultures. Red and blue colors indicate the origin of strains (summer and winter, respectively).

The first isolation of strains from the studied lake and their subsequent toxicological evaluation allowed us to gain deeper insight into the toxic potential of the summer and winter cyanobacterial communities in the studied lake. Thanks to this approach, we have also found genotypes that produce anabaenopeptins, which can exert inhibitory effects on various proteases, phosphatases, and carboxypeptidases [54,55,56] and may also play various biological roles for cyanobacteria [57,58,59]. Initial observations of strains and measurements in cultures during their maintenance under laboratory conditions indicate that strains from Lubosińskie Lake exhibit a wide variation in growth, and, at the same time, a high thermal tolerance of the winter-originated cyanobacteria isolates is noted. Originally collected from ~4 °C lake water and subsequently routinely incubating under laboratory conditions at 20 °C, the winter-originated strains are able to achieve similar or even much higher biomass than the summer-originated isolates within 40 days of cultivation (Figure 8). Interestingly, the highest biomass at 20 °C was achieved by two winter isolates of *A. gracile* (W4, W58), although this species constituted only a small part of the winter community. Preliminary data from strain culturing indicate that the winter community may also contain cyanobacteria genotypes which are not psychrotolerant, perhaps having, in fact, summer or autumn origin. This observation raises the question of whether strains that achieve high biomass under summer conditions (“summer-adapted”) can also survive under winter conditions, and vice versa: whether “winter-adapted” strains can persist in the population during summer. It appears that they may, given that cyanobacteria exhibit wide thermal tolerance (e.g., winter strains from this study grew well, even at 20 °C, under laboratory conditions) and the fact that high intra-/interspecific variability of strains may exist within a bloom [60,61,62,63,64].

On the other hand, the lower growth rate of the summer-adapted strains in seemingly favorable conditions might come from their warm-optimized growth strategy aimed at improving the metabolism of reactive oxygen species. It has been demonstrated by Collins [65] and Lindberg and Collins [66] that temperature elevation can cause an increase in reactive oxygen species in phytoplankton organisms, and, hence, warm-adapted strains can reduce the growth rate at a higher temperature. Thus, the great diversity of strains in the population could facilitate the persistence of cyanobacteria as the main component of phytoplankton in eutrophic lakes, which does not decline even under limiting conditions, such as winter. Comparing thermal reaction norms for growth rate between the originated strains from summer and winter conditions is necessary to understand and explain more precisely the succession of cyanobacteria throughout the seasons in the studied lake.

## 4. Conclusions

This study demonstrates that biomass, community structure, and toxicity may differ greatly between summer and winter cyanobacterial blooms. Alien and invasive cyanobacteria, including *Sphaerospermopsis aphanizomenoides*, *Raphidiopsis raciborskii*, and *Raphidiopsis mediterranea*, were not able to actively overwinter in the studied lake even during mild winter. Investigation of strains from both sampling times revealed that toxigenic cyanobacteria appear in both summer and winter cyanobacterial communities. This study also is the second report in recent years demonstrating a dominance of *Limnothrix* over *Planktothrix* in the winter population of cyanobacteria in Lubosińskie Lake. More frequent eco-phyco-toxicological monitoring is required to understand the observed shifts in the cyanobacteria community structure and toxicity throughout the year in this lake and determine how far these changes are shaped by the incidences of mild winters.

## 5. Materials and Methods

### 5.1. Lake Location and Physicochemical Parameters

Lubosińskie Lake (Central–West Poland; 52°31′40″ N, 16°22′56″ E; mean depth = ~2.4 m, max depth = ~6.5 m) was sampled for physicochemical analyses, quantitative analyses of phytoplankton, and toxicological analyses on 2 September 2019 and 11 February 2020. Samples were collected from the lake surface using a bathometer (UWITEC, Mondsee, Austria). Additional plankton tows were collected for isolation of cyanobacteria using a plankton net with a mesh size of 40 µm. The concentrations of total nitrogen, total phosphorus, dissolved nitrogen forms, and total reactive phosphorus were determined spectrophotometrically, using a HACH DR/2010 spectrophotometer (Hach Company, Loveland, CO, USA). During sampling, water temperature, pH, and conductivity were determined in the field using a YSI multiparameter probe 556 MPS (YSI Inc., Yellow Springs, OH, USA), and Secchi depth was measured using a Secchi disc. Phytoplankton was identified and counted using a Fuchs-Rosenthal chamber. At least 400 cells or filaments were counted to reduce the error to less than 10% (*p* = 0.05). The biomass of specimens (fresh weight) was determined based on volumetric analyses of cells, using geometric approximation. Biomass computed in volume units was transposed to fresh biomass, assuming the density of phytoplankton to be 1 [67].

### 5.2. Biological Material

#### 5.2.1. Isolation, Cultivation, and Identification of Cyanobacterial Strains and Sample Analyses

Isolation of cyanobacteria was performed at the Department of Hydrobiology at Adam Mickiewicz University within a few days after field sampling. The process involved picking cyanobacterial trichomes under the inverted-microscope Leica DM IL LED (Leica Microsystems, Wetzlar, Germany), using microcapillaries and their multiple transfers between drops of sterile WC medium [68] with imipenem antibiotic (100 µg/mL) until only a single trichome and no other phytoplankton organisms were visible in a drop of the medium. Subsequently, single trichomes were placed separately in vials filled with 2 mL of culture medium and incubated in a phytotron Conviron (Winnipeg, MB, Canada) at a temperature of 20 ± 0.5 °C, light intensity of 50 μmol photons m/s, and photoperiod of 16 h:8 h (day/night). Successfully growing monoclonal strains were then identified based on their morphological features, following the determination keys [69,70].

To obtain information about the biomass yield of cyanobacterial strains in routine cultivation, strains were inoculated into the 175 cm^2^ cell culture flasks (Nunc™ EasYFlask™, Thermo Scientific, San Jose, CA, USA) filled with 500 mL of WC medium. Cultures were incubated at 20 ± 0.5 °C, photoperiod of 16 h:8 h (light/dark), and light intensity of ~23 µmol photons m/s. After 40 days of incubation, samples (100 mL) were collected and filtered (GF/C filters, Whatman, Maidstone, UK), and, subsequently, chlorophyll-*a* and phaeophytin concentrations were measured according to method ISO 10260:1992 [71]. The remaining filtrates were analyzed using a flow injection analyzer (FIA compact, MLE GmbH, Radebeul, Germany). Samples were filtered through membrane filters (0.45 μm, Sartorius), consequently determining the dissolved forms only. The total phosphorus was measured according to ISO 15681-1:2003 [72]. The total nitrogen method was analyzed according to ISO 29441:2010 [73].

#### 5.2.2. Isolation of DNA and Phylogenetic Characterization of Cyanobacterial Strains

A culture volume of approximately 15 mL was used for DNA isolation. The procedure of DNA isolation was the same as in the case of lake-water samples. The gene *rpo*B (coding for the bacterial β subunit of RNA polymerase; [74]) was used for taxonomic characterization and phylogenetic analysis of the isolated cyanobacterial strains. This gene was selected over the typical gene, 16S rRNA, because it has shown variability in the size of amplicons and is recommended as marker for better differentiation of cyanobacterial taxa [75]. Two sets of primers amplifying the same region of the gene *rpo*B were selected (520–635 bp): (i) rpoBF/rpoBR (5′-ATGTGCCGCGAGGTGAAACCTAAT-3′/5′-RCMGCMGACGAAGAAGACG-3′), targeting cyanobacteria [74]; and (ii) rpoBF1/rpoBF2 (5′-AGGAATTCACCACCACAACT-3′/5′-ACCATCGGCTAATACCTG-3′), targeting cyanobacteria from the order Oscillatoriales [75]. Purification of PCR products was conducted using the QIAEX II Gel Extraction Kit (Qiagen, Hilden, Germany). Samples were sequenced by Genomed (Warsaw, Poland). The nucleotide BLAST tool was used to verify the identity of sequences with other published cyanobacterial strains. Sequences were aligned with the MUSCLE algorithm [76]. The final aligned dataset included 23 sequences, with 9 cyanobacterial strains isolated in the present study and the other 14 published strains obtained from NCBI databases for comparative purposes, with 460 aligned positions. Phylogenetic analyses were constructed with MEGA11 [77], using Neighbor Joining (NJ) and Maximum Likelihood (ML) methods for the *rpo*B aligned dataset. Bootstrap analyses with 1000 replicates (generated trees) were estimated to assess the robustness of the obtained topologies [78]. For ML analysis, the model that best fit the data was estimated with the tool “Find best DNA Models” implemented in MEGA11. The best-fitting model was K2+G+I, a Kimura 2-parameter model [79], and the following parameters were implemented: nucleotide frequencies as f(A), f(T), f(C), and f(G) = 0.250; substitution rate matrix with r(AT), r(AC), r(TA), r(TG), r(CA), r(CG), r(GT), and r(GC) = 0.047; and r(AG), r(TC), r(CT), and r(GA) = 0.156. The proportions of sites were assumed to be invariable = 0, and gamma shape = 0.49. The final tree was constructed with corelDRAW X7 (Corel Corporation, Ottawa, ON, Canada), using the NJ topology and bootstrap support values for NJ and ML analyses obtained in MEGA11.

### 5.3. Toxicological Investigations

#### 5.3.1. Enzyme-Linked Immunosorbent Assay (ELISA)

Lake-water samples (50 mL) were filtered through GF/C filters (Whatman, Maidstone, UK) and quantitatively analyzed for the presence of anabaenopeptins (APs) and saxitoxins (STXs), using Eurofins Abraxis ELISA kits (APs, Product no. 520070; STXs, 52255B; Warminster, PA, USA). Immunoassays were conducted on filtered water samples according to manufacturer protocols.

The ELISA was also used to analyze samples from 21-day-old strain cultures. The biomass of each strain, concentrated in glass vials, was homogenized by 15 min of ultrasonication in ultrasonic bath RK156 (Bandelin Sonorex, Berlin, Germany). Subsequently, ultrasonication (1 min using ultrasonic probe MS73 microtip and Bandelin Sonopuls HD2070 homogenizer, Bandelin electronic GmbH, Berlin, Germany) was applied. Extracted biomass was centrifuged (10 min at 15,000 rpm). Supernatants were then collected and filtered through a syringe filter with a pore diameter of 0.2 µm, with GHP membrane (PALL Corporation, New York, NY, USA), and stored at −20 °C before analysis. Immunoassays were performed on thawed filtrates using Eurofins Abraxis ELISA kits (Warminster, USA) for detection of the APs (Product No. 520070), ATX-a (520060), and STXs (52255B), according to manufacturer protocols.

#### 5.3.2. Noncompetitive Time-Resolved Fluorescence Immunoassay (TRFIA)

The noncompetitive TRFIA was conducted following the assay concept described by Akter et al. [80], but with a slight modification. The assay detects both microcystin and nodularin, and this screening concerned the strains isolated from the lake. A culture volume of 100 mL of each strain was filtered through GF/C filters (Whatman, Maidstone, UK). The filters were then freeze-dried (Alpha 1-4 LSCplus freeze dryer, Martin Christ Gefriertrocknungsanlagen GmbH, Osterode am Harz, Germany) and extracted in 75% methanol by ultrasonication, as in the case of samples for ELISA. The extracts were centrifuged (10 min at 15,000 rpm), and, subsequently, supernatants were collected and evaporated to dryness using a Reacti-VapTM evaporator (ThermoFisher Scientific, Waltham, USA) with nitrogen gas (>99.99%) and a temperature of 50 °C. The reconstitution of dried extracts was performed with 150 µL of Milli-Q water. After reconstitution, the extracts were filtered through GHP Acrodisc 13 mm syringe filters (0.2 µm pore size). Prior to analyses, the prepared samples were stored at −20 °C. The 1x samples represent the extracted cyanotoxin (from original 100 mL culture) in final 150 µL of Milli-Q water. For TRFIA, each sample was diluted in Milli-Q as 1:10, and 25 µL of this diluted sample was used in each well. Briefly, on a 96-well streptavidin-coated plate, 25 µL of Assay Buffer (Kaivogen, Turku, Finland) was added to every well. Then, sample or standard (0.03–100 µg/L of microcystin-LR, prepared in Milli-Q) was added as 25 µL per well, in duplicates. For blank measurement (6 replicates), Milli-Q was added as 25 µL per well. After that, 25 µL of reagent mixture (containing the biotinylated capture antibody, immunocomplex antibody, and the Europium-labeled tracer antibody) was added to each well. The plate was incubated for 1 h, washed four times, and the time-resolved fluorescence signal of Europium was measured. Based on the blank + 3SD signal, the analytical detection limit was around 0.1 µg/L of microcystin-LR. Authentic standard NIES107 containing dmMC-RR, MC-RR, MC-YR, dmMC-LR, and MC-LR; and standard PCC8720 containing dmMC-LR, MC-LR, MC-LY, MC-LW, and MC-LF were used as positive controls in the immunoassay.

#### 5.3.3. High-Performance Liquid Chromatography with Diode Array UV Detection (HPLC-DAD)

Lake-water samples were analyzed for the presence of microcystins (MCs) and cylindrospermopsin (CYN) using the HPLC-DAD system accessible at the UNESCO Chair on Ecohydrology and Applied Ecology at the University of Lodz (Poland). Biomass of cyanobacteria in a known volume was collected on Whatman GF/C filters. Further processing of the samples and chromatographic separation were conducted using the same instruments, equipment, and methodology as described previously [44]. Identification of cyanometabolites in the samples was based on the comparison of the retention times and UV spectra of the compounds to the standards (absorption maximum at 238 nm for MCs and at 262 nm for CYN). For calibration of HPLC analysis, MC-LR, MC-YR, MC-RR, and CYN standards provided by the TOXIC project (European Commission; contract number EVK1-CT-2002-00107) were used.

Cyanobacterial strains isolated from the lake were screened for the presence of ATX-a, CYN, MCs, and NOD using the Agilent 1100 series HPLC system (Agilent Technologies, Waldbronn, Germany) accessible at the Biochemistry and Cell Biology, Faculty of Science and Engineering, Åbo Akademi University (Finland). Samples were processed using the same technique, instruments, and equipment as in the case of TRFIA immunoassay. The reconstitution of dried extracts was performed with either 150 µL of 75% methanol (MCs and NOD analyses) or 150 µL of Milli-Q water (CYN). After reconstitution, the extracts were filtered through GHP Acrodisc 13 mm syringe filters (0.2 µm pore size), and the prepared samples were stored at −20 °C until the analysis. Chromatographic separation of MCs and NOD was conducted on a Supelco Ascentis RP-Amide column (100 mm × 4 mm I.D., with 3 μm particles protected by a 4 × 2 mm C8 guard column), and ATX-a and CYN on a Merck Purospher Star RP-18e column (55 mm × 4 mm I.D., with 3 μm particles protected by a 4 × 4 mm C18 guard column). The mobile phase was the same as in the case of analyses of lake-water samples: solvent A was water, and solvent B was acetonitrile (both solvents with added 0.05% trifluoroacetic acid). The flow rate and linear gradient programs for particular cyanometabolite analyses were as follows: ATX-a and CYN = 0 min of 1% B, 20 min of 70% B, 22 min of 70% B, 22.1 min of 1% B, stop time of 30 min, and flow rate of 1 mL/min; and MCs and NOD = 0 min of 25% B, 7 min of 70% B, 10 min of 70% B, 10.1 min of 25% B, stop time of 15 min, and flow rate of 1 mL/min. Identification of cyanometabolites was conducted using chromatography data system ChemStation (Agilent Technologies, Waldbronn, Germany) and involved comparing the retention times and UV absorption spectra of the compounds to the standards. CYN and NOD were acquired from National Research Council (Halifax, N.S., Canada); ATX-a and MCs were present in-house standards prepared from cyanobacterial cultures ANA123 (ATX-a), NIES (MCs), and PCC7820 (MCs) originating from Finnish Environment Institute (Helsinki, Finland), National Institute for Environmental Studies (Tsukuba, Japan), and Pasteur Culture Collection of Cyanobacteria (Paris, France), respectively.

#### 5.3.4. Liquid Chromatography–Mass Spectrometry (LC-MS)

Liquid chromatography coupled with mass spectrometry (LC-MS) was conducted on the samples from the strains using an Agilent 1200 Rapid Resolution LC coupled to a Bruker (Bruker Daltonik GmbH, Bremen, Germany) HCT ultra-ion-trap mass spectrometer with electrospray ion source. Separation of the cyanometabolites was conducted at 40 °C on a Supelco Ascentis C18 column (50 mm × 3 mm I.D. column, with 3 µm particles protected by a 4 × 2 mm C8 guard column). Extracts were injected in a volume of 5 µL, and the flow rate was 0.5 mL/min. The mobile phase was 99% water–1% acetonitrile–0.1% formic acid (solvent A) and 100% acetonitrile–0.1% formic acid (solvent B). The linear gradient solvent programs employed for each cyanotoxin were as follows: ATX-a = 0 min of 0% B, 5 min of 0% B, 7 min of 70% B, 9 min of 70% B, 9.1 min of 0% B, and stop time of 16 min; CYN, MCs, and NOD = 0 min of 0% B, 2 min of 0% B, 2.1 min of 25% B, 12 min of 70% B, 14 min of 70% B, 14.1 min of 0% B, and stop time of 21 min. The mass spectra were observed in the positive electrospray mode, with a dry temperature of 350 °C, dry gas flow of 10 L/min, nebulizer pressure of 40 psi, and capillary voltage of 4 kV. The MS scan range was *m*/*z* 160–190 (ATX-a), *m*/*z* 400–525 (CYN), and *m*/*z* 800–1050 (MCs and NOD). Identification of cyanotoxins was conducted using Compass 1.3 for HCT/esquire software. Retention times and the observed *m*/*z* values of the compounds were compared to the standards.

#### 5.3.5. Isolation of DNA and Toxigenicity Assessments

DNA isolation was conducted on lake-water samples (100 mL) and on the samples from strain cultures (15 mL). Samples were filtered through nitrocellulose membrane filters (a pore diameter = 0.45 µm; Millipore, Burlington, MA, USA) and processed as described previously [81]. Isolated DNA was adopted as the template for qualitative (PCR, polymerase chain reaction) determination of the following genetic elements: *ana*F (467bp), *cyr*J (578bp), and *mcy*E (405bp) (Table 2).

## Figures and Tables

**Table 1:** Summary of the toxicological screening of summer and winter lake-water samples and cyanobacterial strains isolated from summer and winter phytoplankton of Lubosińskie Lake. Note: “+” means a given cyanometabolite or gene involved in its synthesis was detected; “(f+)” means false positive result; “NA”—missing data. Lack of symbol means the absence of a given cyanometabolite or gene responsible for its synthesis.

Cyanometabolite	Method	Summer Sampling				Winter Sampling				
		*P. agardhii* W49	*P. agardhii* W67	*R. raciborskii* W73	*R. raciborskii* W88	*A. gracile* W4	*A. gracile* W58	*A. gracile* W71	*A. gracile* W89	*P. agardhii* W70
APs	ELISA	+	+			+	+	+	+	+
ATX-a	ELISA		(f+)			(f+)			(f+)	
	HPLC-DAD						NA			
	LC-MS						NA			
	*ana*F gene									
CYN	HPLC-DAD						NA			
	LC-MS						NA			
	*cyr*J gene									
MCs	HPLC-DAD		+				NA			+
	LC-MS		+				NA			+
	*mcy*E gene		+							+
NOD	HPLC-DAD						NA			
	LC-MS						NA			
MCs/NOD	TRFIA		+				NA			+
STXs	ELISA									

**Table 2:** Primers used in the present study.

Gene	Associated Metabolite	Primer Name [Primer Sequence (5′–3′)]	Annealing Temperature	Reference
*ana*F	ATX-a	Atoaf [TCGGAAGCGCGATCGCAAAT] Atxar [GCTTCCTGAGAAGGTCCGCT]	57 °C	[82]
*cyr*J	CYN	cynsulfF [ACTTCTCTCCTTTCCCTATC] cylnamR [GAGTGAAAATGCGTAGAACTTG]	57 °C	[83]
*mcy*E	MCs	mcyE-F2 [GAAATTTGTGTAGAAGGTGC] mcyE-R4 [AATTCTAAAGCCCAAAGACG]	56 °C	[84]

## Data Availability

Data are provided in the Supplementary Materials of this article. More information could be provided upon request.

## Supplementary Materials

The following supporting information can be downloaded at https://www.mdpi.com/article/10.3390/toxins16080357/s1, Supplementary Information S1: Biomasses of phytoplankton taxa in Lubosińskie Lake during summer (2 September 2019) and winter (11 February 2020); Supplementary Information S2: Pairwise distance matrix between cyanobacterial strains isolated from summer and winter phytoplankton of Lubosińskie Lake and comparative sequences from GenBank, using the Kimura2-parameter model; Supplementary Information S3: The results of HPLC-DAD chromatography and ELISA immunoassay for lake-water samples; Supplementary Information S4: The results of ELISA immunoassay for extracts from cyanobacterial strains; Supplementary Information S5: The results of TRFIA immunoassay for extracts from cyanobacterial strains; Supplementary Information S6: The results of HPLC-DAD for extracts from cyanobacterial strains; Supplementary Information S7: The results of LC-MS for extracts from cyanobacterial strains; Supplementary Information S8: The results of toxigenicity assessment for extracts of summer and winter cyanobacterial strains from Lubosińskie Lake.
