# Supplementary material for: Community Structure and Toxicity Potential of Cyanobacteria during Summer and Winter in a Temperate-Zone Lake Susceptible to Phytoplankton Blooms
Source: Toxins (Basel). 2024 Aug 14;16(8):357. doi: 10.3390/toxins16080357 (PMC11359657; doi:10.3390/toxins16080357)
Supplement: Supplementary file 1 [file toxins-16-00357-s001.zip › S2.pdf]

Community Structure and Toxicity Potential of Cyanobacteria during Summer and Winter in a Temperate-Zone Lake Susceptible to Phytoplankton Blooms

Łukasz Wejnerowski[1*], Tamara Dulić[2], Sultana Akter[3], Arnoldo Font-Nájera[4], Michał Rybak[5], Oskar Kamiński[1], Anna Czerepska[1], Marcin Krzysztof Dziuba[6], Tomasz Jurczak[7], Jussi Meriluoto[2*], Joanna Mankiewicz-Boczek[7], Mikołaj Kokociński[1]

[1] Department of Hydrobiology, Institute of Environmental Biology, Faculty of Biology, Adam Mickiewicz University, Uniwersytetu Poznańskiego 6, 61-614 Poznań, Poland;

[2] Biochemistry and Cell Biology, Faculty of Science and Engineering, Åbo Akademi University, Tykistökatu 6A, 20520 Turku, Finland;

[3] Biotechnology, Department of Life Technologies, Faculty of Technology, University of Turku, 20520 Turku, Finland;

[4] European Regional Centre for Ecohydrology of the Polish Academy of Sciences, Tylna 3, 90-364 Łódź, Poland;

[5] Department of Water Protection, Institute of Environmental Biology; Faculty of Biology; Adam Mickiewicz University; Uniwersytetu Poznańskiego 6, 61-614 Poznań, Poland;

[6] Department of Ecology and Evolutionary Biology, University of Michigan; MI 48109 Ann Arbor, USA;

[7] University of Lodz, Faculty of Biology and Environmental Protection, UNESCO Chair on Ecohydrology and Applied Ecology; Banacha 12/16, 90-237 Łódź, Poland;

Correspondence: wejner@amu.edu.pl (Ł.W.); Jussi.Meriluoto@abo.fi (J.M.)

Supplementary Information S2

Pairwise distance matrix between cyanobacterial strains isolated from summer and winter phytoplankton of Lubosińskie Lake and comparative sequences from GenBank using the Kimura2-parameter model

List of examined strains of cyanobacteria from Lubosińskie Lake

Origin	Strain	Identification based on morphology
Summer period 02.09.2019	W67	*Planktothrix agardhii*
	W49	*Planktothrix agardhii*
	W88	*Raphidiopsis raciborskii*
	W73	*Raphidiopsis raciborskii*
Winter period 11.02.2020	W70	*Planktothrix agardhii*
	W58	*Aphanizomenon gracile*
	W89	*Aphanizomenon gracile*
	W4	*Aphanizomenon gracile*
	W71	*Aphanizomenon gracile*

Pairwise distance matrix between cyanobacterial strains isolated from summer and winter phytoplankton of Lubosińskie Lake and comparative sequences from GenBank using the Kimura2-parameter model

	Raphidiopsis curvata NIES-932	*Raphidiopsis curvispora* GIHE-G1	*Raphidiopsis raciborskii* CS-506	*Raphidiopsis brookii* D9	*Aphanizomenon flos-aquae* 2012/KM1/D3	*Aphanizomenon flos-aquae* KM1D3-PB	*Cuspidothrix issatschenkoi* CHARLIE-1	*Planktothrix agardhii* NIVA-CYA 126	*Planktothrix agardhii* PCC7811	*Planktothrix agardhii* PCC10110	*Microcystis aeruginosa* PCC7806	*Planktothrix agardhii* W70	*Aphanizomenon gracile* W58	*Aphanizomenon gracile* W89	*Aphanizomenon gracile* W4	*Aphanizomenon gracile* W71	*Planktothrix agardhii* W67	*Planktothrix agardhii* W49	*Raphidiopsis raciborskii* W88	*Raphidiopsis raciborskii* W73	*Thermus thermophilus* AK1	*Aphanizomenon gracile* HIANEY	*Aphanizomenon gracile* 1tu26s16
Raphidiopsis curvata NIES-932																							
Raphidiopsis curvispora GIHE-G1	0.011																						
Raphidiopsis raciborskii CS-506	0.031	0.026																					
Raphidiopsis brookii D9	0.040	0.034	0.025																				
Aphanizomenon flos-aquae 2012/KM1/D3	0.317	0.322	0.312	0.322																			
Aphanizomenon flos-aquae KM1D3-PB	0.317	0.322	0.312	0.322	0.000																		
Cuspidothrix issatschenkoi CHARLIE-1	0.282	0.286	0.273	0.273	0.171	0.171																	
Planktothrix agardhii NIVA-CYA 126	0.449	0.455	0.461	0.441	0.393	0.393	0.455																
Planktothrix agardhii PCC7811	0.449	0.455	0.461	0.441	0.393	0.393	0.455	0.000															
Planktothrix agardhii PCC10110	0.449	0.455	0.461	0.441	0.393	0.393	0.455	0.000	0.000														
Microcystis aeruginosa PCC7806	0.460	0.467	0.490	0.468	0.418	0.418	0.449	0.482	0.482	0.482													
Planktothrix agardhii W70	0.439	0.445	0.438	0.418	0.372	0.372	0.439	0.024	0.024	0.024	0.482												
Aphanizomenon gracile W58	0.316	0.316	0.312	0.312	0.053	0.053	0.168	0.466	0.466	0.466	0.411	0.443											
Aphanizomenon gracile W89	0.316	0.316	0.312	0.312	0.053	0.053	0.168	0.466	0.466	0.466	0.411	0.443	0.000										
Aphanizomenon gracile W4	0.316	0.316	0.312	0.312	0.053	0.053	0.168	0.466	0.466	0.466	0.411	0.443	0.000	0.000									
Aphanizomenon gracile W71	0.316	0.316	0.312	0.312	0.053	0.053	0.168	0.466	0.466	0.466	0.411	0.443	0.000	0.000	0.000								
Planktothrix agardhii W67	0.449	0.455	0.461	0.441	0.393	0.393	0.455	0.000	0.000	0.000	0.482	0.024	0.466	0.466	0.466	0.466							
Planktothrix agardhii W49	0.449	0.455	0.461	0.441	0.393	0.393	0.455	0.000	0.000	0.000	0.482	0.024	0.466	0.466	0.466	0.466	0.000						
Raphidiopsis raciborskii W88	0.031	0.026	0.000	0.025	0.312	0.312	0.273	0.461	0.461	0.461	0.490	0.438	0.312	0.312	0.312	0.312	0.461	0.461					
Raphidiopsis raciborskii W73	0.031	0.026	0.000	0.025	0.312	0.312	0.273	0.461	0.461	0.461	0.490	0.438	0.312	0.312	0.312	0.312	0.461	0.461	0.000				
Thermus thermophilus AK1	0.786	0.774	0.807	0.817	0.807	0.807	0.823	0.830	0.830	0.830	0.644	0.864	0.802	0.802	0.802	0.802	0.830	0.830	0.807	0.807			
Aphanizomenon gracile HIANEY	0.317	0.322	0.303	0.322	0.008	0.008	0.160	0.392	0.392	0.392	0.419	0.372	0.056	0.056	0.056	0.056	0.392	0.392	0.303	0.303	0.817		
Aphanizomenon gracile 1tu26s16	0.321	0.326	0.307	0.317	0.017	0.017	0.160	0.403	0.403	0.403	0.424	0.382	0.047	0.047	0.047	0.047	0.403	0.403	0.307	0.307	0.820	0.008	
