# Supplementary material for: Community Structure and Toxicity Potential of Cyanobacteria during Summer and Winter in a Temperate-Zone Lake Susceptible to Phytoplankton Blooms
Source: Toxins (Basel). 2024 Aug 14;16(8):357. doi: 10.3390/toxins16080357 (PMC11359657; doi:10.3390/toxins16080357)
Supplement: Supplementary file 1 [file toxins-16-00357-s001.zip › S3.pdf]

Community Structure and Toxicity Potential of Cyanobacteria during Summer and Winter in a Temperate-Zone Lake Susceptible to Phytoplankton Blooms

Łukasz Wejnerowski[1*], Tamara Dulić[2], Sultana Akter[3], Arnoldo Font-Nájera[4], Michał Rybak[5], Oskar Kamiński[1], Anna Czerepska[1], Marcin Krzysztof Dziuba[6], Tomasz Jurczak[7], Jussi Meriluoto[2*], Joanna Mankiewicz-Boczek[7], Mikołaj Kokociński[1]

[1] Department of Hydrobiology, Institute of Environmental Biology, Faculty of Biology, Adam Mickiewicz University, Uniwersytetu Poznańskiego 6, 61-614 Poznań, Poland;

[2] Biochemistry and Cell Biology, Faculty of Science and Engineering, Åbo Akademi University, Tykistökatu 6A, 20520 Turku, Finland;

[3] Biotechnology, Department of Life Technologies, Faculty of Technology, University of Turku, 20520 Turku, Finland;

[4] European Regional Centre for Ecohydrology of the Polish Academy of Sciences, Tylna 3, 90-364 Łódź, Poland;

[5] Department of Water Protection, Institute of Environmental Biology; Faculty of Biology; Adam Mickiewicz University; Uniwersytetu Poznańskiego 6, 61-614 Poznań, Poland;

[6] Department of Ecology and Evolutionary Biology, University of Michigan; MI 48109 Ann Arbor, USA;

[7] University of Lodz, Faculty of Biology and Environmental Protection, UNESCO Chair on Ecohydrology and Applied Ecology; Banacha 12/16, 90-237 Łódź, Poland;

Correspondence: wejner@amu.edu.pl (Ł.W.); Jussi.Meriluoto@abo.fi (J.M.)

Supplementary Information S3

The results of HPLC-DAD chromatography and ELISA immunoassay for lake water samples

HPLC-DAD
CYN

Chromatogram of CYN standard and UV spectrum of CYN

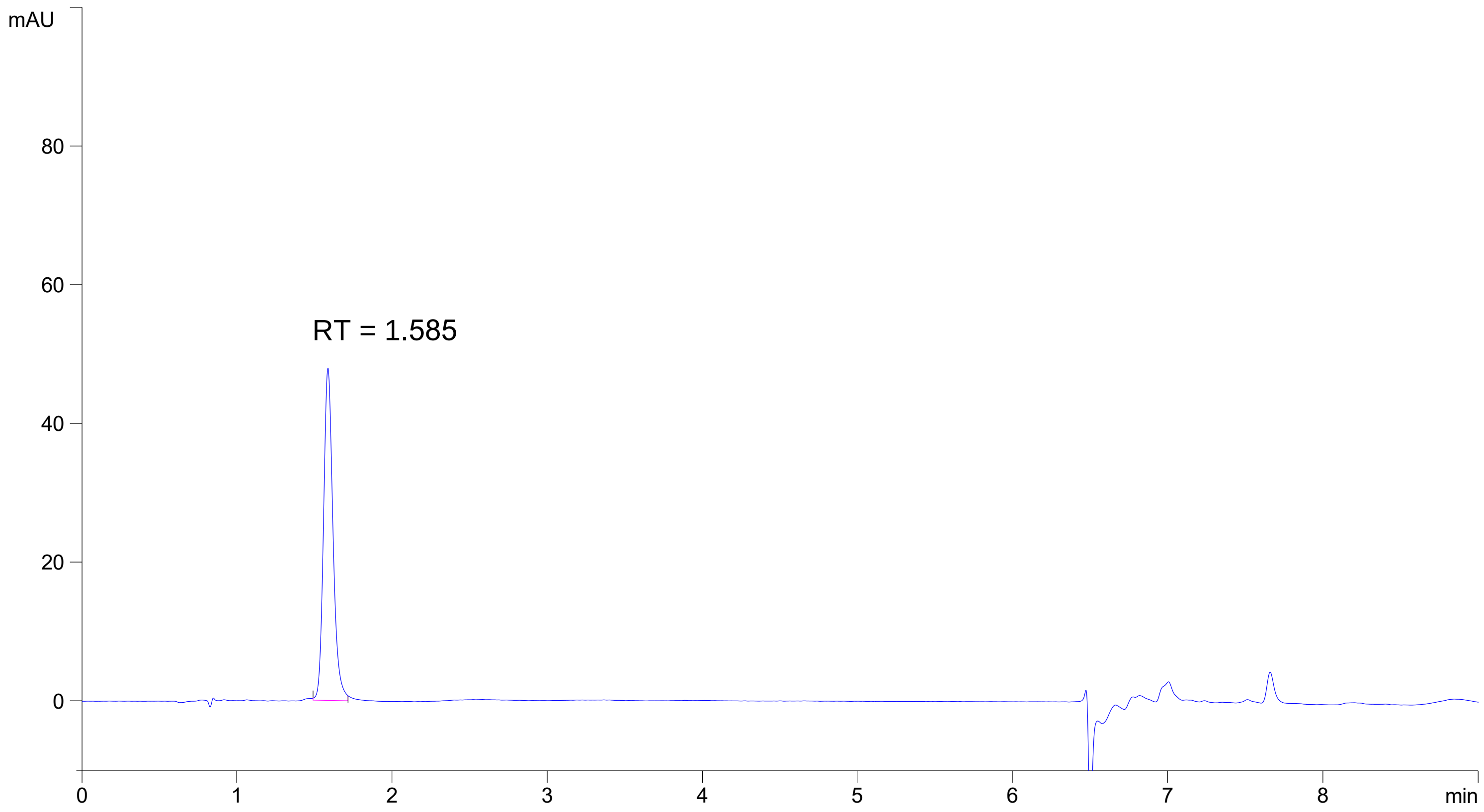

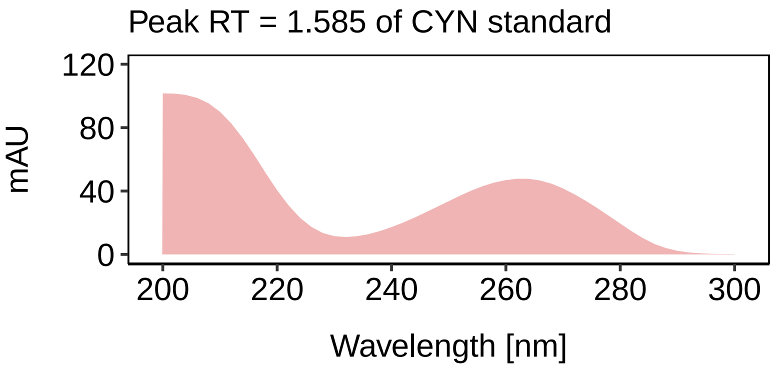

Chromatogram of summer sample from Lubosińskie Lake

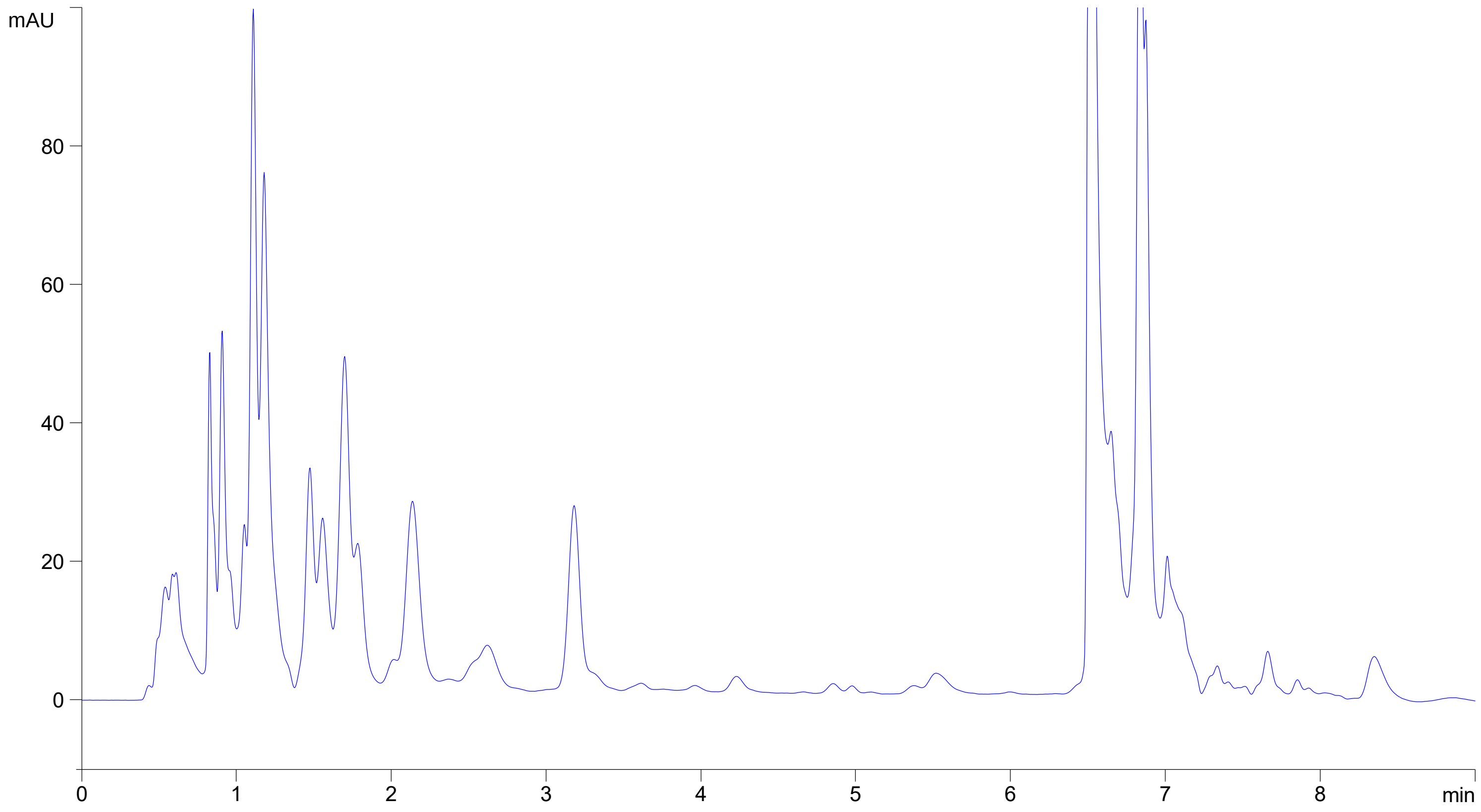

Chromatogram of winter sample from Lubosińskie Lake

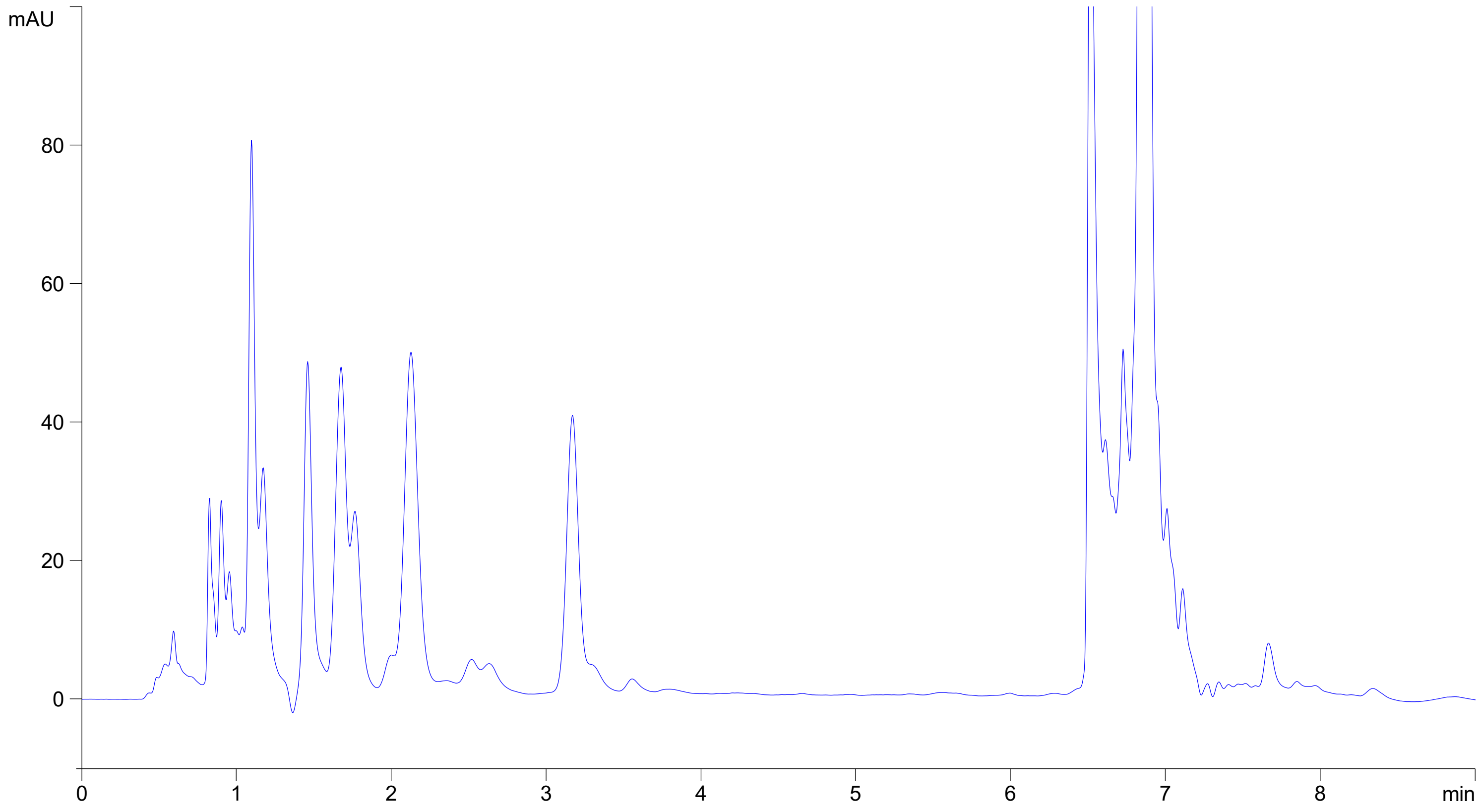

Compounds of similar RT to CYN had a spectrum that did not match the spectrum of the standard

HPLC-DAD
MCs

Chromatogram of MC-RR, MC-YR, MC-LR standard and their UV spectra

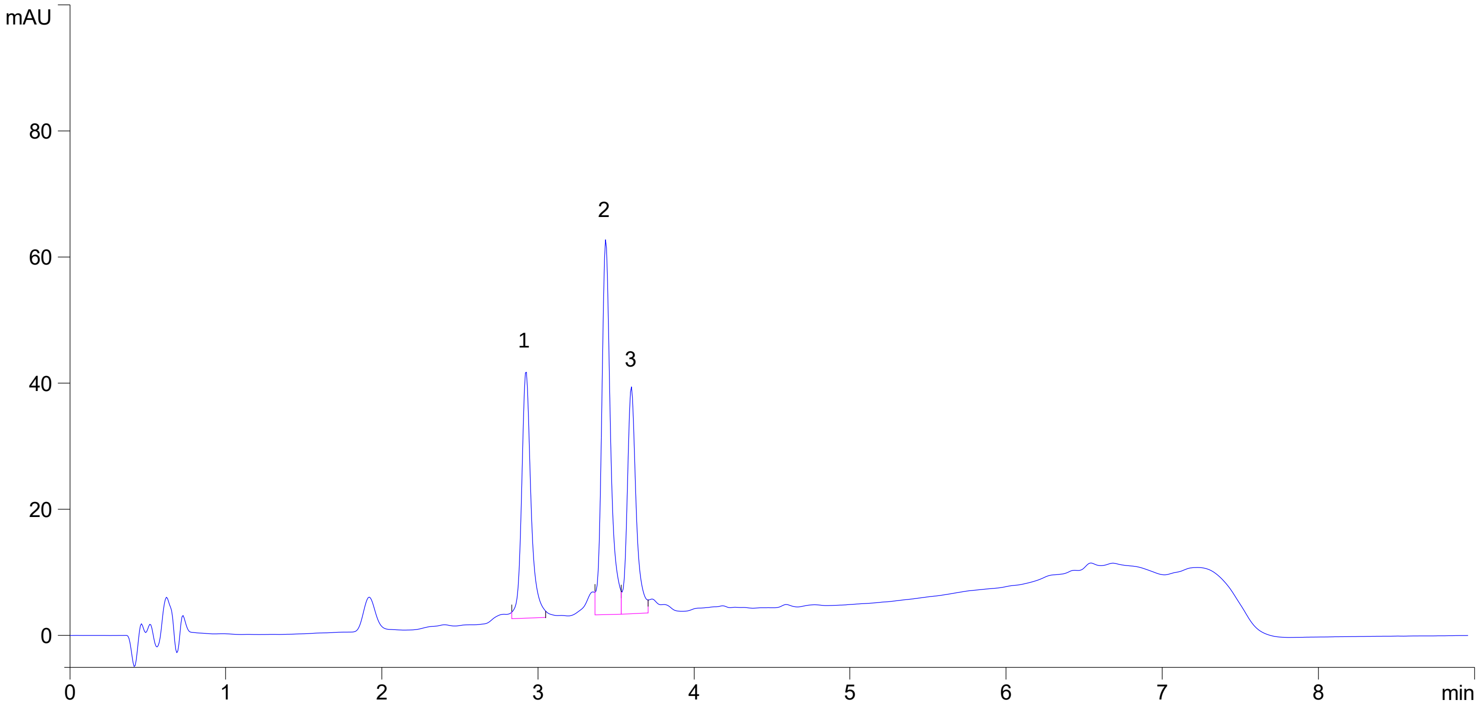

1 - Peak RT = 2.921 of MC−RR standard

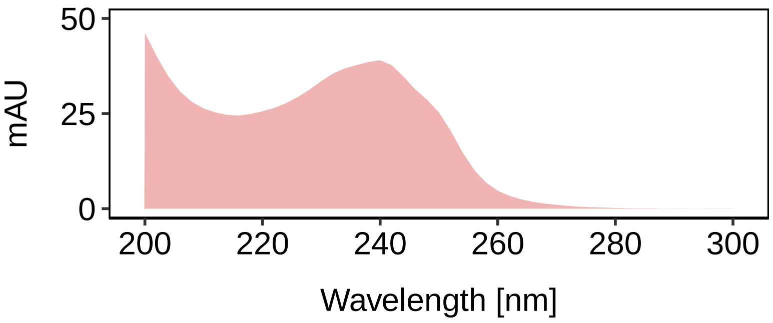

2 - Peak RT = 3.432 of MC−YR standard

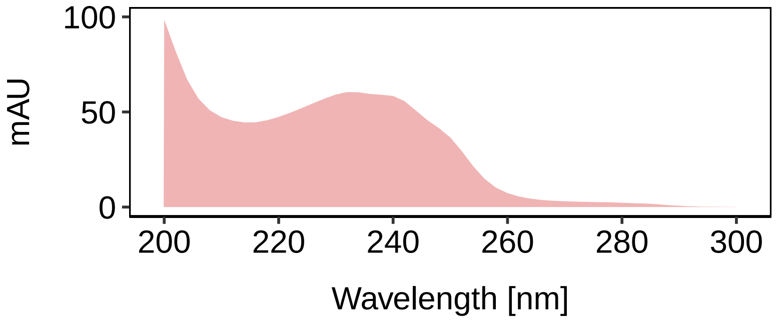

3 - Peak RT = 3.596 of MC−LR standard

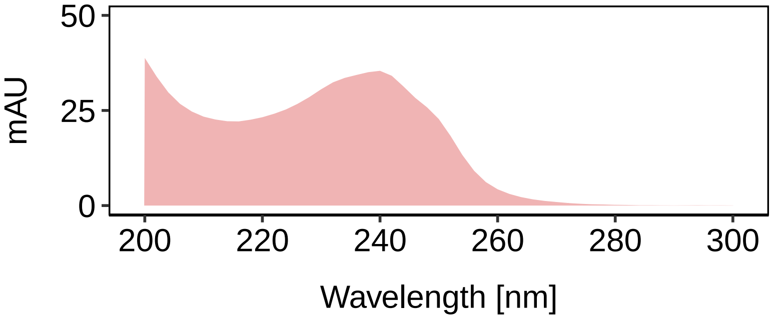

Chromatogram of summer sample from Lubosińskie Lake and selected UV spectra

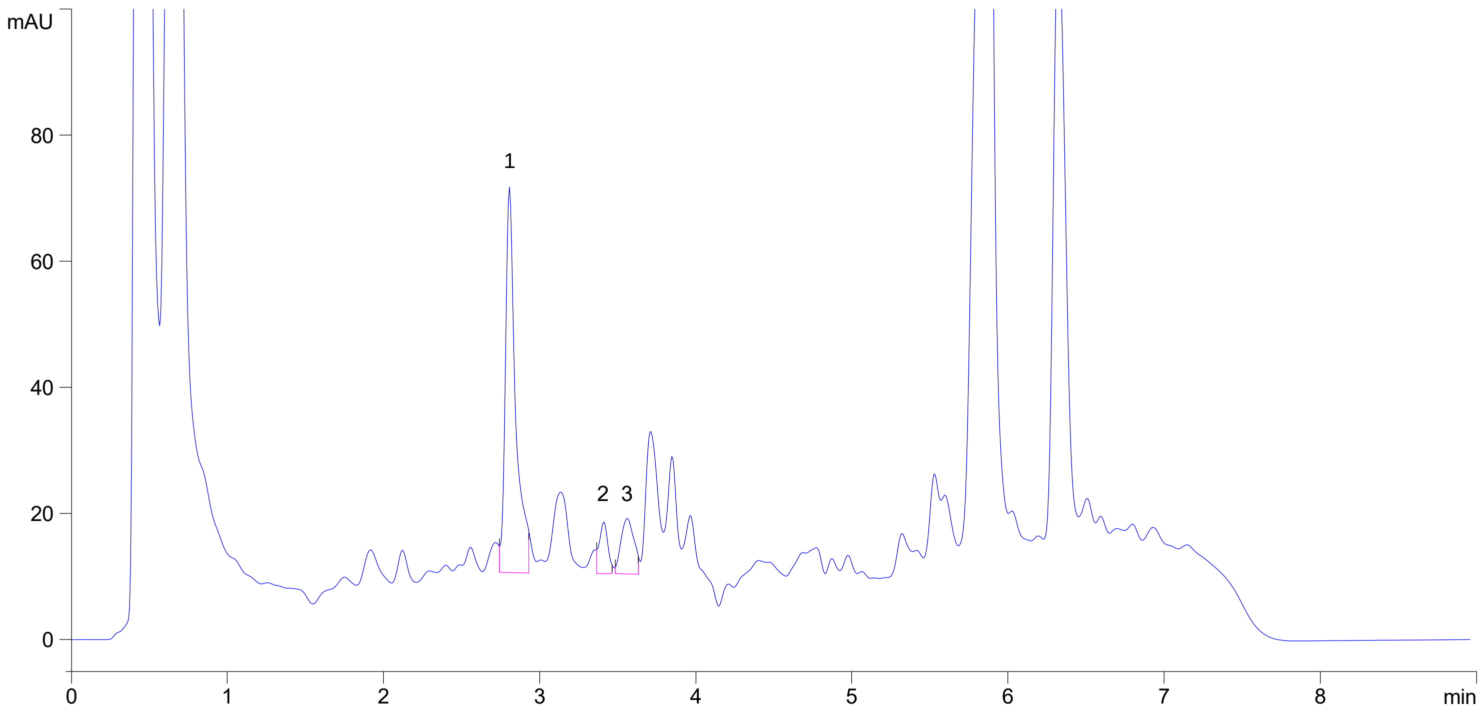

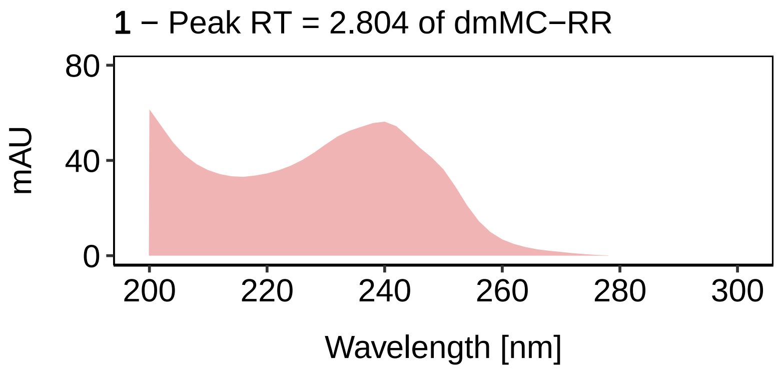

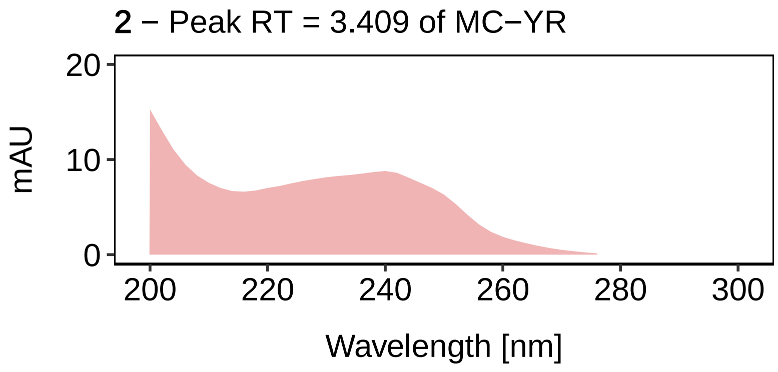

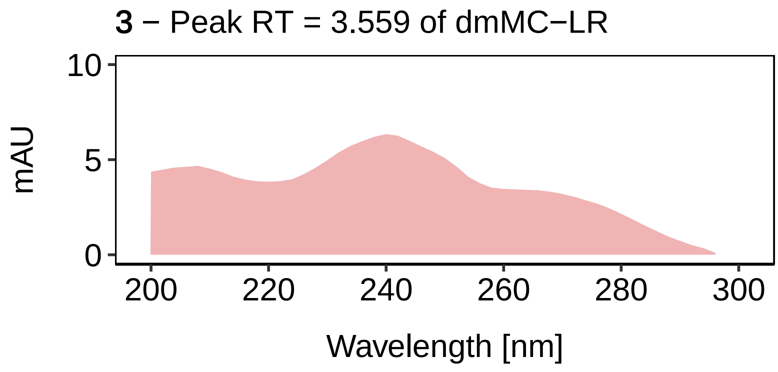

Chromatogram of winter sample from Lubosińskie Lake

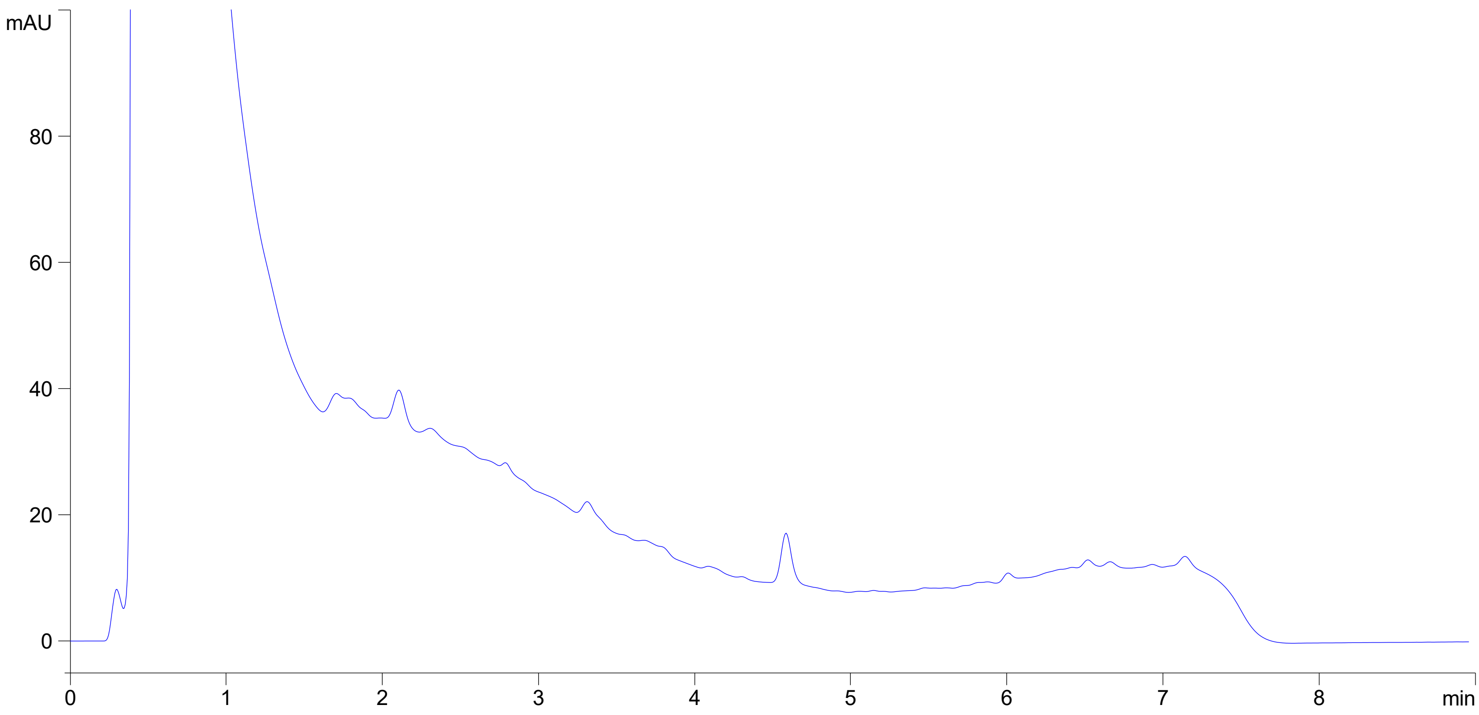

ELISA
immunoassay

A)

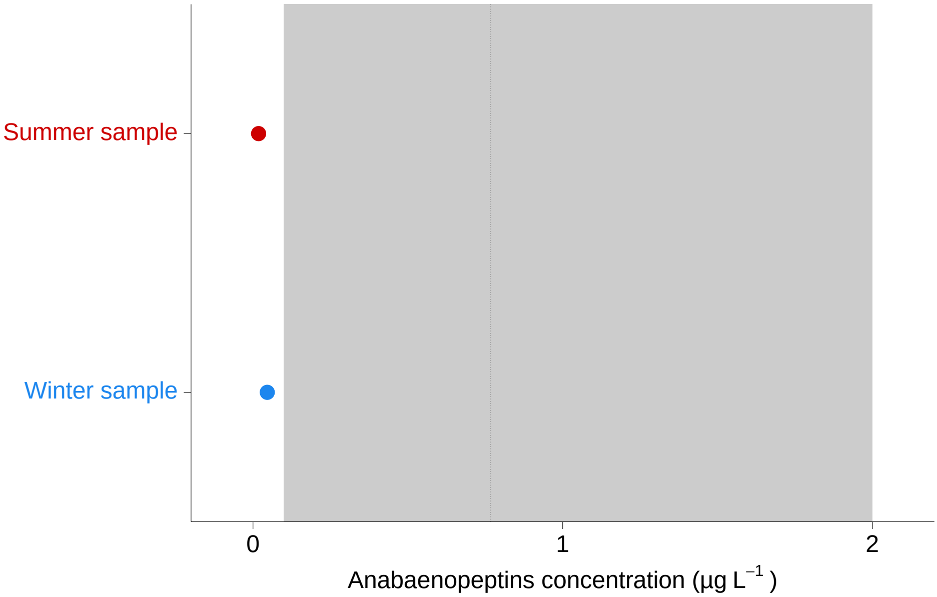
Summer sample
Winter sample
0
1
2
Anabaenopeptins concentration (µg L⁻¹)

B)

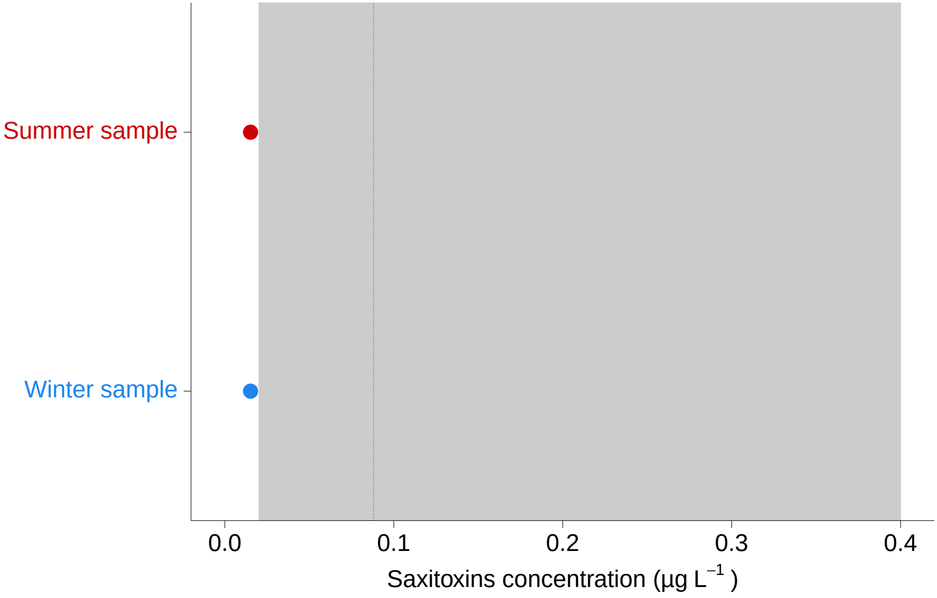
Summer sample
Winter sample
0.0
0.1
0.2
0.3
0.4
Saxitoxins concentration (µg L⁻¹)
