# Supplementary material for: Community Structure and Toxicity Potential of Cyanobacteria during Summer and Winter in a Temperate-Zone Lake Susceptible to Phytoplankton Blooms
Source: Toxins (Basel). 2024 Aug 14;16(8):357. doi: 10.3390/toxins16080357 (PMC11359657; doi:10.3390/toxins16080357)
Supplement: Supplementary file 1 [file toxins-16-00357-s001.zip › S4.pdf]

Community Structure and Toxicity Potential of Cyanobacteria during Summer and Winter in a Temperate-Zone Lake Susceptible to Phytoplankton Blooms

Łukasz Wejnerowski[1*], Tamara Dulić[2], Sultana Akter[3], Arnoldo Font-Nájera[4], Michał Rybak[5], Oskar Kamiński[1], Anna Czerepska[1], Marcin Krzysztof Dziuba[6], Tomasz Jurczak[7], Jussi Meriluoto[2*], Joanna Mankiewicz-Boczek[7], Mikołaj Kokociński[1]

[1] Department of Hydrobiology, Institute of Environmental Biology, Faculty of Biology, Adam Mickiewicz University, Uniwersytetu Poznańskiego 6, 61-614 Poznań, Poland;

[2] Biochemistry and Cell Biology, Faculty of Science and Engineering, Åbo Akademi University, Tykistökatu 6A, 20520 Turku, Finland;

[3] Biotechnology, Department of Life Technologies, Faculty of Technology, University of Turku, 20520 Turku, Finland;

[4] European Regional Centre for Ecohydrology of the Polish Academy of Sciences, Tylna 3, 90-364 Łódź, Poland;

[5] Department of Water Protection, Institute of Environmental Biology; Faculty of Biology; Adam Mickiewicz University; Uniwersytetu Poznańskiego 6, 61-614 Poznań, Poland;

[6] Department of Ecology and Evolutionary Biology, University of Michigan; MI 48109 Ann Arbor, USA;

[7] University of Lodz, Faculty of Biology and Environmental Protection, UNESCO Chair on Ecohydrology and Applied Ecology; Banacha 12/16, 90-237 Łódź, Poland;

Correspondence: wejner@amu.edu.pl (Ł.W.); Jussi.Meriluoto@abo.fi (J.M.)

Supplementary Information S4

The results of ELISA immunoassay for extracts from cyanobacterial strains

Figure description: The results of ELISA immunoassay for the presence of anabaenopeptins (**A**), anatoxin-a (**B**), and saxitoxins (**C**) in the strains isolated from summer (red) and winter (blue) cyanosphere of Lubosińskie Lake. Circles indicate the concentration of a given toxin. Detection limit is demonstrated by the grey area. White vertical line indicates the concentration of the cyanotoxin in positive control that should be 0.75 μg L^{-1} (anabaenopeptins), 0.75 μg L^{-1} (anatoxin-a), 0.075 μg L^{-1} (saxitoxins).

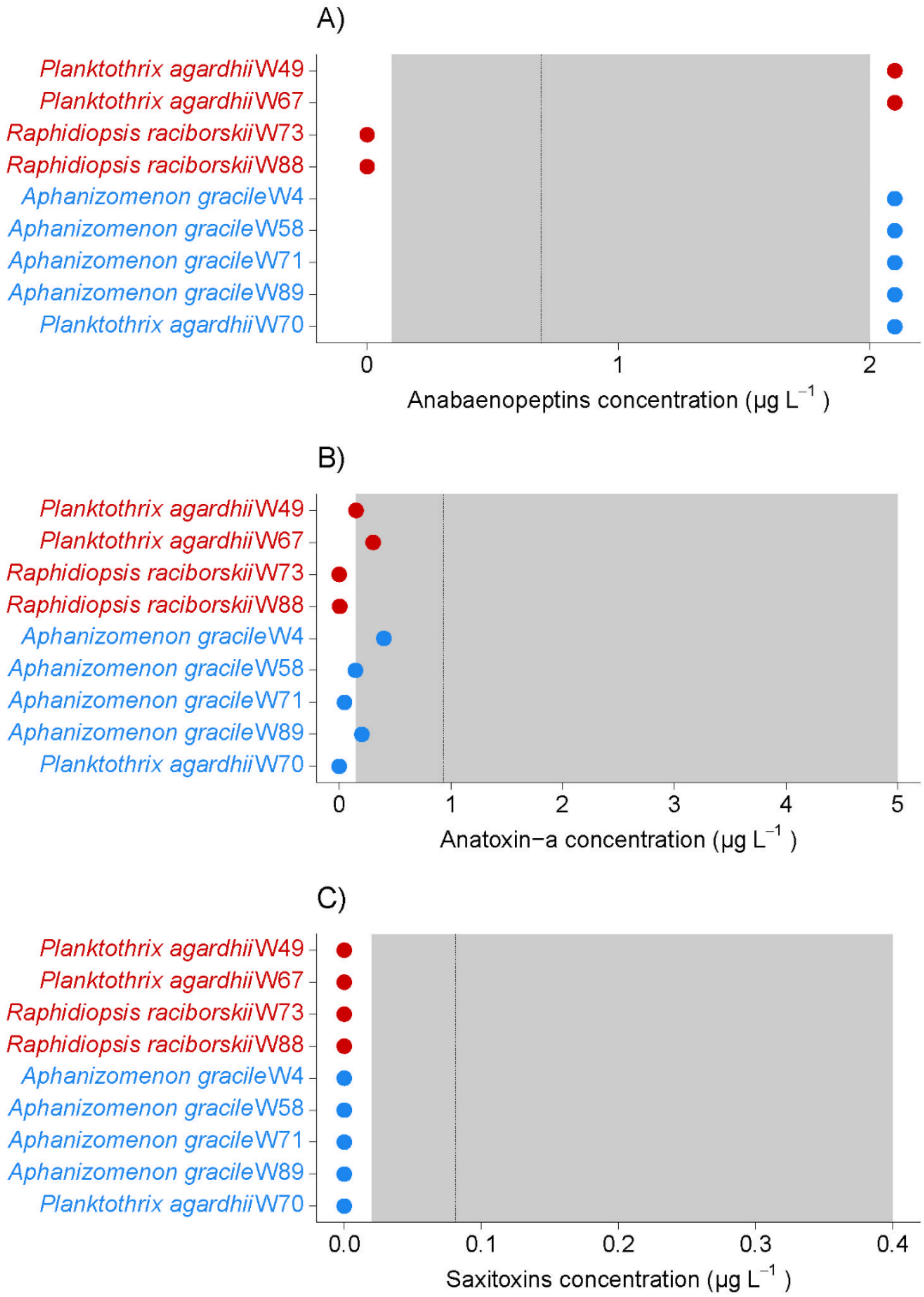

Commentary: Please note that detected concentrations of anatoxin-a close to lower detection limit in some strains by ELISA immunoassay were false positive as no evidence for anatoxin-a presence was found by other methods: HPLC-DAD, LC-MS, and genotoxicity assessment (*ana*F).
