# Supplementary material for: Community Structure and Toxicity Potential of Cyanobacteria during Summer and Winter in a Temperate-Zone Lake Susceptible to Phytoplankton Blooms
Source: Toxins (Basel). 2024 Aug 14;16(8):357. doi: 10.3390/toxins16080357 (PMC11359657; doi:10.3390/toxins16080357)
Supplement: Supplementary file 1 [file toxins-16-00357-s001.zip › S6.pdf]

Community Structure and Toxicity Potential of Cyanobacteria during Summer and Winter in a Temperate-Zone Lake Susceptible to Phytoplankton Blooms

Łukasz Wejnerowski[1*], Tamara Dulić[2], Sultana Akter[3], Arnoldo Font-Nájera[4], Michał Rybak[5], Oskar Kamiński[1], Anna Czerepska[1], Marcin Krzysztof Dziuba[6], Tomasz Jurczak[7], Jussi Meriluoto[2*], Joanna Mankiewicz-Boczek[7], Mikołaj Kokociński[1]

[1] Department of Hydrobiology, Institute of Environmental Biology, Faculty of Biology, Adam Mickiewicz University, Uniwersytetu Poznańskiego 6, 61-614 Poznań, Poland;

[2] Biochemistry and Cell Biology, Faculty of Science and Engineering, Åbo Akademi University, Tykistökatu 6A, 20520 Turku, Finland;

[3] Biotechnology, Department of Life Technologies, Faculty of Technology, University of Turku, 20520 Turku, Finland;

[4] European Regional Centre for Ecohydrology of the Polish Academy of Sciences, Tylna 3, 90-364 Łódź, Poland;

[5] Department of Water Protection, Institute of Environmental Biology; Faculty of Biology; Adam Mickiewicz University; Uniwersytetu Poznańskiego 6, 61-614 Poznań, Poland;

[6] Department of Ecology and Evolutionary Biology, University of Michigan; MI 48109 Ann Arbor, USA;

[7] University of Lodz, Faculty of Biology and Environmental Protection, UNESCO Chair on Ecohydrology and Applied Ecology; Banacha 12/16, 90-237 Łódź, Poland;

Correspondence: wejner@amu.edu.pl (Ł.W.); Jussi.Meriluoto@abo.fi (J.M.)

Supplementary Information S6

The results of HPLC-DAD for extracts from cyanobacterial strains

ATX-a

The spectrum of the compound of similar RT to ATX-a is not visualized if it does not match the spectrum of the ATX-a in the standard.

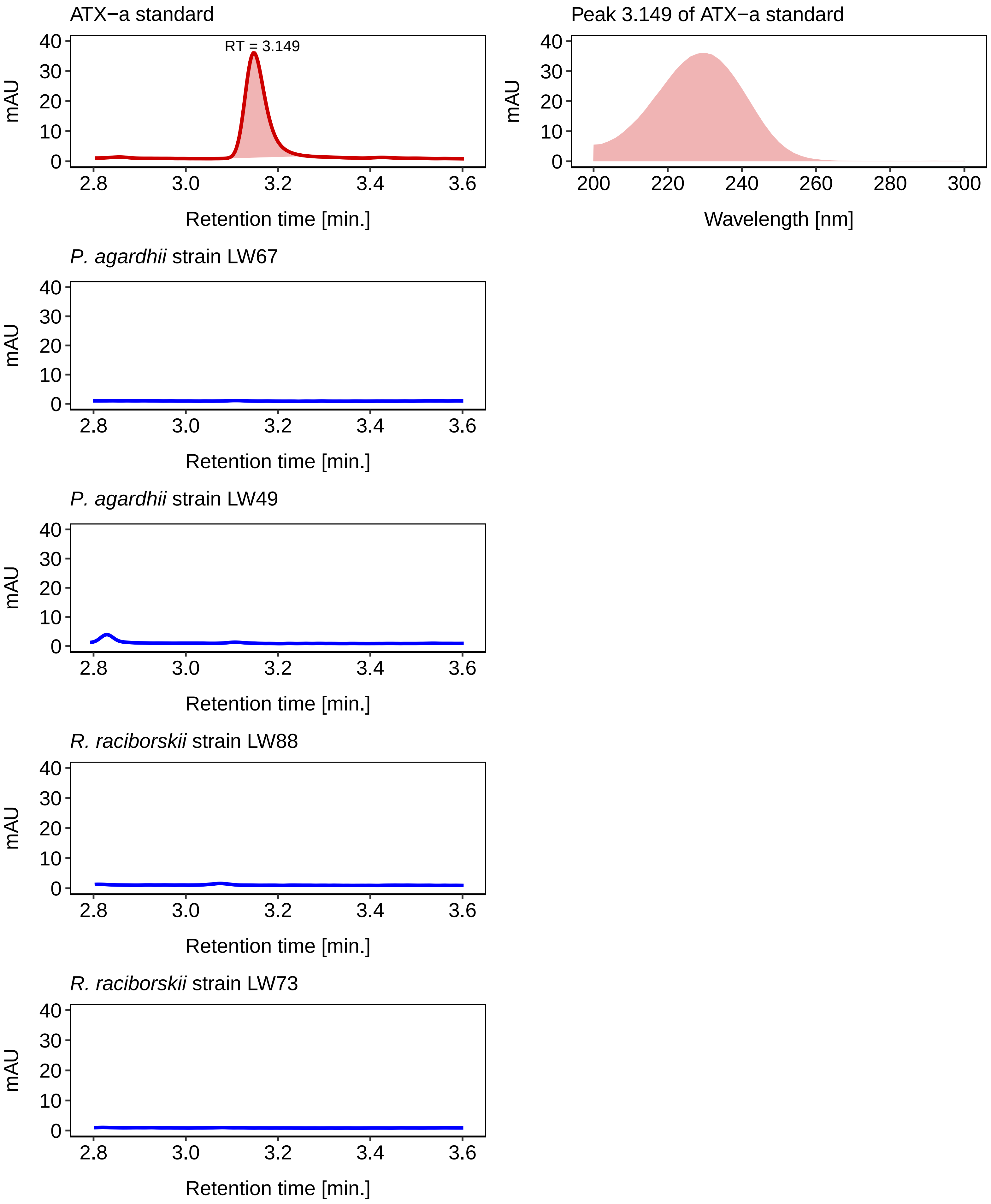
ATX−a standard
RT = 3.149
mAU
Retention time [min.]
Peak 3.149 of ATX−a standard
Wavelength [nm]
P. agardhii strain LW67
P. agardhii strain LW49
R. raciborskii strain LW88
R. raciborskii strain LW73
40
30
20
10
0
2.8
3.0
3.2
3.4
3.6
200
220
240
260
280
300

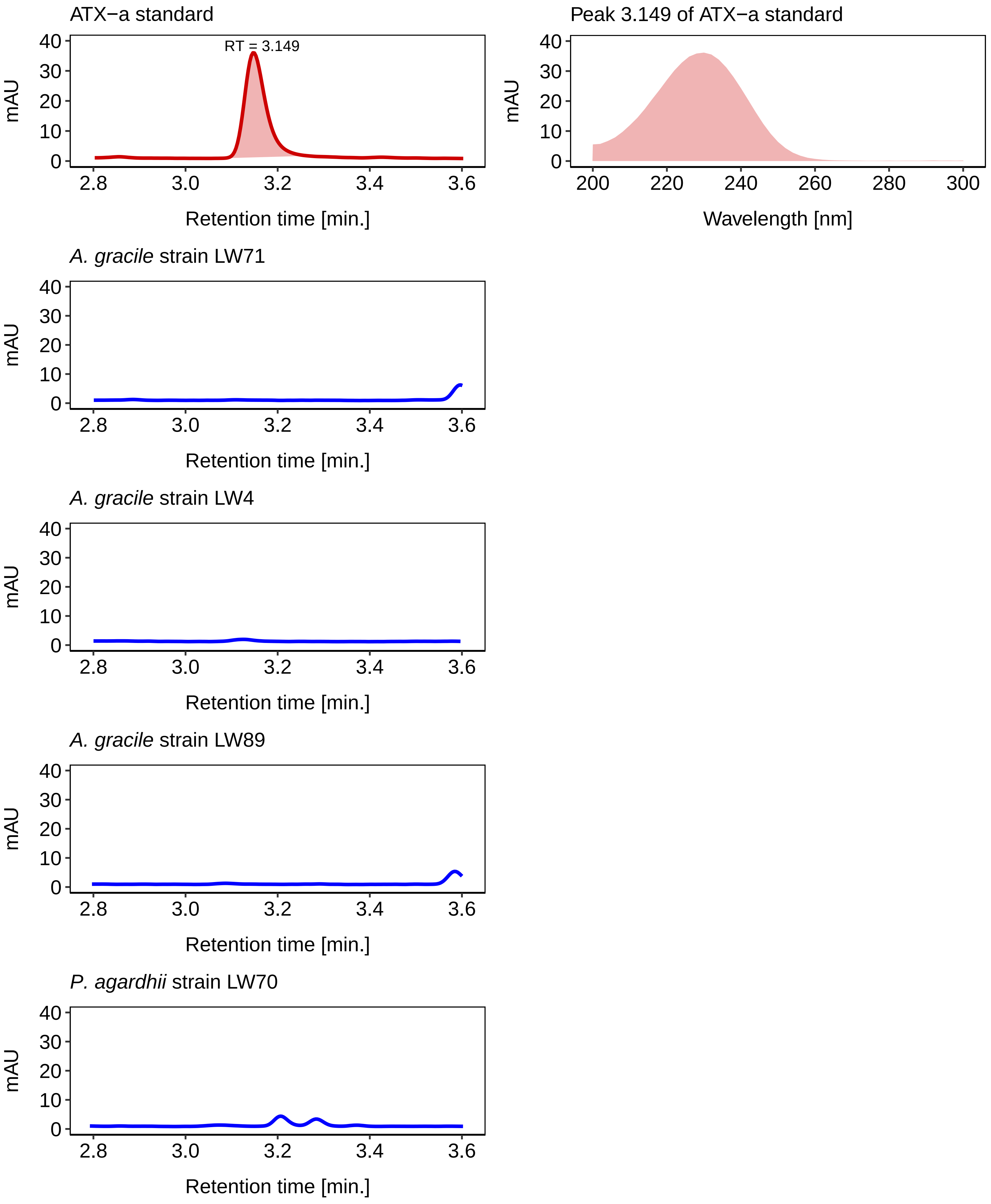

ATX−a standard
RT = 3.149
mAU
Retention time [min.]
Peak 3.149 of ATX−a standard
Wavelength [nm]
A. gracile strain LW71
A. gracile strain LW4
A. gracile strain LW89
P. agardhii strain LW70

CYN

The spectrum of the compound of similar RT to CYN is not visualized if it does not match the spectrum of the CYN in the standard.

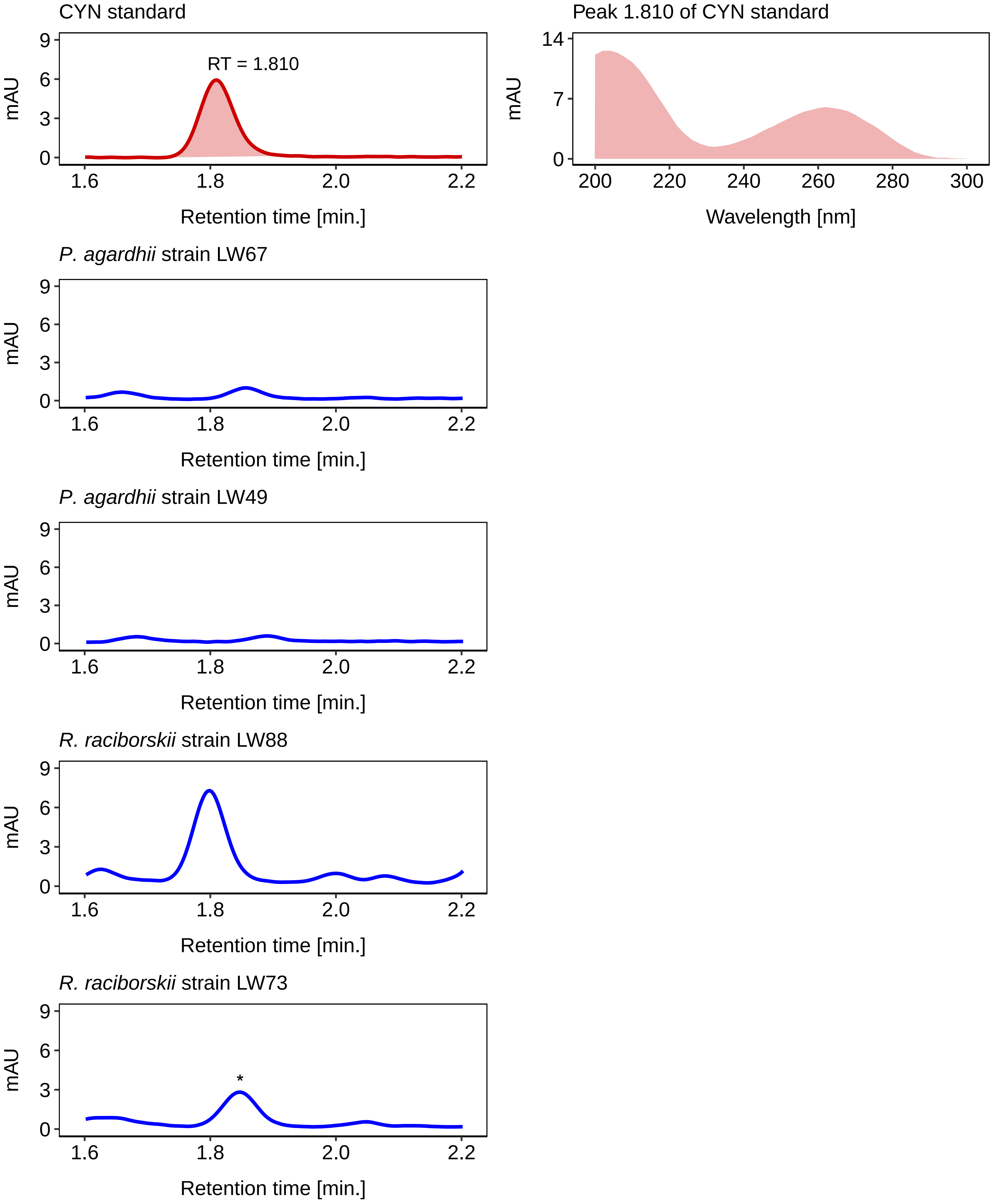
CYN standard
RT = 1.810
mAU
Retention time [min.]
Peak 1.810 of CYN standard
Wavelength [nm]
P. agardhii strain LW67
P. agardhii strain LW49
R. raciborskii strain LW88
R. raciborskii strain LW73
*

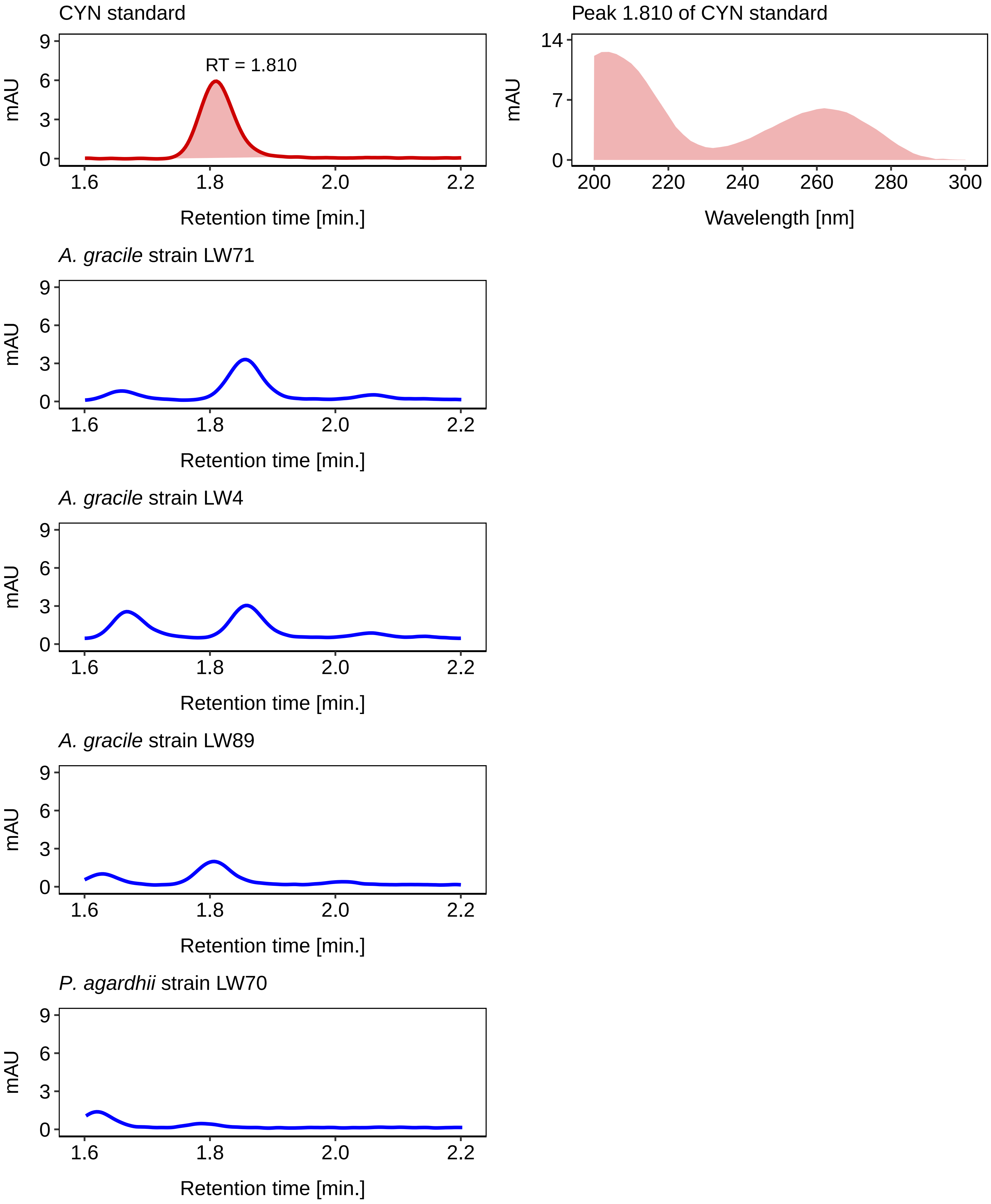
CYN standard
RT = 1.810
mAU
Retention time [min.]
Peak 1.810 of CYN standard
mAU
Wavelength [nm]
A. gracile strain LW71
mAU
Retention time [min.]
A. gracile strain LW4
mAU
Retention time [min.]
A. gracile strain LW89
mAU
Retention time [min.]
P. agardhii strain LW70
mAU
Retention time [min.]

MCs

The spectrum of the compound of similar RT to a given MC variant is not visualized if it does not match the spectrum of the MC variant in the standard. Chromatograms with spectra of the MCs detected in *P. agardhii* LW67 (dmMC-RR, MC-YR, dmMC-LR) and *P. agardhii* LW70 (dmMC-RR, dmMC-LR) are presented in the publication.

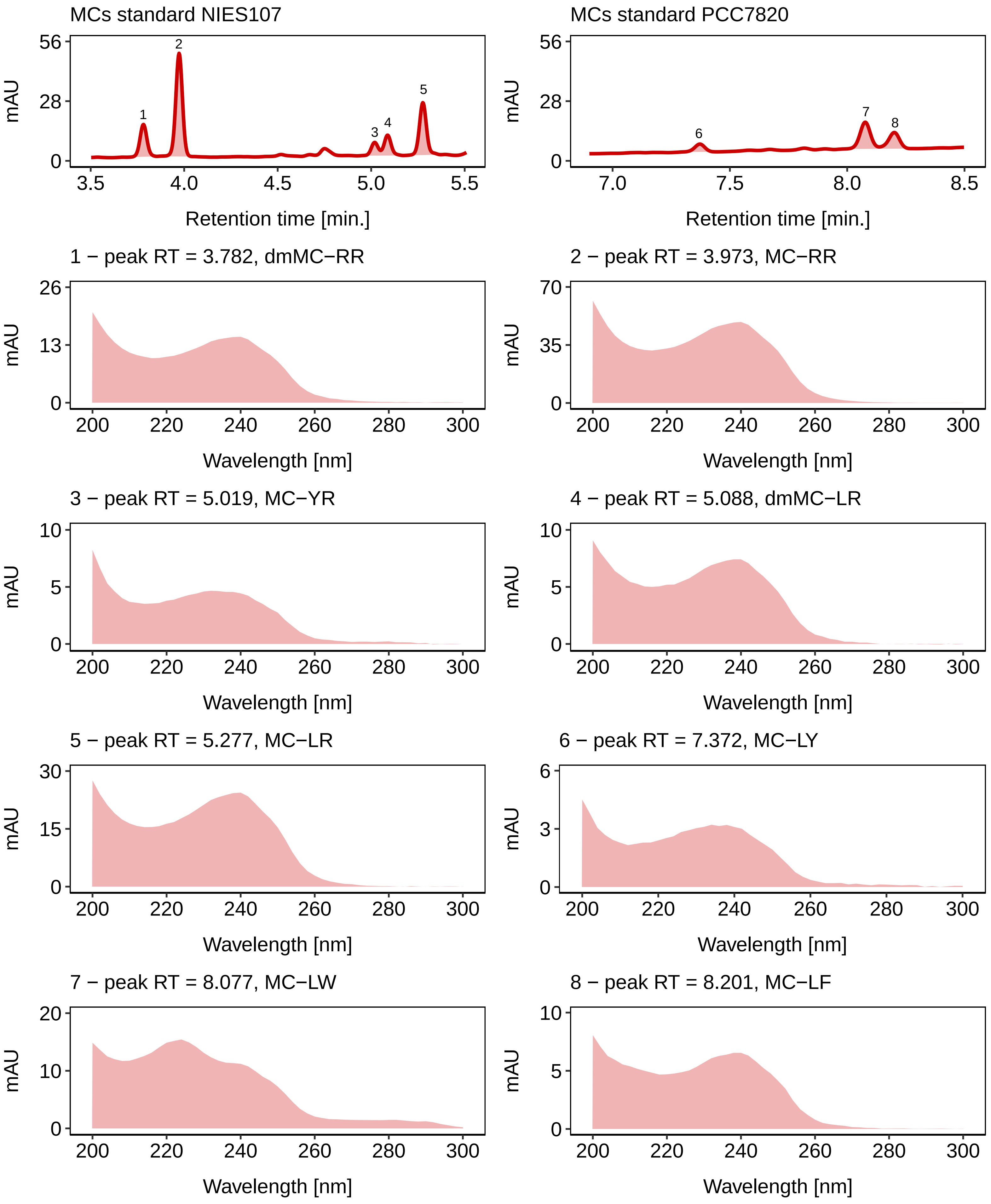

MCs standard NIES107
MCs standard PCC7820
mAU
Retention time [min.]
1 − peak RT = 3.782, dmMC−RR
2 − peak RT = 3.973, MC−RR
3 − peak RT = 5.019, MC−YR
4 − peak RT = 5.088, dmMC−LR
5 − peak RT = 5.277, MC−LR
6 − peak RT = 7.372, MC−LY
7 − peak RT = 8.077, MC−LW
8 − peak RT = 8.201, MC−LF
Wavelength [nm]

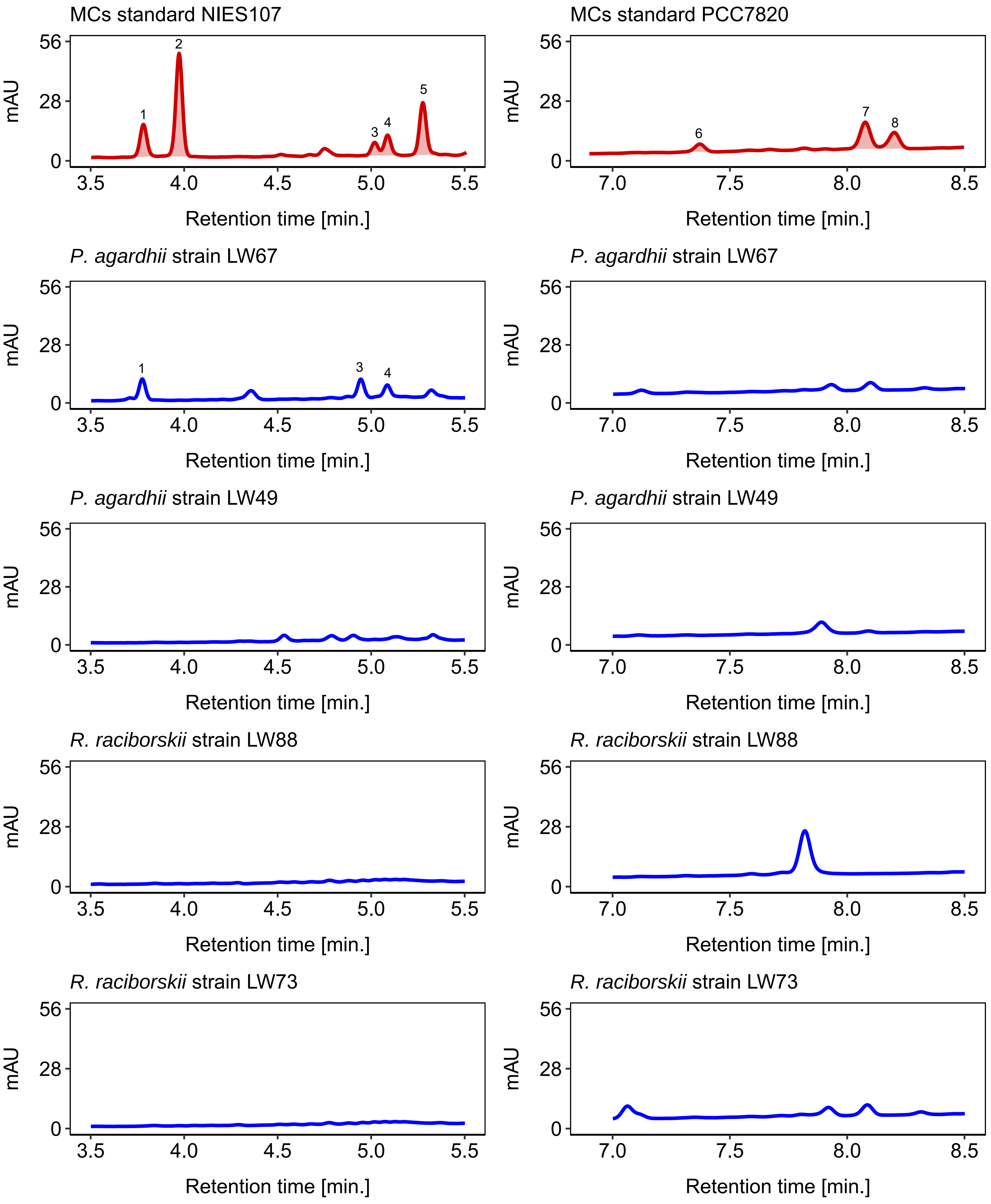
MCs standard NIES107
MCs standard PCC7820
1
2
3
4
5
6
7
8
P. agardhii strain LW67
P. agardhii strain LW67
1
3
4
P. agardhii strain LW49
P. agardhii strain LW49
R. raciborskii strain LW88
R. raciborskii strain LW88
R. raciborskii strain LW73
R. raciborskii strain LW73
mAU
0
28
56
Retention time [min.]
3.5
4.0
4.5
5.0
5.5
7.0
7.5
8.0
8.5

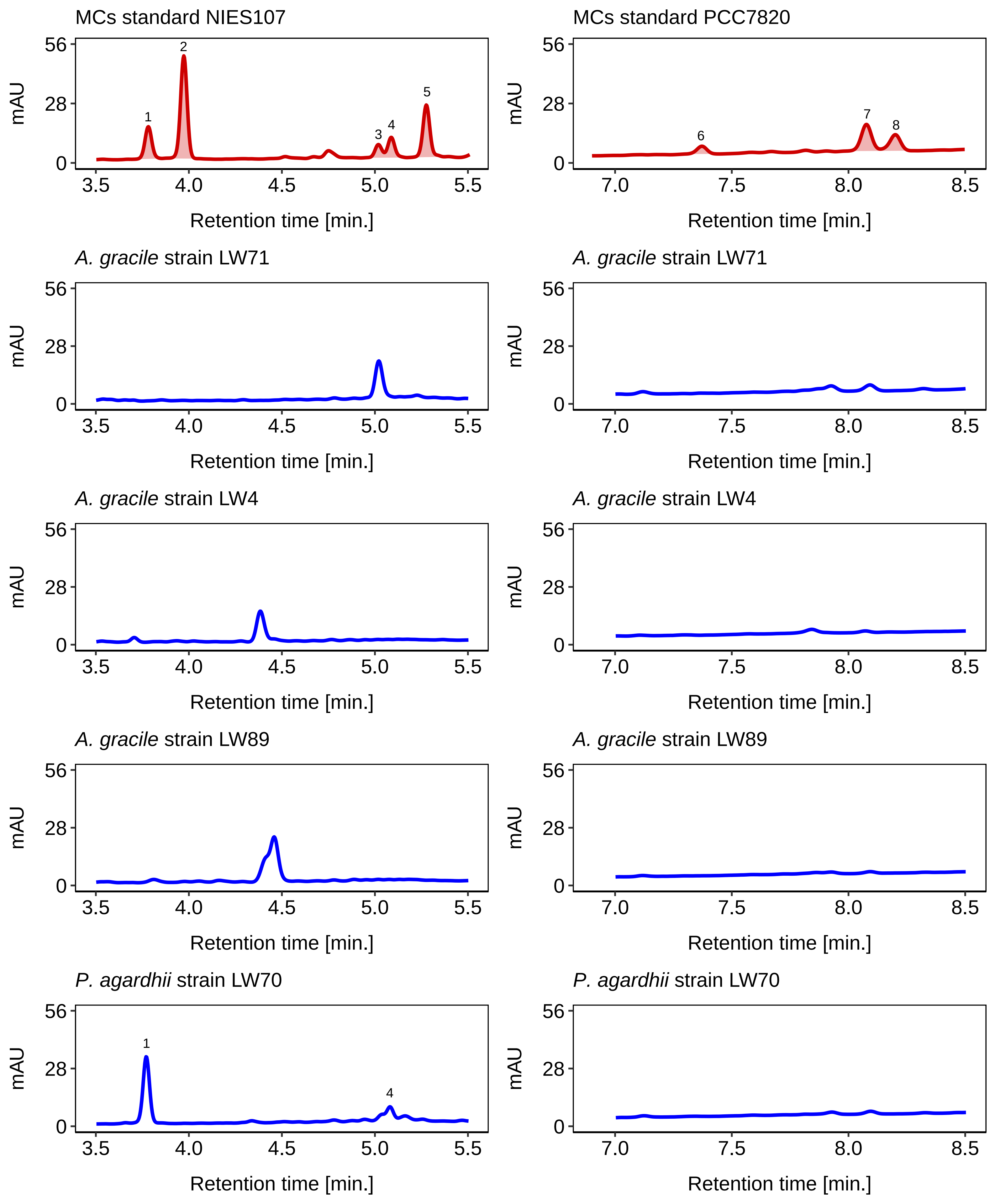
MCs standard NIES107
MCs standard PCC7820
A. gracile strain LW71
A. gracile strain LW71
A. gracile strain LW4
A. gracile strain LW4
A. gracile strain LW89
A. gracile strain LW89
P. agardhii strain LW70
P. agardhii strain LW70
mAU
0
28
56
1
2
3
4
5
6
7
8
3.5
4.0
4.5
5.0
5.5
7.0
7.5
8.0
8.5
Retention time [min.]

NOD

The spectrum of the compound of similar RT to NOD is not visualized if it does not match the spectrum of the NOD in the standard.

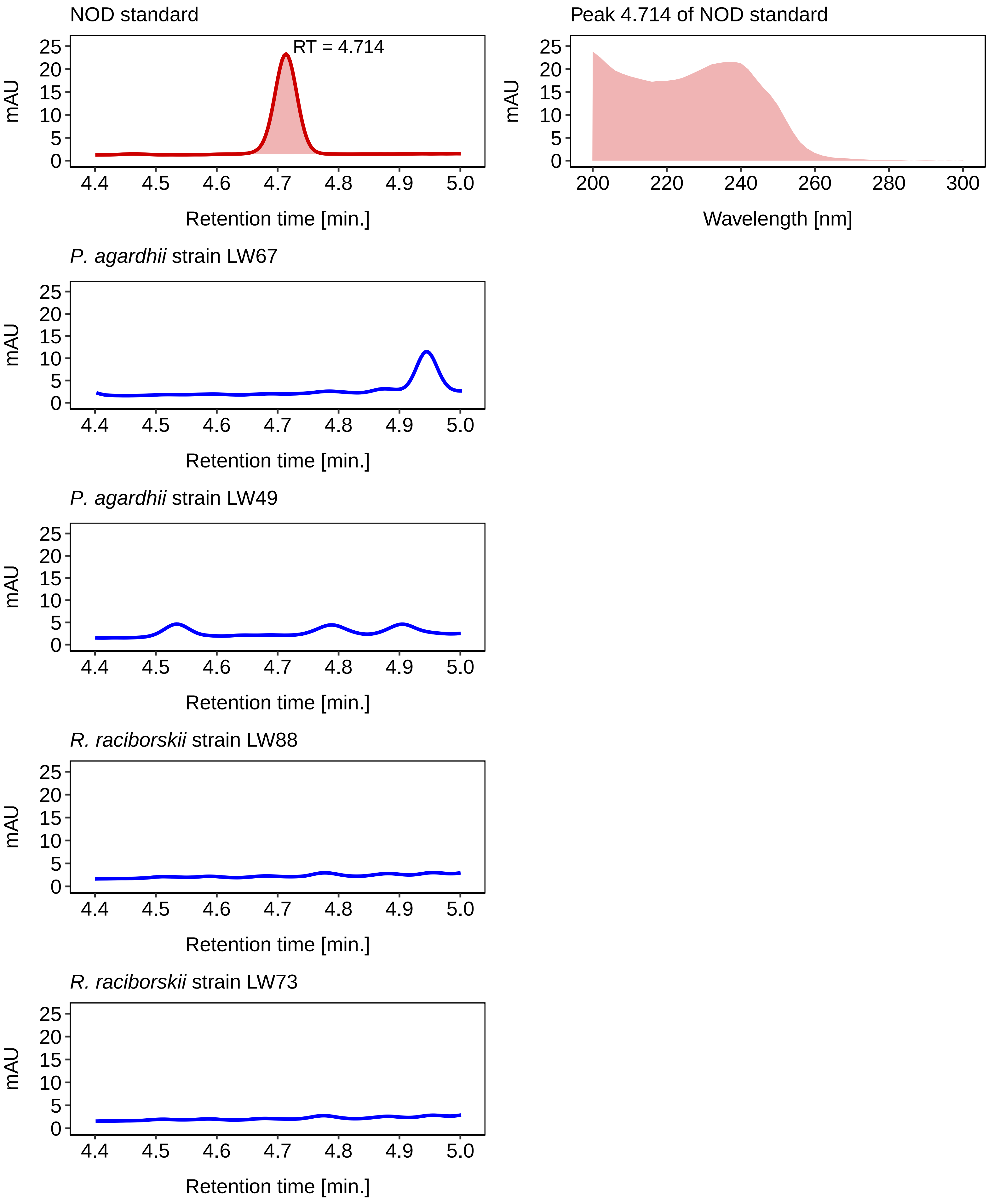
NOD standard
RT = 4.714
mAU
Retention time [min.]
Peak 4.714 of NOD standard
mAU
Wavelength [nm]
P. agardhii strain LW67
mAU
Retention time [min.]
P. agardhii strain LW49
mAU
Retention time [min.]
R. raciborskii strain LW88
mAU
Retention time [min.]
R. raciborskii strain LW73
mAU
Retention time [min.]

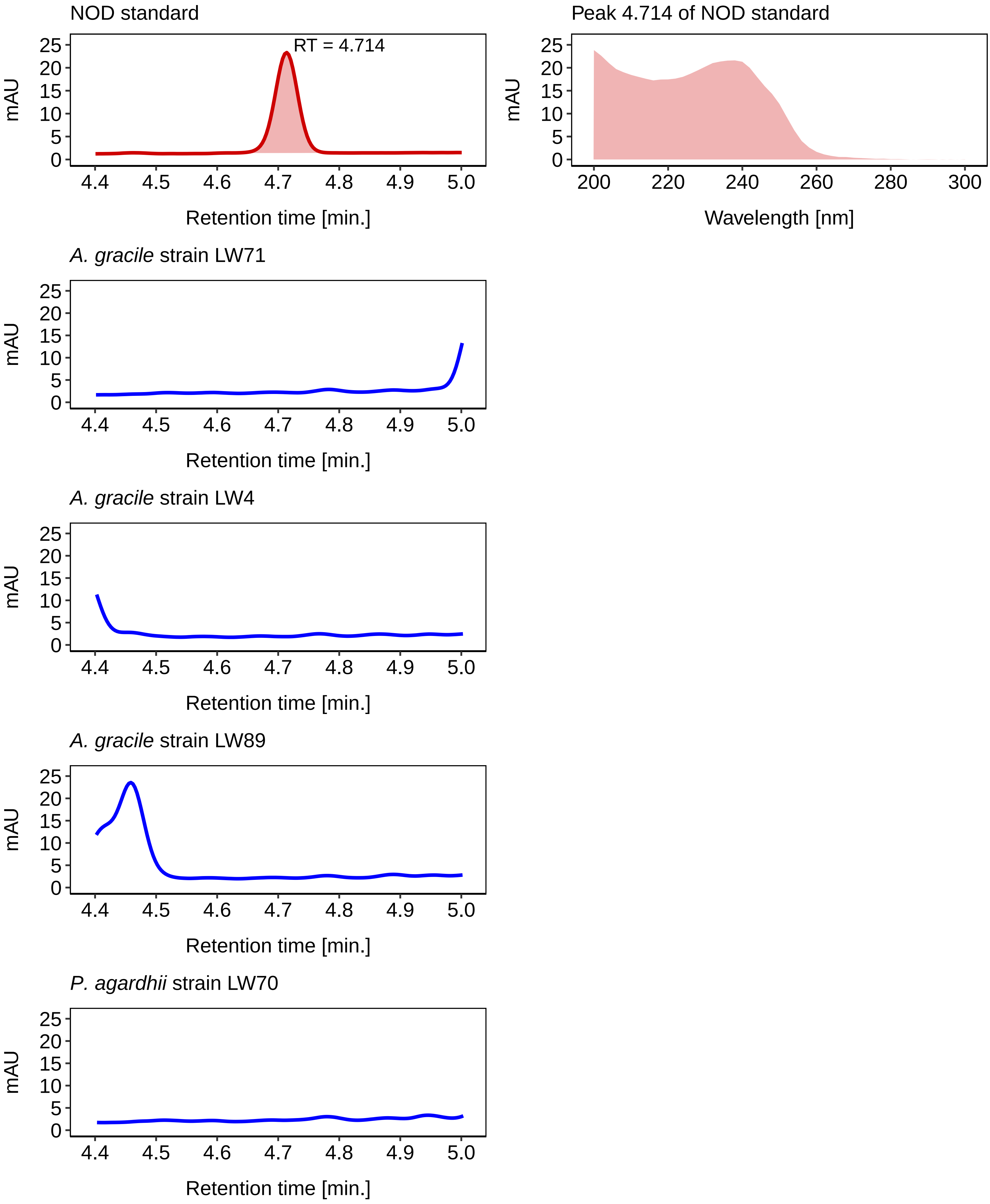

NOD standard
RT = 4.714
mAU
Retention time [min.]
Peak 4.714 of NOD standard
mAU
Wavelength [nm]
A. gracile strain LW71
mAU
Retention time [min.]
A. gracile strain LW4
mAU
Retention time [min.]
A. gracile strain LW89
mAU
Retention time [min.]
P. agardhii strain LW70
mAU
Retention time [min.]
