# Supplementary material for: Community Structure and Toxicity Potential of Cyanobacteria during Summer and Winter in a Temperate-Zone Lake Susceptible to Phytoplankton Blooms
Source: Toxins (Basel). 2024 Aug 14;16(8):357. doi: 10.3390/toxins16080357 (PMC11359657; doi:10.3390/toxins16080357)

Community Structure and Toxicity Potential of Cyanobacteria during Summer and Winter in a Temperate-Zone Lake Susceptible to Phytoplankton Blooms

Łukasz Wejnerowski[1*], Tamara Dulić[2], Sultana Akter[3], Arnoldo Font-Nájera[4], Michał Rybak[5], Oskar Kamiński[1], Anna Czerepska[1], Marcin Krzysztof Dziuba[6], Tomasz Jurczak[7], Jussi Meriluoto[2*], Joanna Mankiewicz-Boczek[7], Mikołaj Kokociński[1]

[1] Department of Hydrobiology, Institute of Environmental Biology, Faculty of Biology, Adam Mickiewicz University, Uniwersytetu Poznańskiego 6, 61-614 Poznań, Poland;

[2] Biochemistry and Cell Biology, Faculty of Science and Engineering, Åbo Akademi University, Tykistökatu 6A, 20520 Turku, Finland;

[3] Biotechnology, Department of Life Technologies, Faculty of Technology, University of Turku, 20520 Turku, Finland;

[4] European Regional Centre for Ecohydrology of the Polish Academy of Sciences, Tylna 3, 90-364 Łódź, Poland;

[5] Department of Water Protection, Institute of Environmental Biology; Faculty of Biology; Adam Mickiewicz University; Uniwersytetu Poznańskiego 6, 61-614 Poznań, Poland;

[6] Department of Ecology and Evolutionary Biology, University of Michigan; MI 48109 Ann Arbor, USA;

[7] University of Lodz, Faculty of Biology and Environmental Protection, UNESCO Chair on Ecohydrology and Applied Ecology; Banacha 12/16, 90-237 Łódź, Poland;

Correspondence: wejner@amu.edu.pl (Ł.W.); Jussi.Meriluoto@abo.fi (J.M.)

Supplementary Information S7

The results of LC-MS for extracts from cyanobacterial strains

ATX-a

Extracted ion chromatograms are presented for all samples. MS-MS spectrum is shown for examined strains of cyanobacteria in the case when it matched the MS-MS spectrum of the ATX-a in the standard.

LC-MS analysis | extracted ion chromatogram (*m/z* 166.3) of ATX-a standard and MS-MS spectrum of the ATX-a in the standard

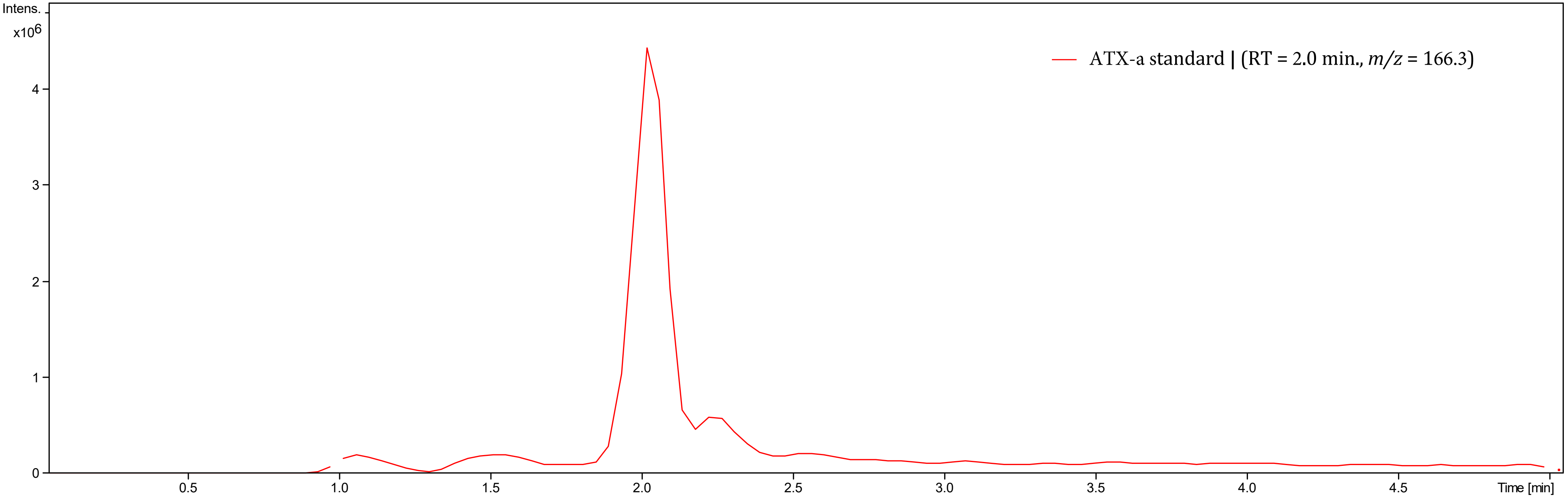

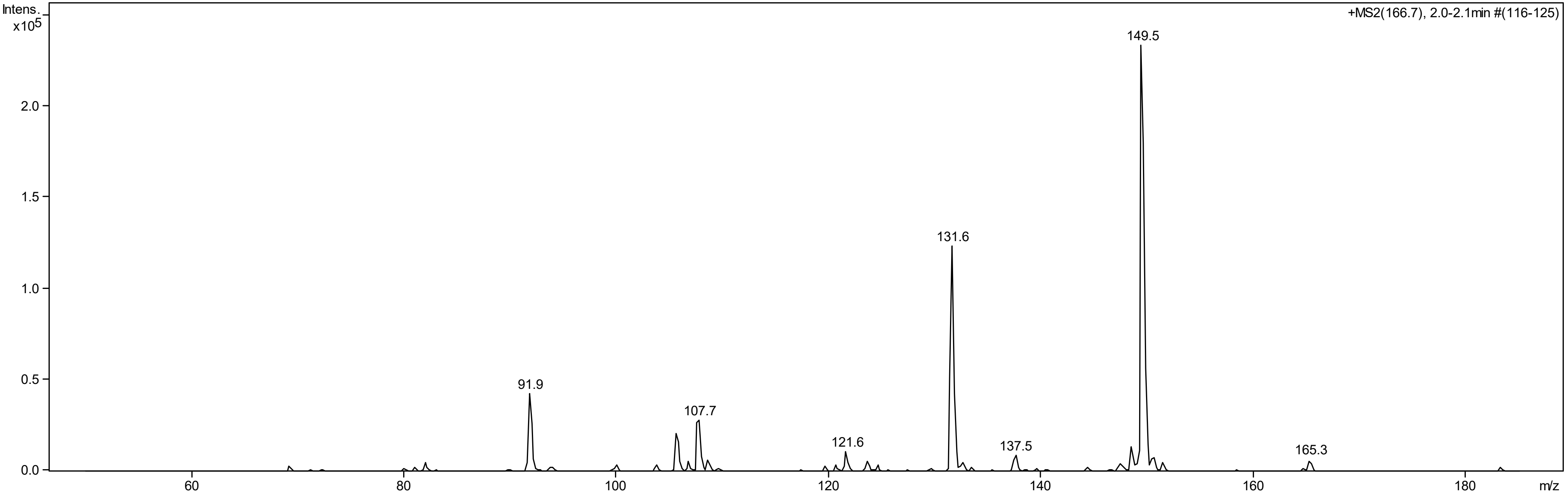

LC-MS analysis | extracted ion chromatogram (*m/z* 166.3) of ATX-a standard and *P. agardhii* W67

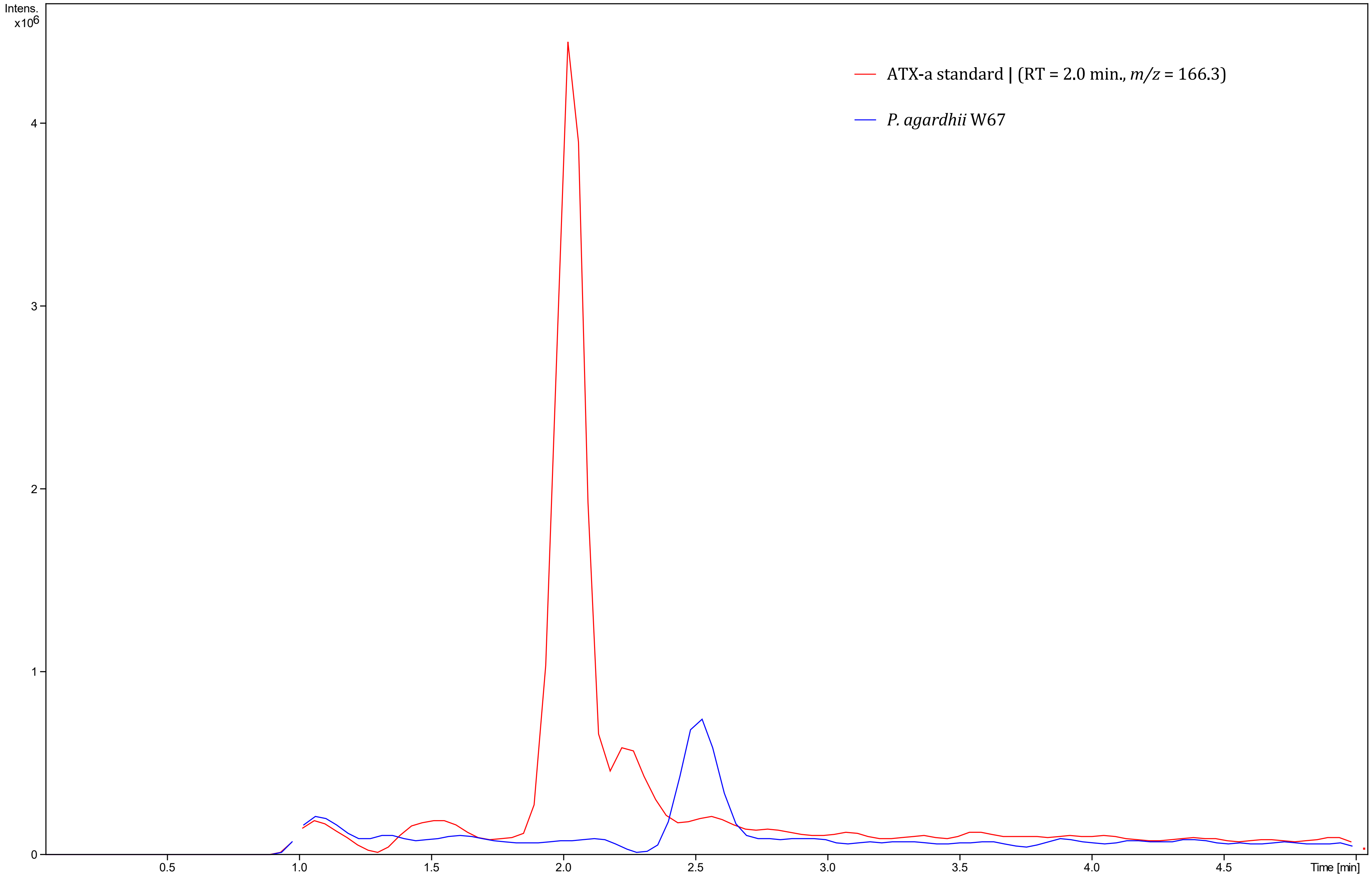

LC-MS analysis | extracted ion chromatogram (*m/z* 166.3) of ATX-a standard and *A. gracile* W4

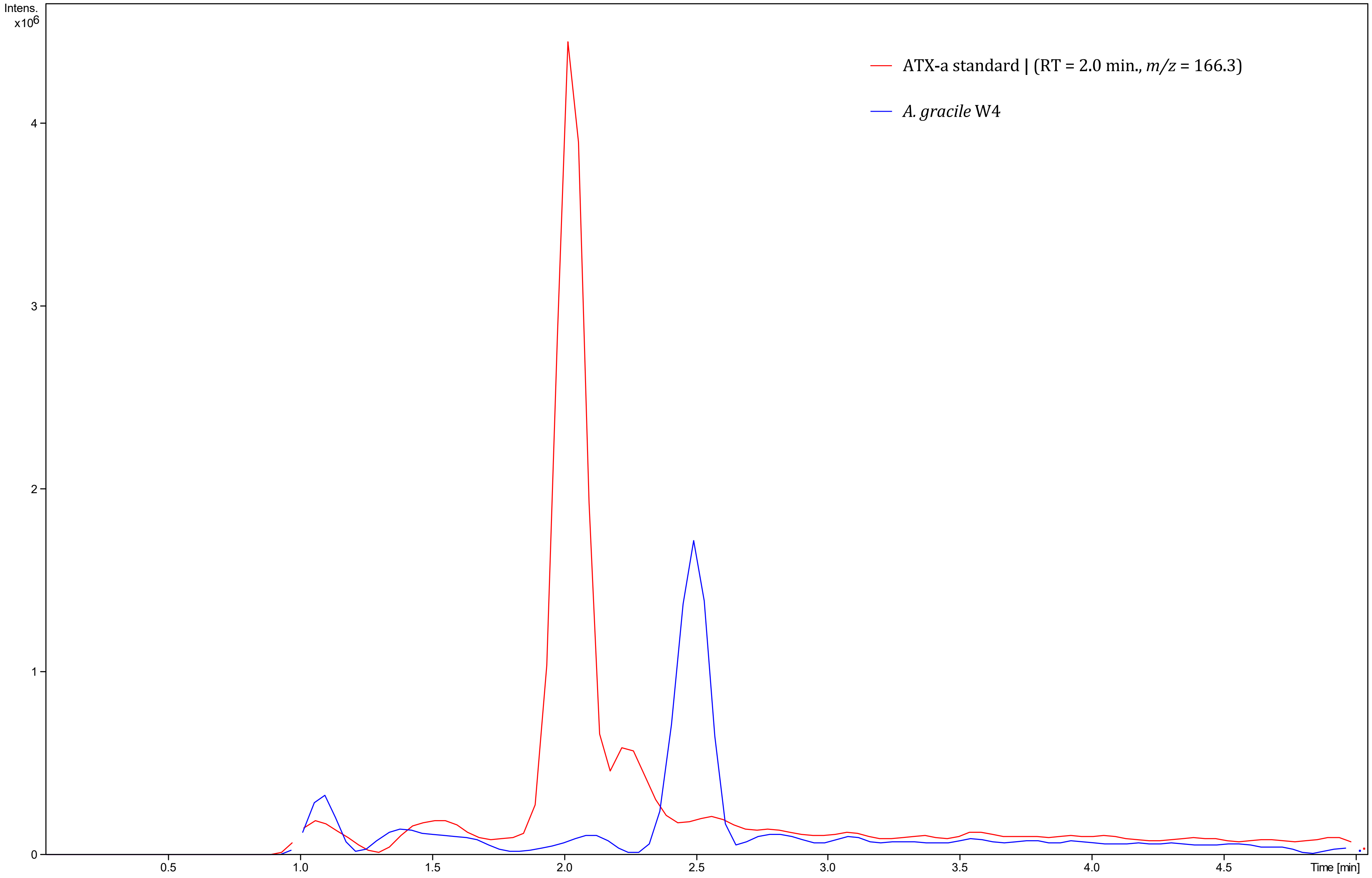

LC-MS analysis | extracted ion chromatogram (*m/z* 166.3) of ATX-a standard and *A. gracile* W89

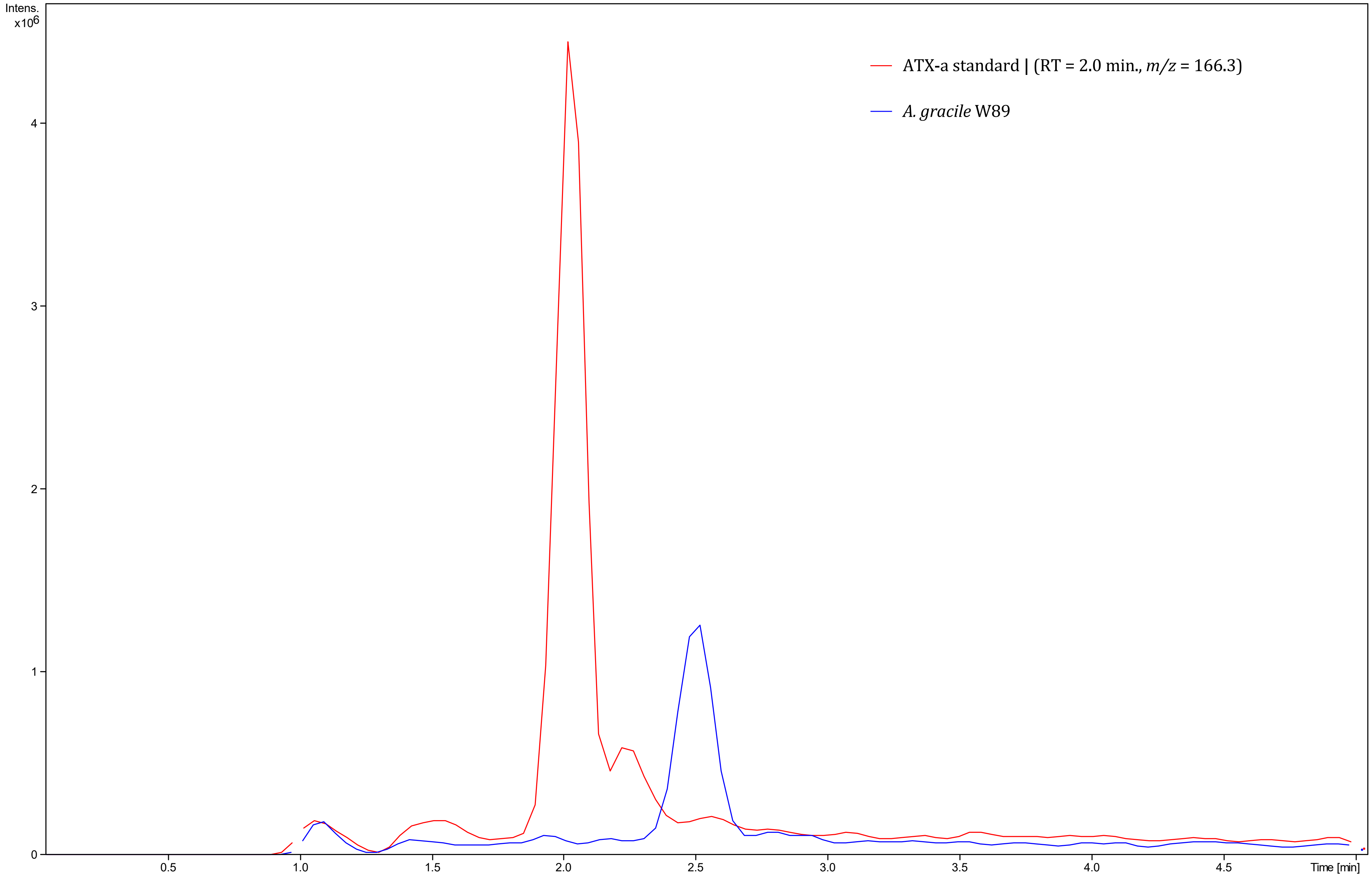

#9 .

Extracted ion chromatograms are presented for all samples. MS-MS spectrum is shown for examined strains of cyanobacteria in case when it matched the MS-MS spectrum of the CYN in the standard.

LC-MS analysis | extracted ion chromatogram (*m/z* 416.4) of CYN standard and MS-MS spectrum of the CYN in the standard

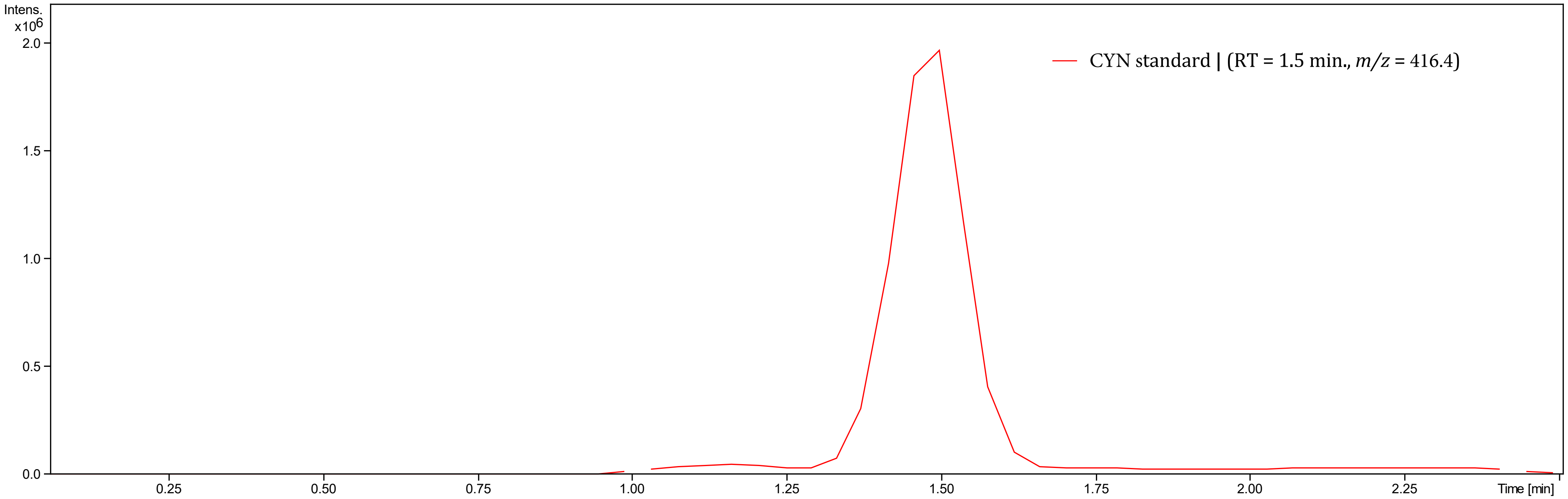

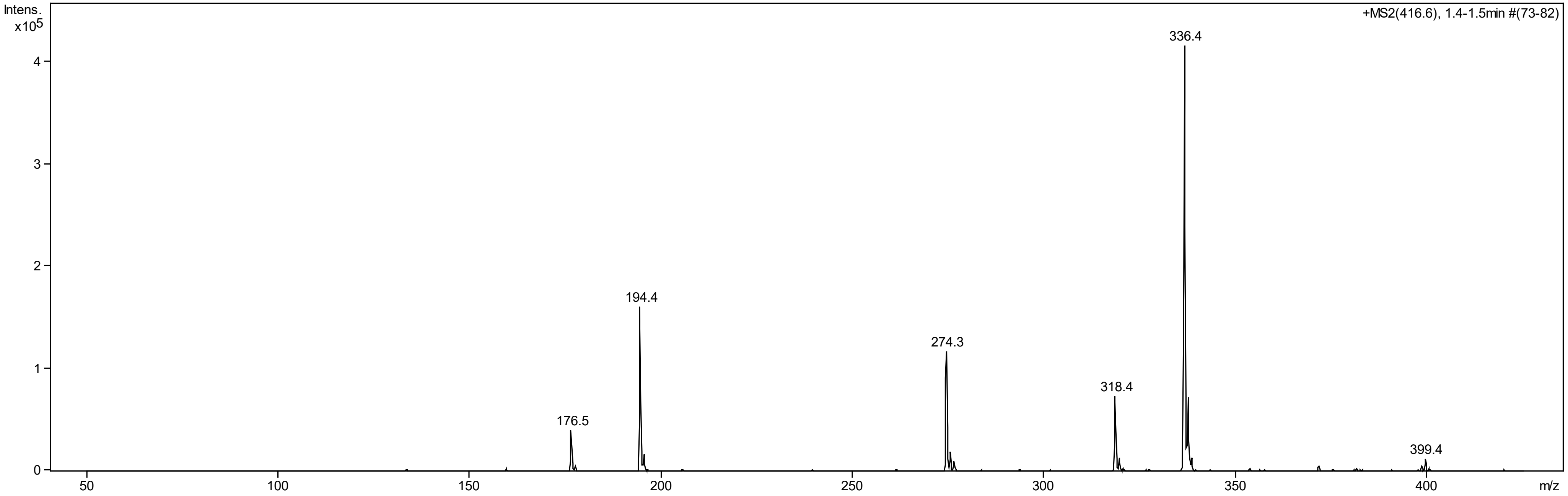

LC-MS analysis | extracted ion chromatogram (*m/z* 416.4) of CYN standard and *P. agardhii* W67

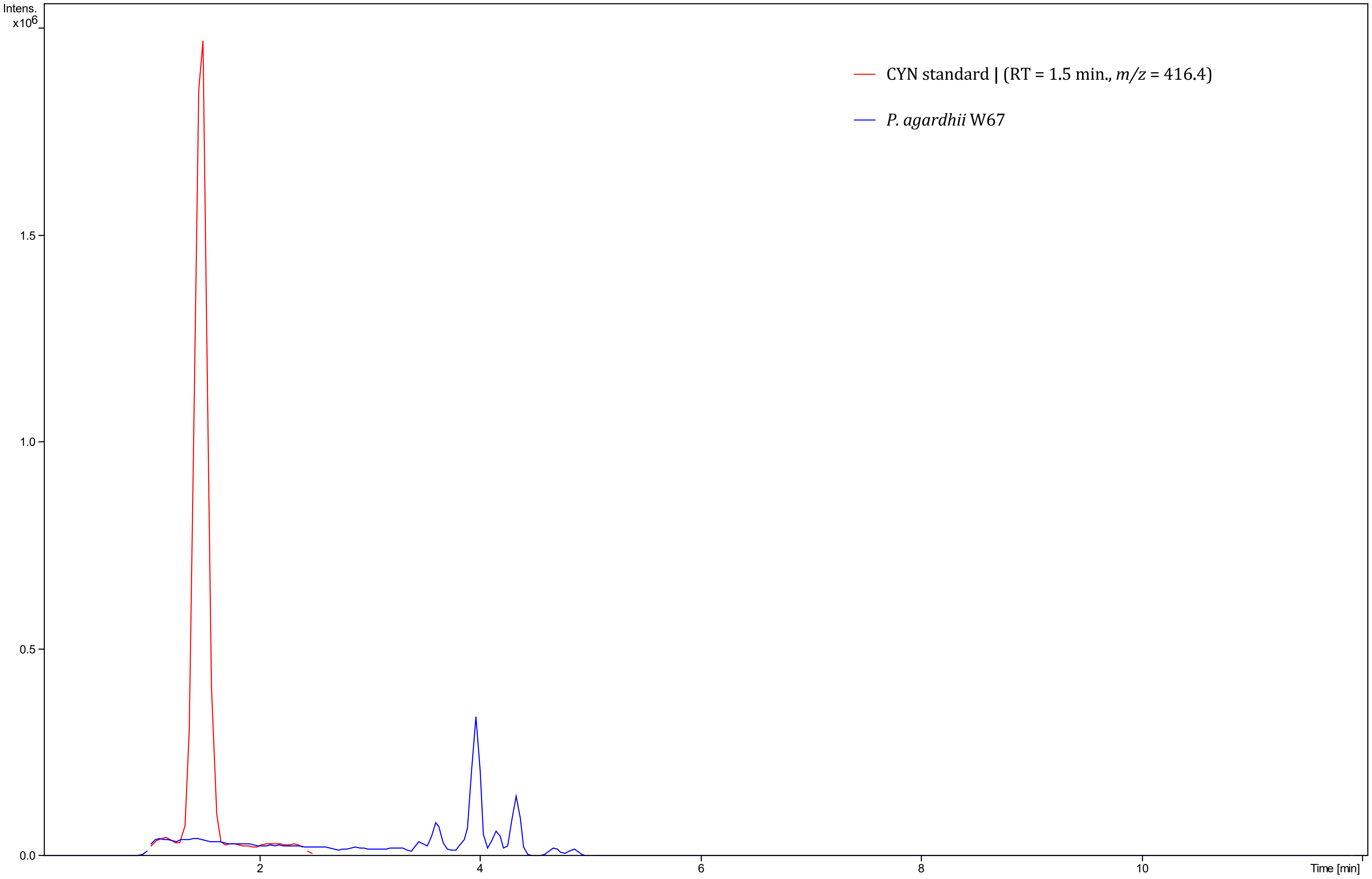

LC-MS analysis | extracted ion chromatogram (*m/z* 416.4) of CYN standard and *P. agardhii* W49

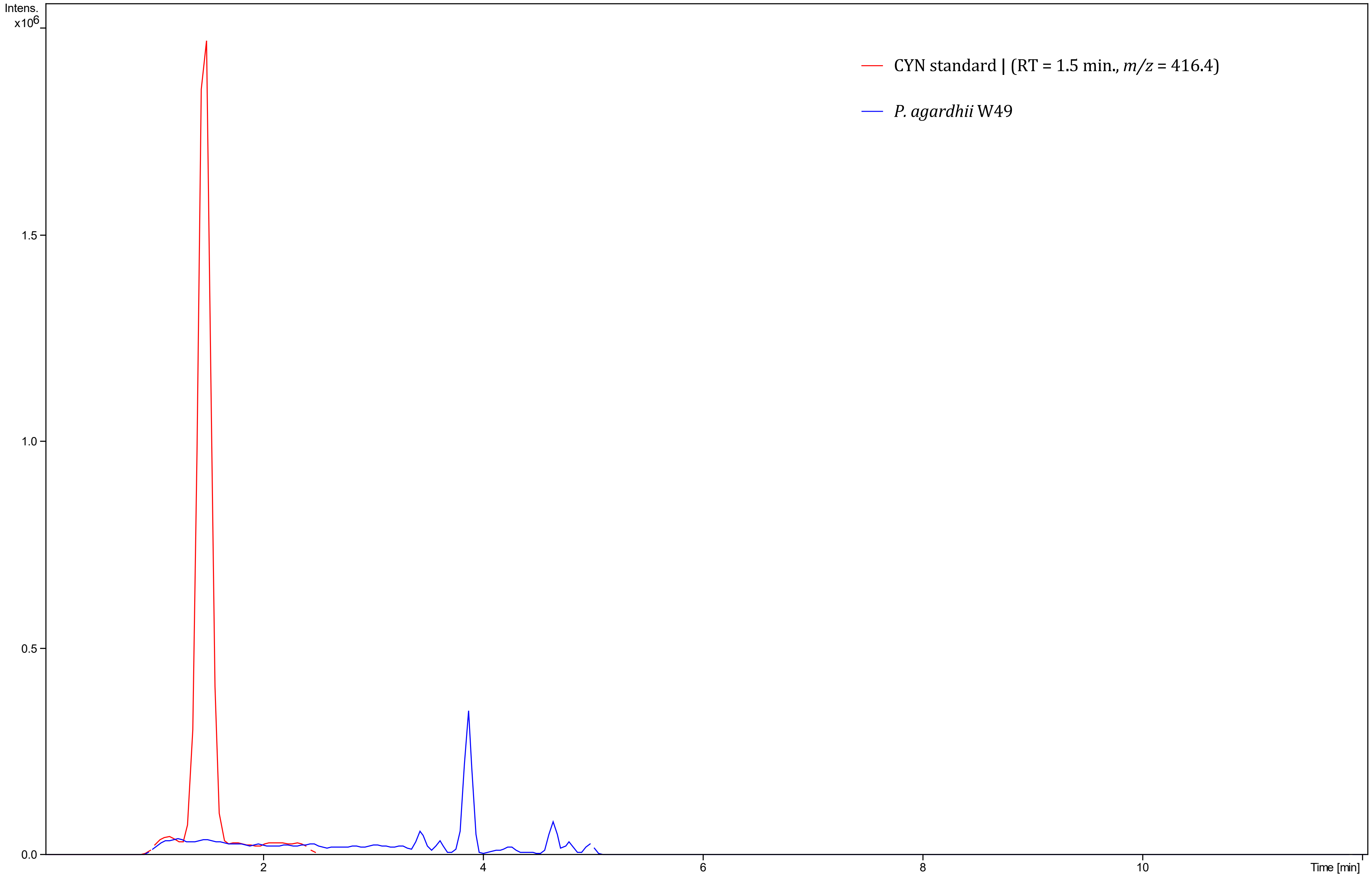

LC-MS analysis | extracted ion chromatogram (m/z 416.4) of CYN standard and *R. raciborskii* W88

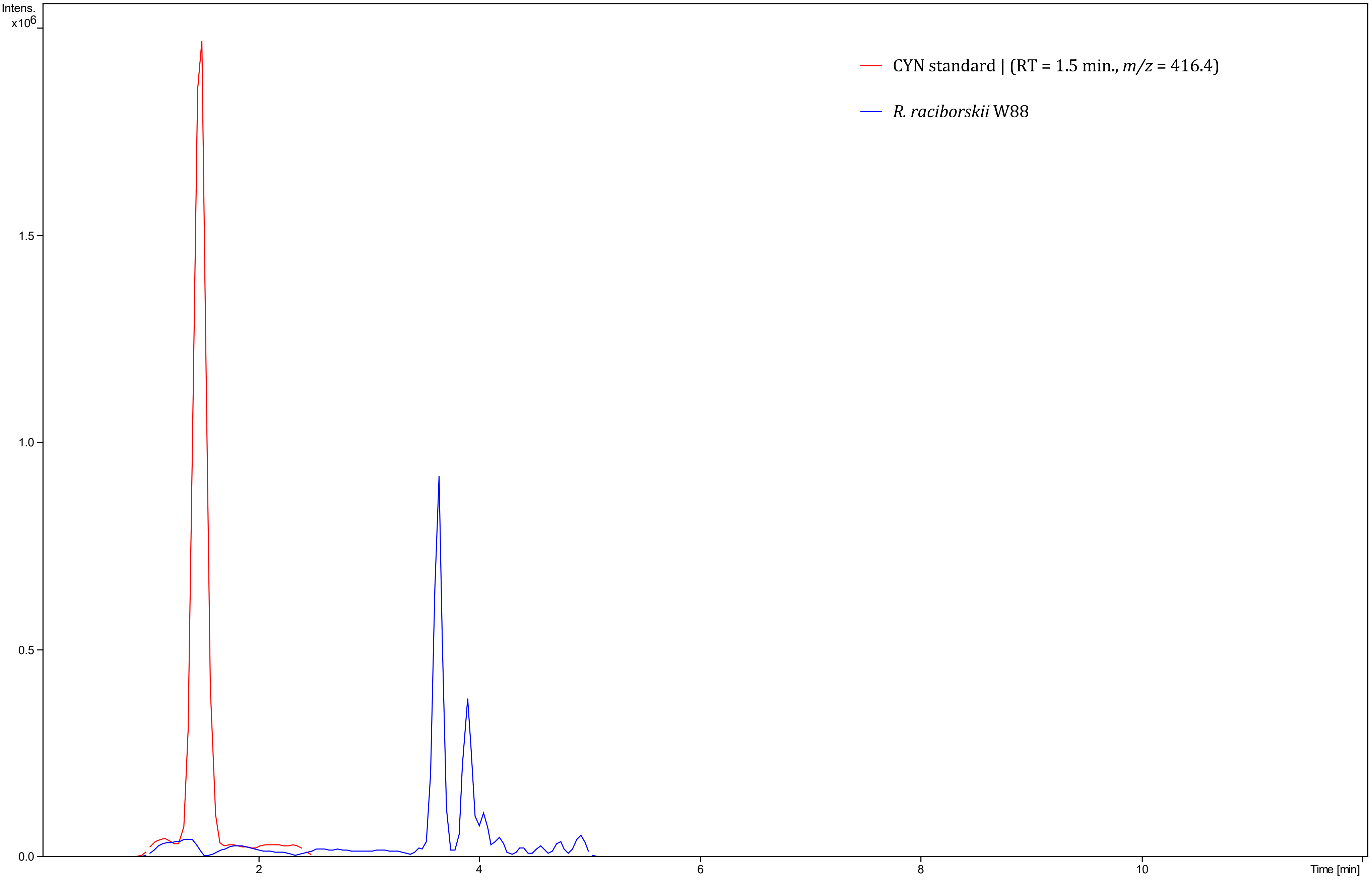

LC-MS analysis | extracted ion chromatogram (*m/z* 416.4) of CYN standard and *R. raciborskii* W73

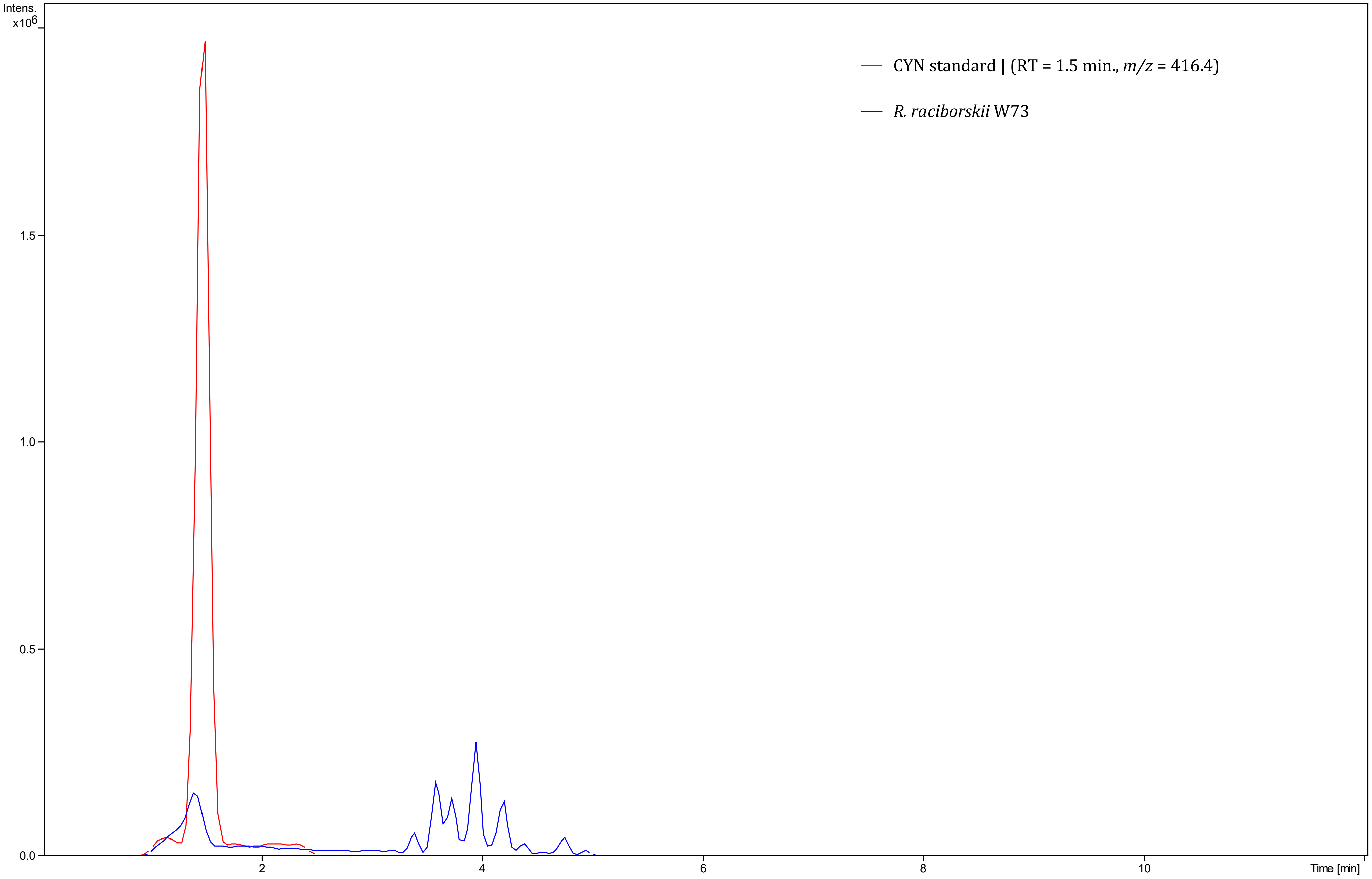

LC-MS analysis | extracted ion chromatogram (*m/z* 416.4) of CYN standard and *A. gracile* W71

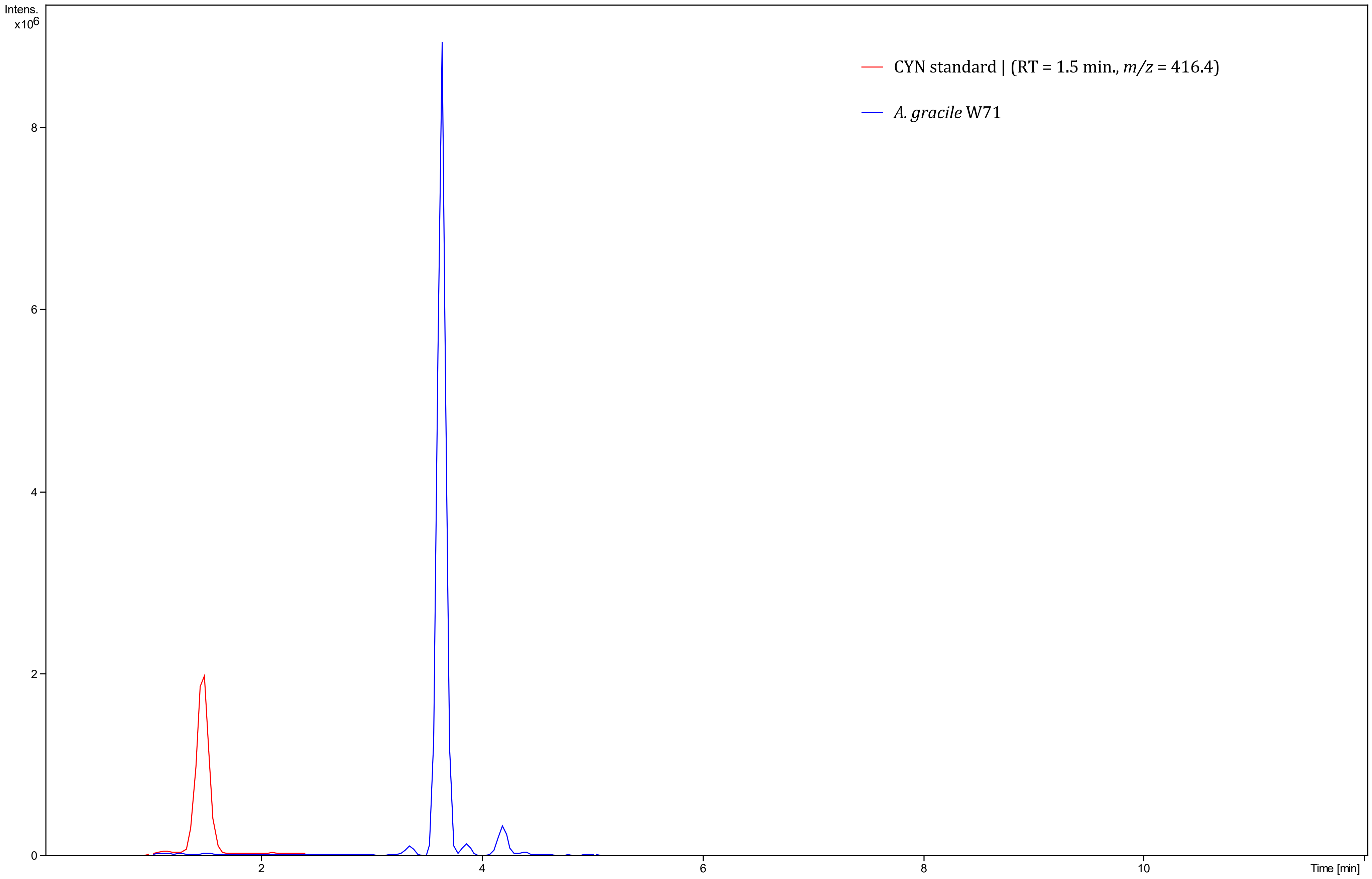

LC-MS analysis | extracted ion chromatogram (*m/z* 416.4) of CYN standard and *A. gracile* W4

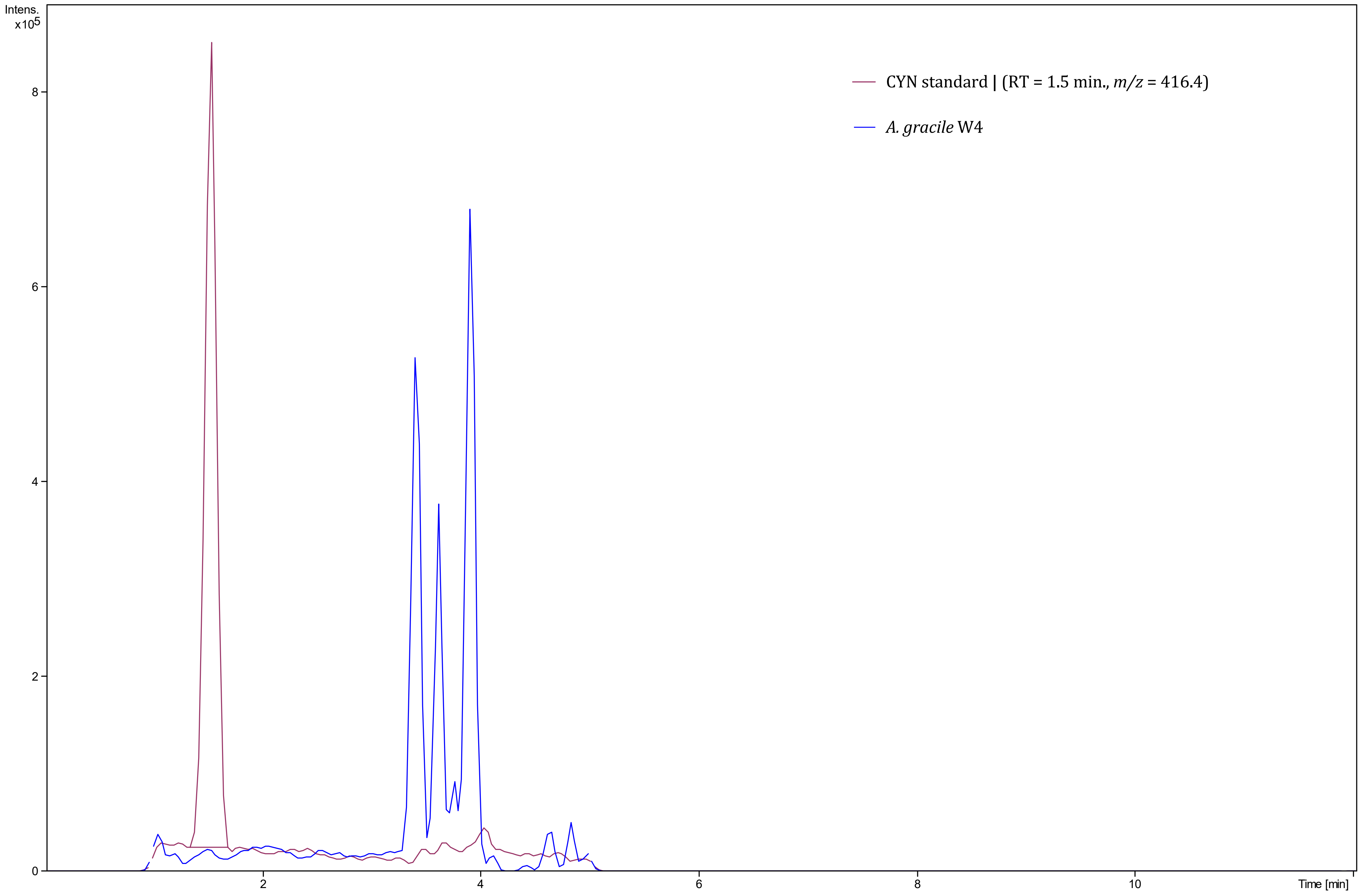

LC-MS analysis | extracted ion chromatogram (*m/z* 416.4) of CYN standard and *A. gracile* W89

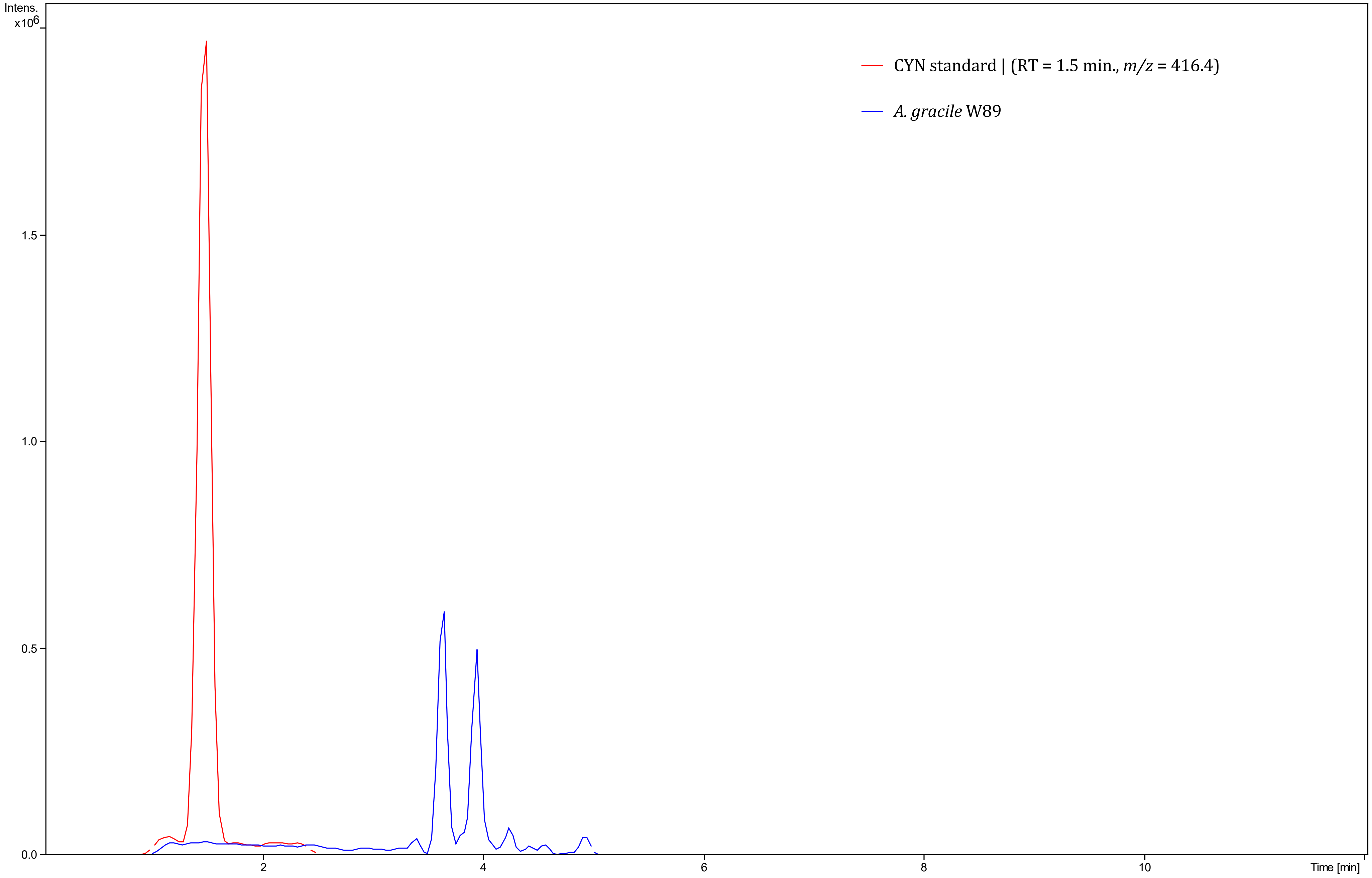

LC-MS analysis | extracted ion chromatogram (*m/z* 416.4) of CYN standard and *A. gracile* W70

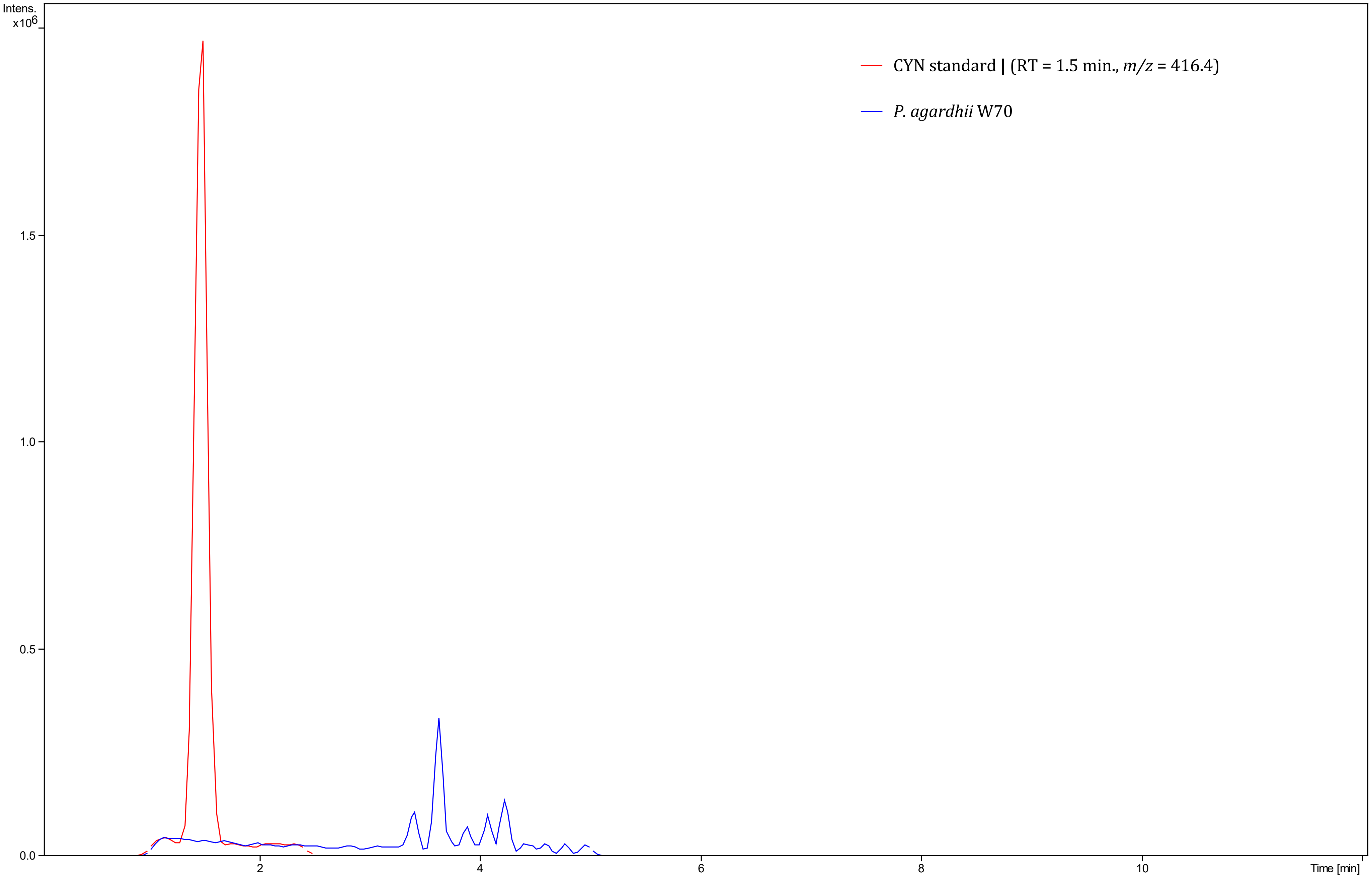

MCs

Extracted ion chromatograms are presented for all samples. Mass spectrum scan is shown for examined strains of cyanobacteria in the case when it matched the mass spectrum scan of a given MC variant in the standard.

LC-MS analysis | extracted ion chromatogram (*m/z* 513.0) of NIES107 standard and mass spectrum scan of the dmMC-RR in the standard

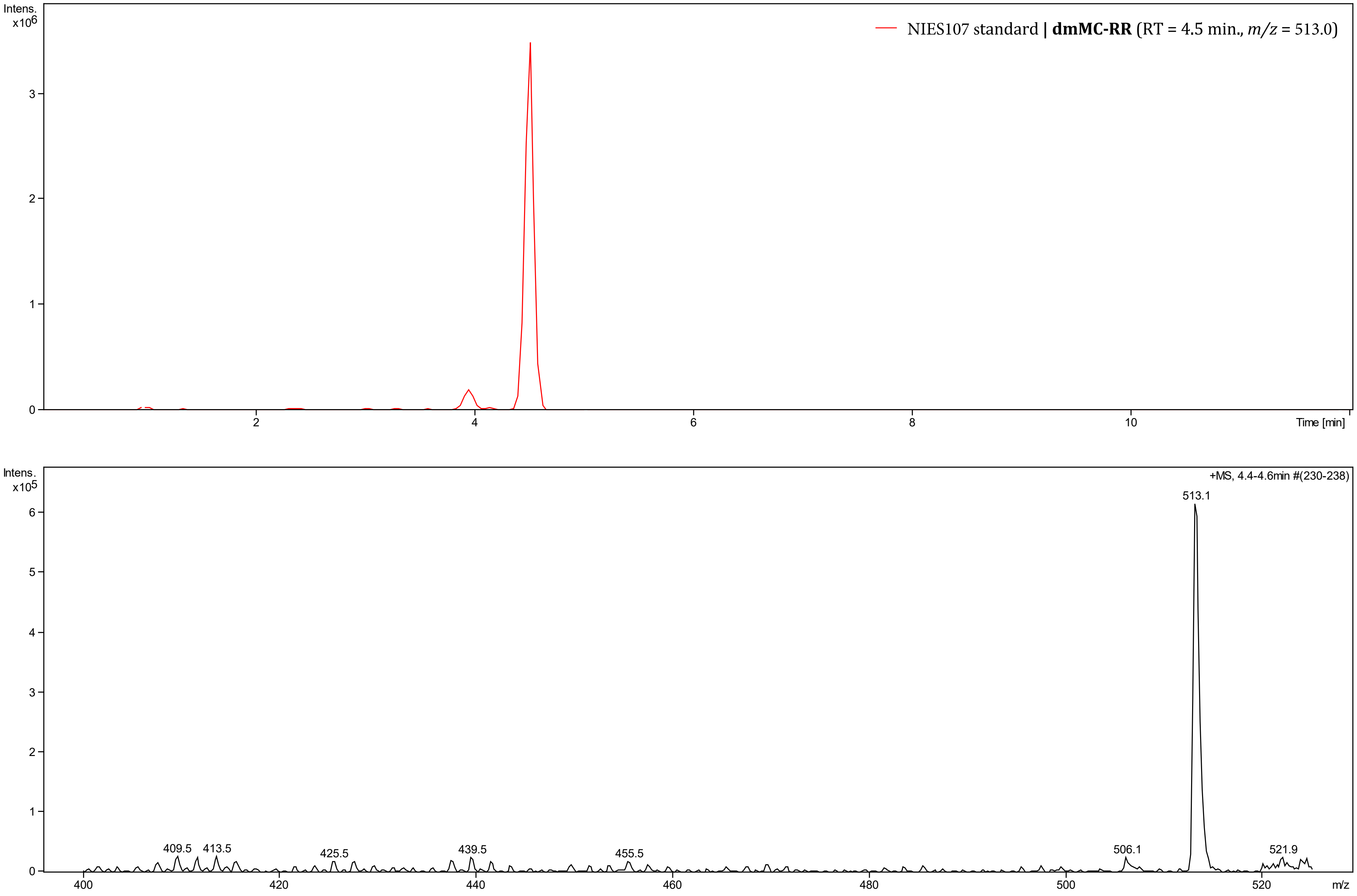

LC-MS analysis | extracted ion chromatogram (*m/z* 520.0) of NIES107 standard and mass spectrum scan of the MC-RR in the standard

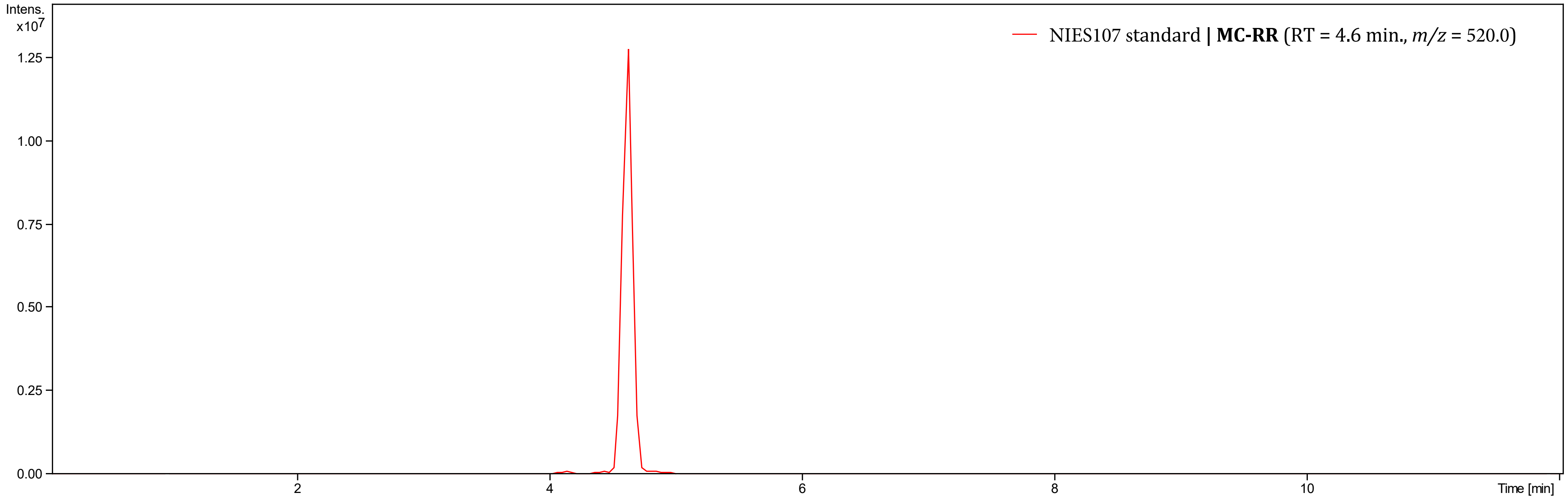

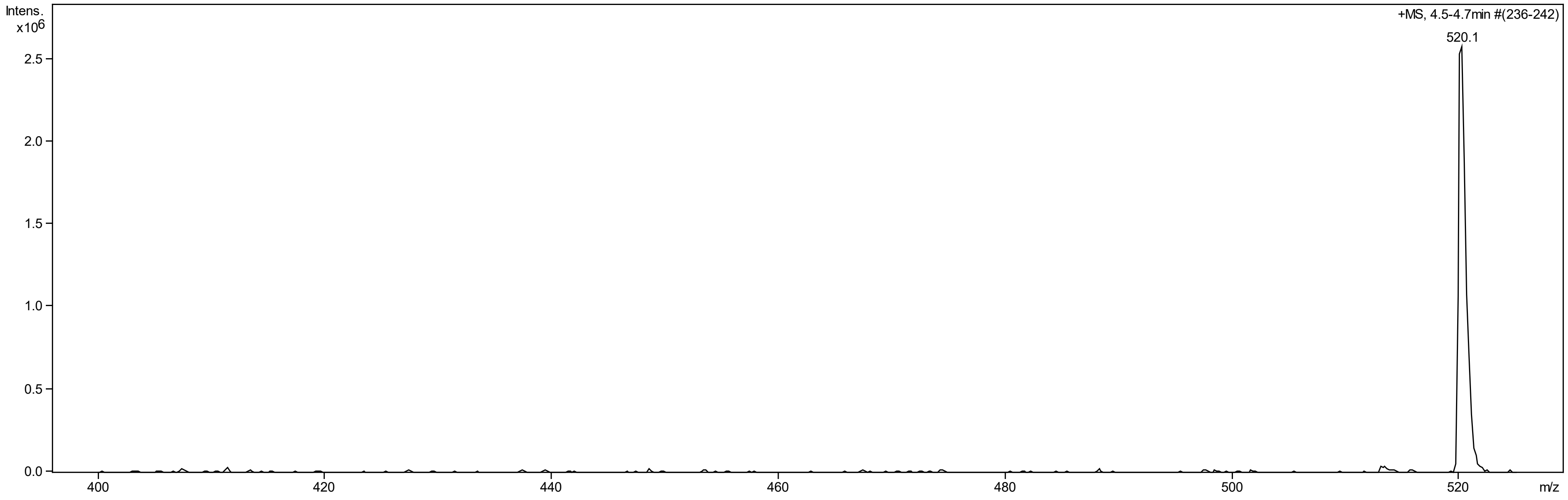

LC-MS analysis | extracted ion chromatogram (m/z 1045.6) of NIES107 standard and mass spectrum scan of the MC-YR in the standard

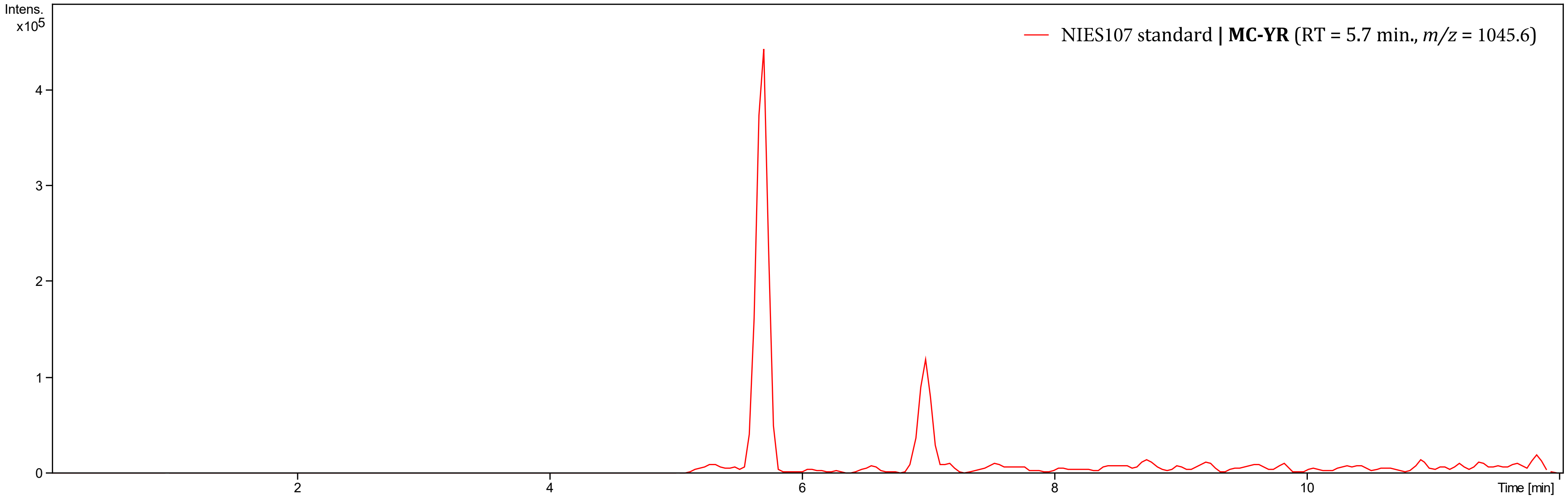

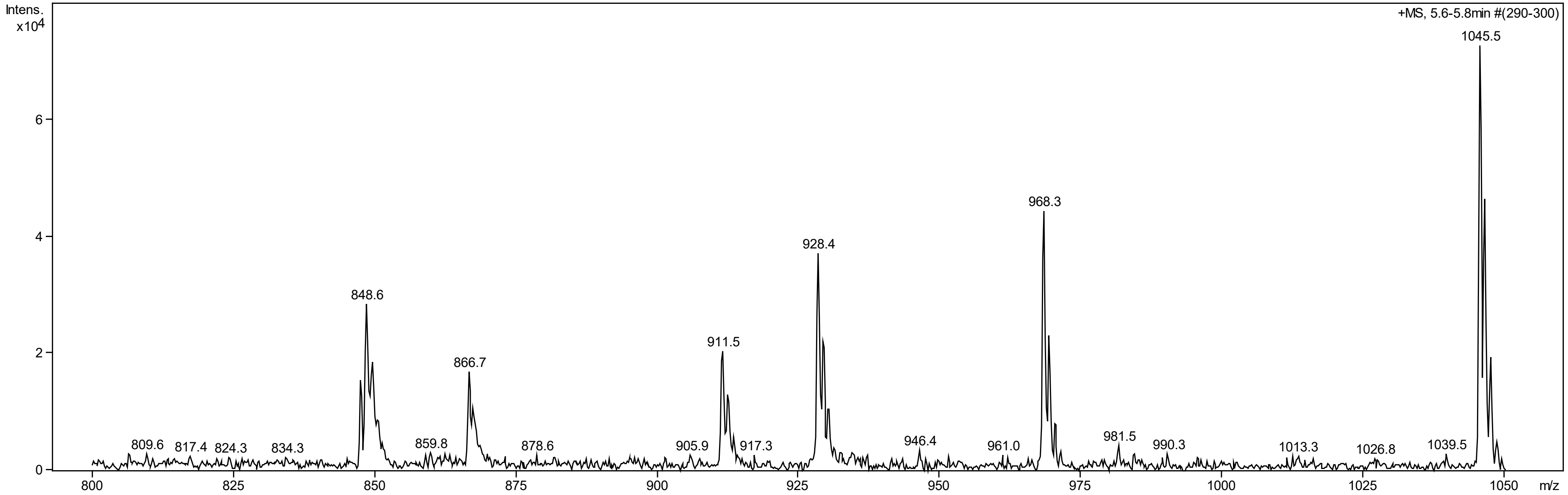

LC-MS analysis | extracted ion chromatogram (*m/z* 995.5) of PCC7820 standard and mass spectrum scan of the MC-LR in the standard

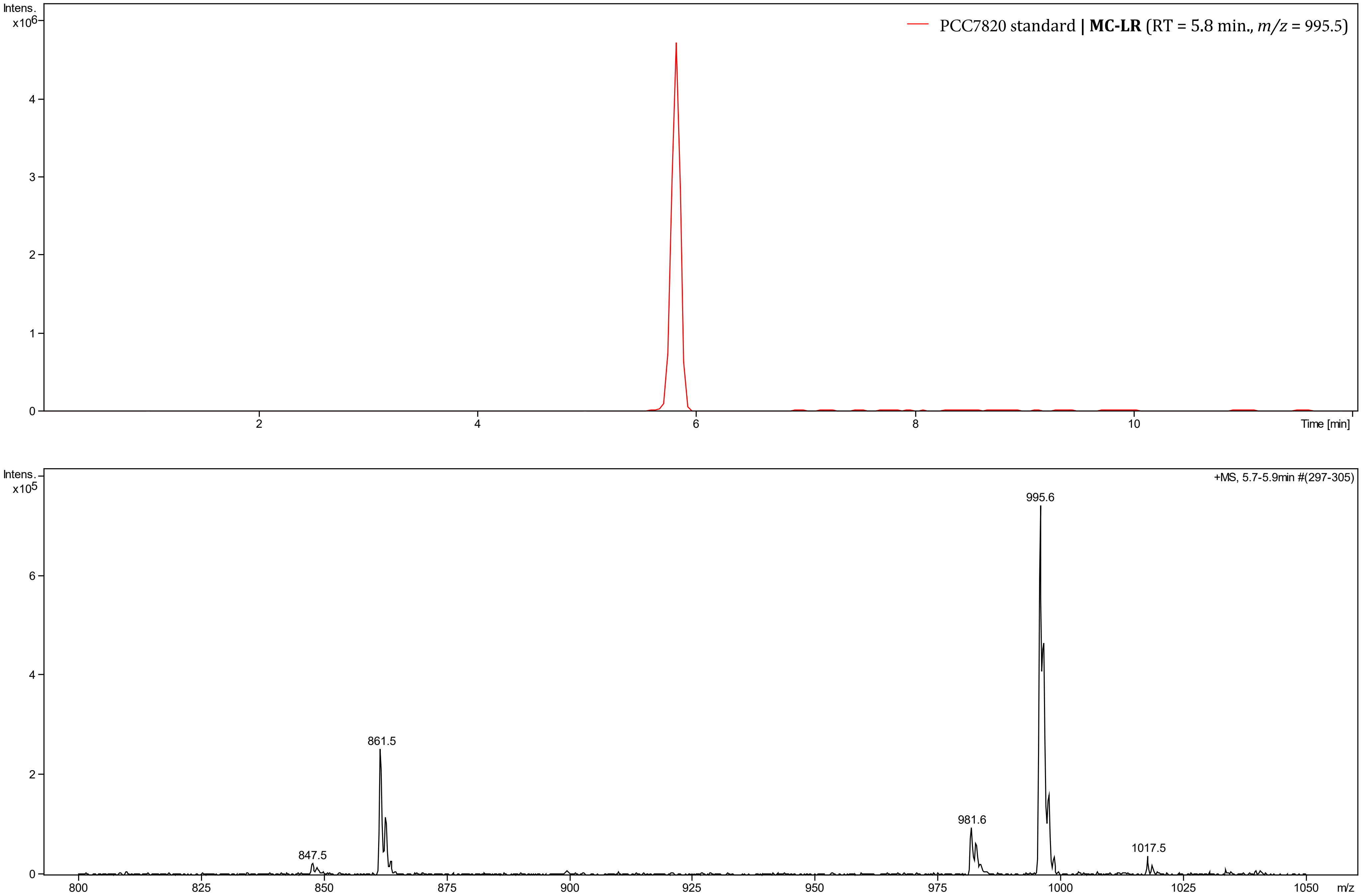

LC-MS analysis | extracted ion chromatogram (m/z 1002.5) of PCC7820 standard and mass spectrum scan of the MC-LY in the standard

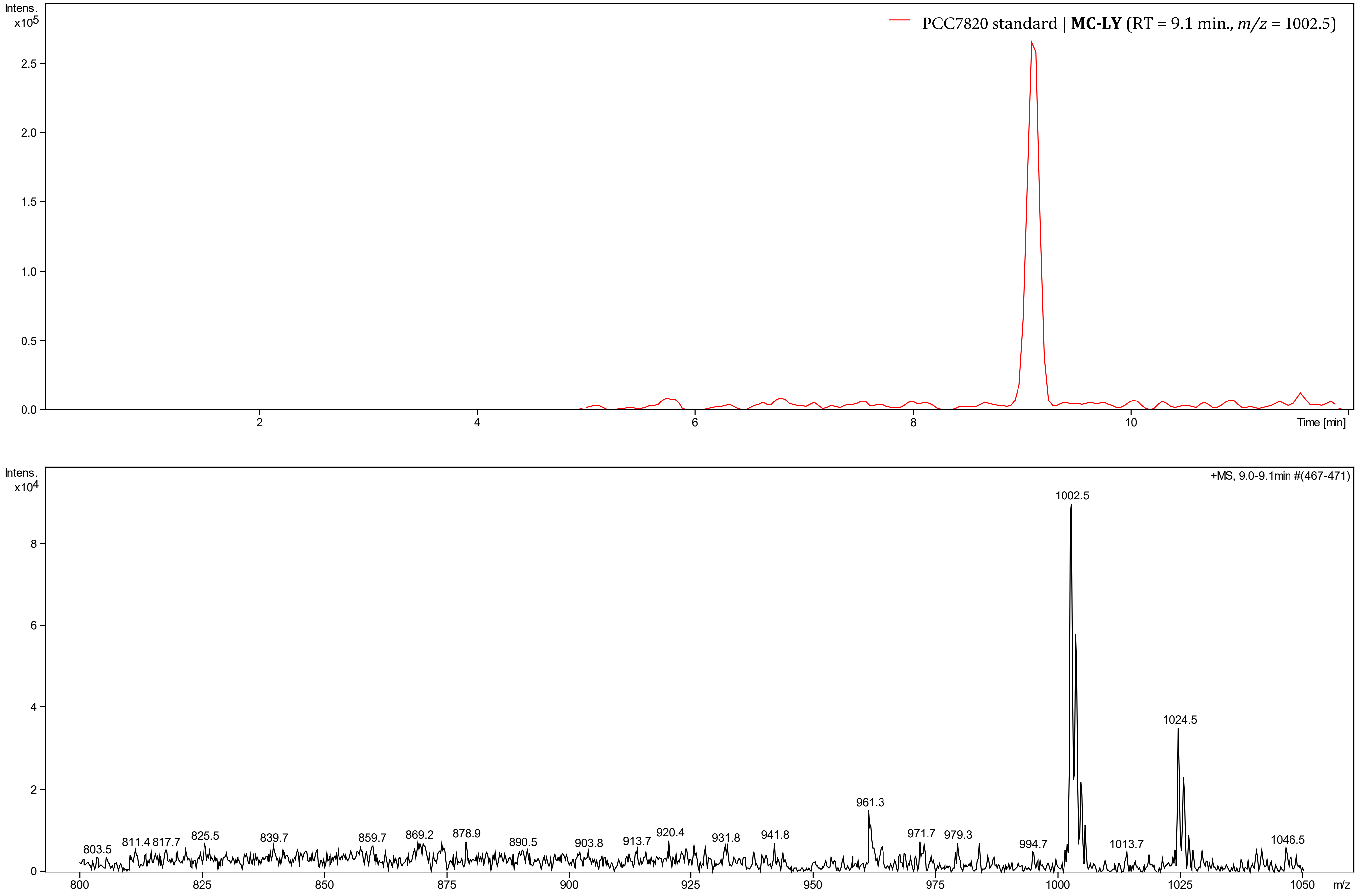

LC-MS analysis | extracted ion chromatogram (m/z 1025.9) of PCC7820 standard and mass spectrum scan of the MC-LW in the standard

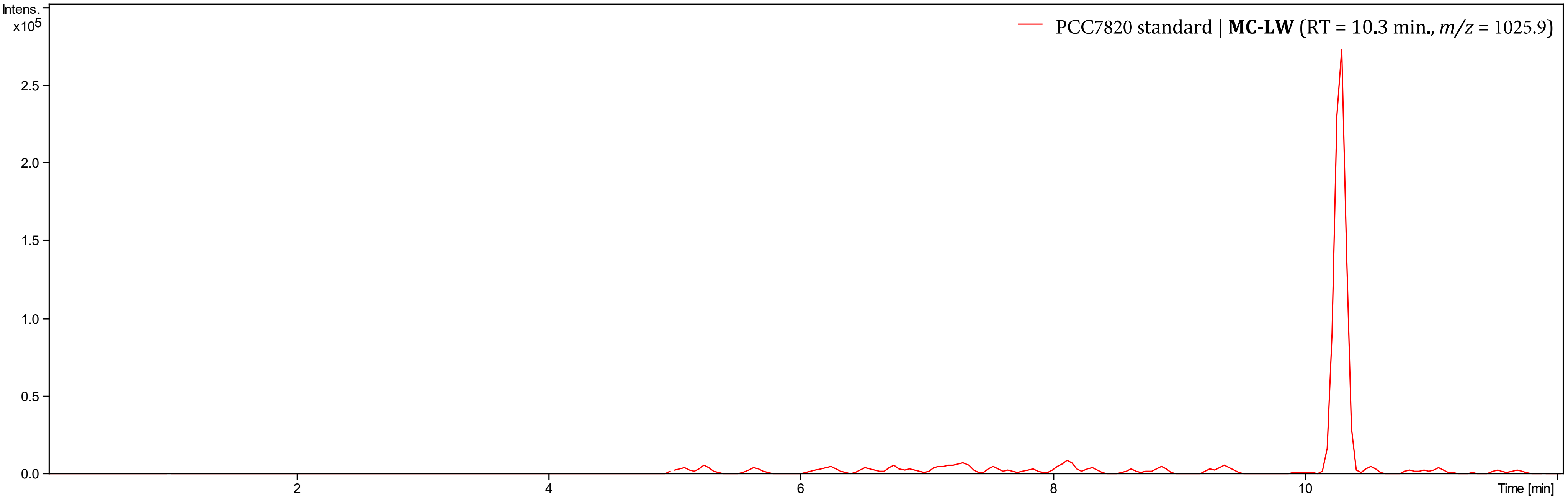

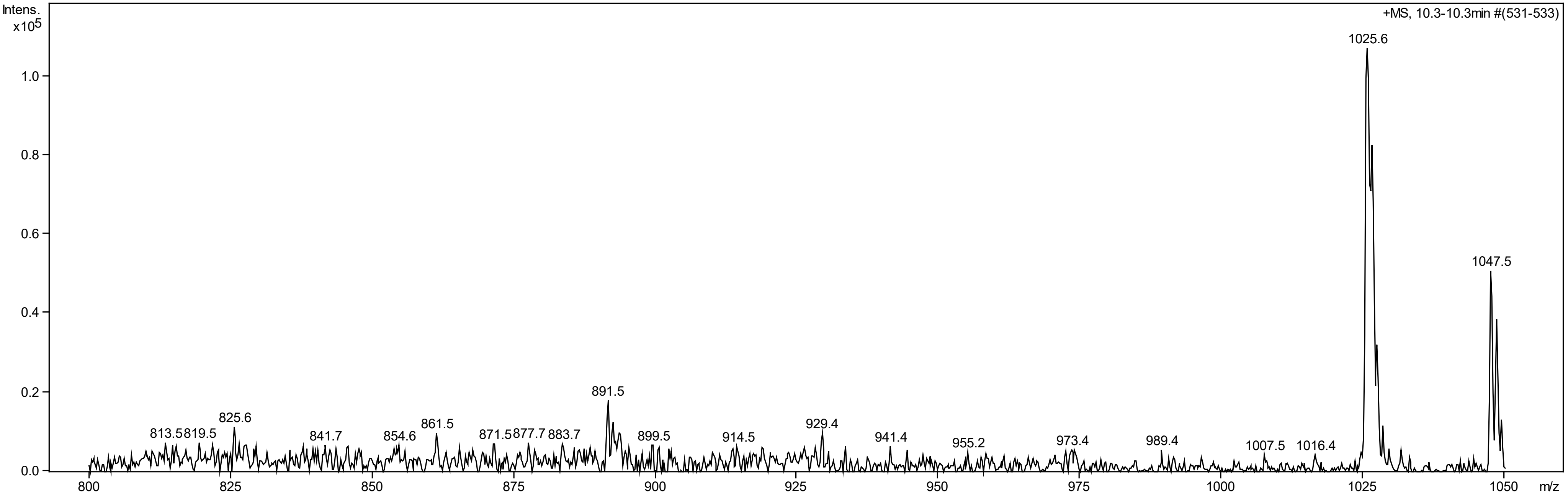

LC-MS analysis | extracted ion chromatogram (m/z 986.6) of PCC7820 standard and mass spectrum scan of the MC-LF in the standard

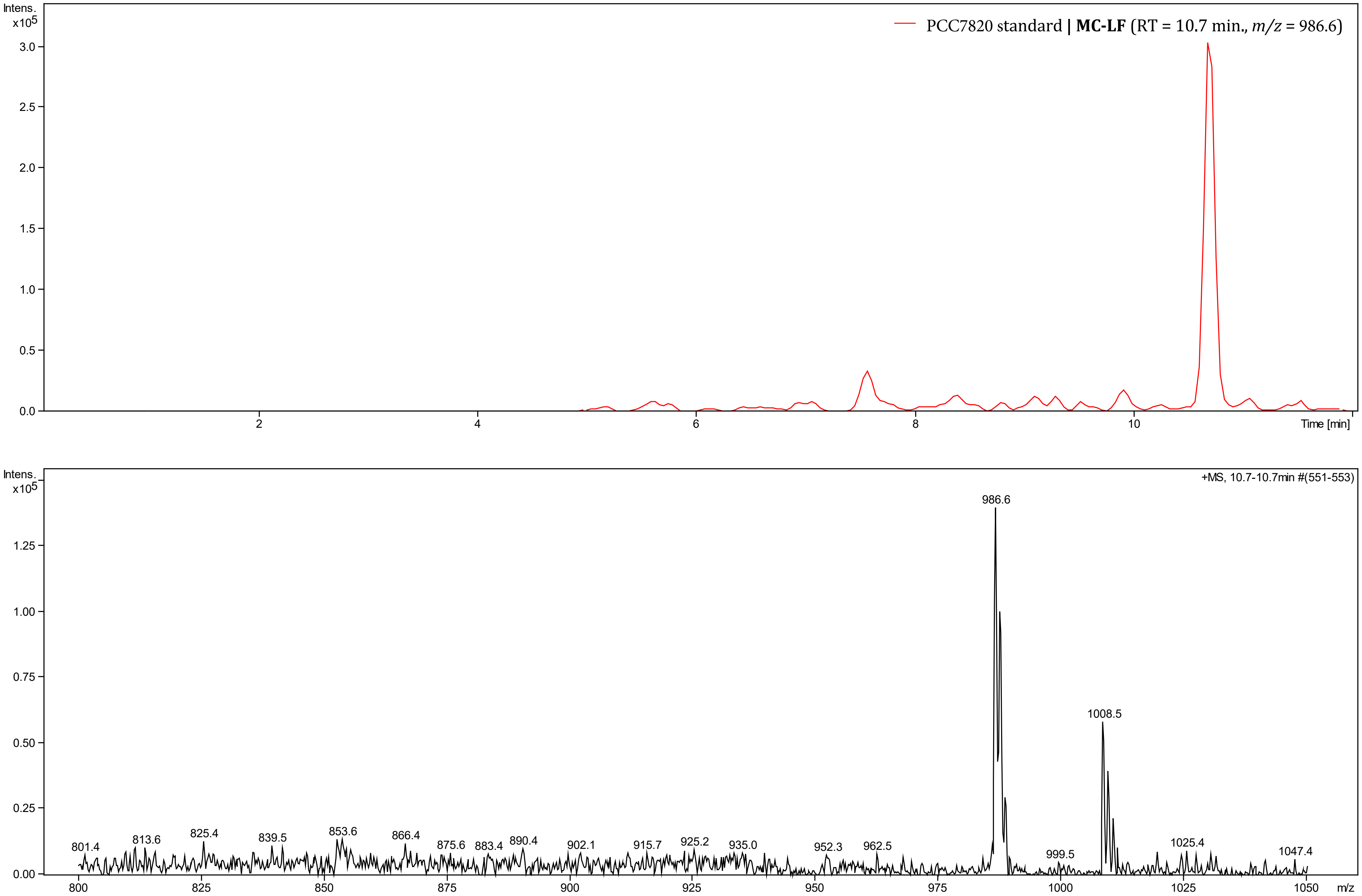

***P. agardhii* W67**

LC-MS analysis | extracted ion chromatogram (*m/z* 513.0) of NIES107 standard, *P. agardhii* W67, and mass spectrum scan of the dmMC-RR in W67 strain

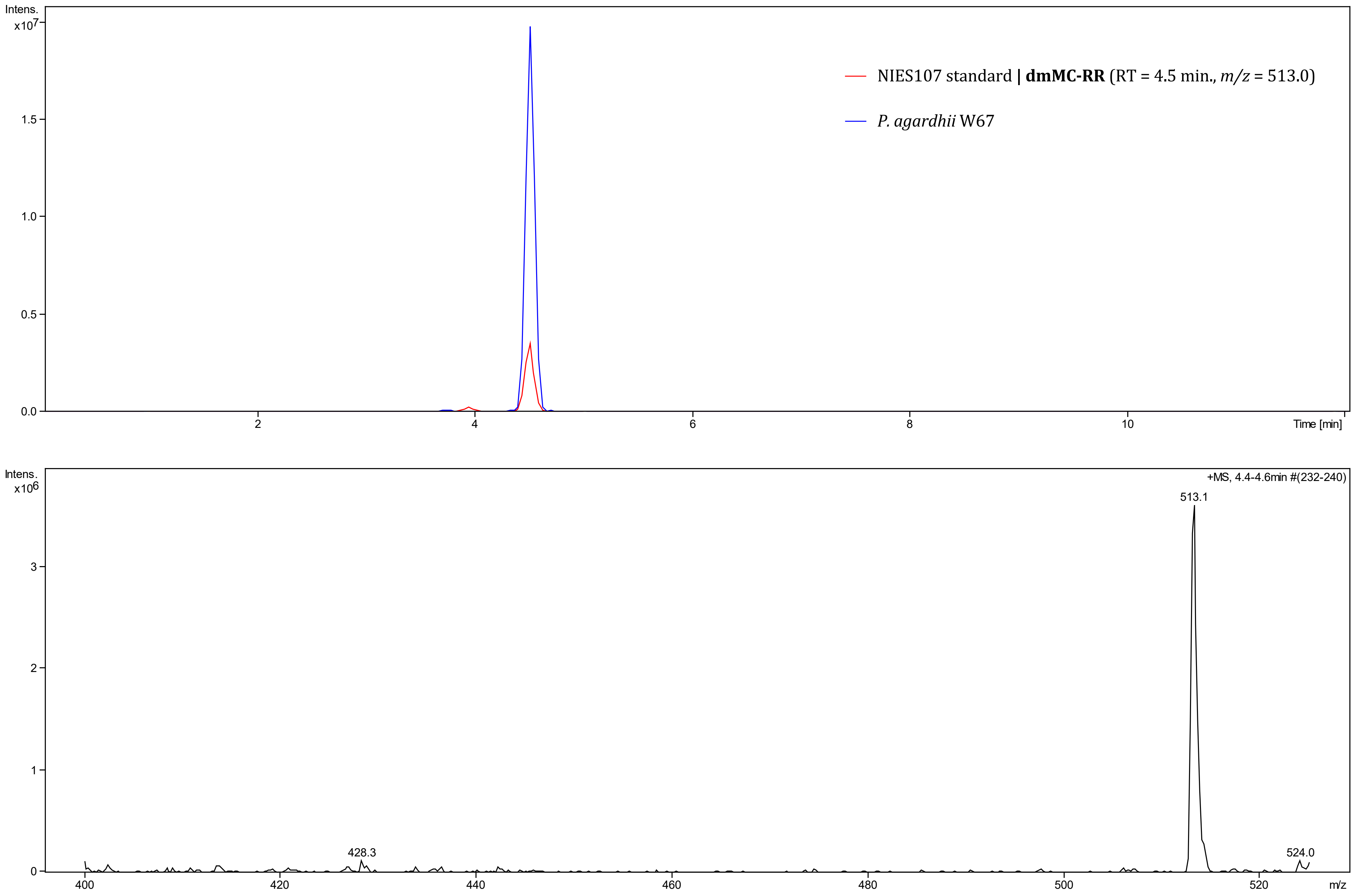

LC-MS analysis | extracted ion chromatogram (*m/z* 520.0) of NIES107 standard and *P. agardhii* W67

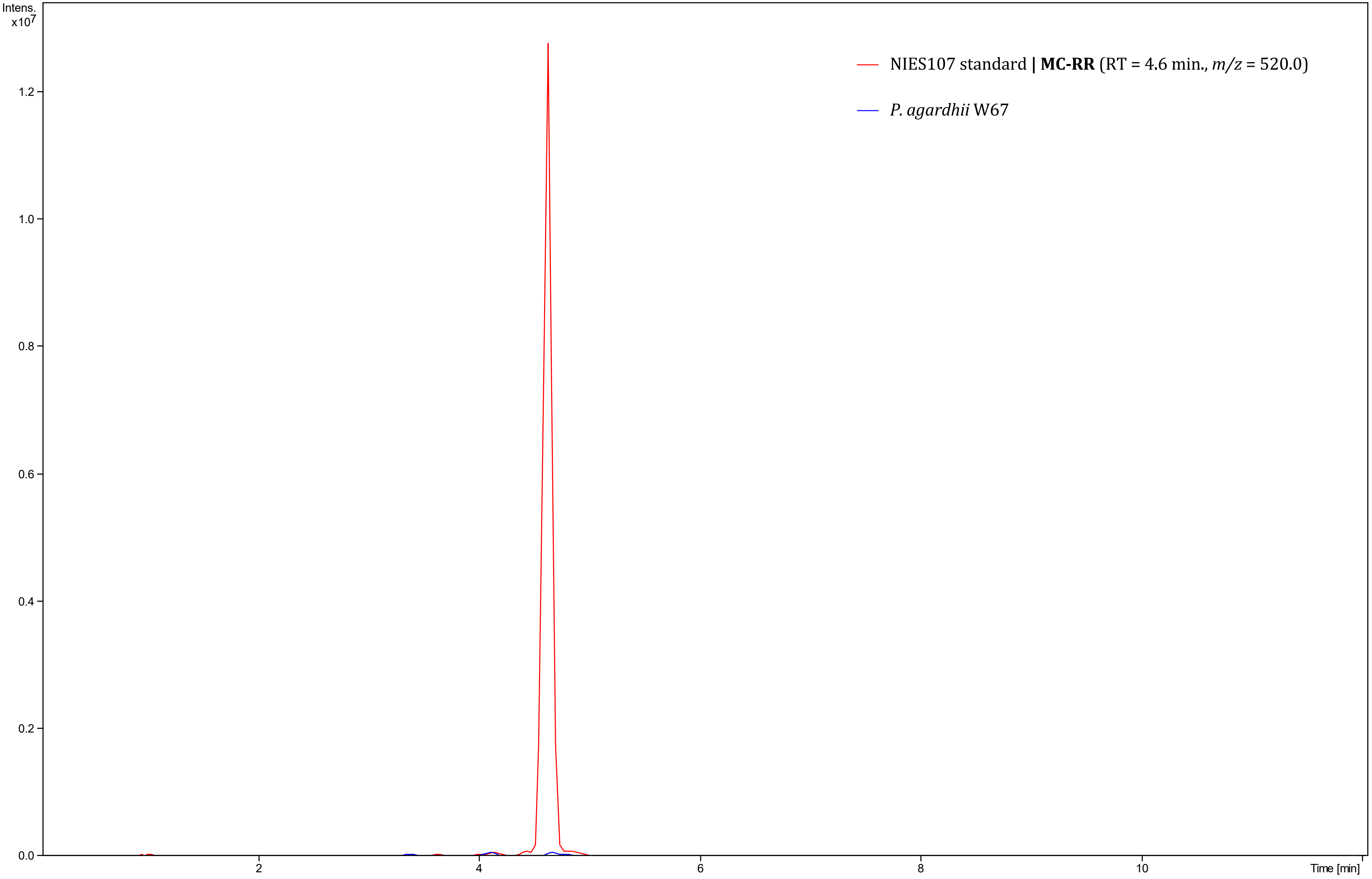

LC-MS analysis | extracted ion chromatogram (*m/z* 1045.6) of NIES107 standard, *P. agardhii* W67, and mass spectrum scan of the MC-YR in W67 strain

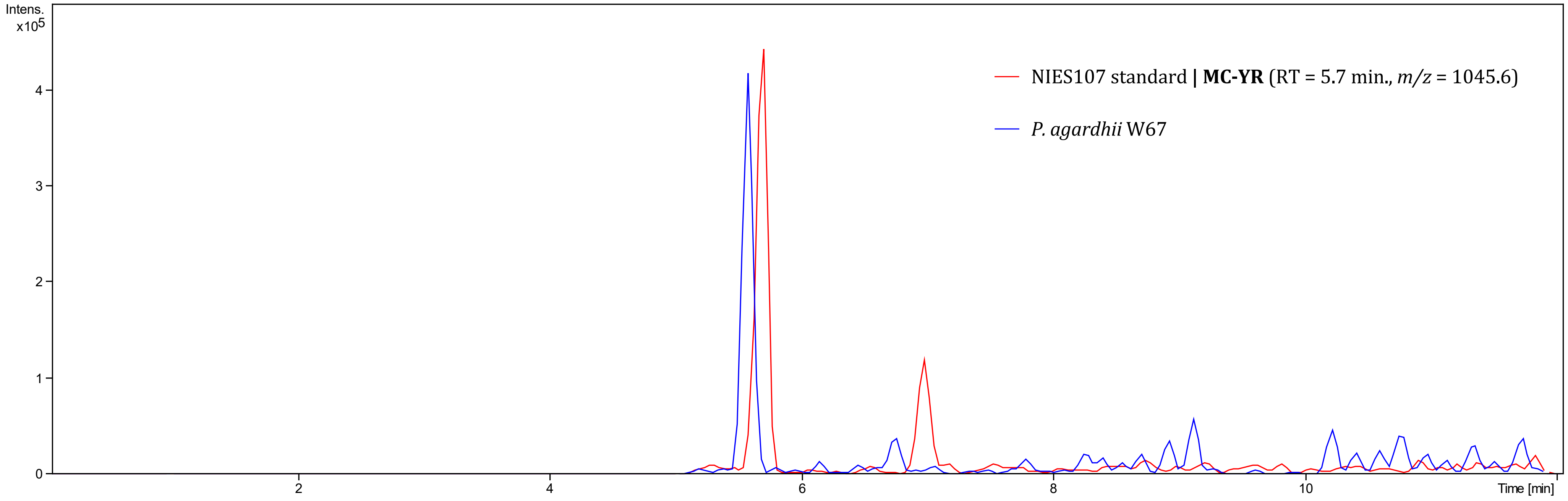

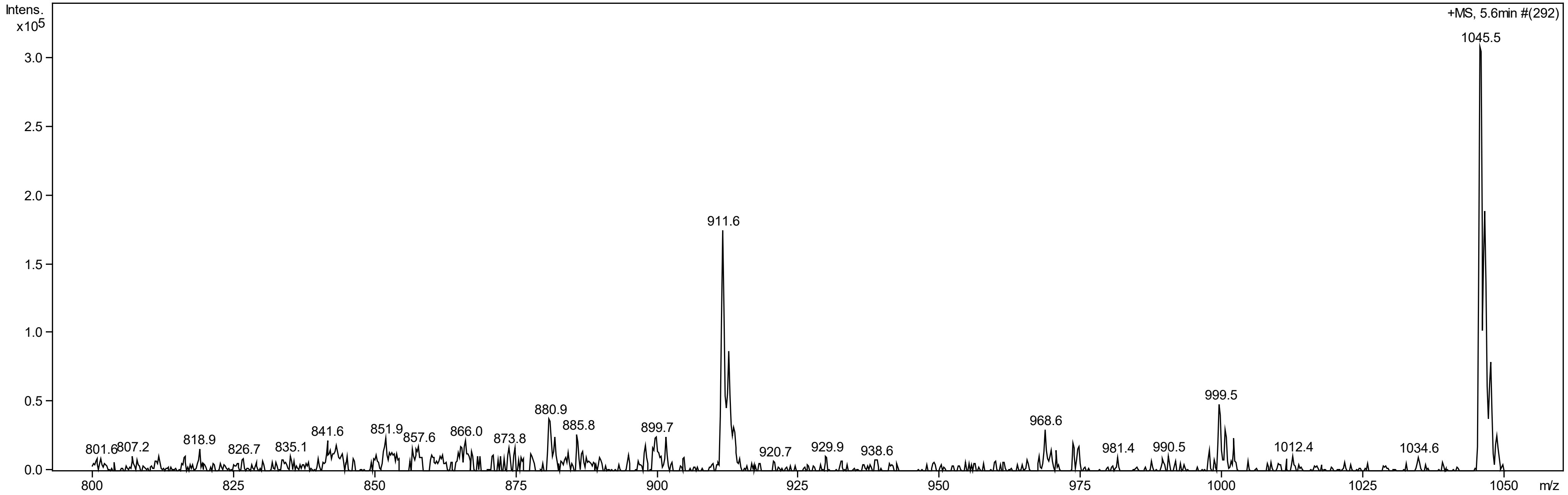

LC-MS analysis | extracted ion chromatogram (*m/z* 995.5) of PCC7820 standard and *P. agardhii* W67

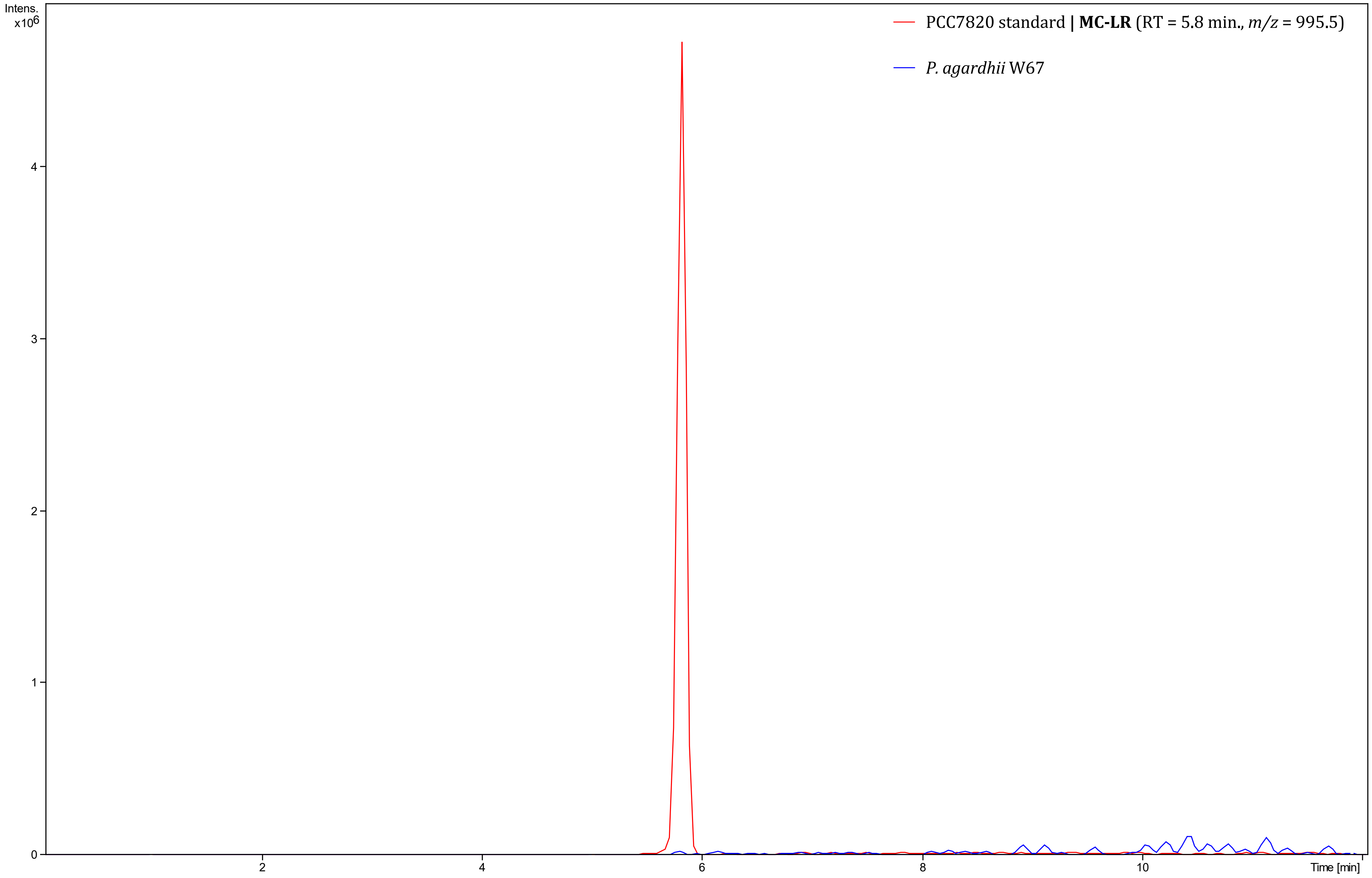

LC-MS analysis | extracted ion chromatogram (*m/z* 1002.5) of PCC7820 standard and *P. agardhii* W67

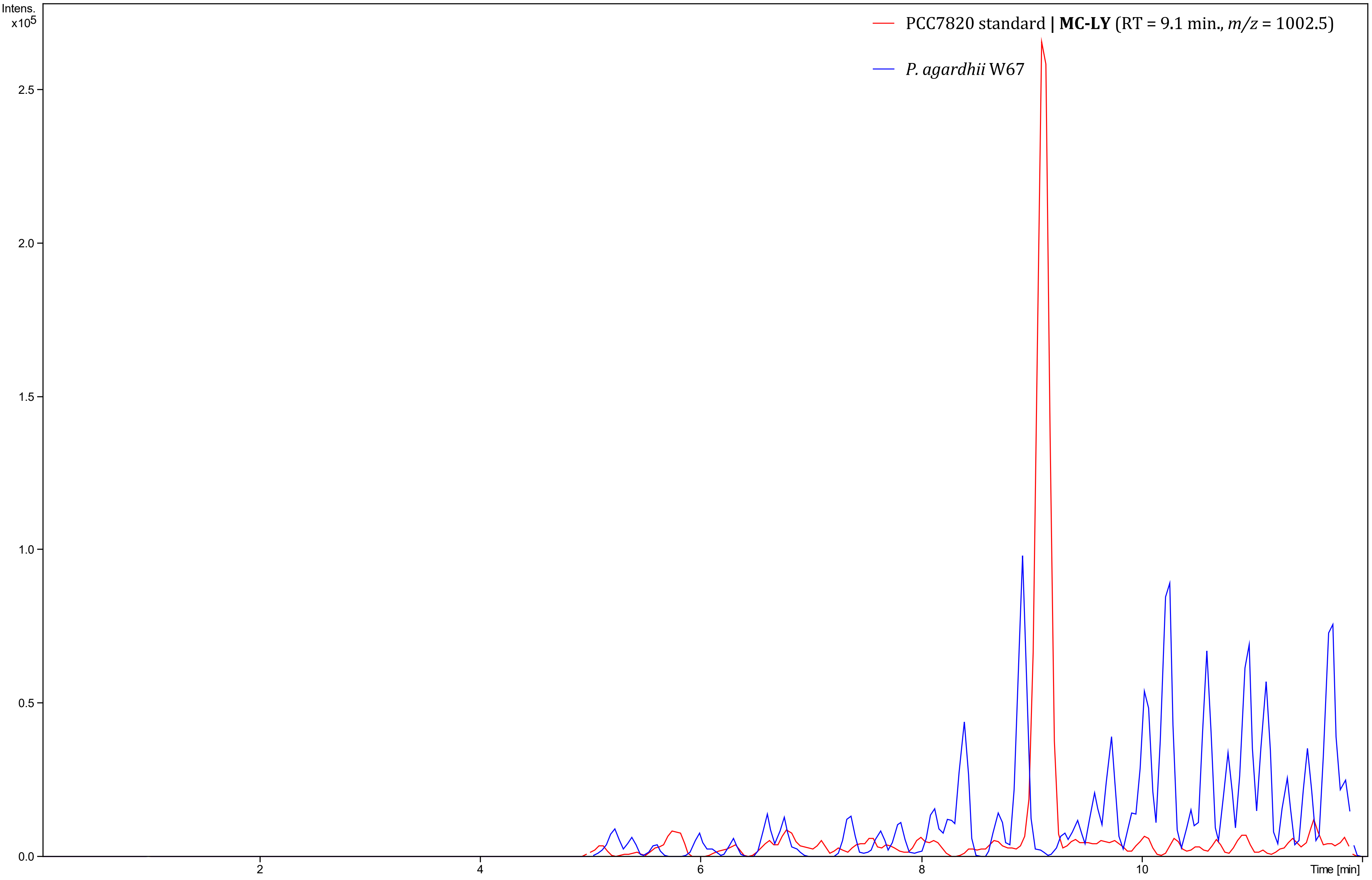

LC-MS analysis | extracted ion chromatogram (*m/z* 1025.9) of PCC7820 standard and *P. agardhii* W67

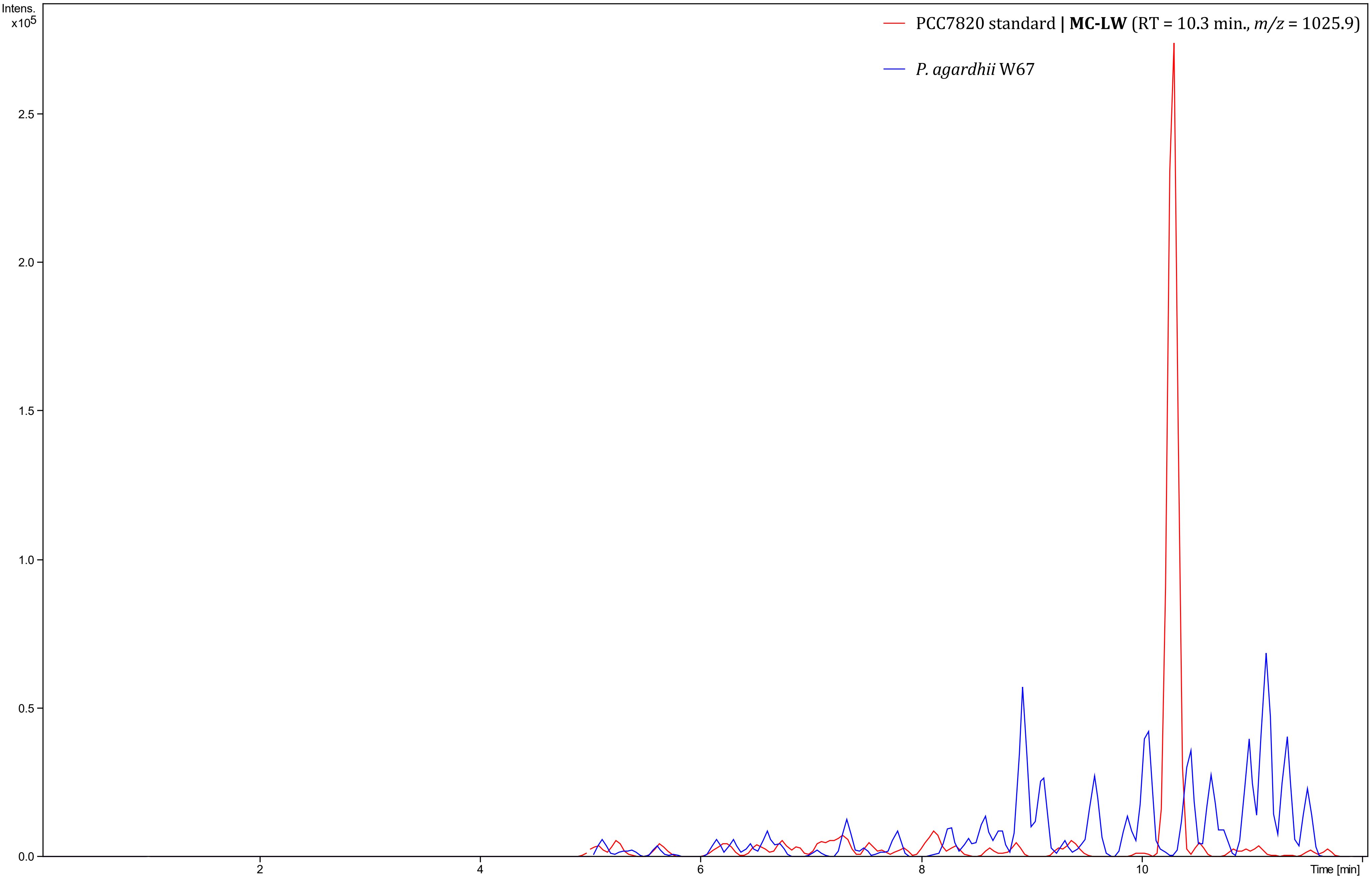

LC-MS analysis | extracted ion chromatogram (*m/z* 986.6) of PCC7820 standard and *P. agardhii* W67

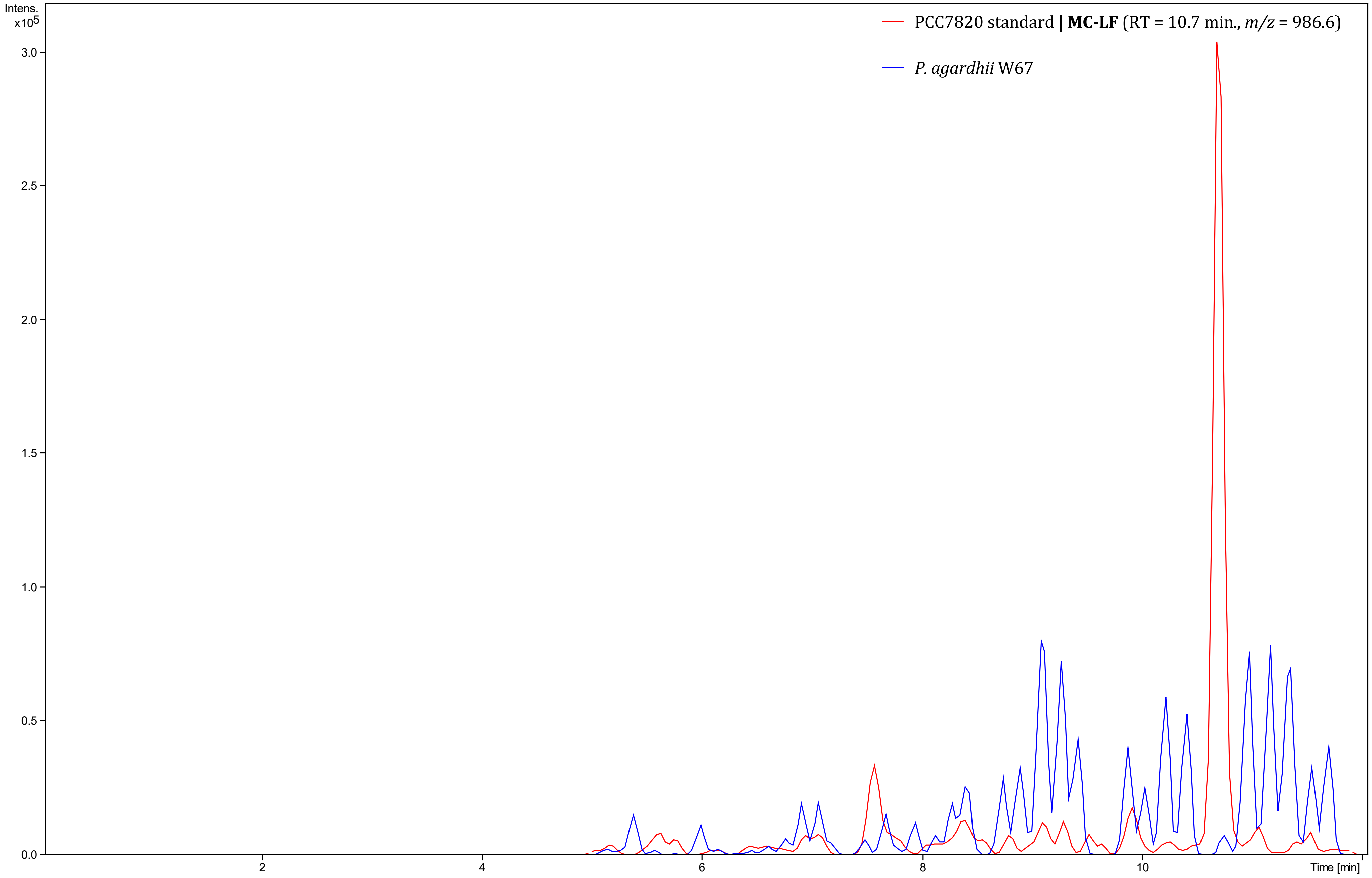

***P. agardhii* W49**

LC-MS analysis | extracted ion chromatogram (*m/z* 513.0) of NIES107 standard and *P. agardhii* W49

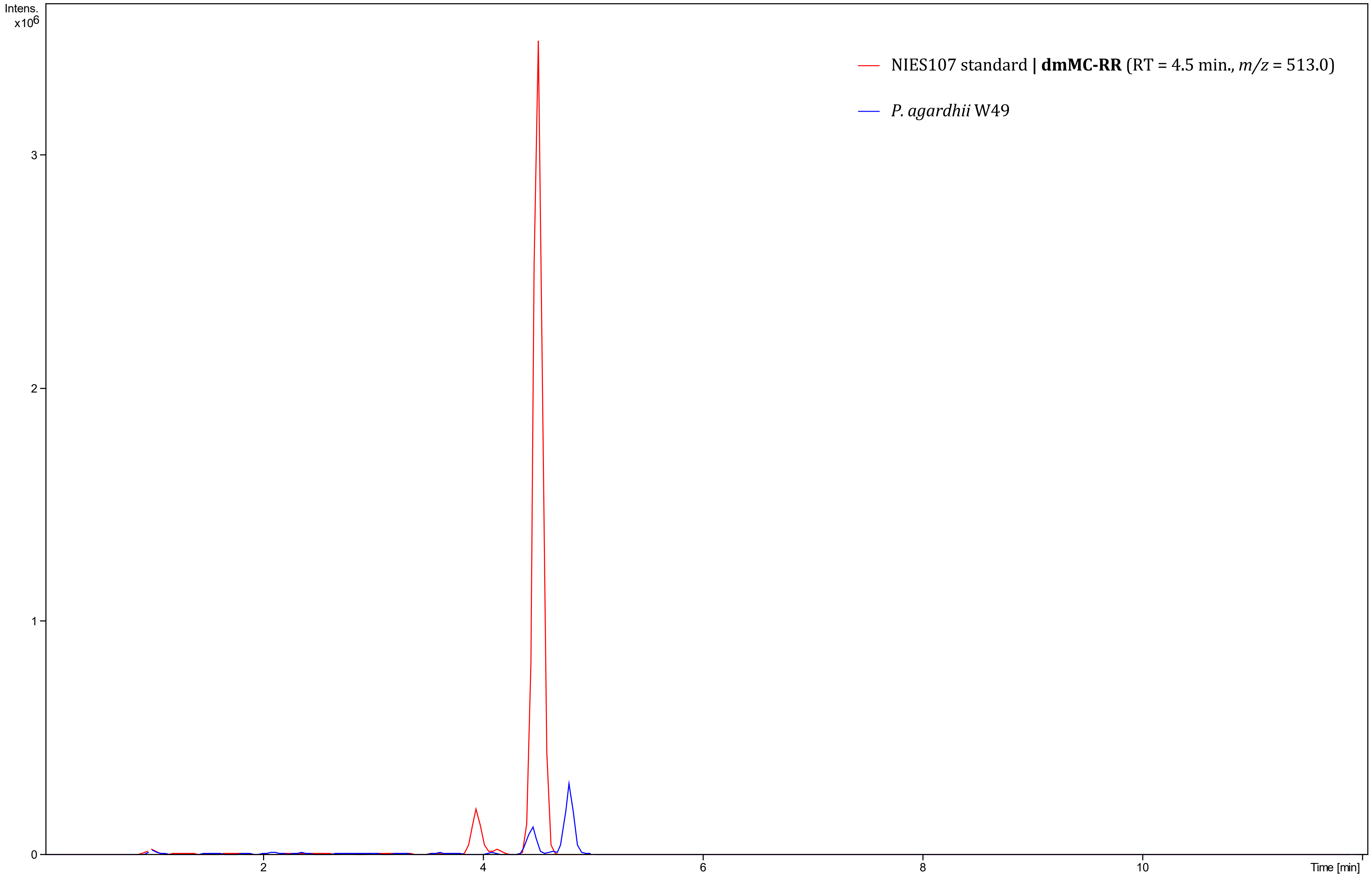

LC-MS analysis | extracted ion chromatogram (*m/z* 520.0) of NIES107 standard and *P. agardhii* W49

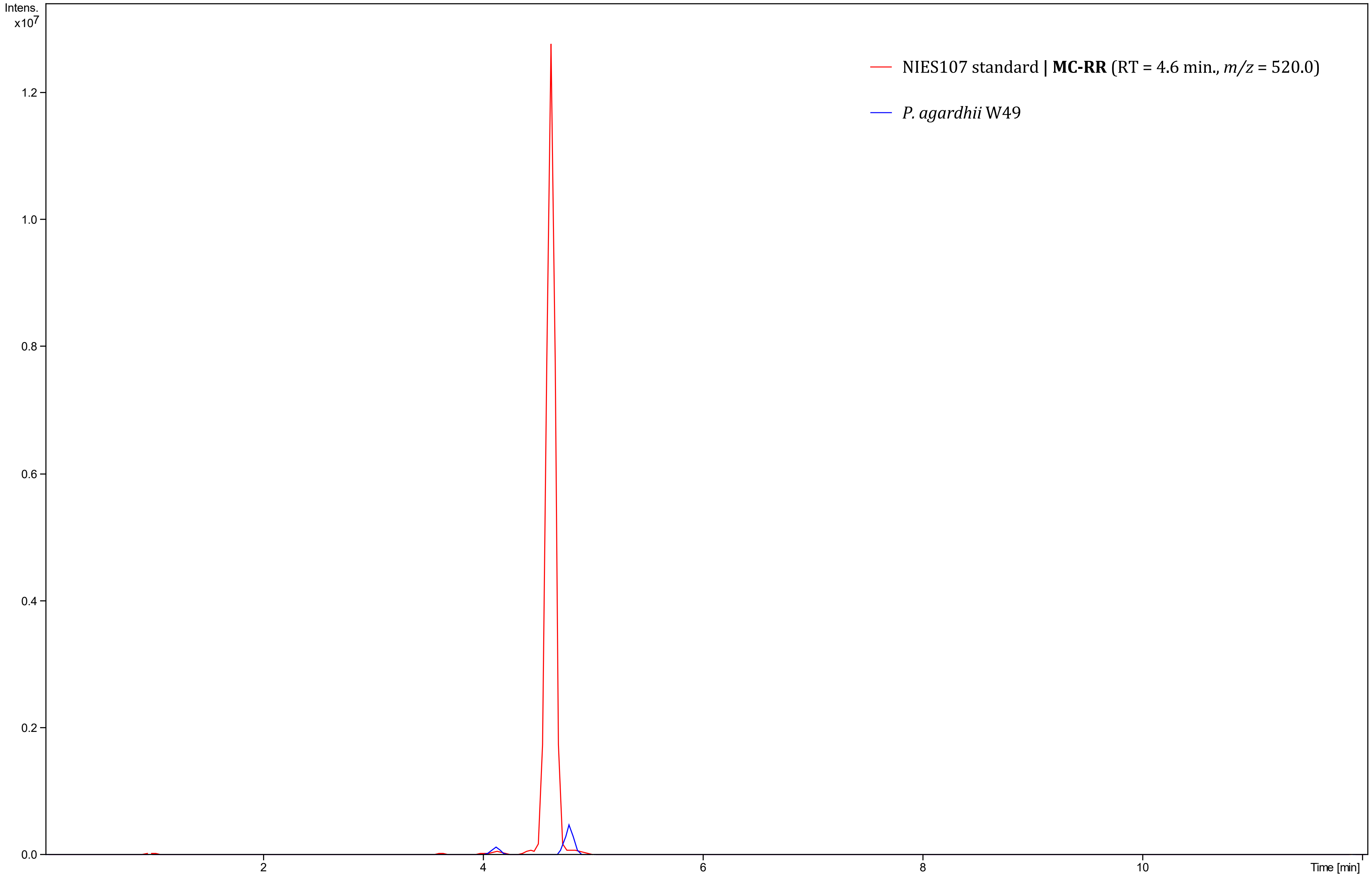

LC-MS analysis | extracted ion chromatogram (*m/z* 1045.6) of NIES107 standard and *P. agardhii* W49

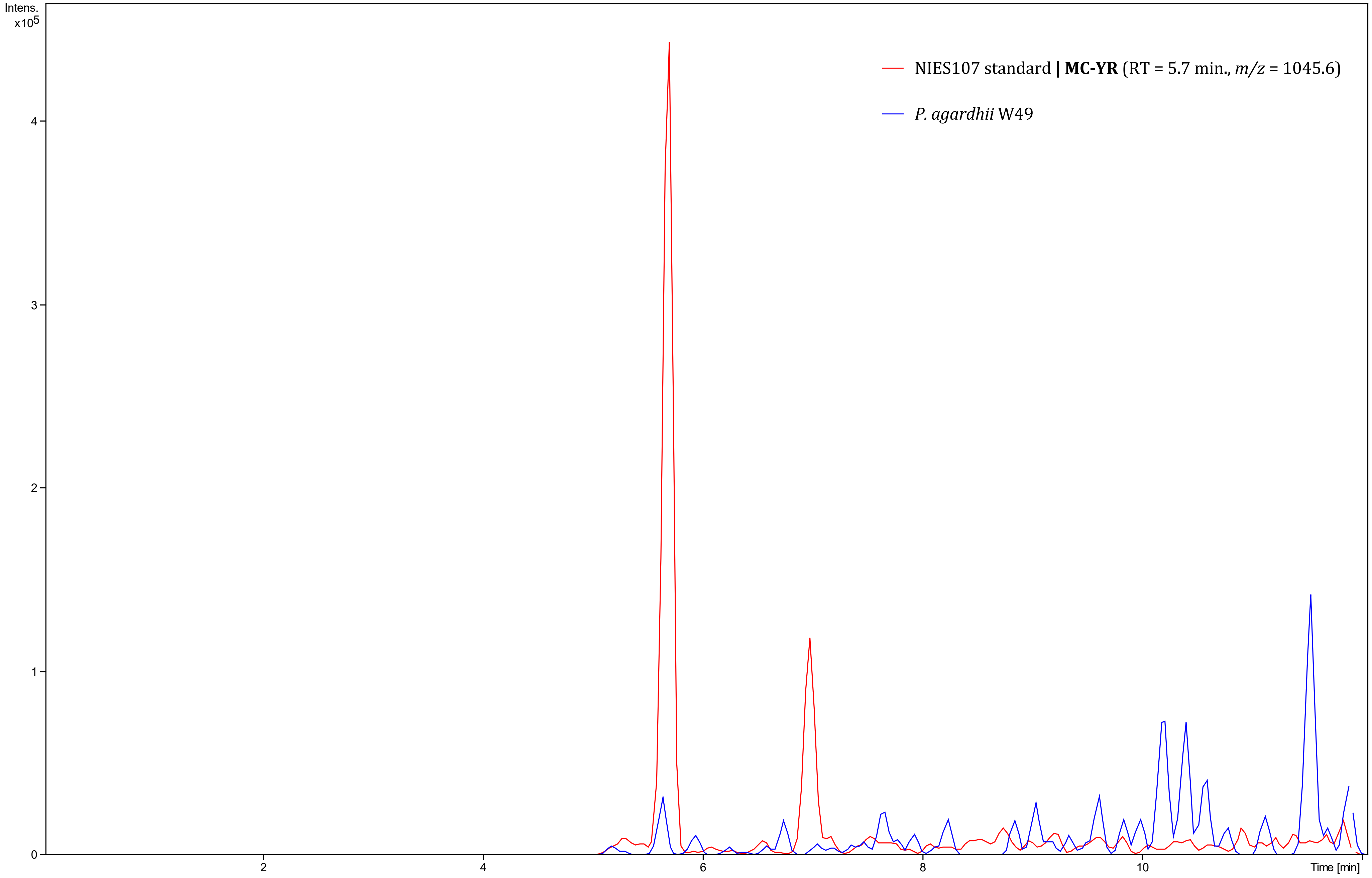

LC-MS analysis | extracted ion chromatogram (*m/z* 995.5) of PCC7820 standard and *P. agardhii* W49

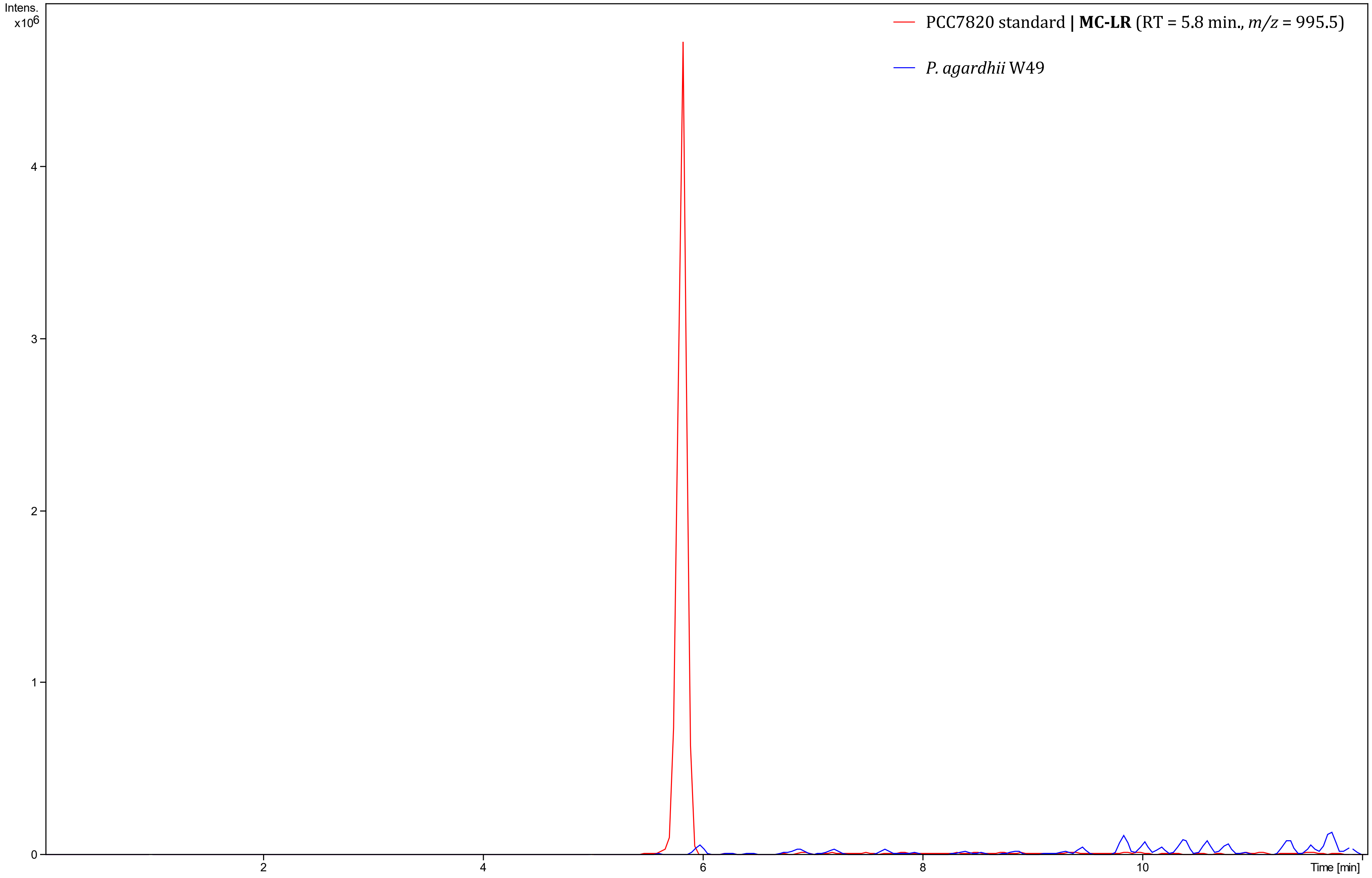

LC-MS analysis | extracted ion chromatogram (*m/z* 1002.5) of PCC7820 standard and *P. agardhii* W49

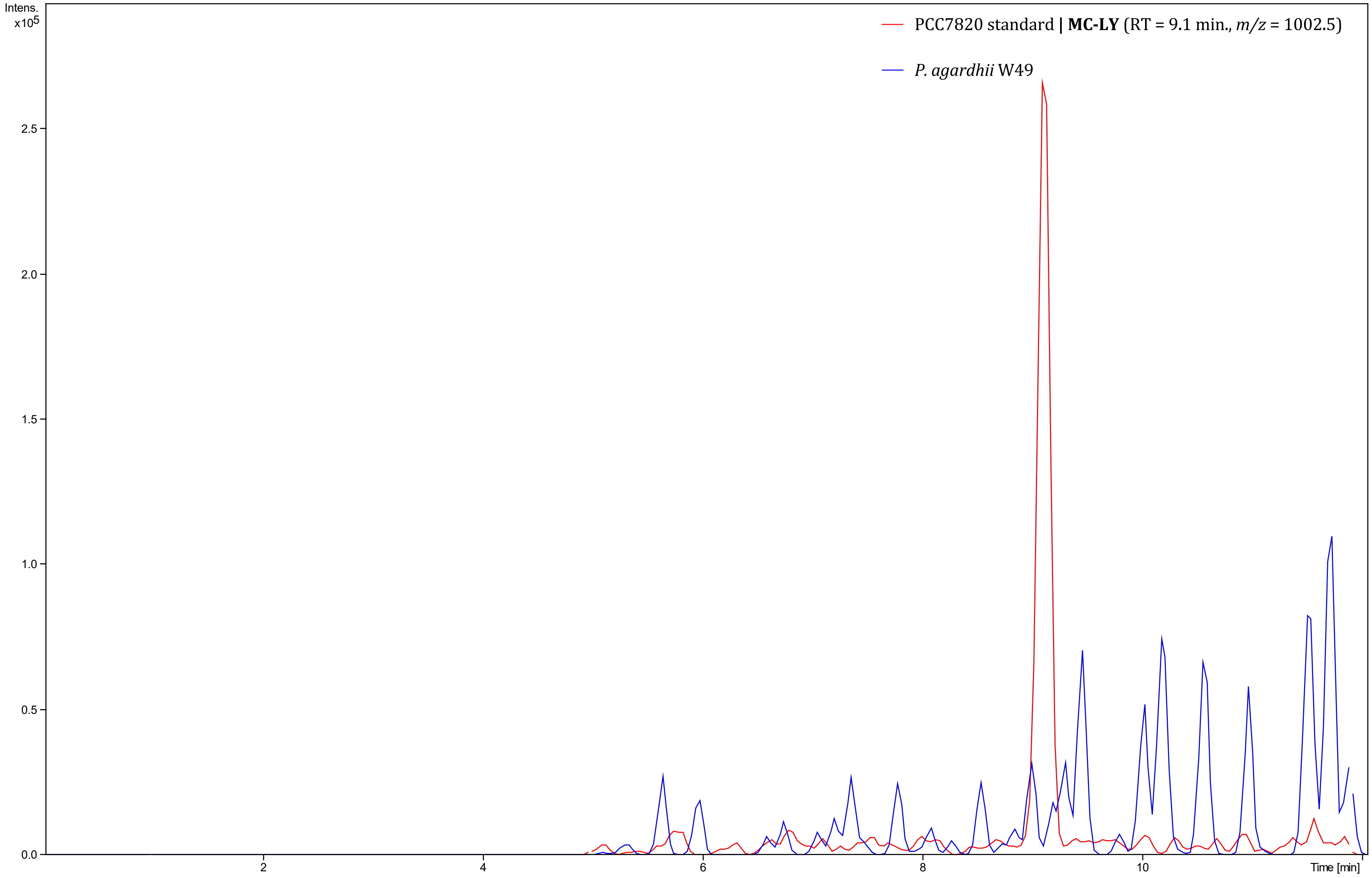

LC-MS analysis | extracted ion chromatogram (*m/z* 1025.9) of PCC7820 standard and *P. agardhii* W49

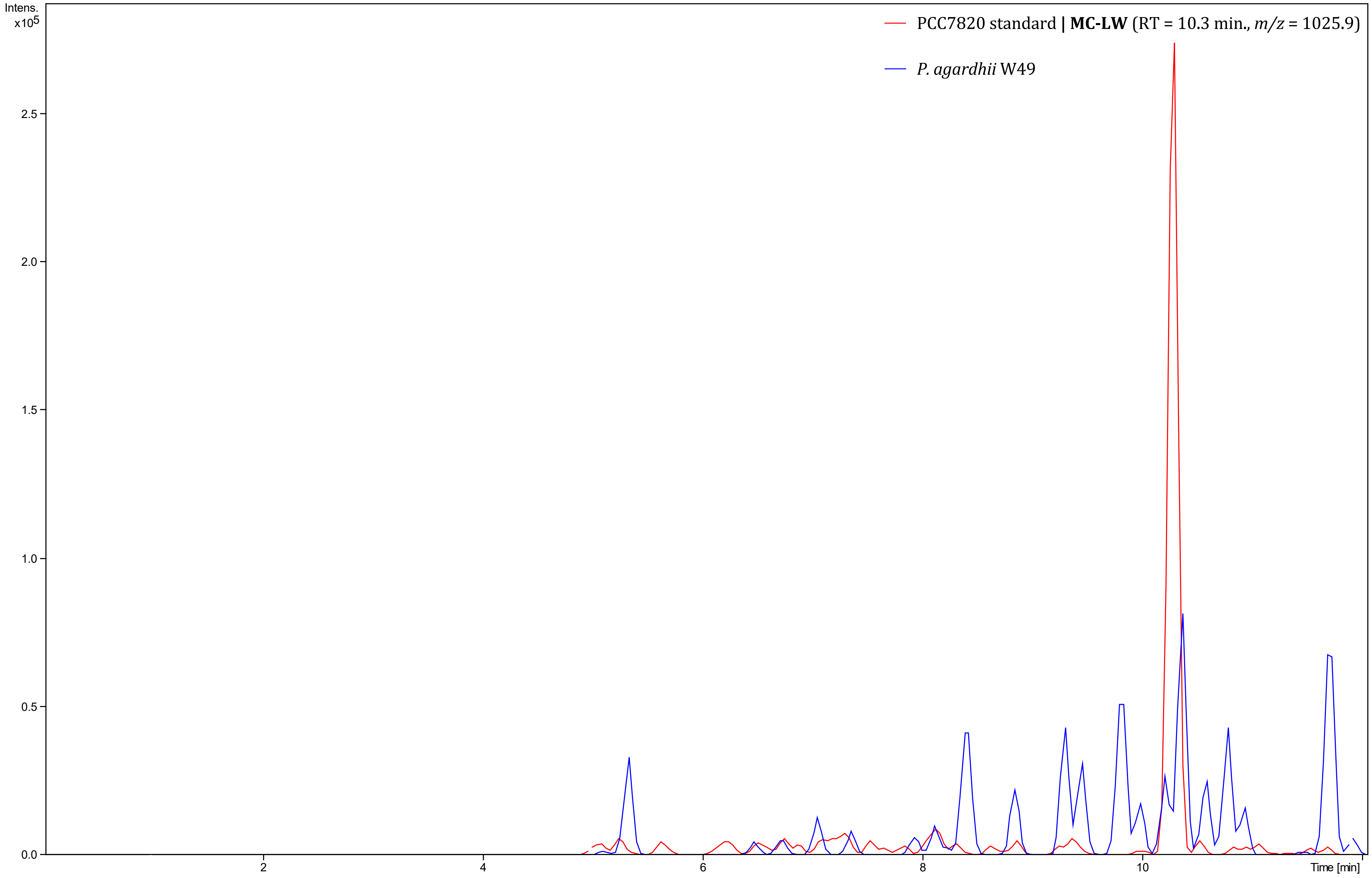

LC-MS analysis | extracted ion chromatogram (*m/z* 986.6) of PCC7820 standard and *P. agardhii* W49

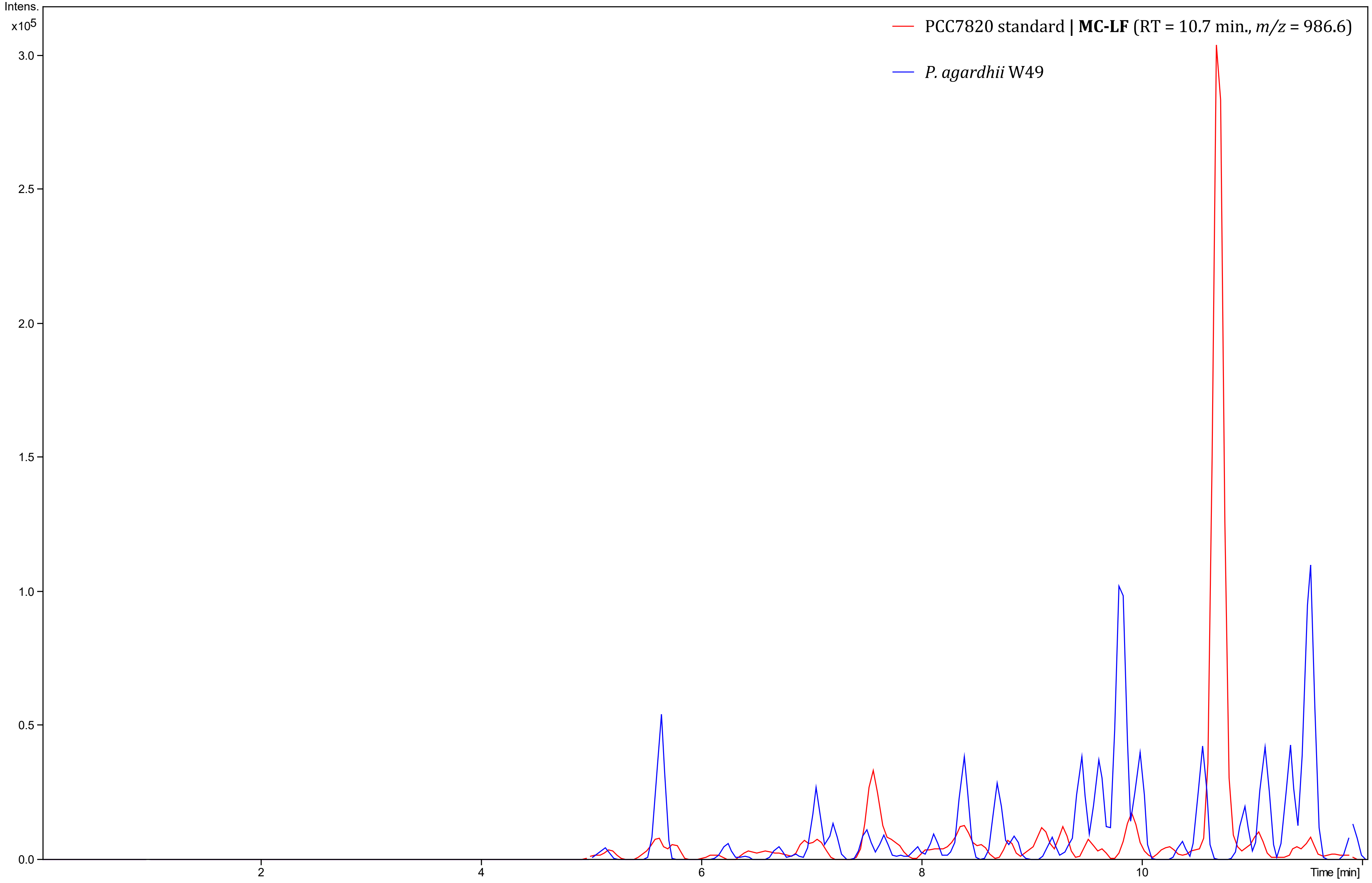

***R. raciborskii* W88**

LC-MS analysis | extracted ion chromatogram (*m/z* 513.0) of NIES107 standard and *R. raciborskii* W88

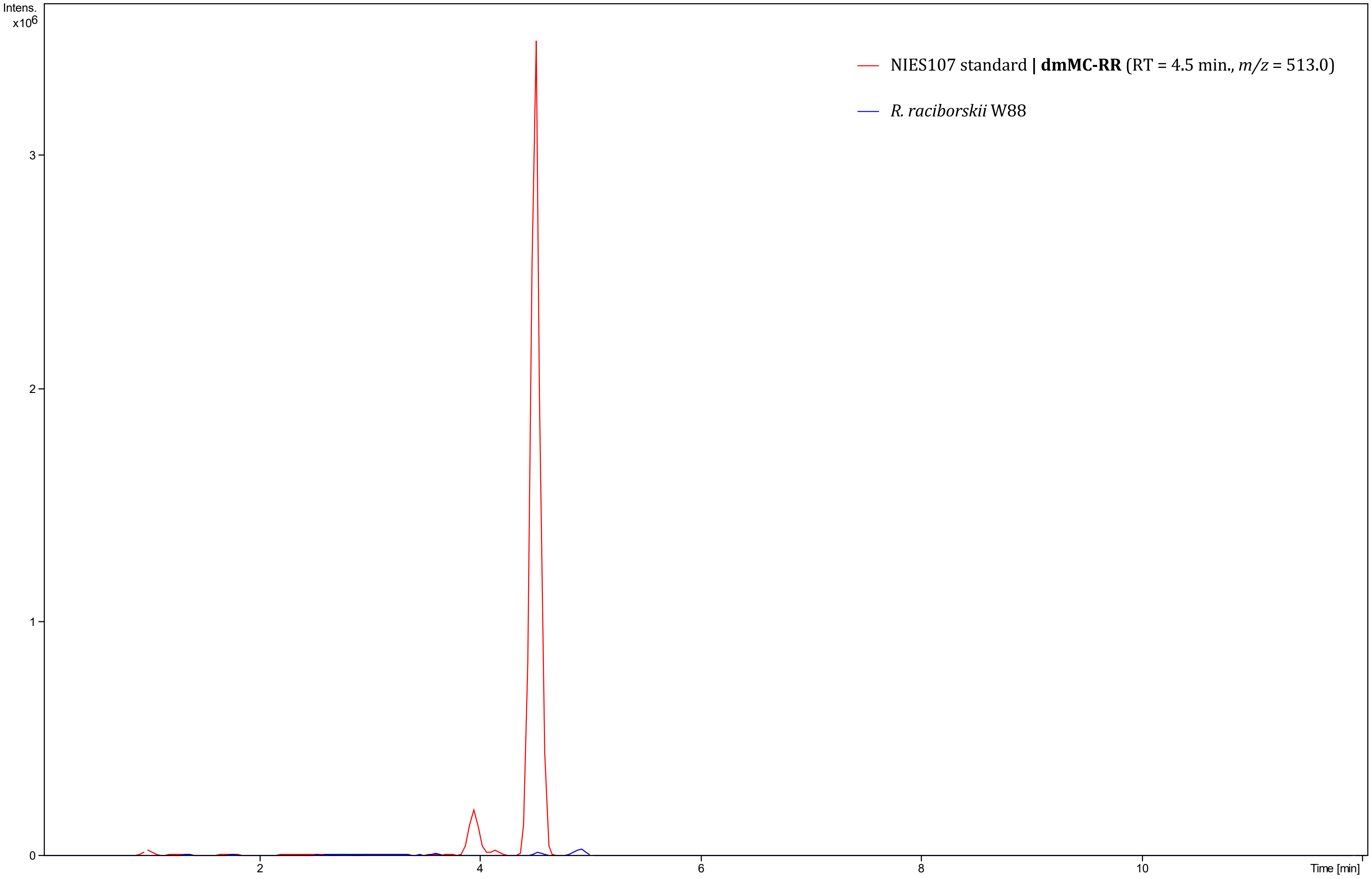

LC-MS analysis | extracted ion chromatogram (*m/z* 520.0) of NIES107 standard and *R. raciborskii* W88

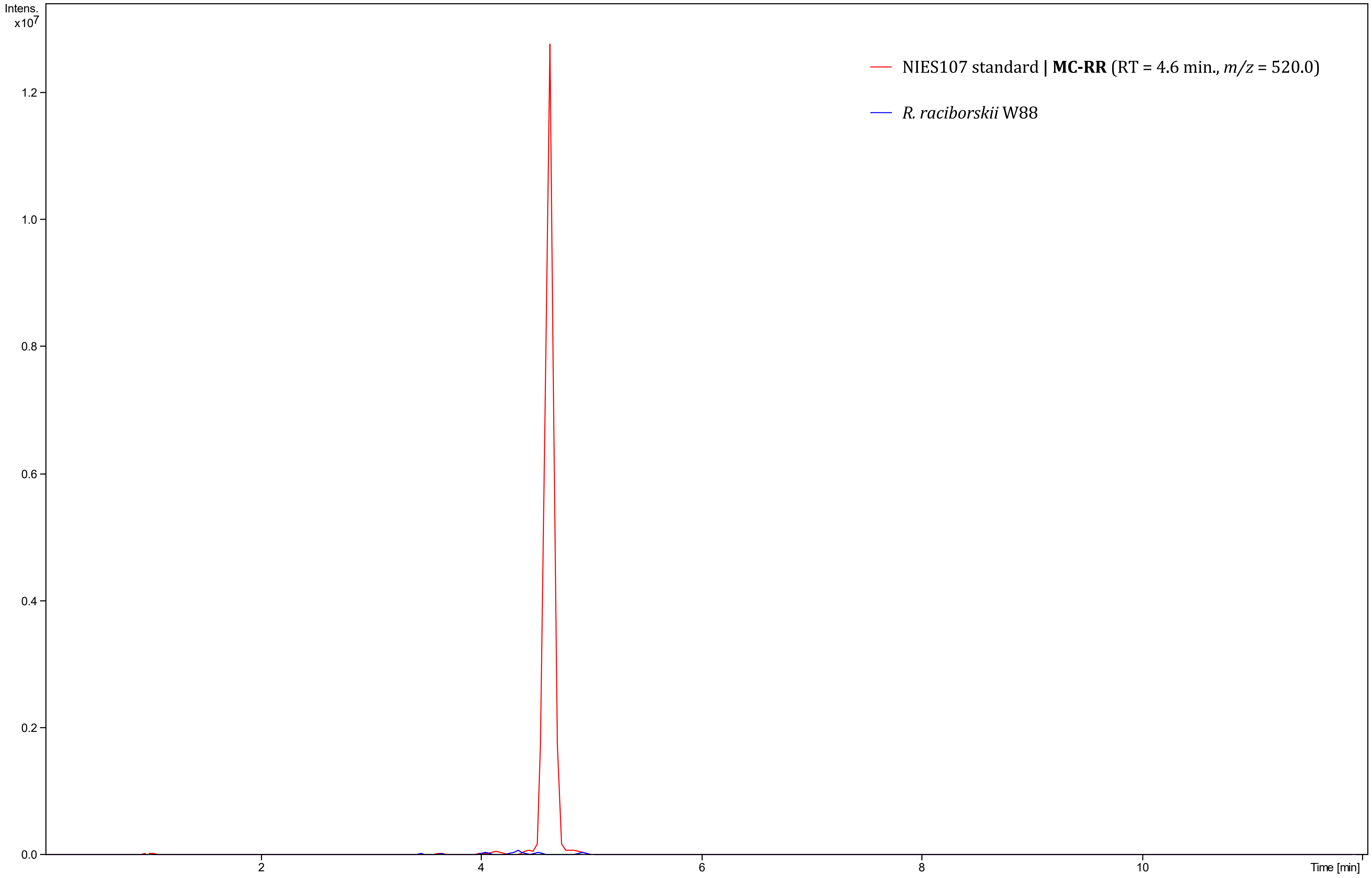

LC-MS analysis | extracted ion chromatogram (*m/z* 1045.6) of NIES107 standard and *R. raciborskii* W88

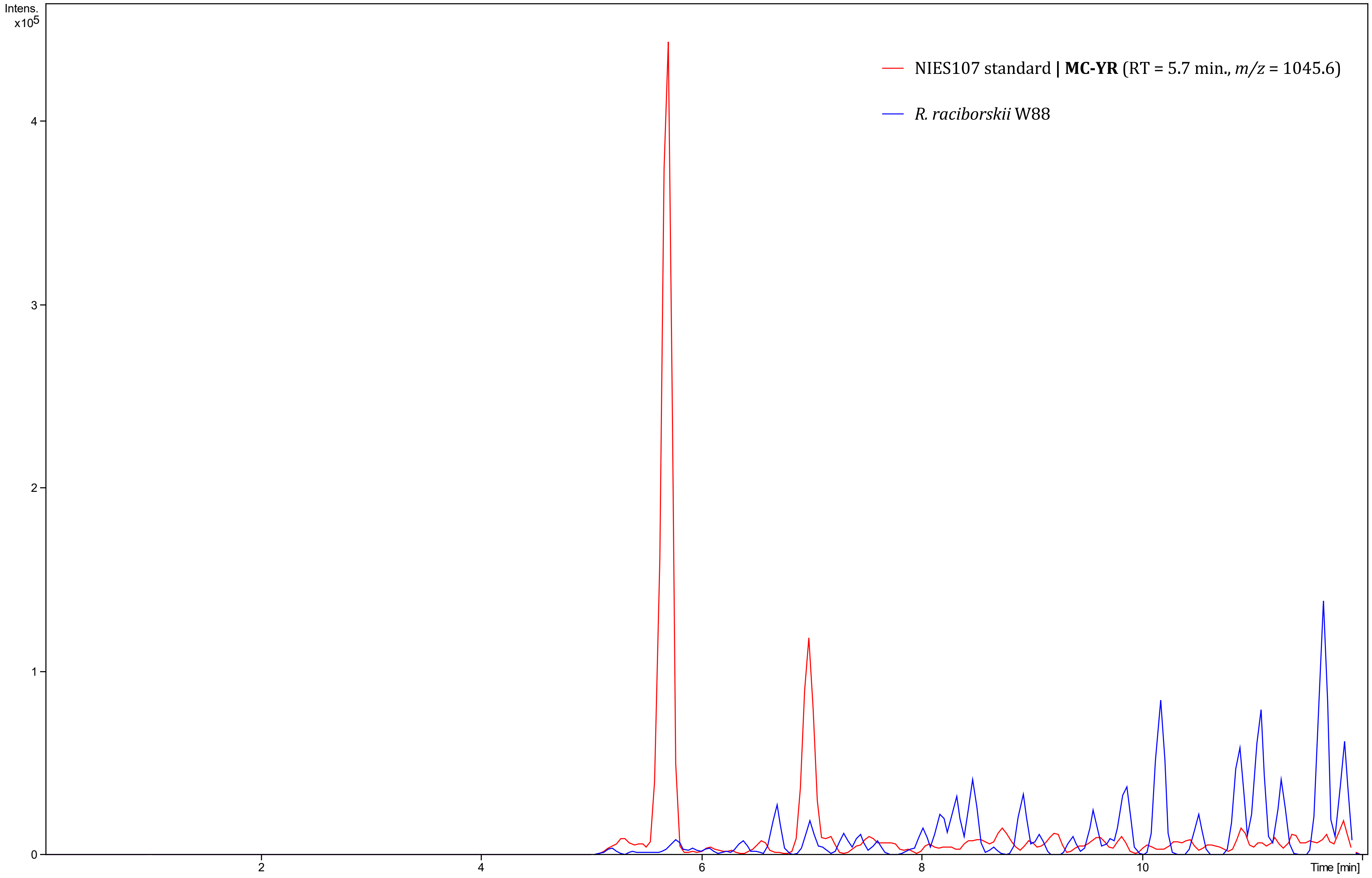

LC-MS analysis | extracted ion chromatogram (*m/z* 995.5) of PCC7820 standard and *R. raciborskii* W88

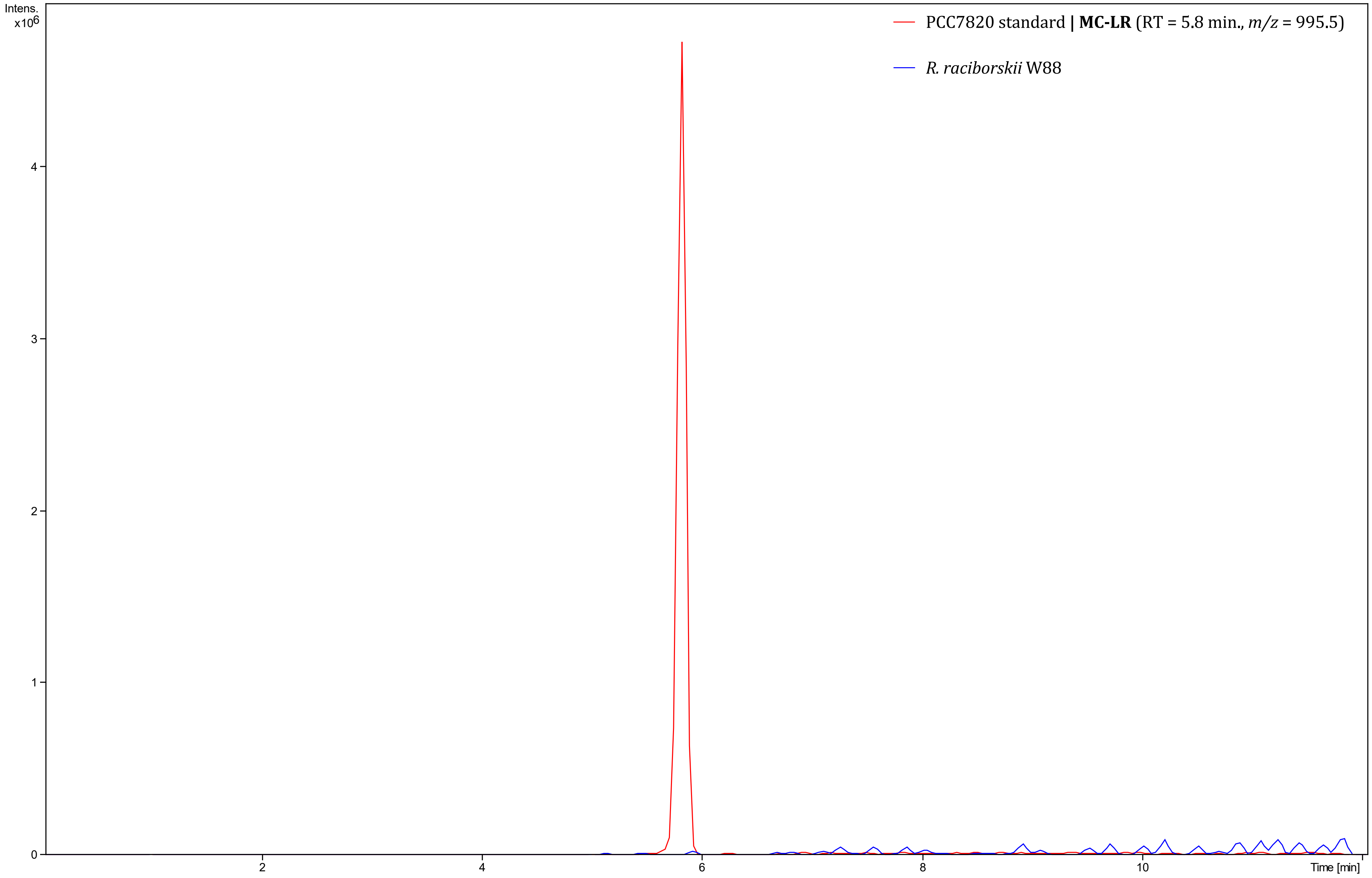

LC-MS analysis | extracted ion chromatogram (*m/z* 1002.5) of PCC7820 standard and *R. raciborskii* W88

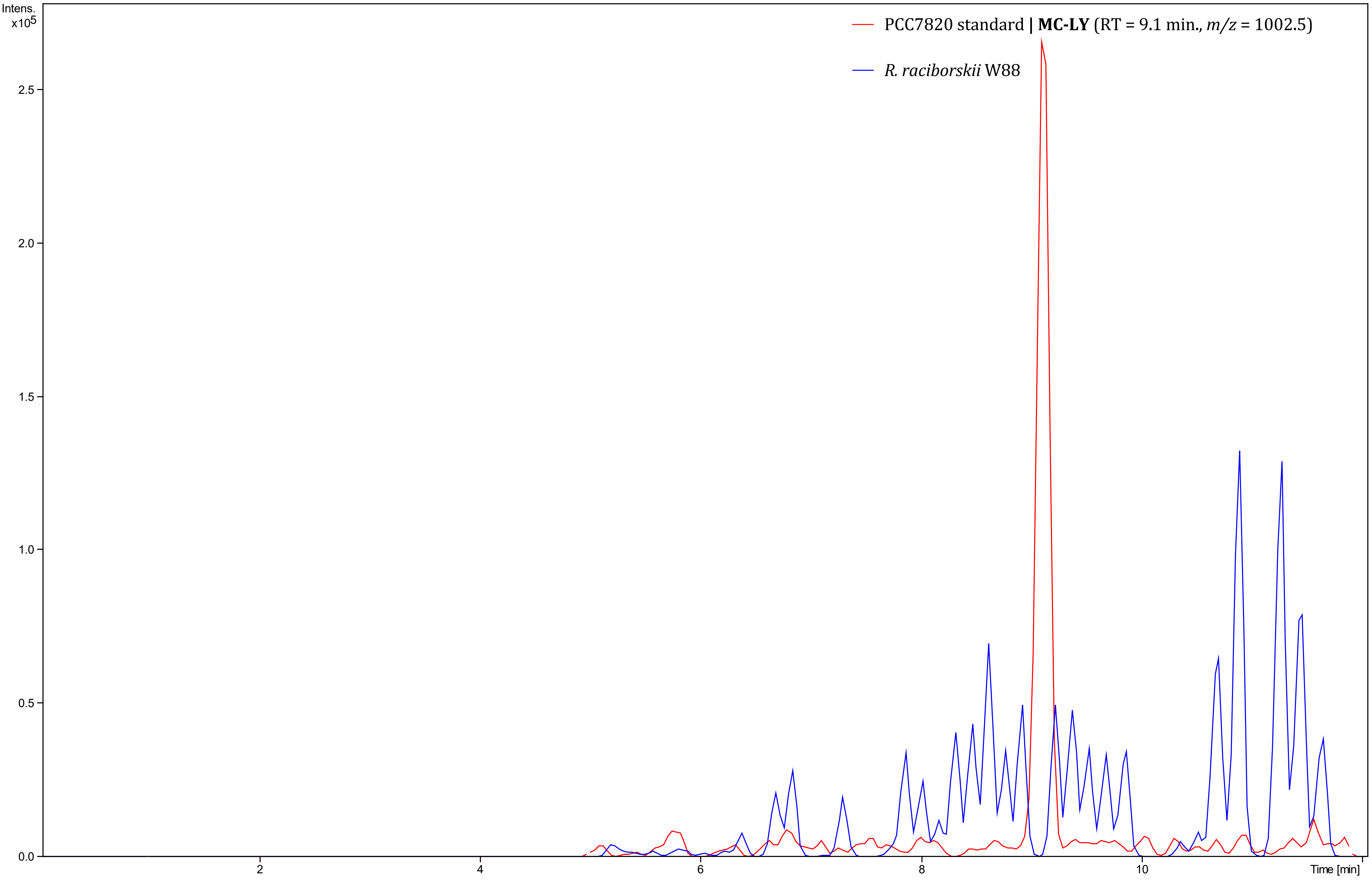

LC-MS analysis | extracted ion chromatogram (*m/z* 1025.9) of PCC7820 standard and *R. raciborskii* W88

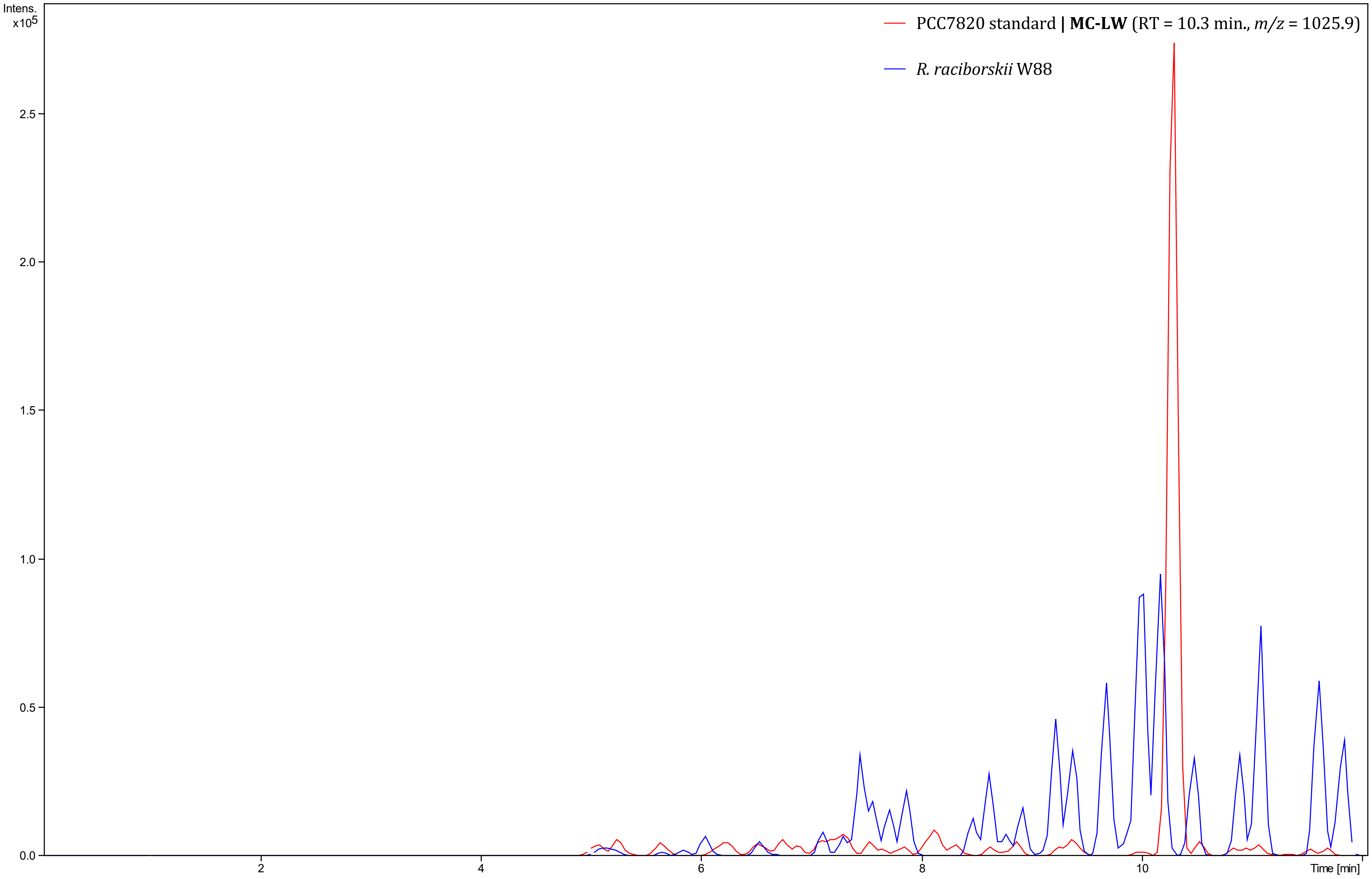

LC-MS analysis | extracted ion chromatogram (*m/z* 986.6) of PCC7820 standard and *R. raciborskii* W88

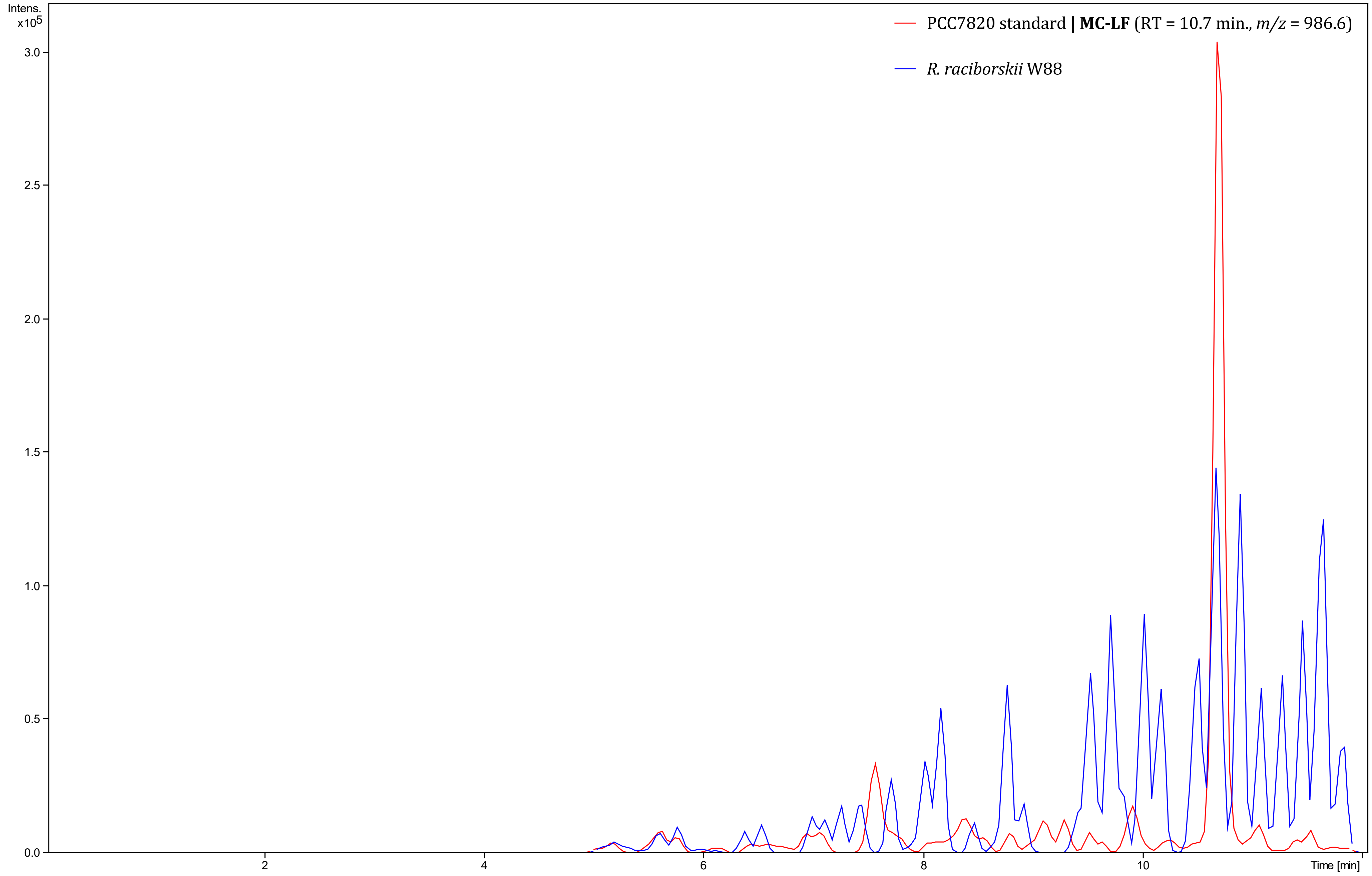

***R. raciborskii* W73**

LC-MS analysis | extracted ion chromatogram (*m/z* 513.0) of NIES107 standard and *R. raciborskii* W73

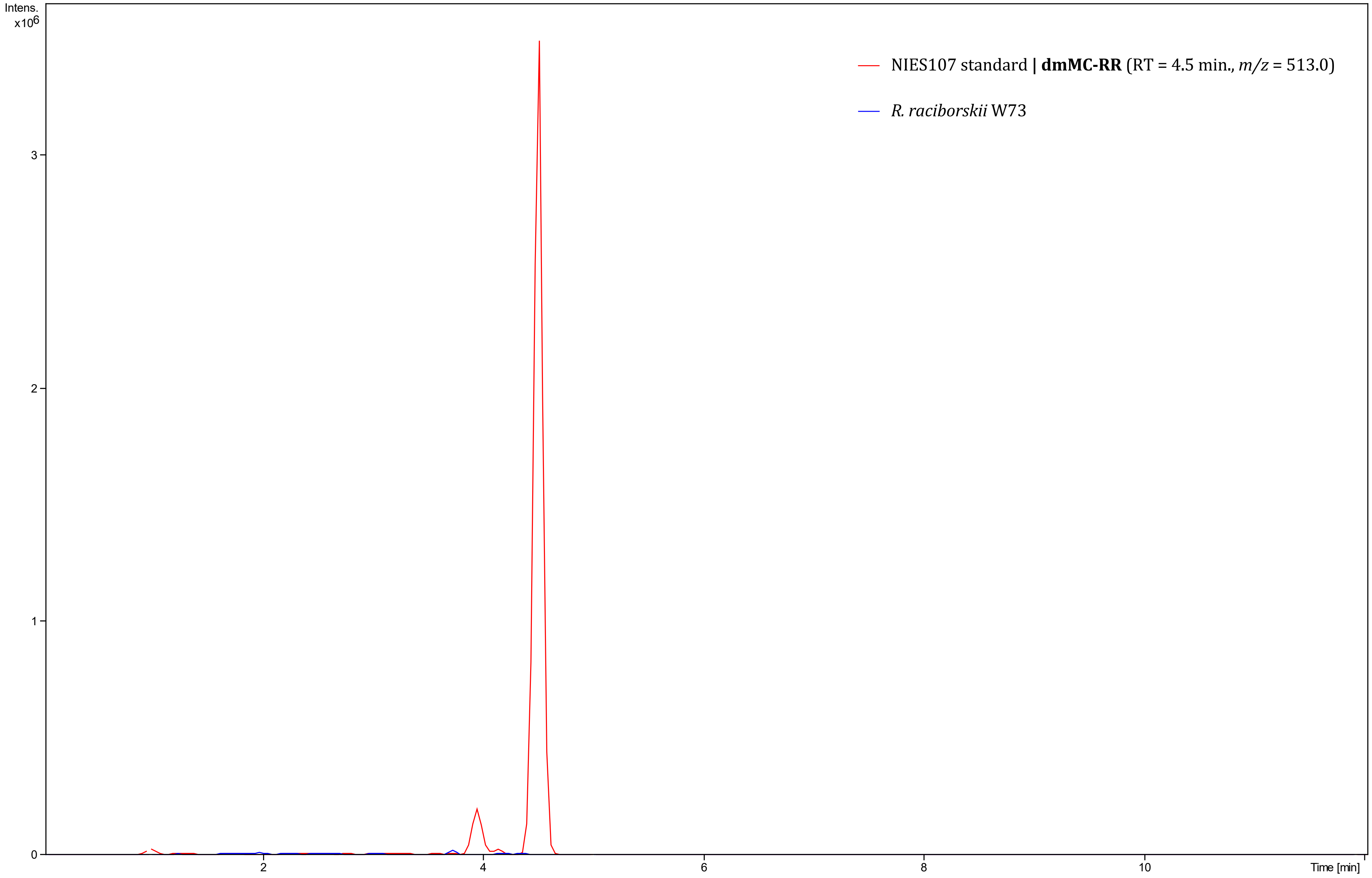

LC-MS analysis | extracted ion chromatogram (*m/z* 520.0) of NIES107 standard and *R. raciborskii* W73

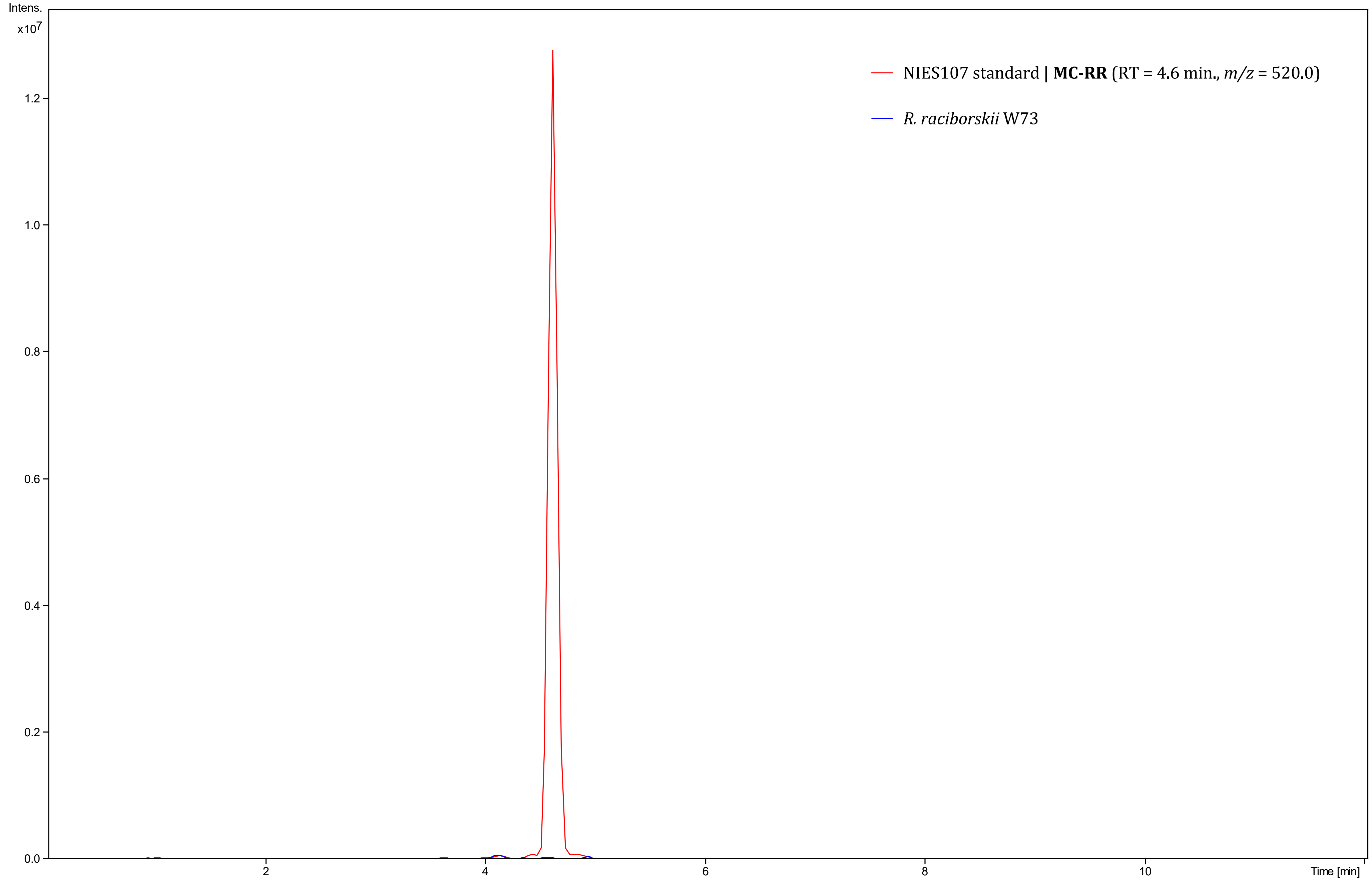

LC-MS analysis | extracted ion chromatogram (*m/z* 1045.6) of NIES107 standard and *R. raciborskii* W73

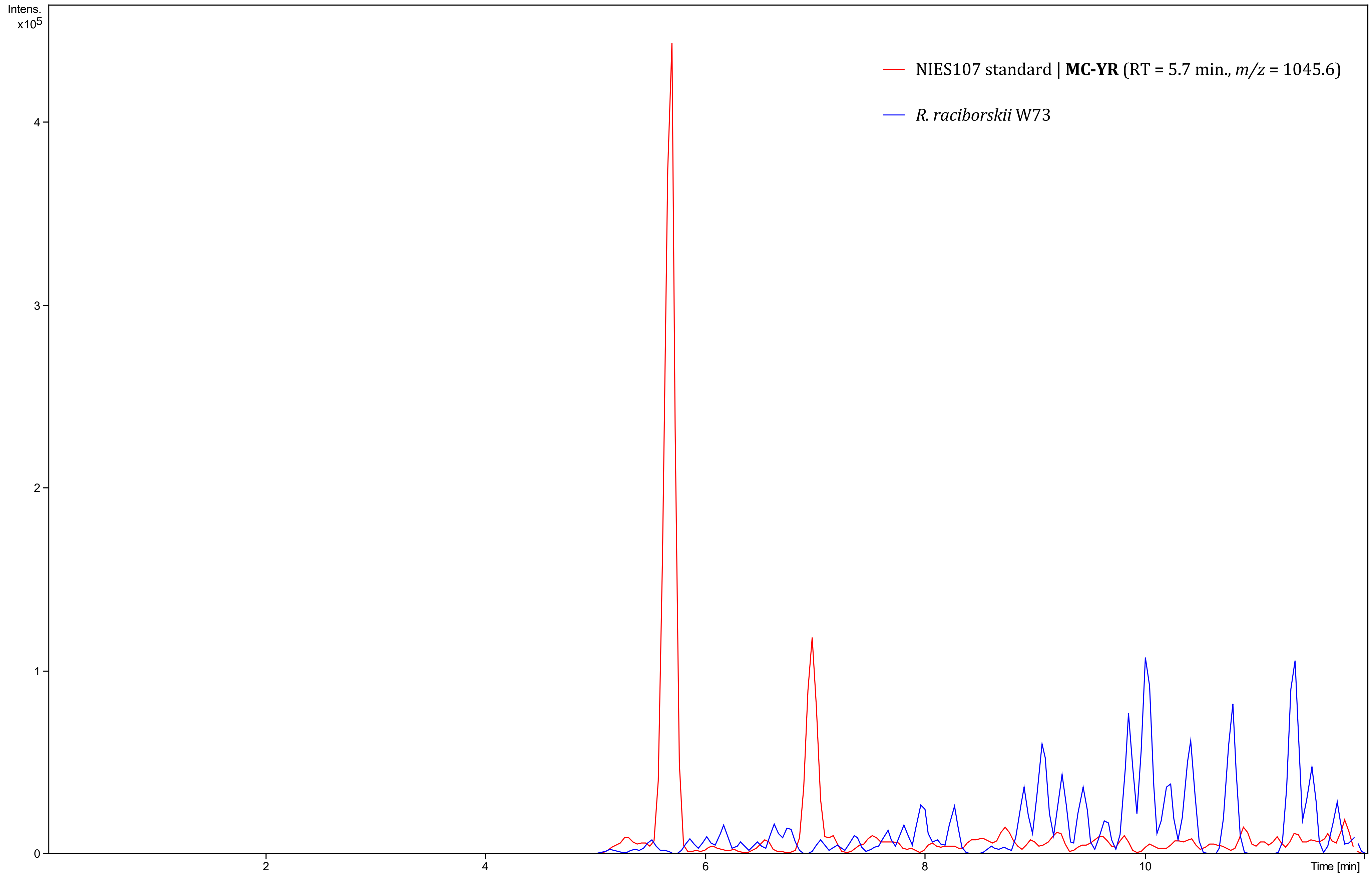

LC-MS analysis | extracted ion chromatogram (m/z 995.5) of PCC7820 standard and *R. raciborskii* W73

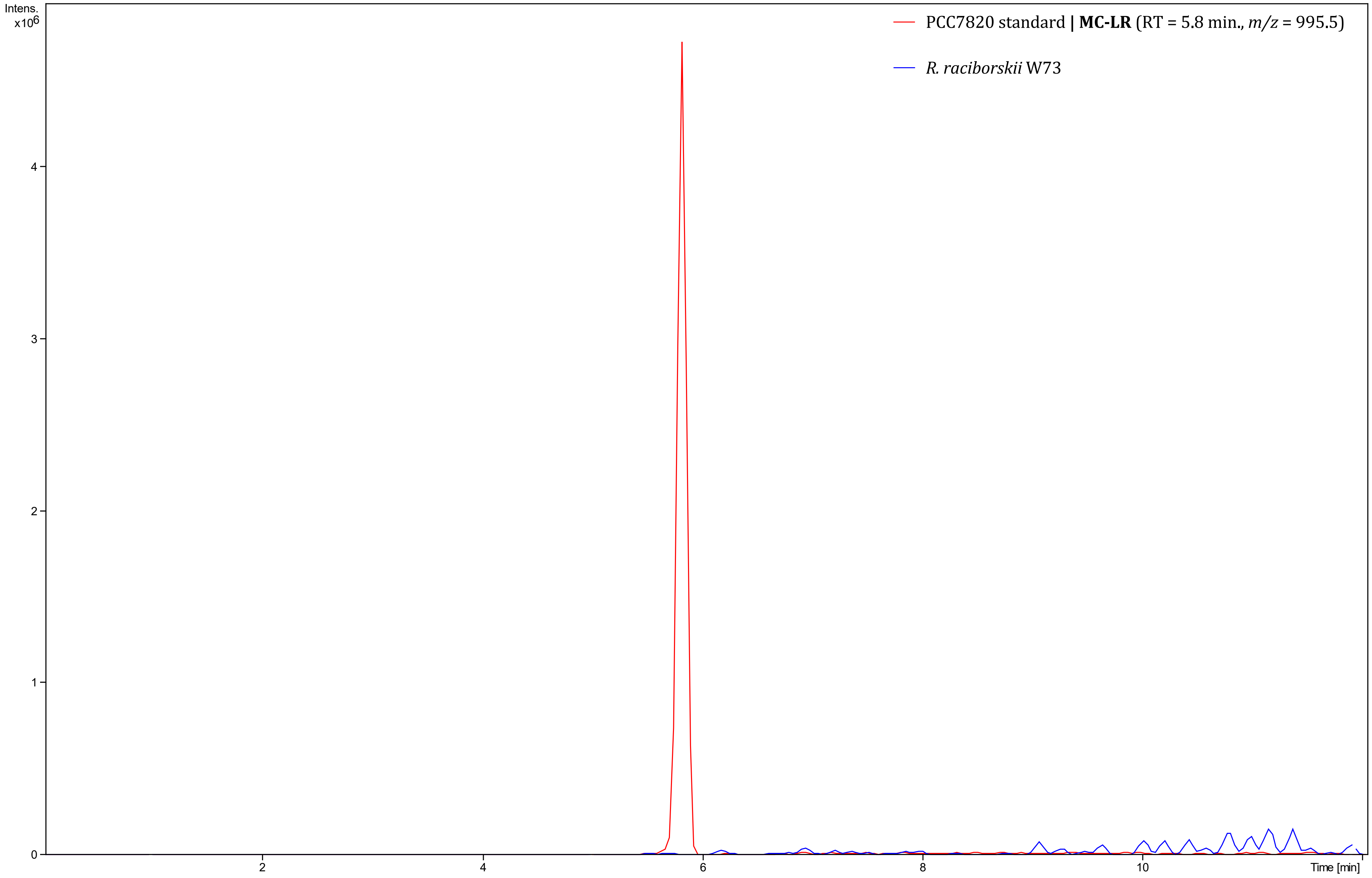

LC-MS analysis | extracted ion chromatogram (*m/z* 1002.5) of PCC7820 standard and *R. raciborskii* W73

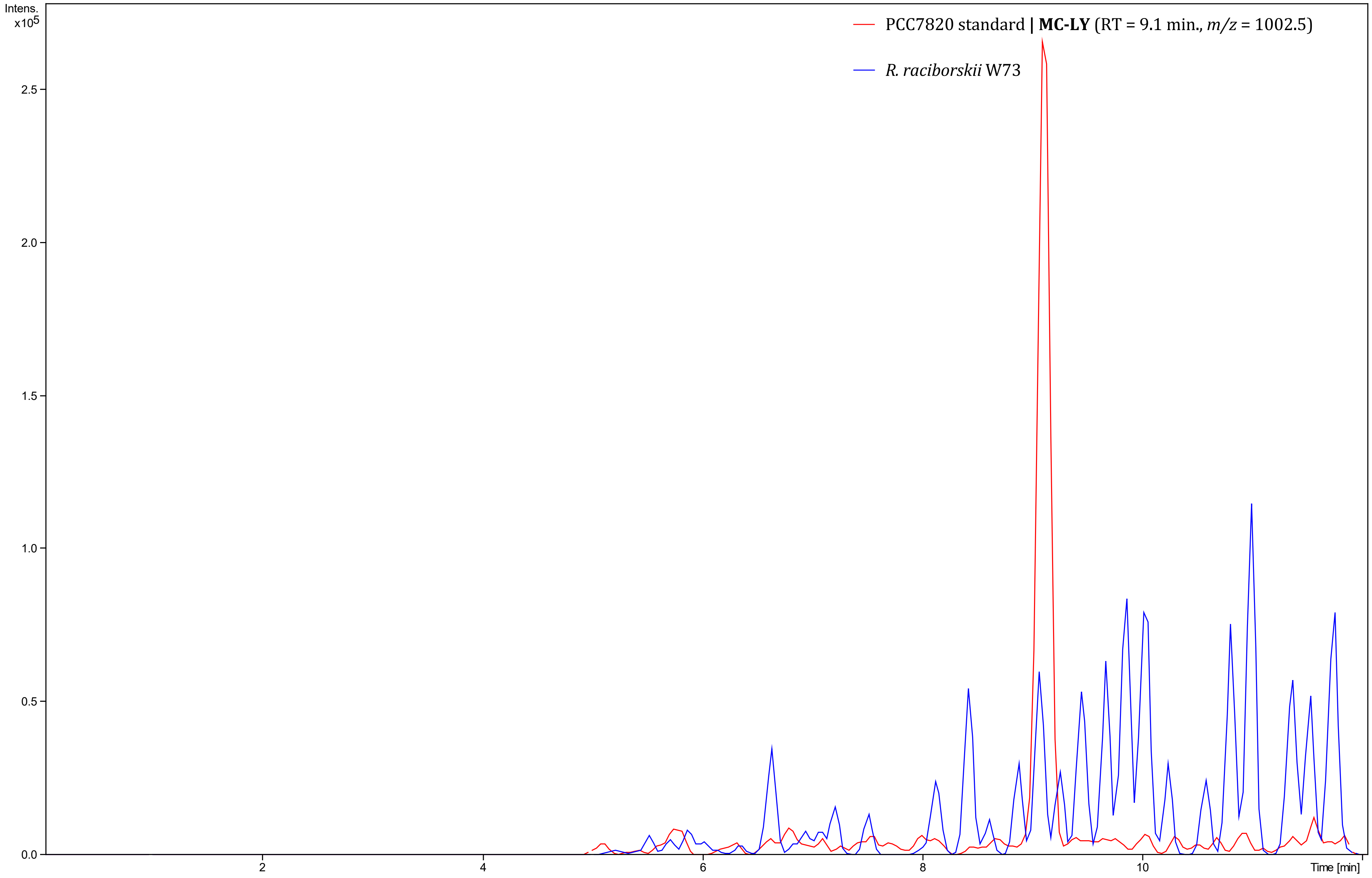

LC-MS analysis | extracted ion chromatogram (*m/z* 1025.9) of PCC7820 standard and *R. raciborskii* W73

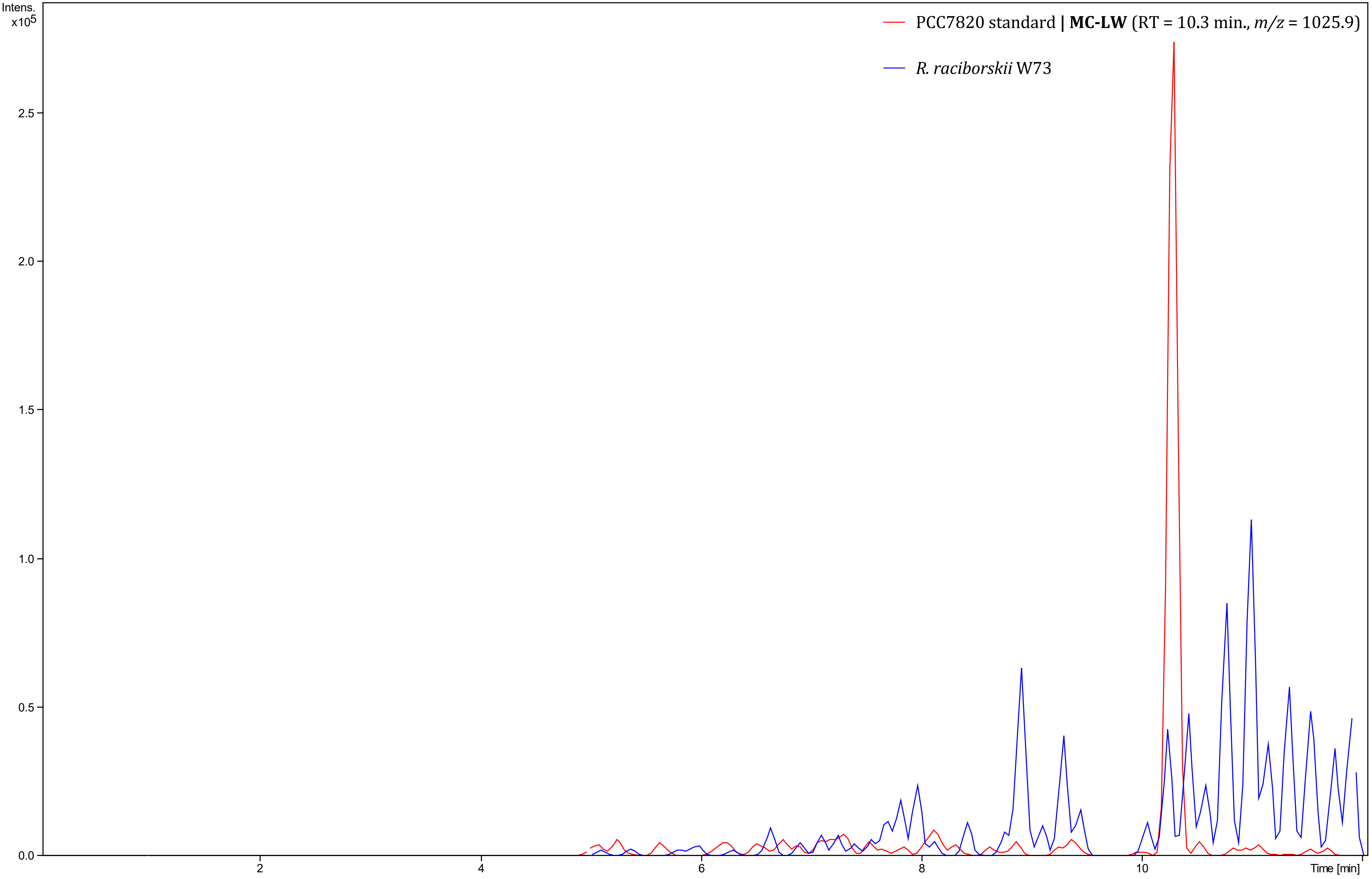

LC-MS analysis | extracted ion chromatogram (*m/z* 986.6) of PCC7820 standard and *R. raciborskii* W73

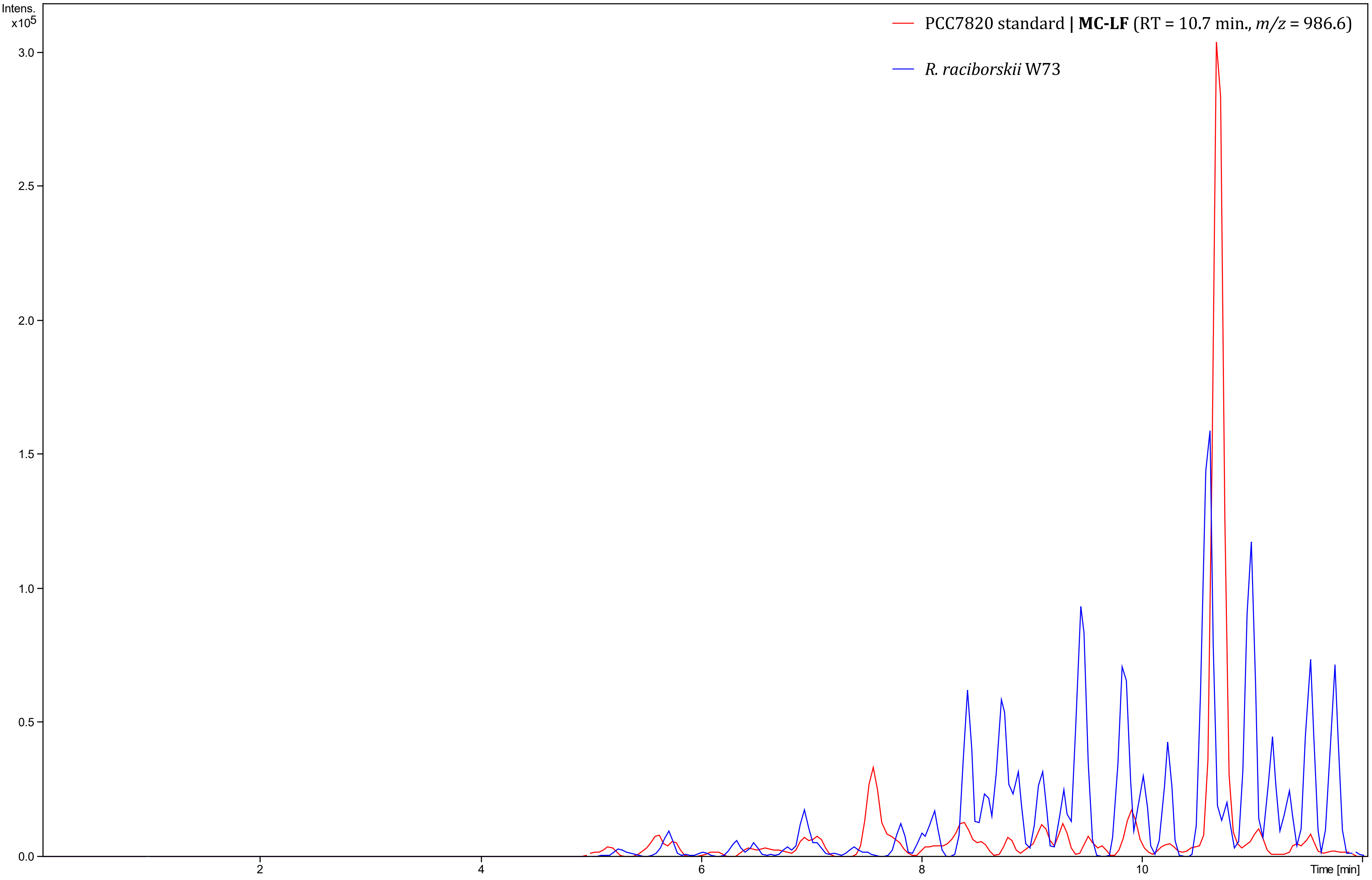

***A. gracile* W71**

LC-MS analysis | extracted ion chromatogram (*m/z* 513.0) of NIES107 standard and *A. gracile* W71

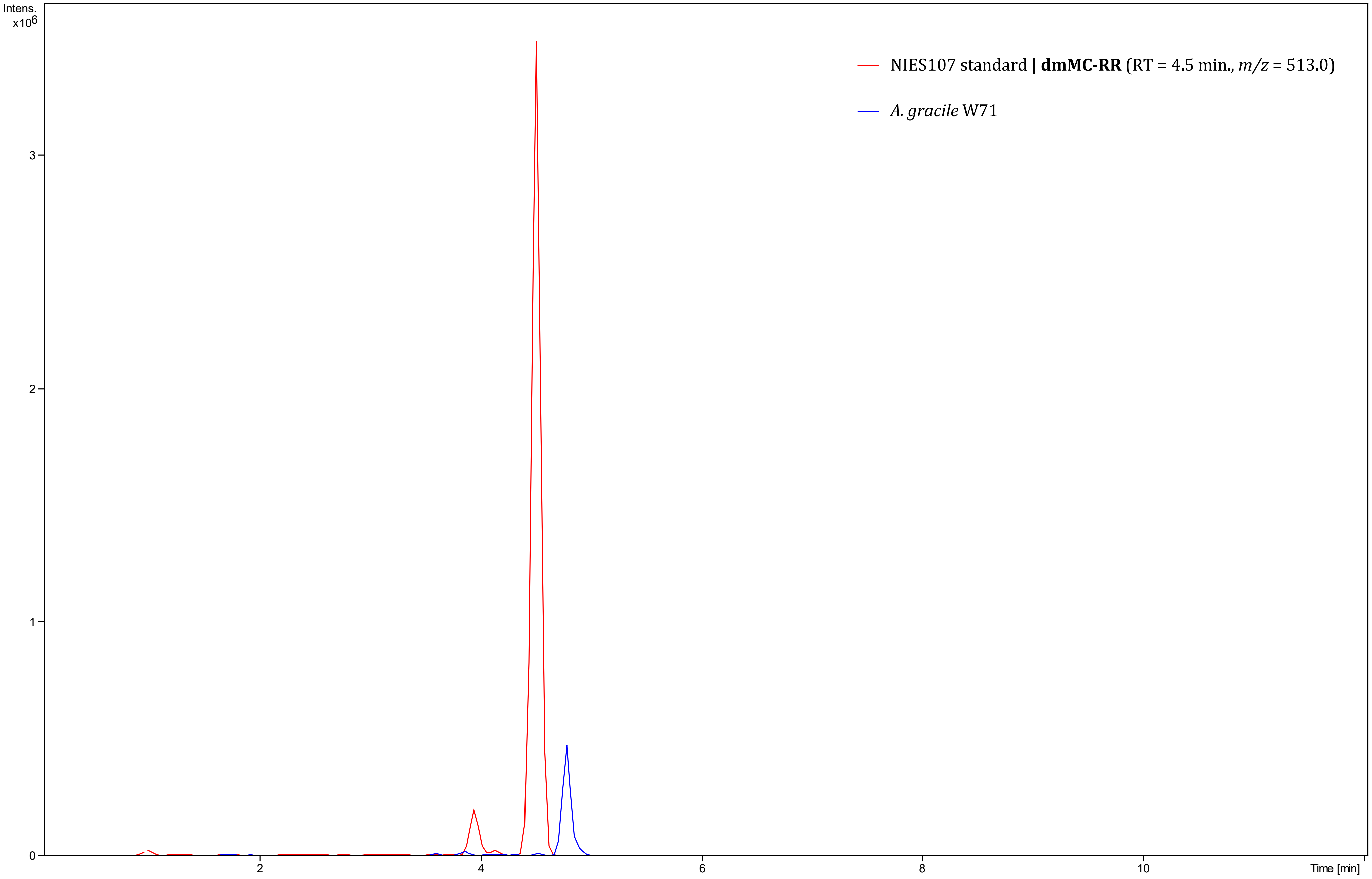

LC-MS analysis | extracted ion chromatogram (*m/z* 520.0) of NIES107 standard and *A. gracile* W71

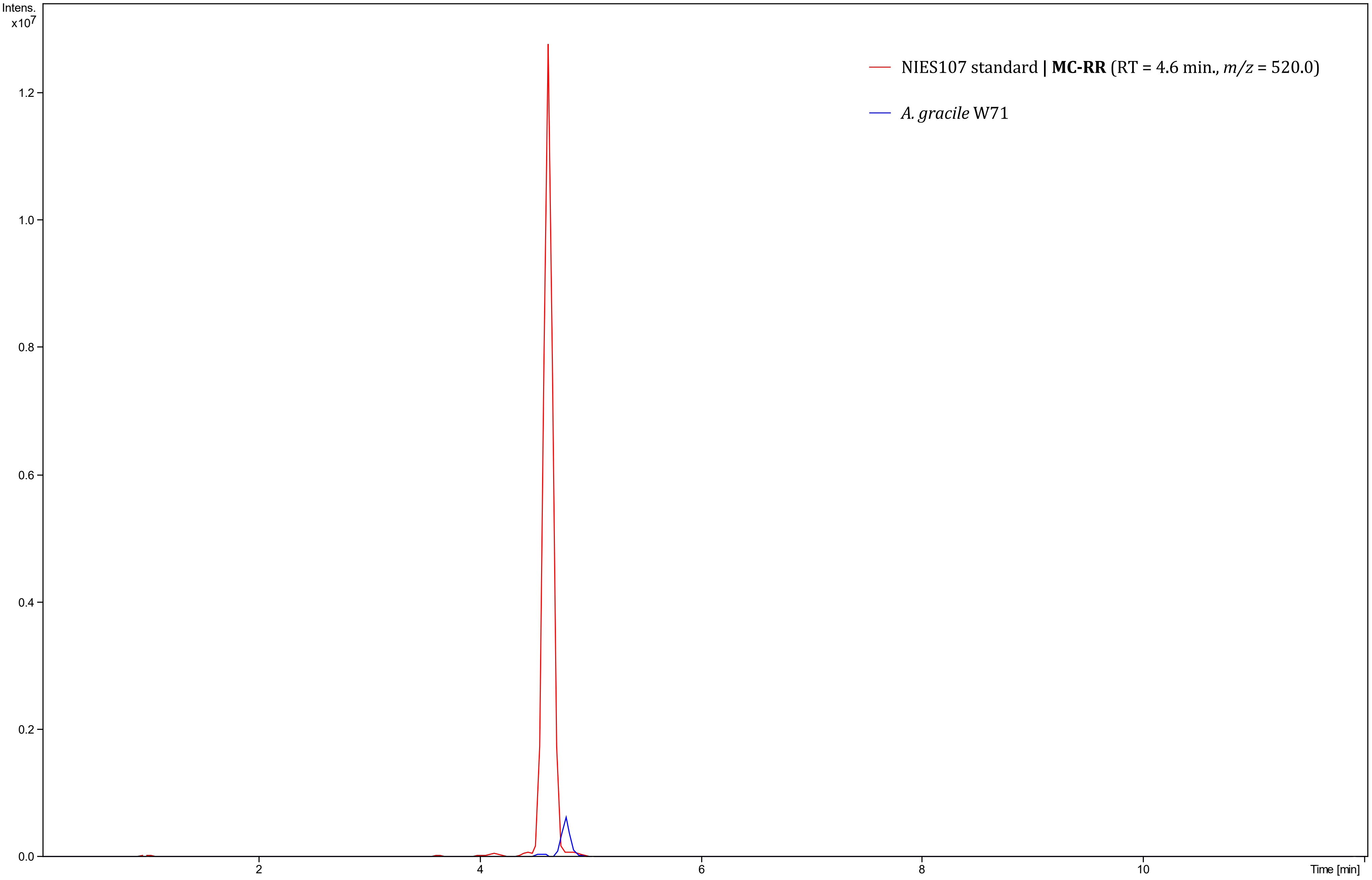

LC-MS analysis | extracted ion chromatogram (*m/z* 1045.6) of NIES107 standard and *A. gracile* W71

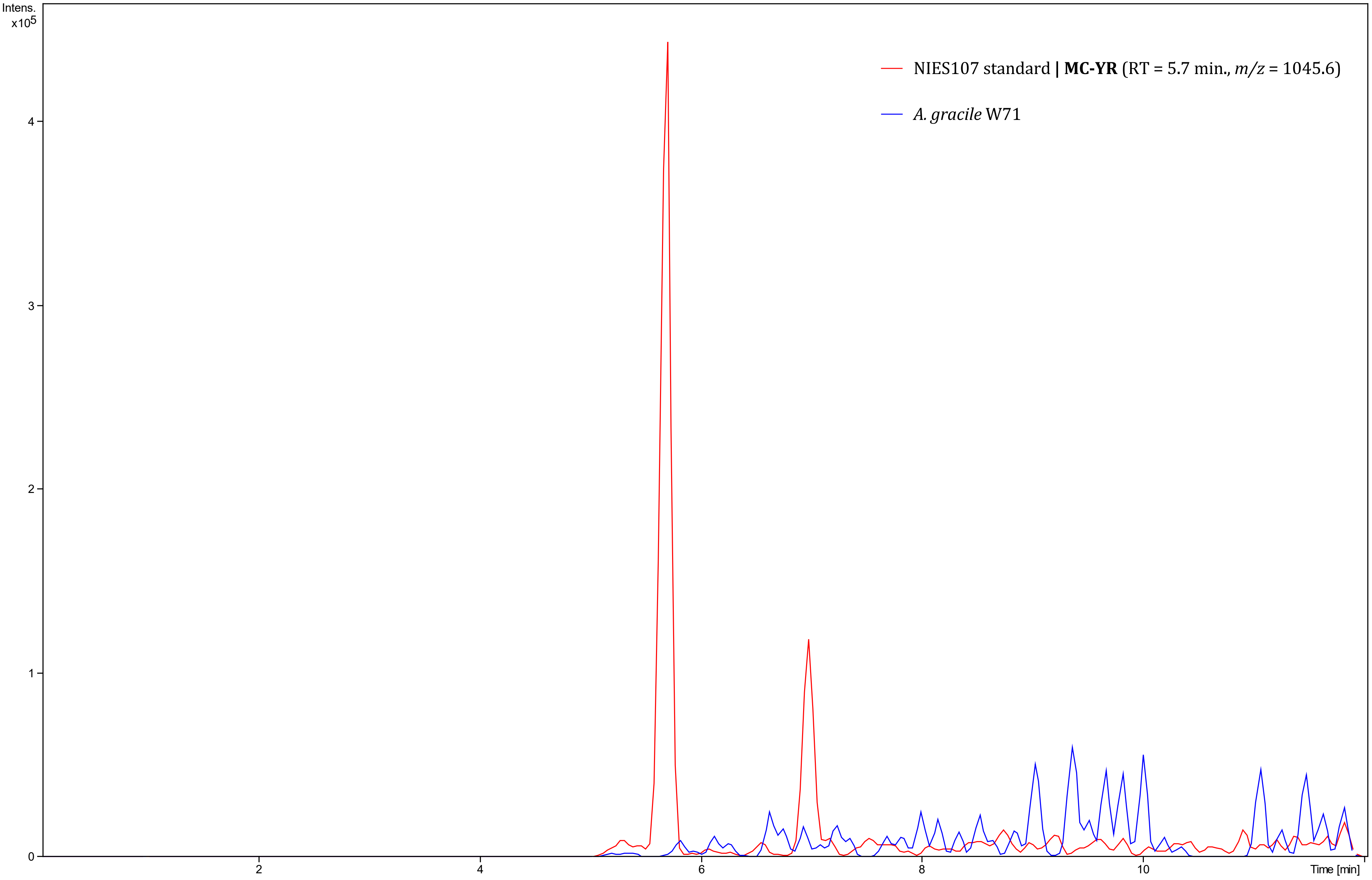

LC-MS analysis | extracted ion chromatogram (*m/z* 995.5) of PCC7820 standard and *A. gracile* W71

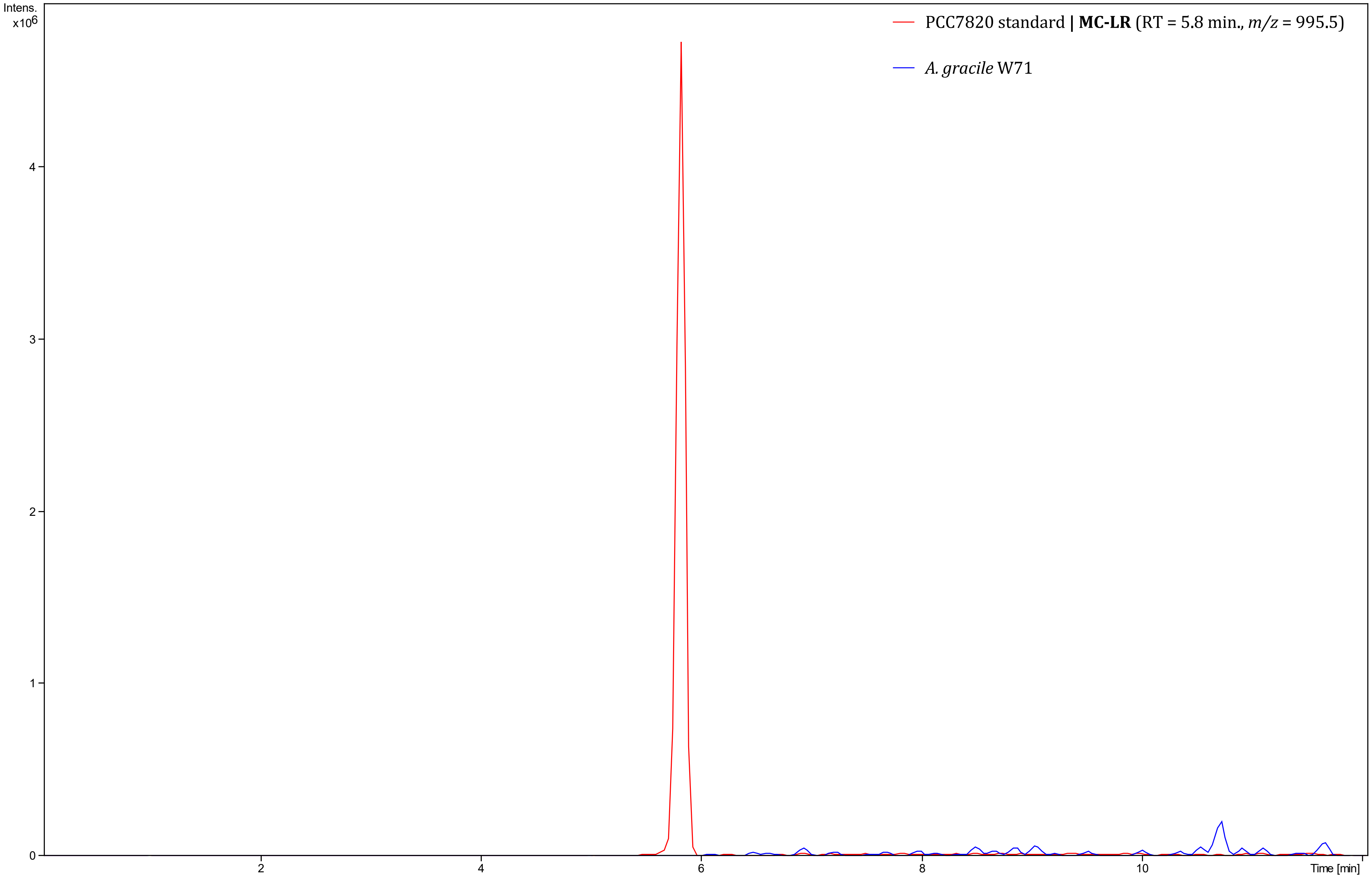

LC-MS analysis | extracted ion chromatogram (*m/z* 1002.5) of PCC7820 standard and *A. gracile* W71

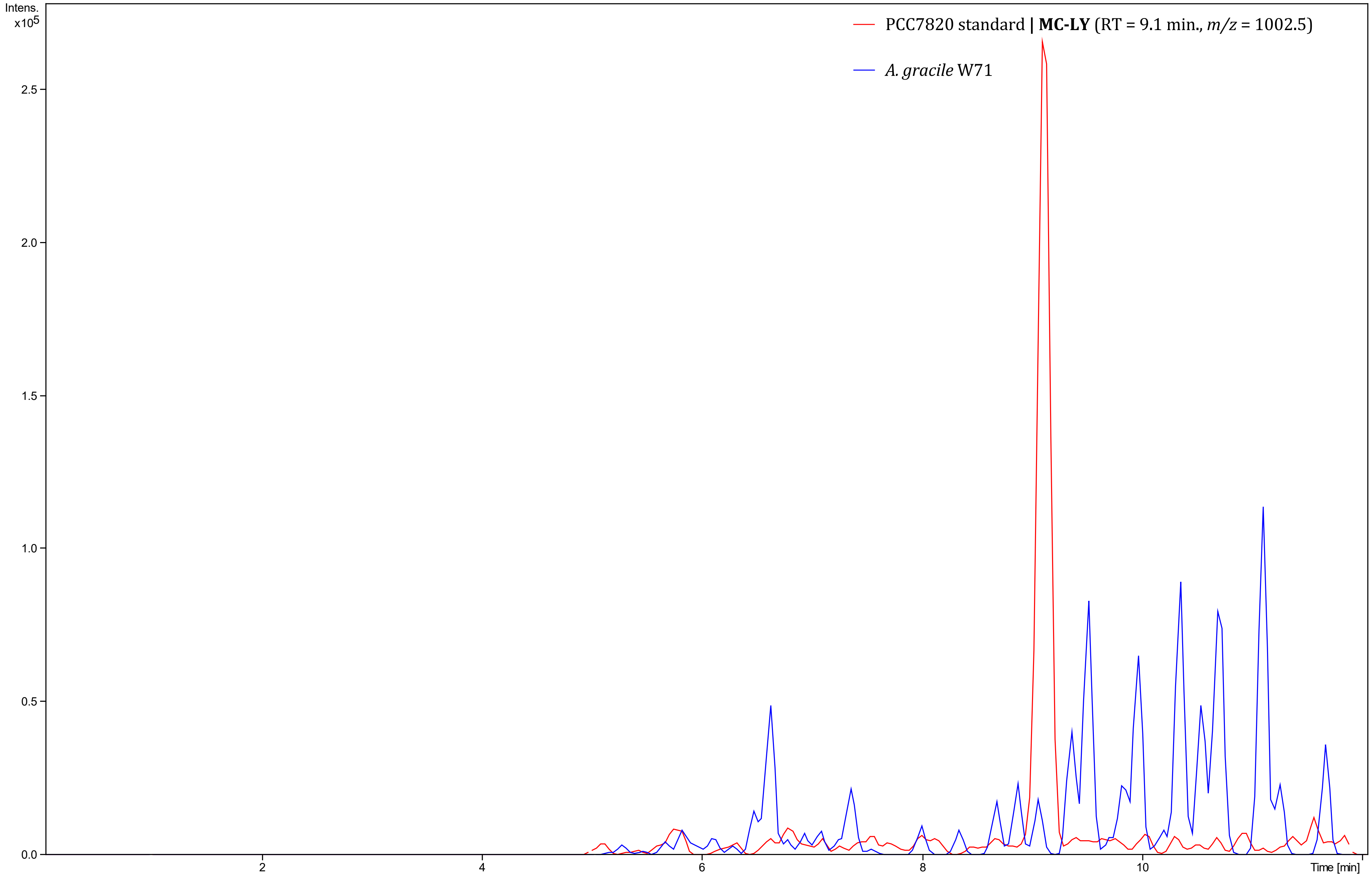

LC-MS analysis | extracted ion chromatogram (*m/z* 1025.9) of PCC7820 standard and *A. gracile* W71

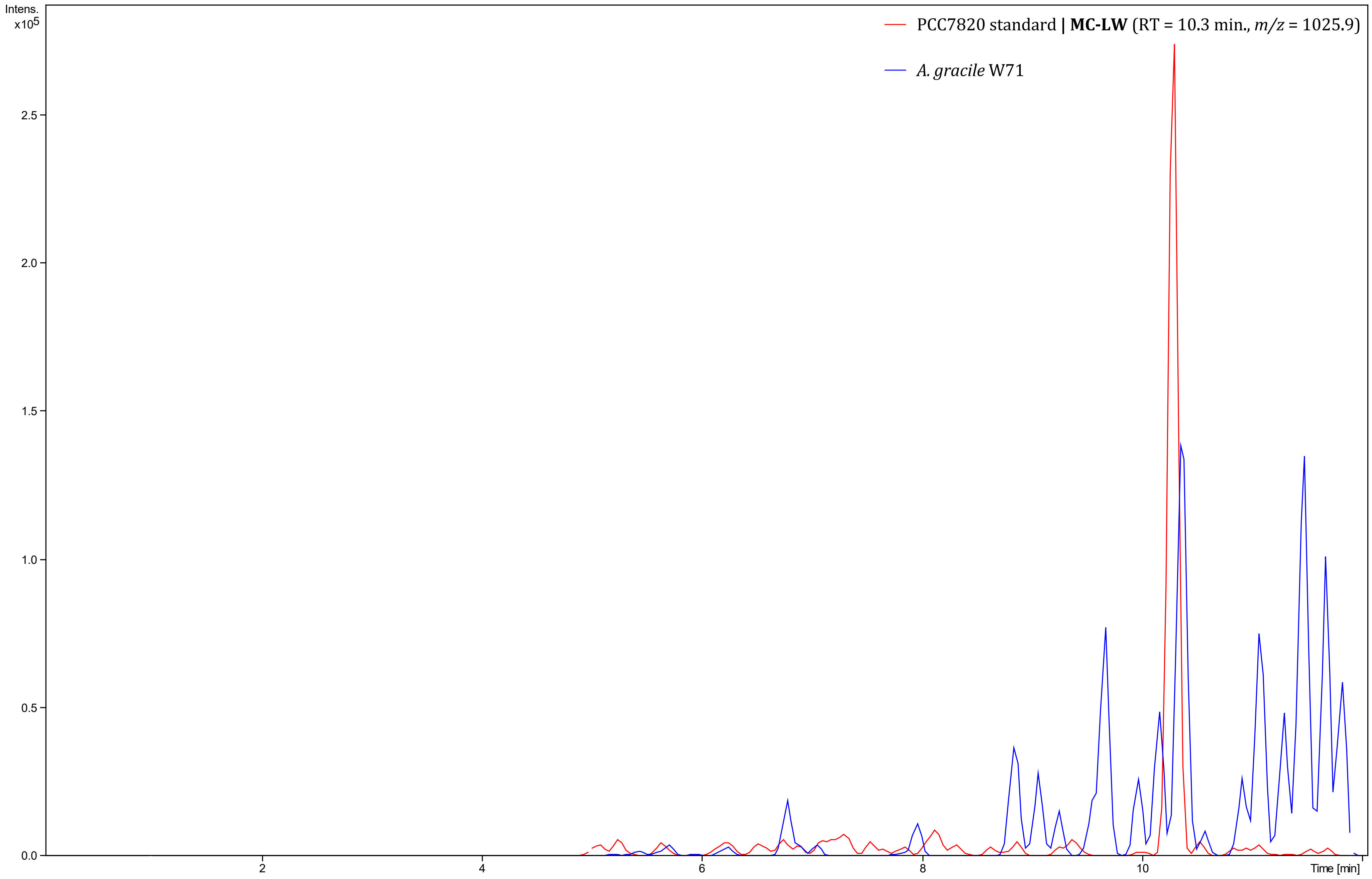

LC-MS analysis | extracted ion chromatogram (*m/z* 986.6) of PCC7820 standard and *A. gracile* W71

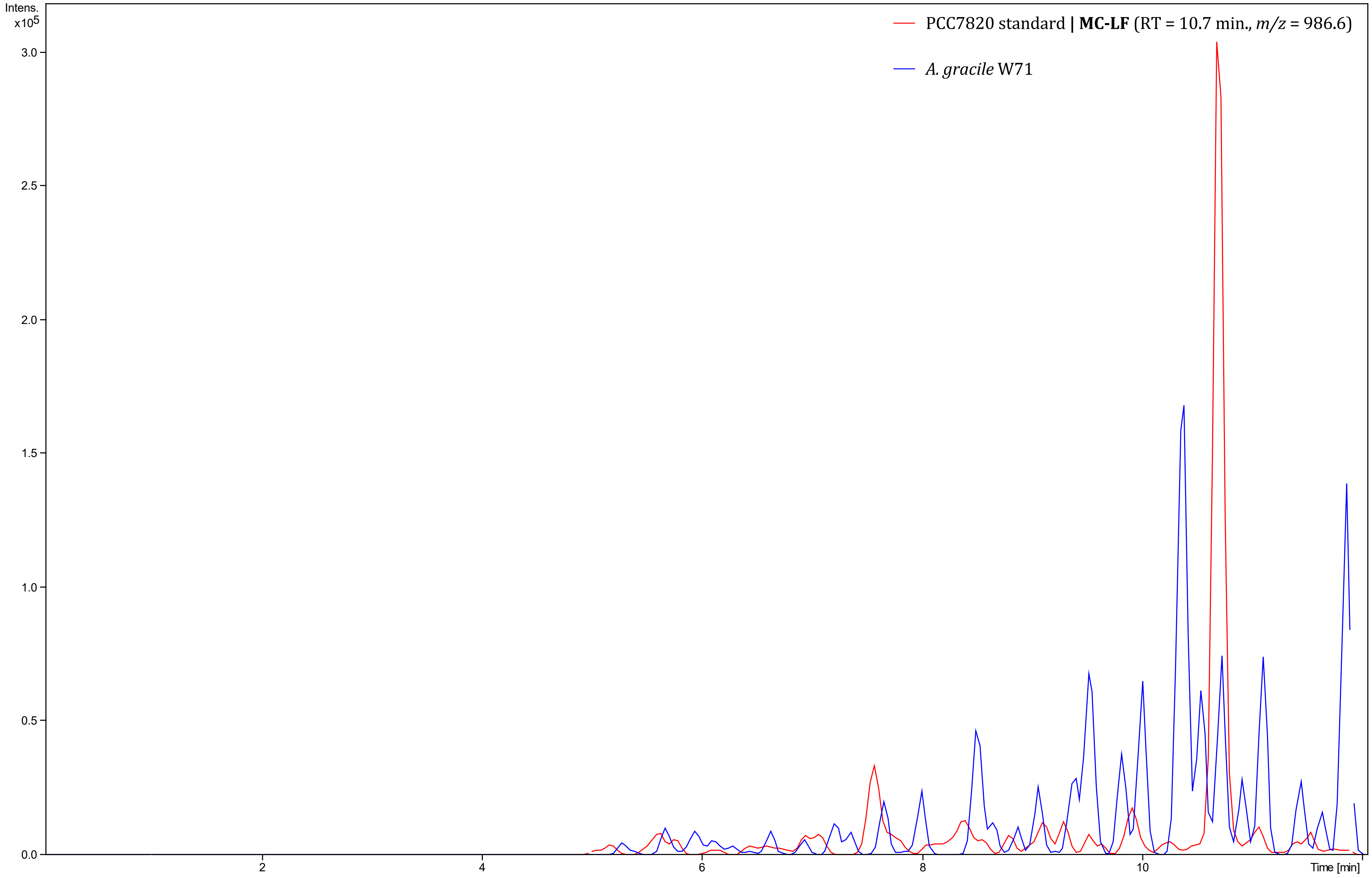

***A. gracile* W4**

LC-MS analysis | extracted ion chromatogram (*m/z* 513.0) of NIES107 standard and *A. gracile* W4

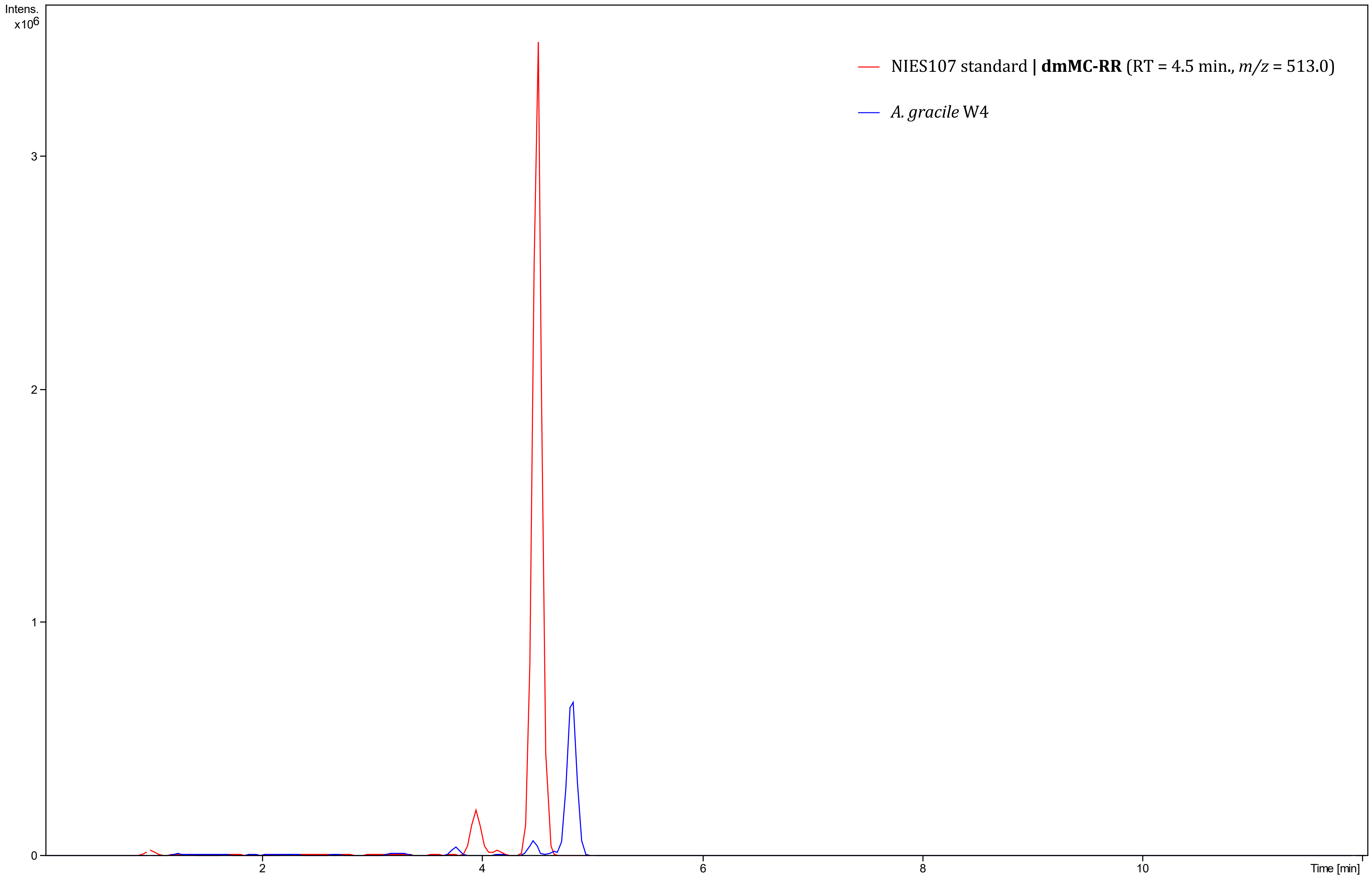

LC-MS analysis | extracted ion chromatogram (*m/z* 520.0) of NIES107 standard and *A. gracile* W4

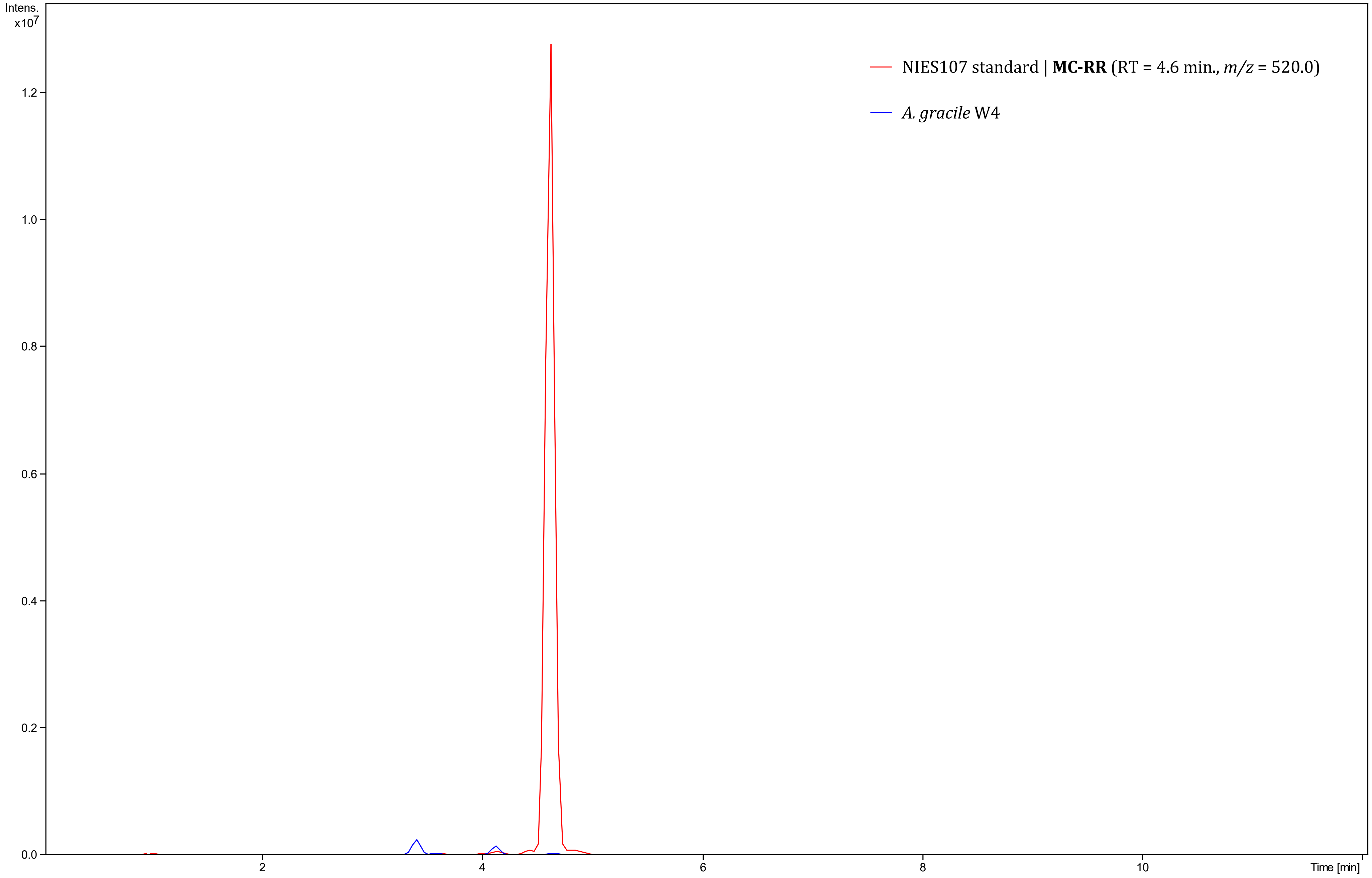

LC-MS analysis | extracted ion chromatogram (*m/z* 1045.6) of NIES107 standard and *A. gracile* W4

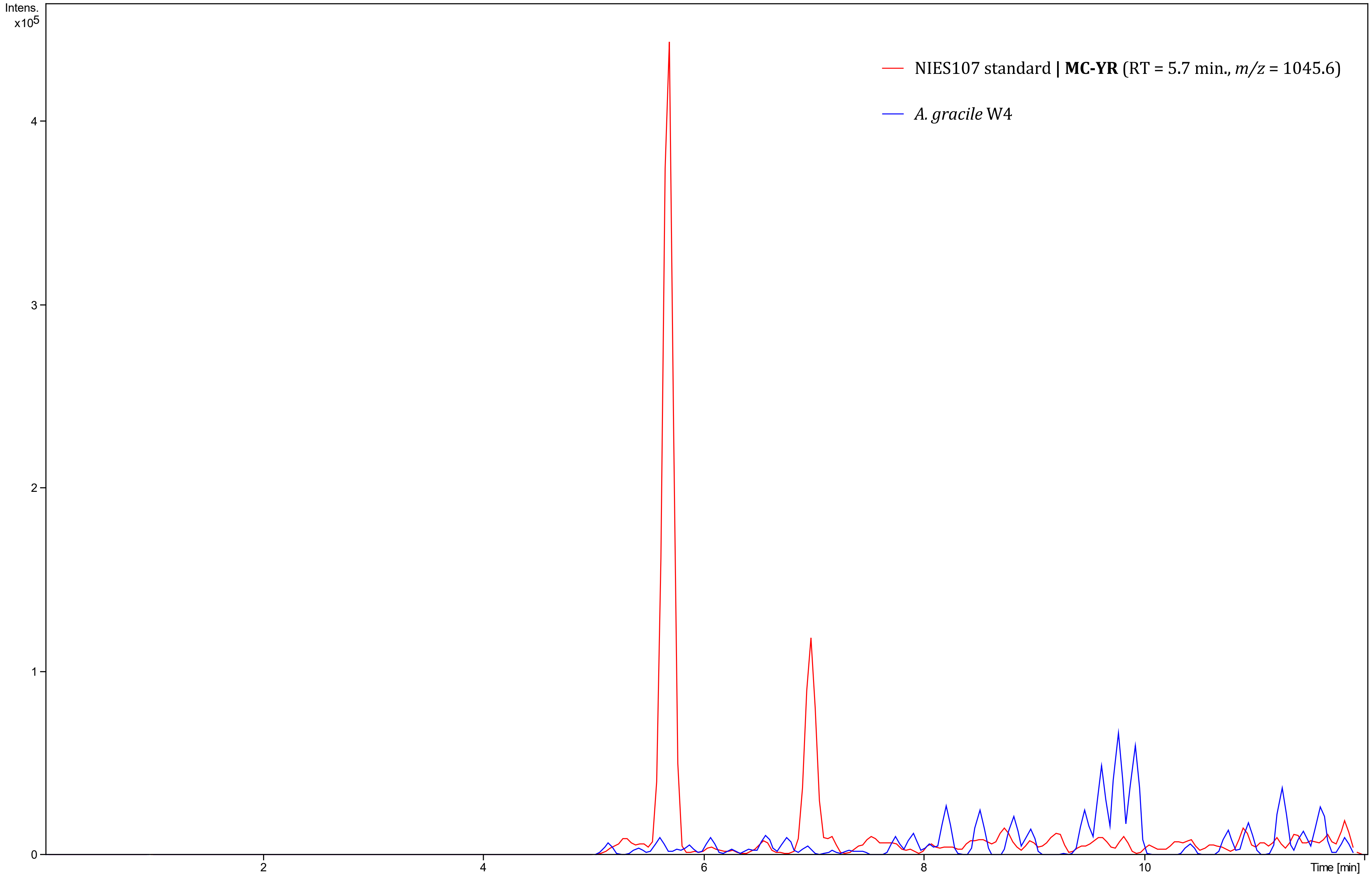

LC-MS analysis | extracted ion chromatogram (*m/z* 995.5) of PCC7820 standard and *A. gracile* W4

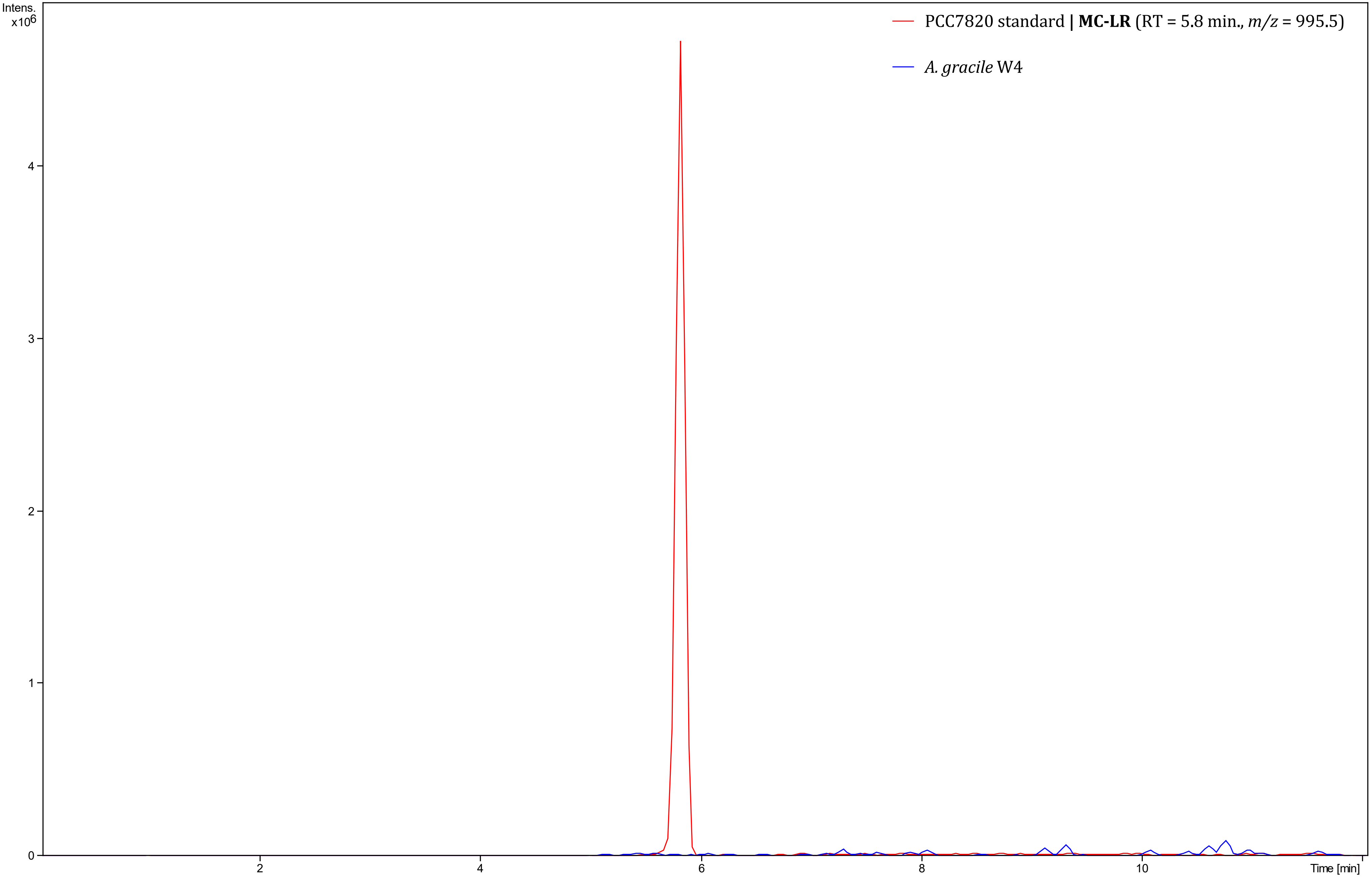

LC-MS analysis | extracted ion chromatogram (m/z 1002.5) of PCC7820 standard and *A. gracile* W4

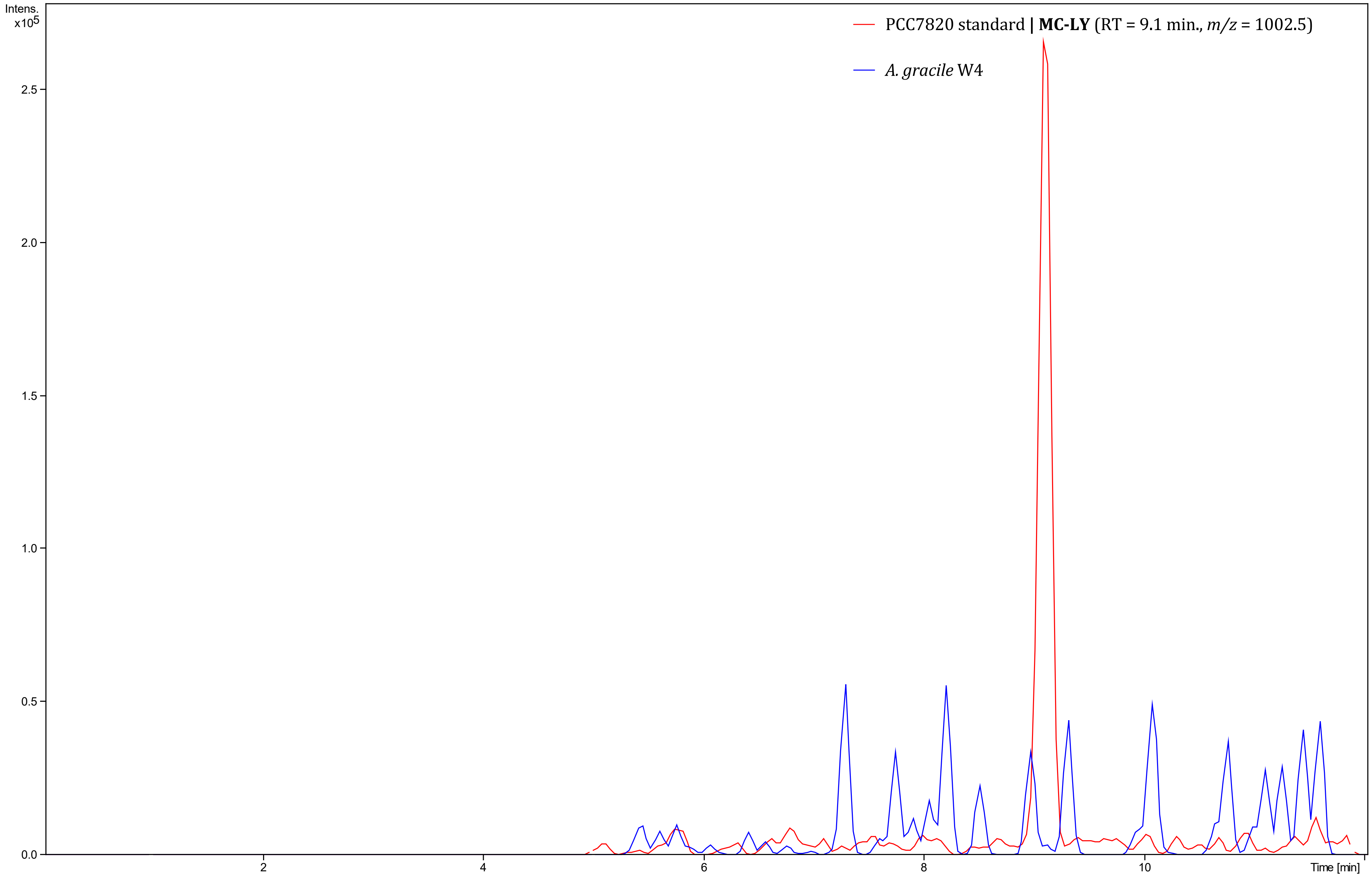

LC-MS analysis | extracted ion chromatogram (*m/z* 1025.9) of PCC7820 standard and *A. gracile* W4

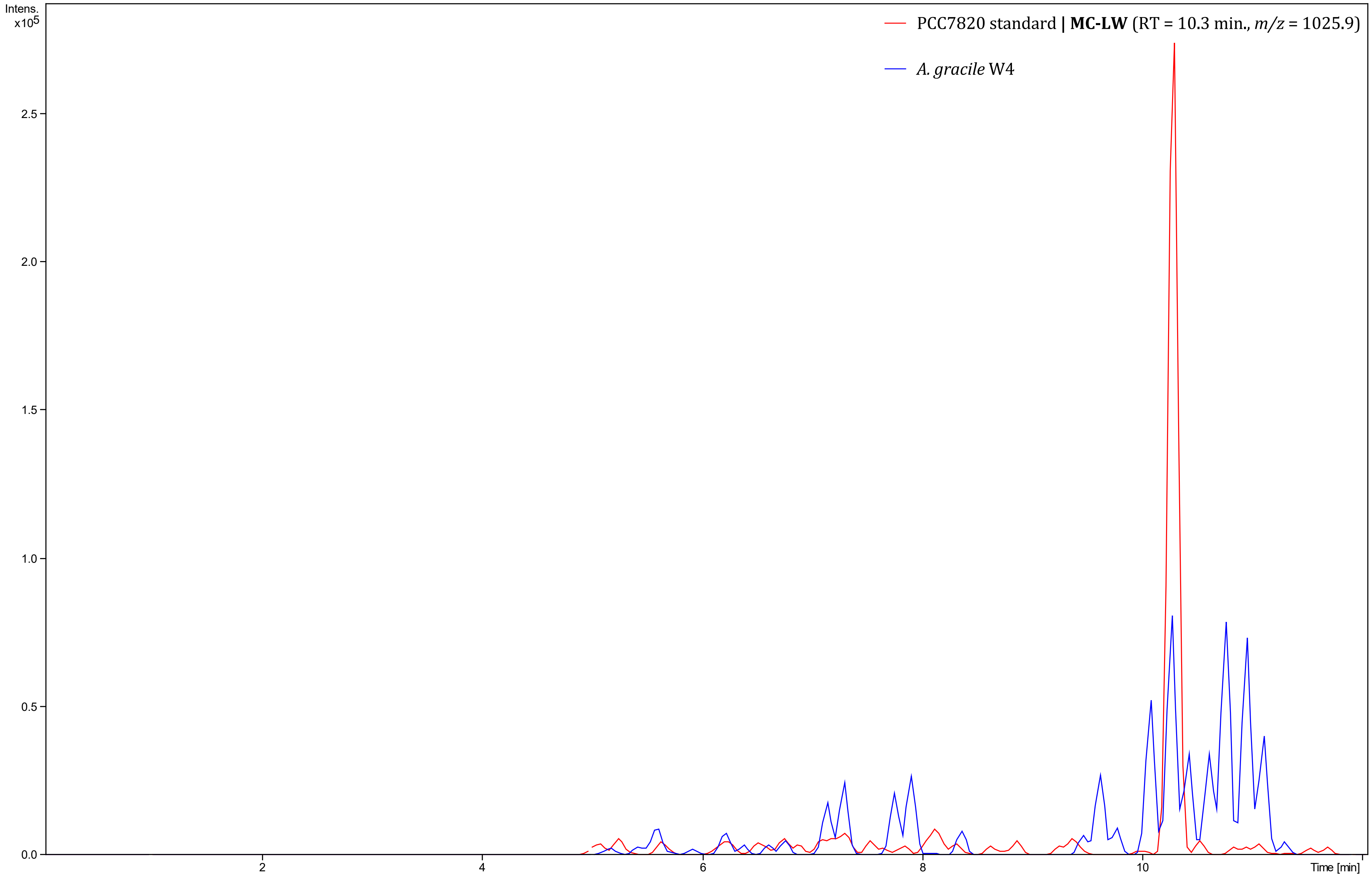

LC-MS analysis | extracted ion chromatogram (*m/z* 986.6) of PCC7820 standard and *A. gracile* W4

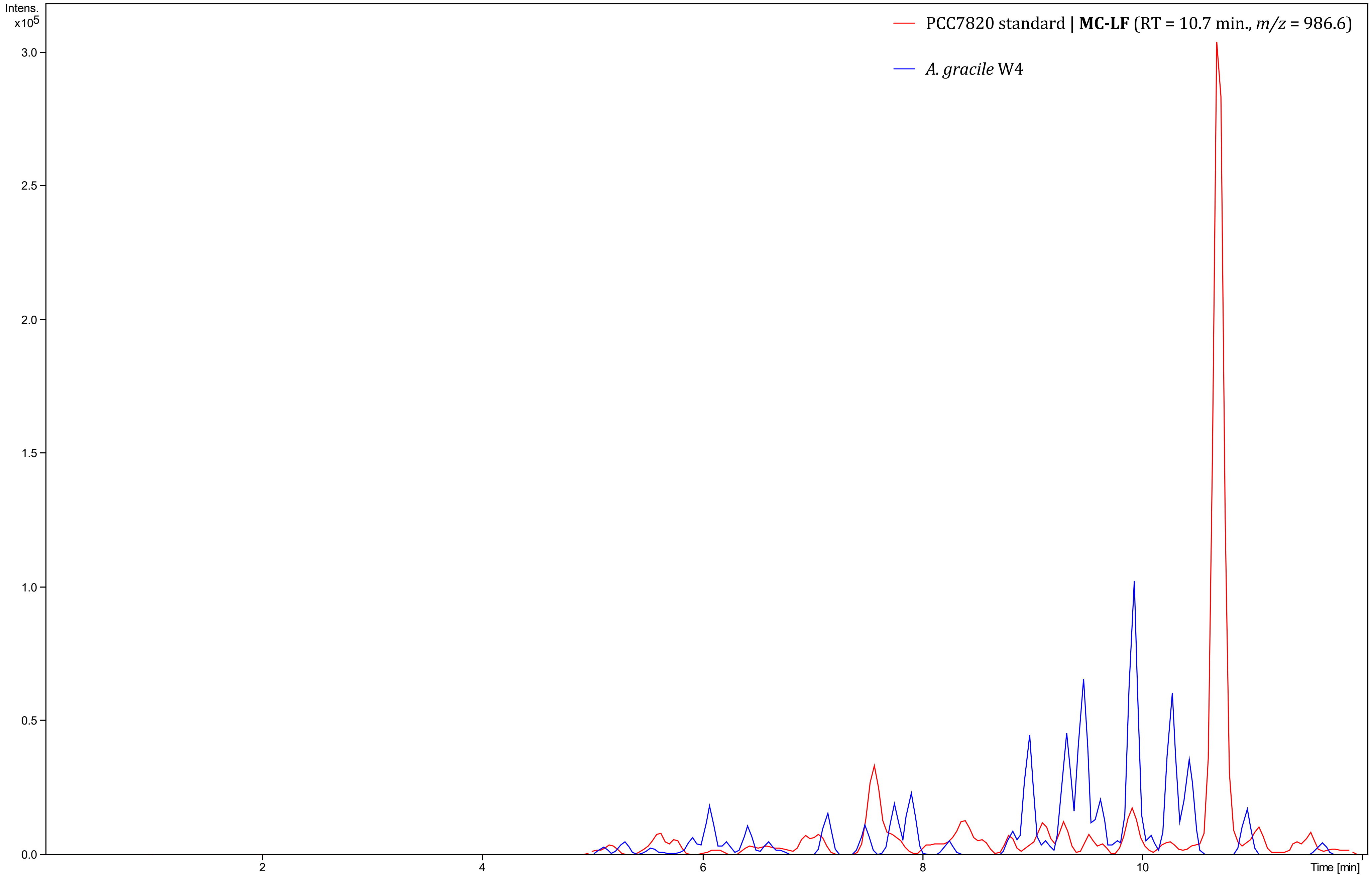

***A. gracile* W89**

LC-MS analysis | extracted ion chromatogram (*m/z* 513.0) of NIES107 standard and *A. gracile* W89

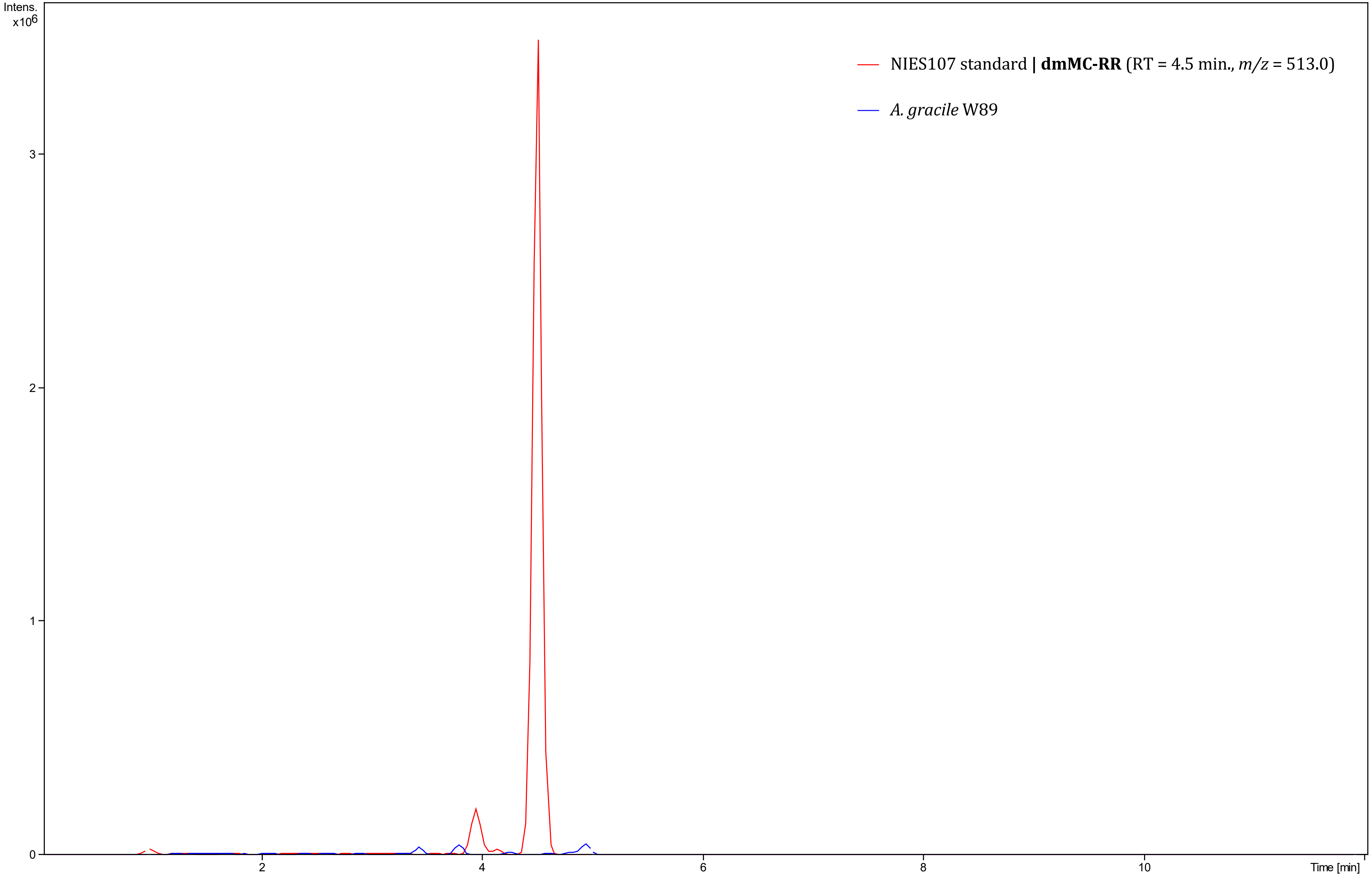

LC-MS analysis | extracted ion chromatogram (m/z 520.0) of NIES107 standard and *A. gracile* W89

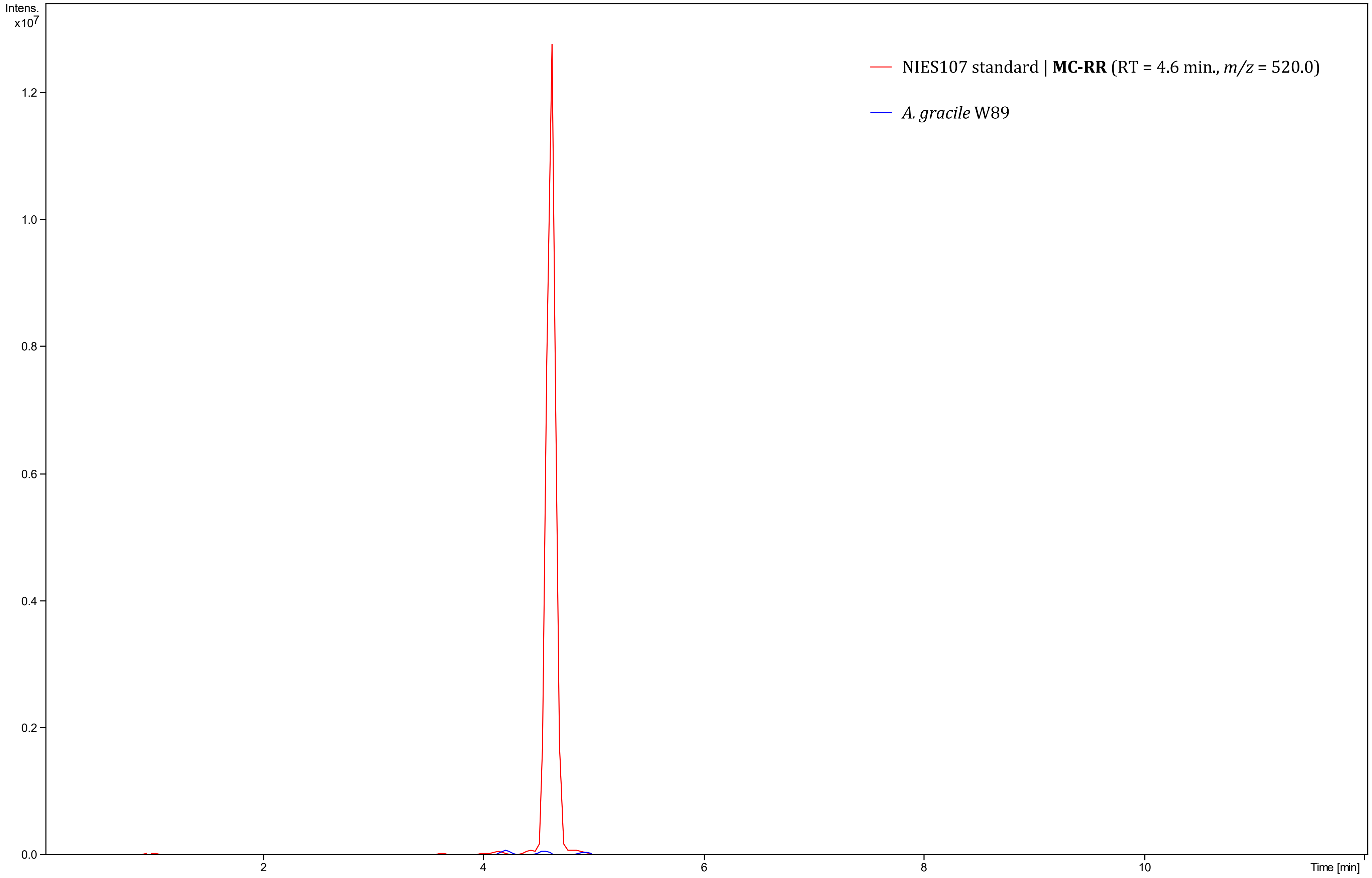

LC-MS analysis | extracted ion chromatogram (*m/z* 1045.6) of NIES107 standard and *A. gracile* W89

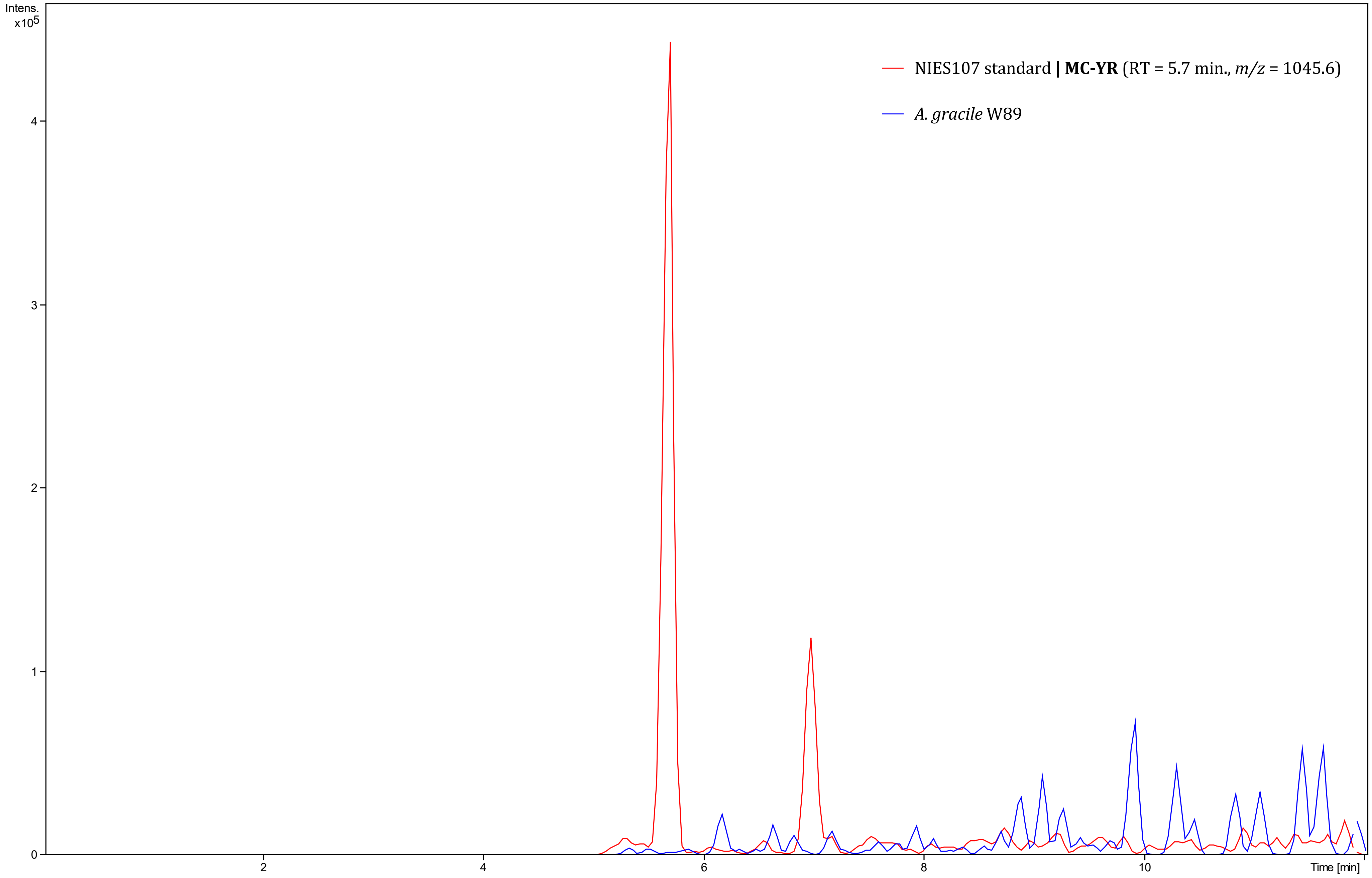

LC-MS analysis | extracted ion chromatogram (*m/z* 995.5) of PCC7820 standard and *A. gracile* W89

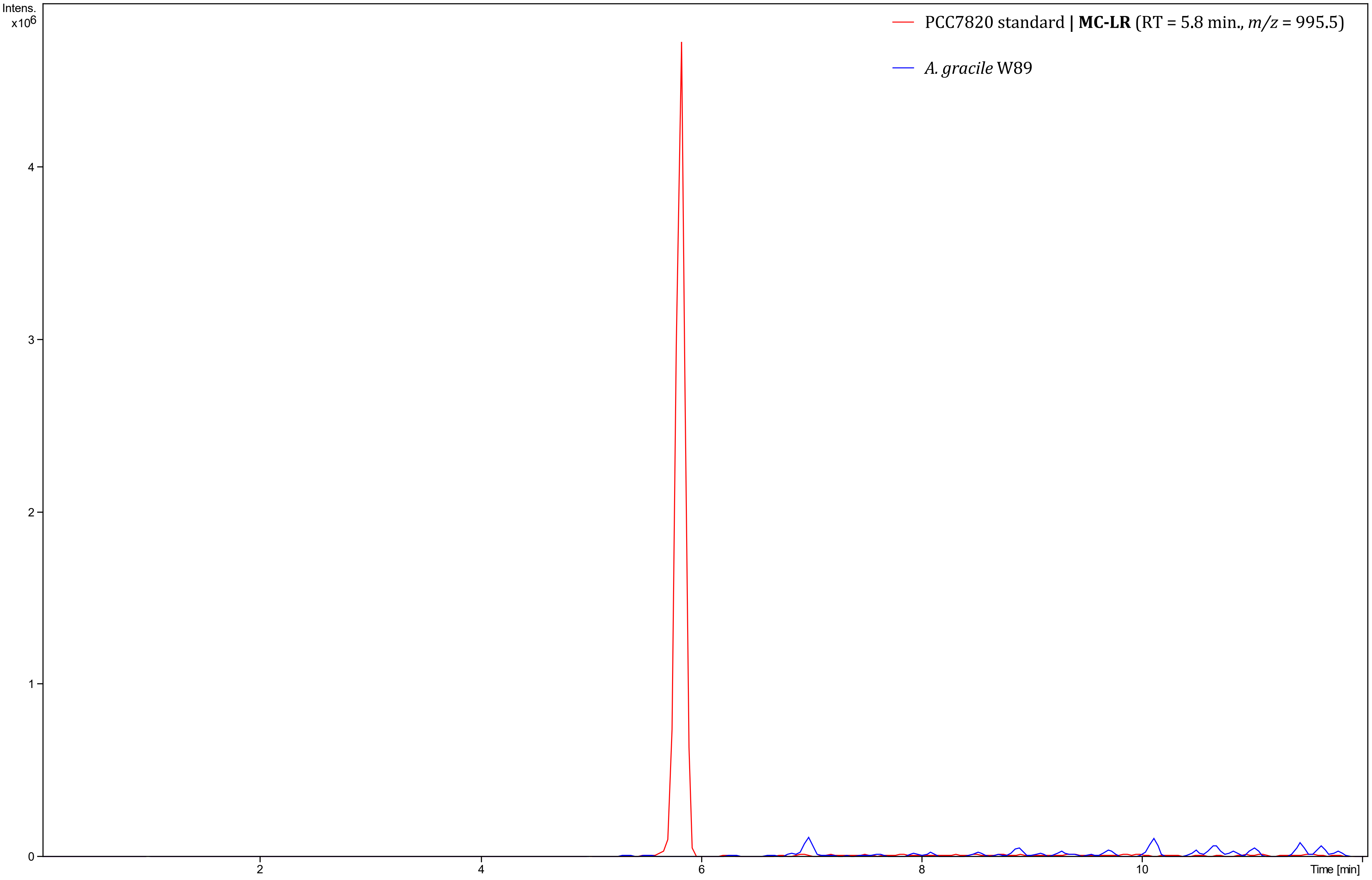

LC-MS analysis | extracted ion chromatogram (*m/z* 1002.5) of PCC7820 standard and *A. gracile* W89

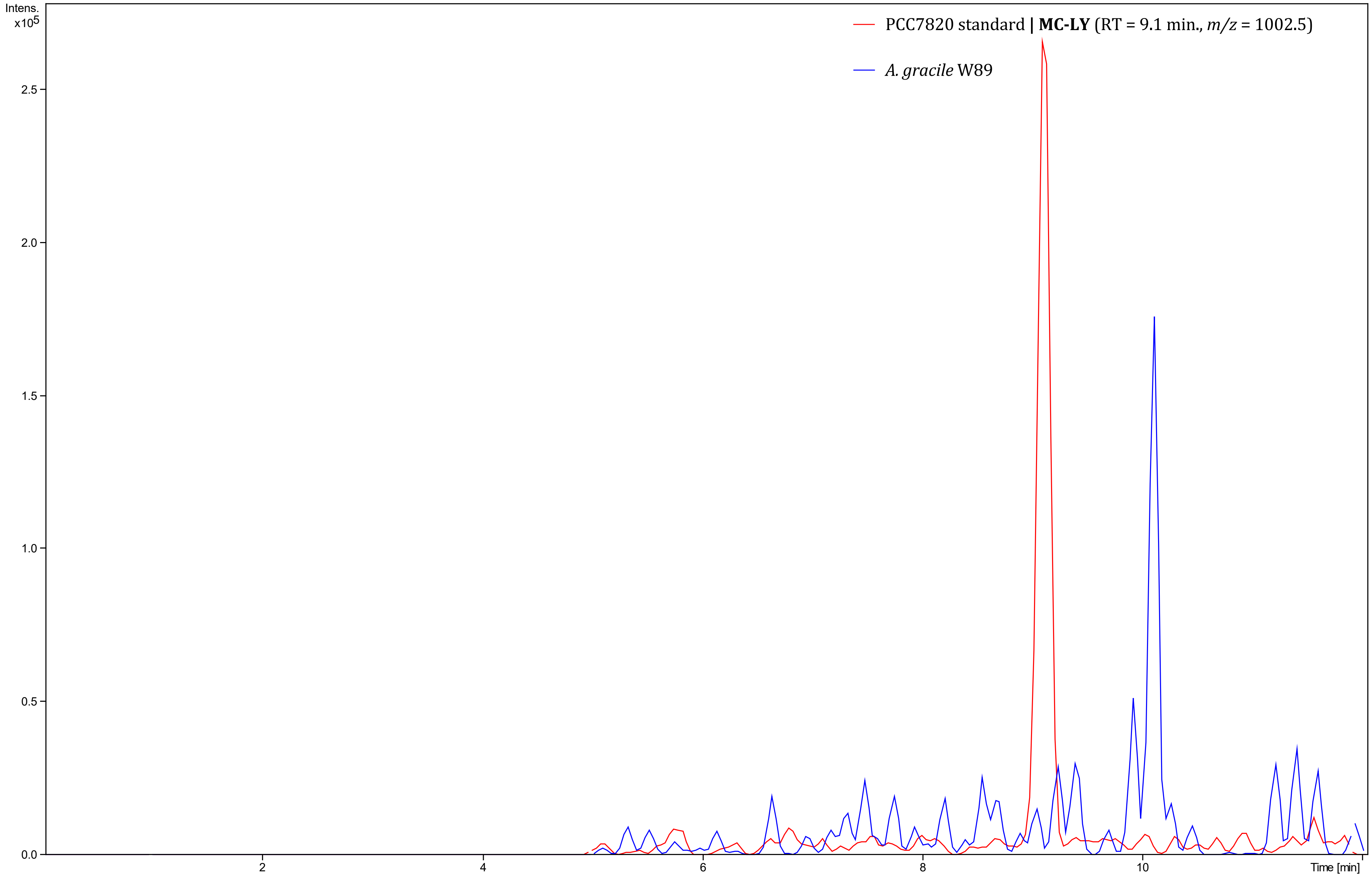

LC-MS analysis | extracted ion chromatogram (*m/z* 1025.9) of PCC7820 standard and *A. gracile* W89

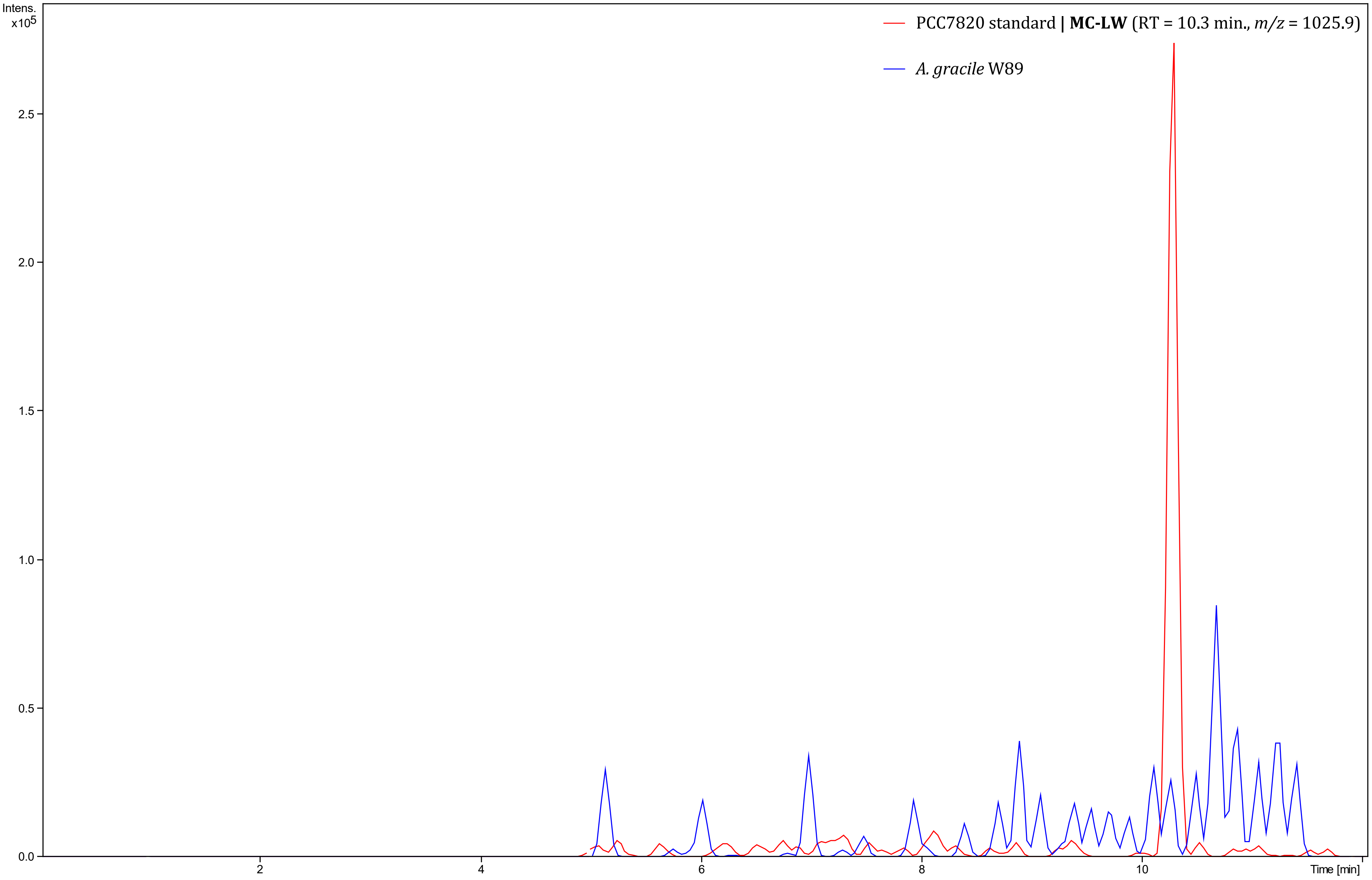

LC-MS analysis | extracted ion chromatogram (*m/z* 986.6) of PCC7820 standard and *A. gracile* W89

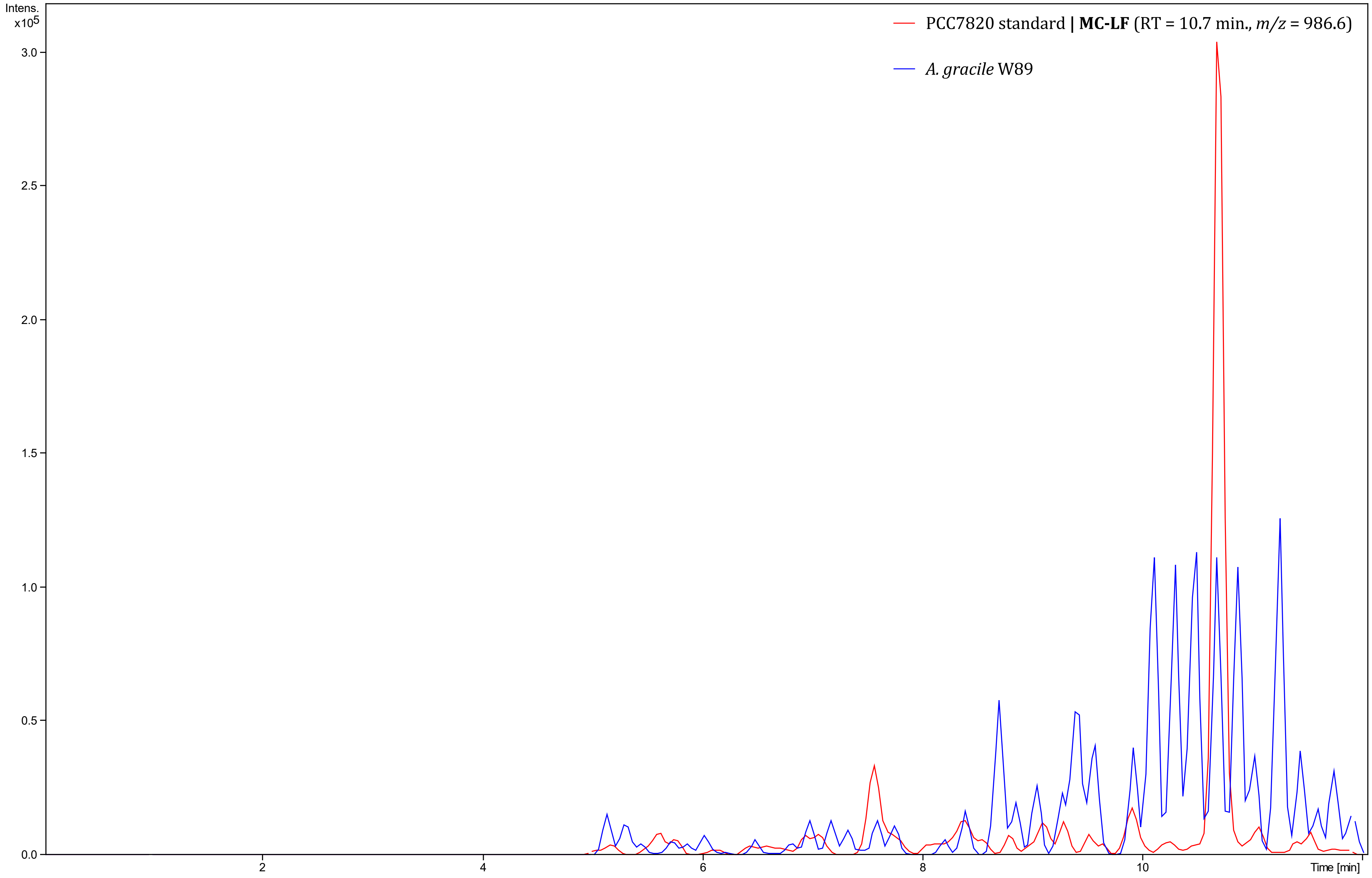

***P. agardhii* W70**

LC-MS analysis | extracted ion chromatogram (*m/z* 513.0) of NIES107 standard, *P. agardhii* W70, and mass spectrum scan of the dmMC-RR in W70 strain

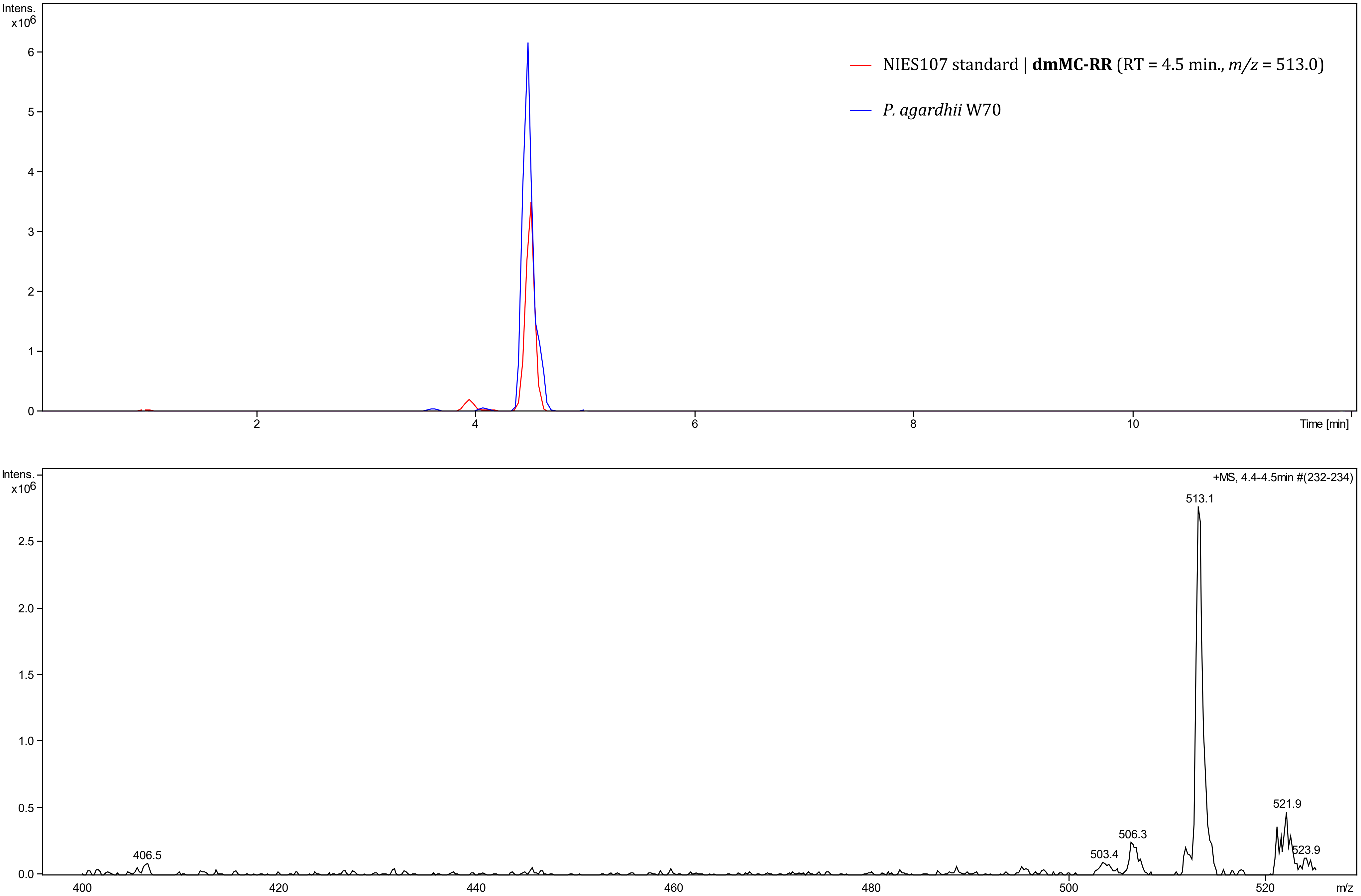

LC-MS analysis | extracted ion chromatogram (*m/z* 520.0) of NIES107 standard and *P. agardhii* W70

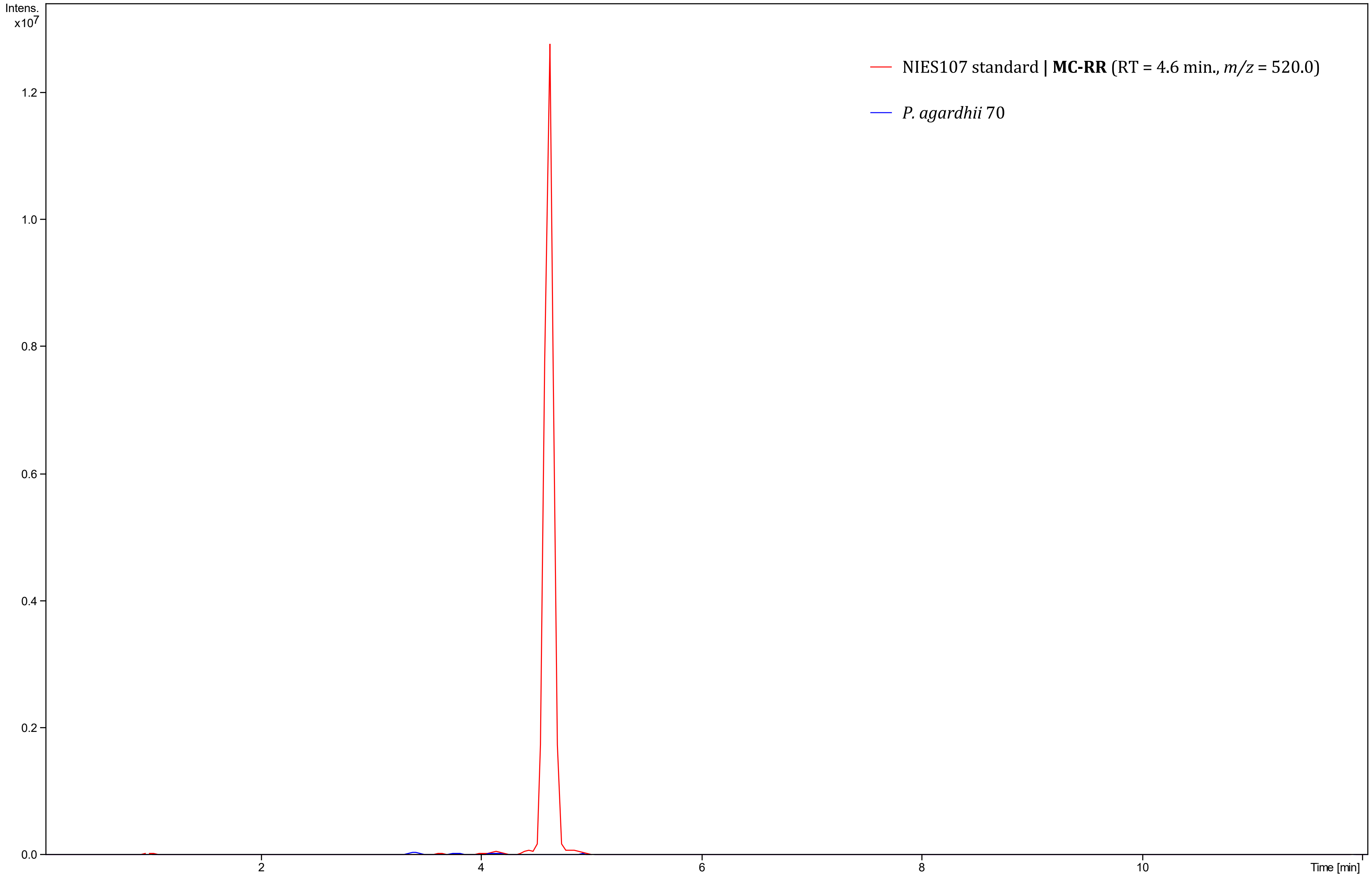

LC-MS analysis | extracted ion chromatogram (*m/z* 1045.6) of NIES107 standard and *P. agardhii* W70

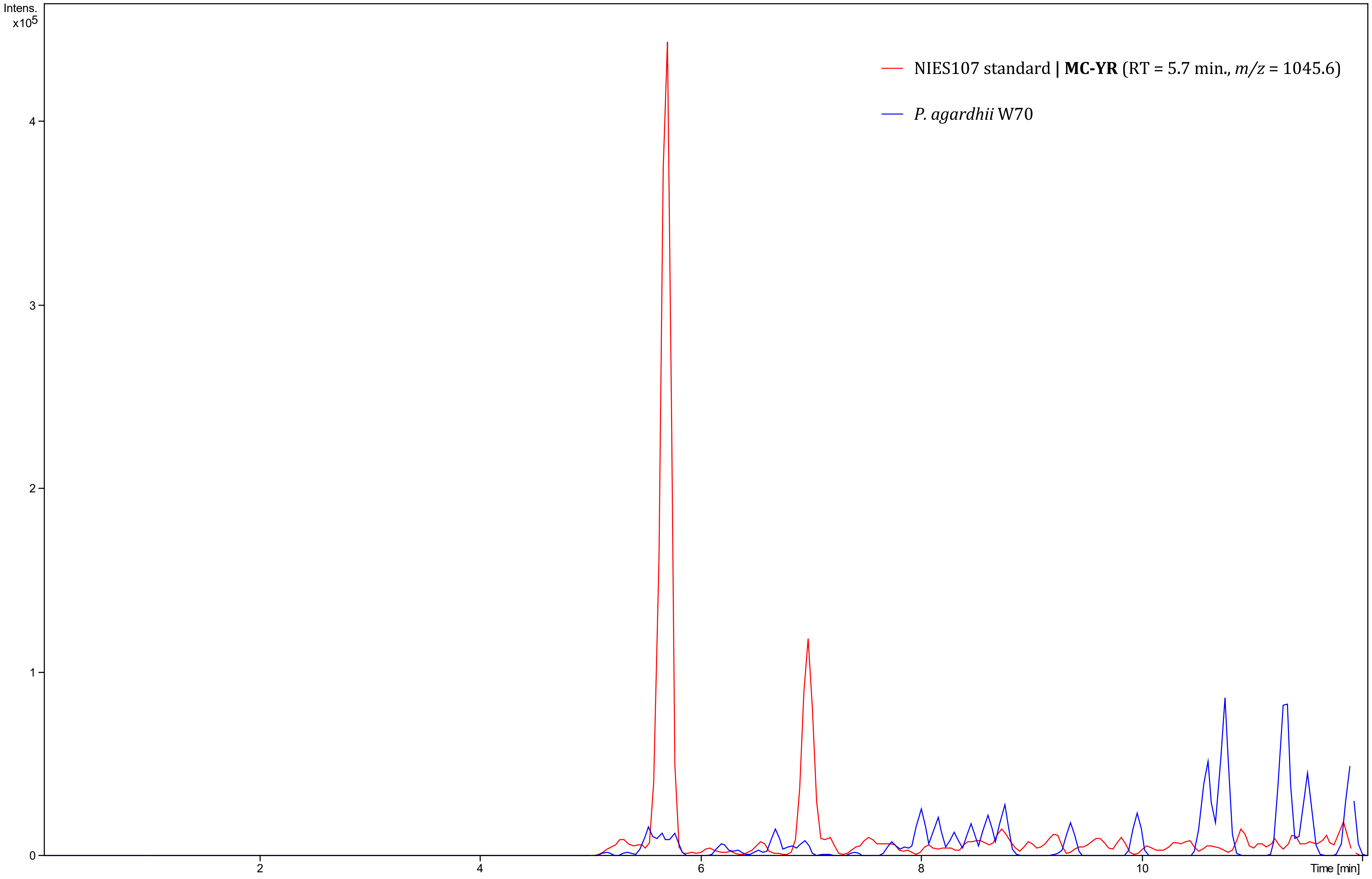

LC-MS analysis | extracted ion chromatogram (*m/z* 995.5) of PCC7820 standard and *P. agardhii* W70

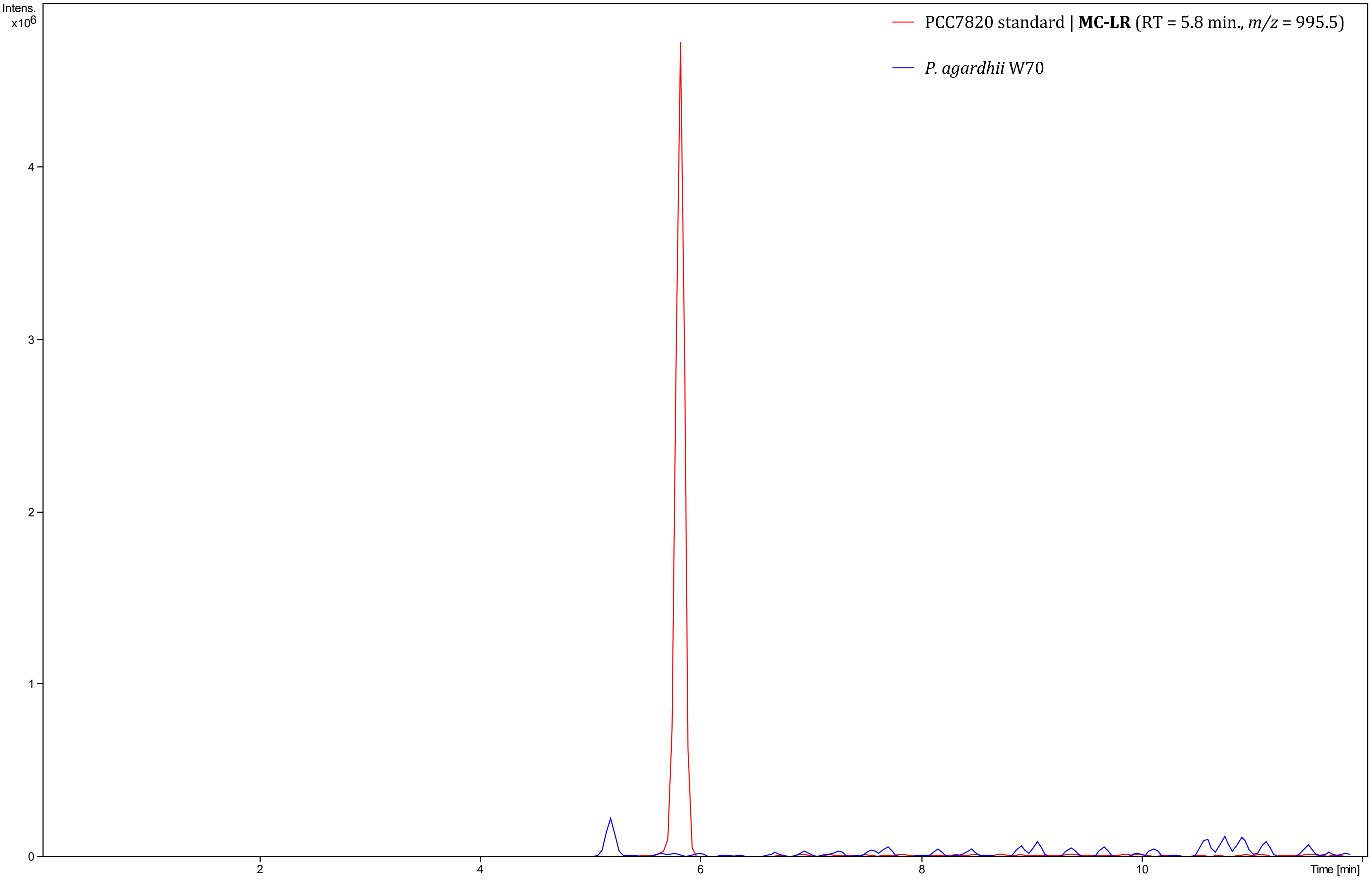

LC-MS analysis | extracted ion chromatogram (*m/z* 1002.5) of PCC7820 standard and *P. agardhii* W70

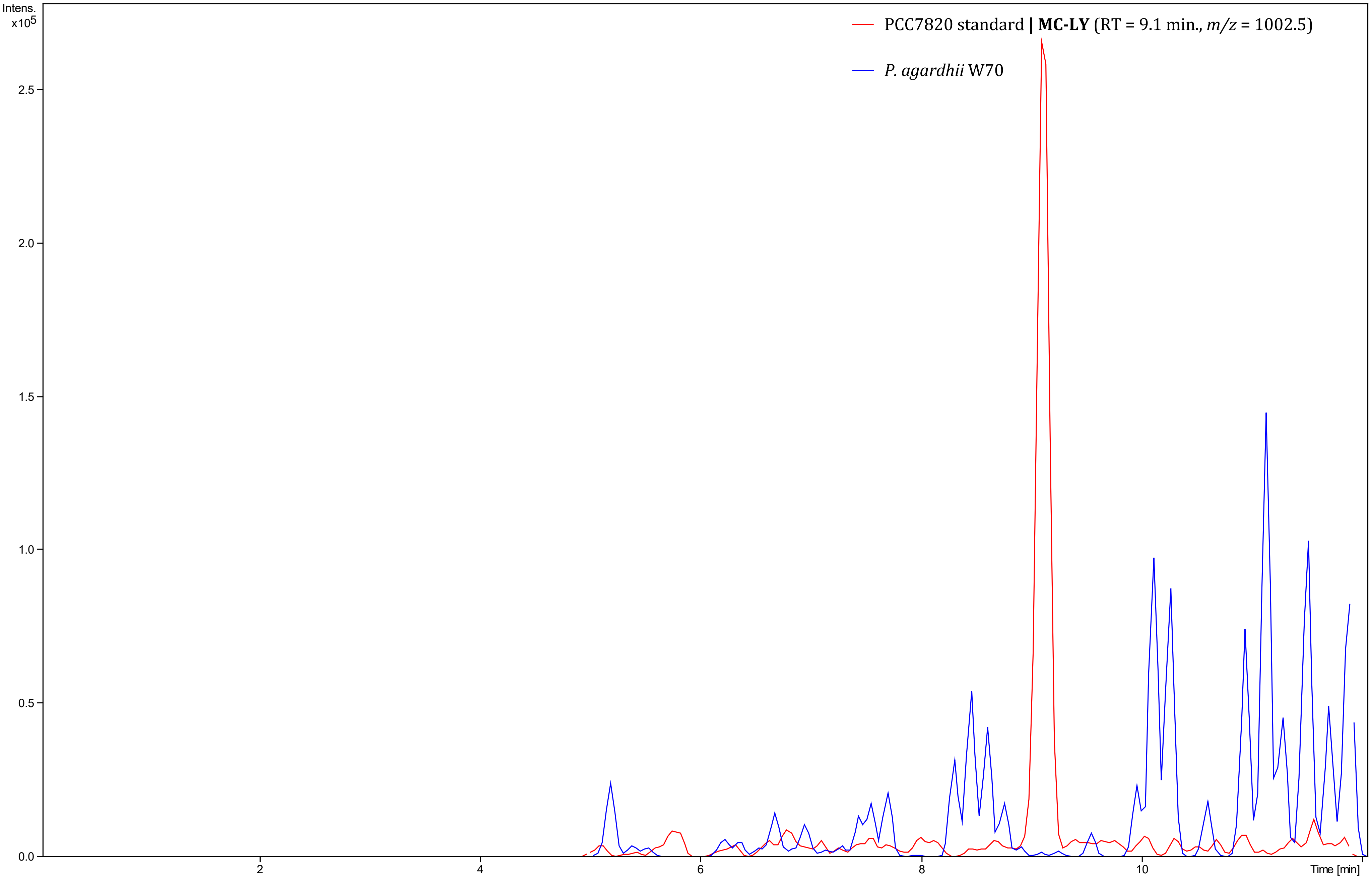

LC-MS analysis | extracted ion chromatogram (m/z 1025.9) of PCC7820 standard and *P. agardhii* W70

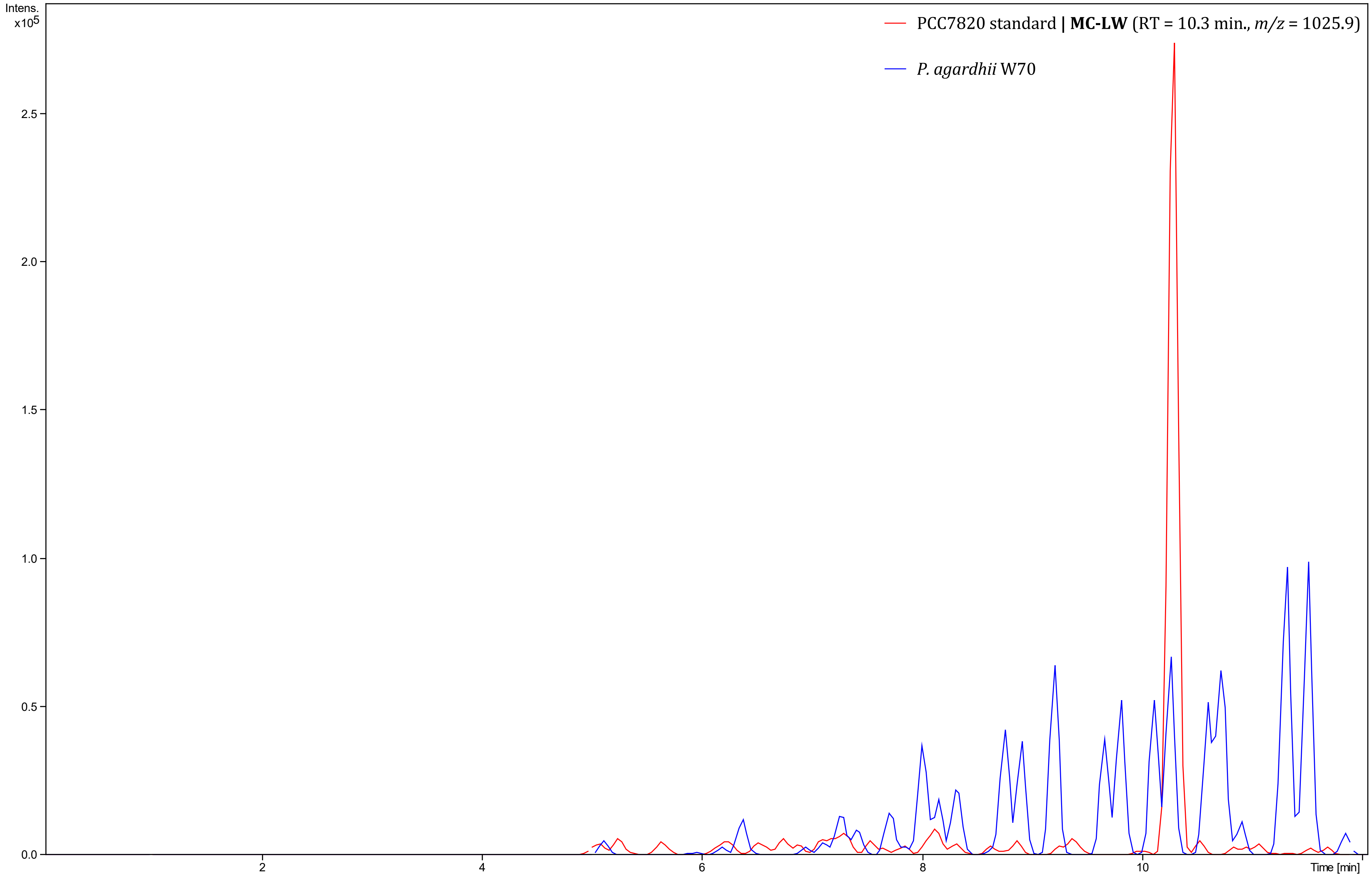

LC-MS analysis | extracted ion chromatogram (*m/z* 986.6) of PCC7820 standard and *P. agardhii* W70

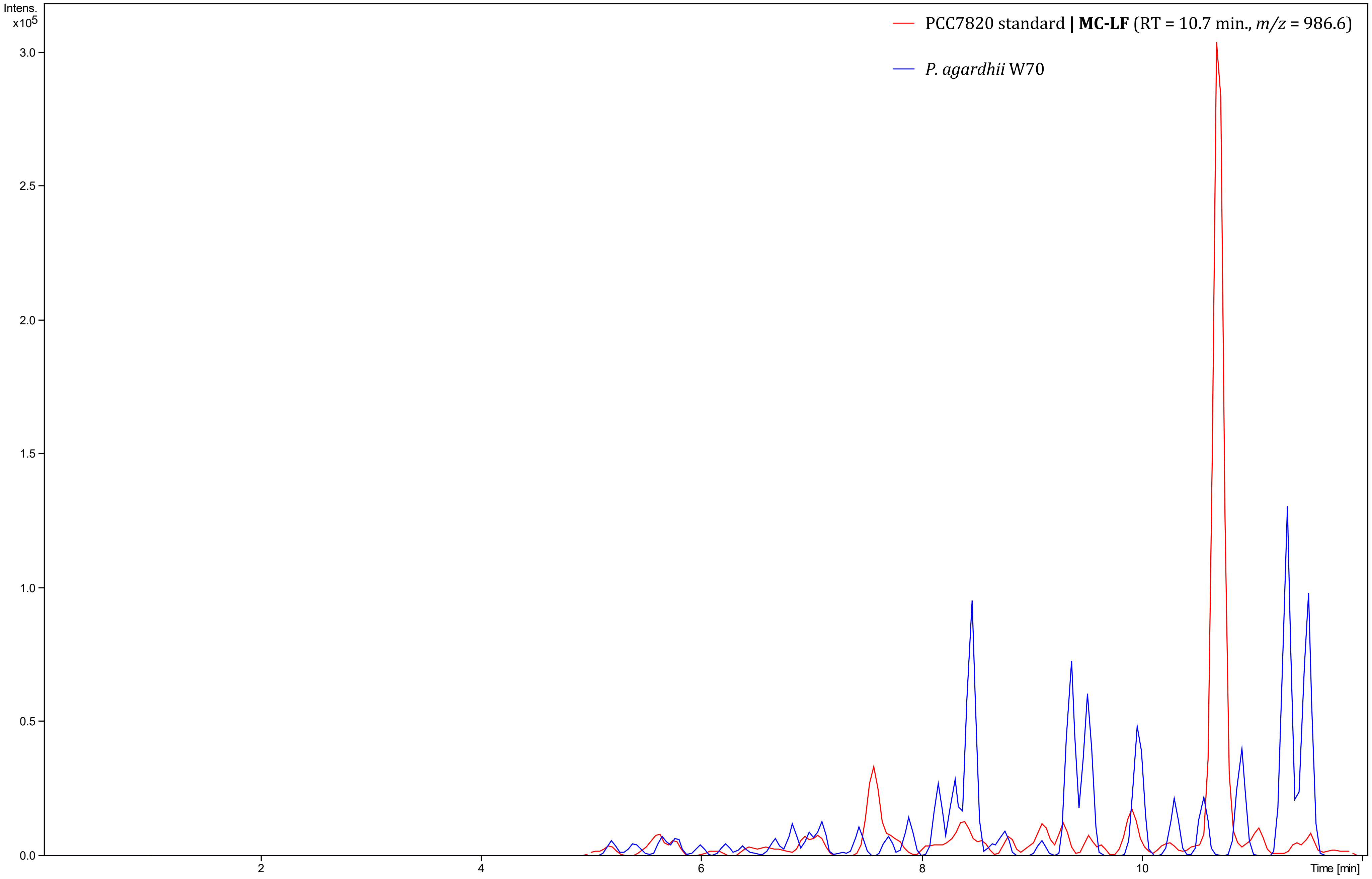

. / $

Extracted ion chromatograms are presented for all samples. Mass spectrum scan is shown for examined strains of cyanobacteria in the case when it matched the mass spectrum scan of the NOD in the standard.

LC-MS analysis | extracted ion chromatogram (*m/z* 825.5) of NOD standard and mass spectrum scan of the NOD in the standard

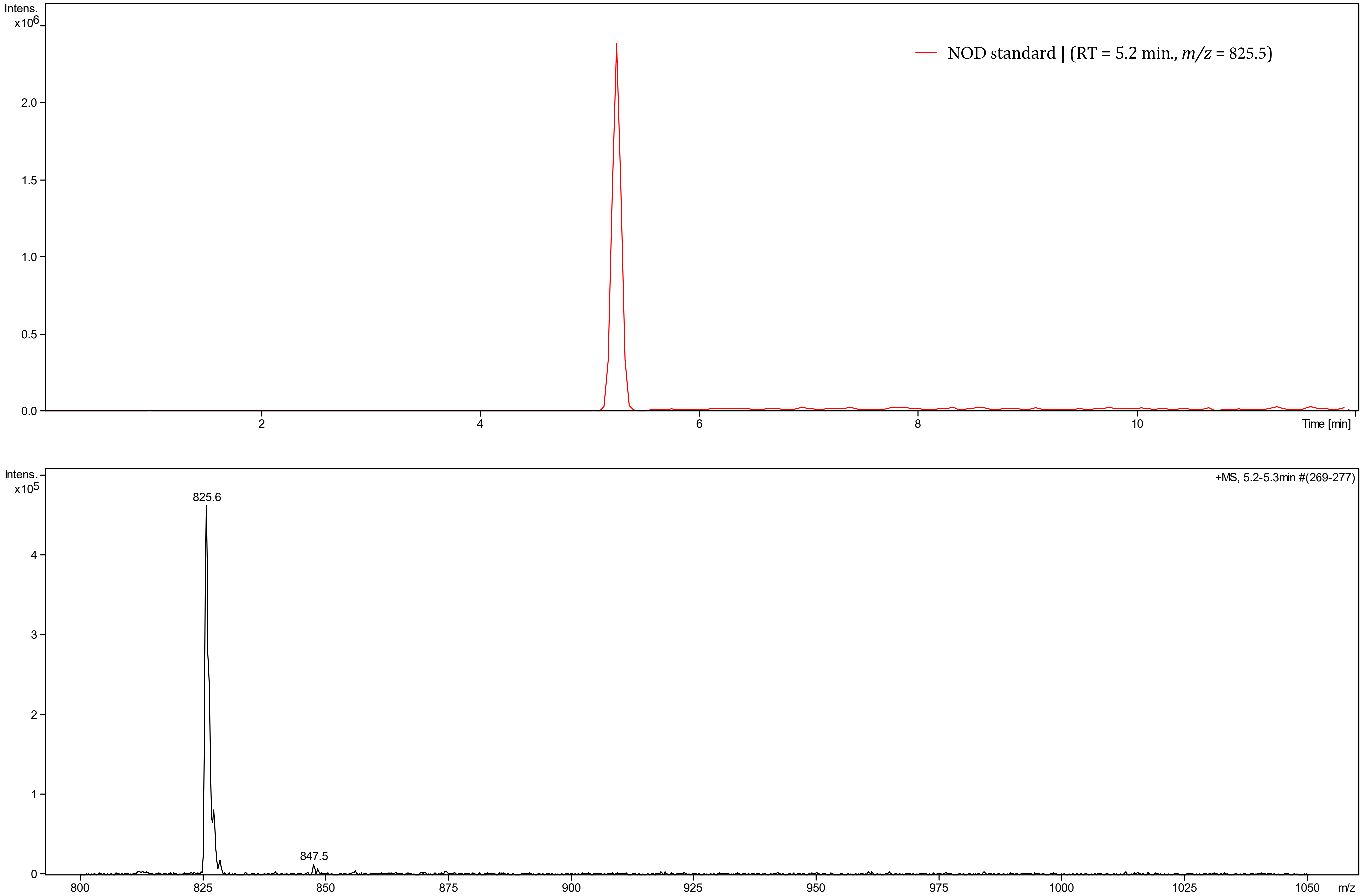

LC-MS analysis | extracted ion chromatogram (*m/z* 825.5) of NOD standard and *P. agardhii* W67

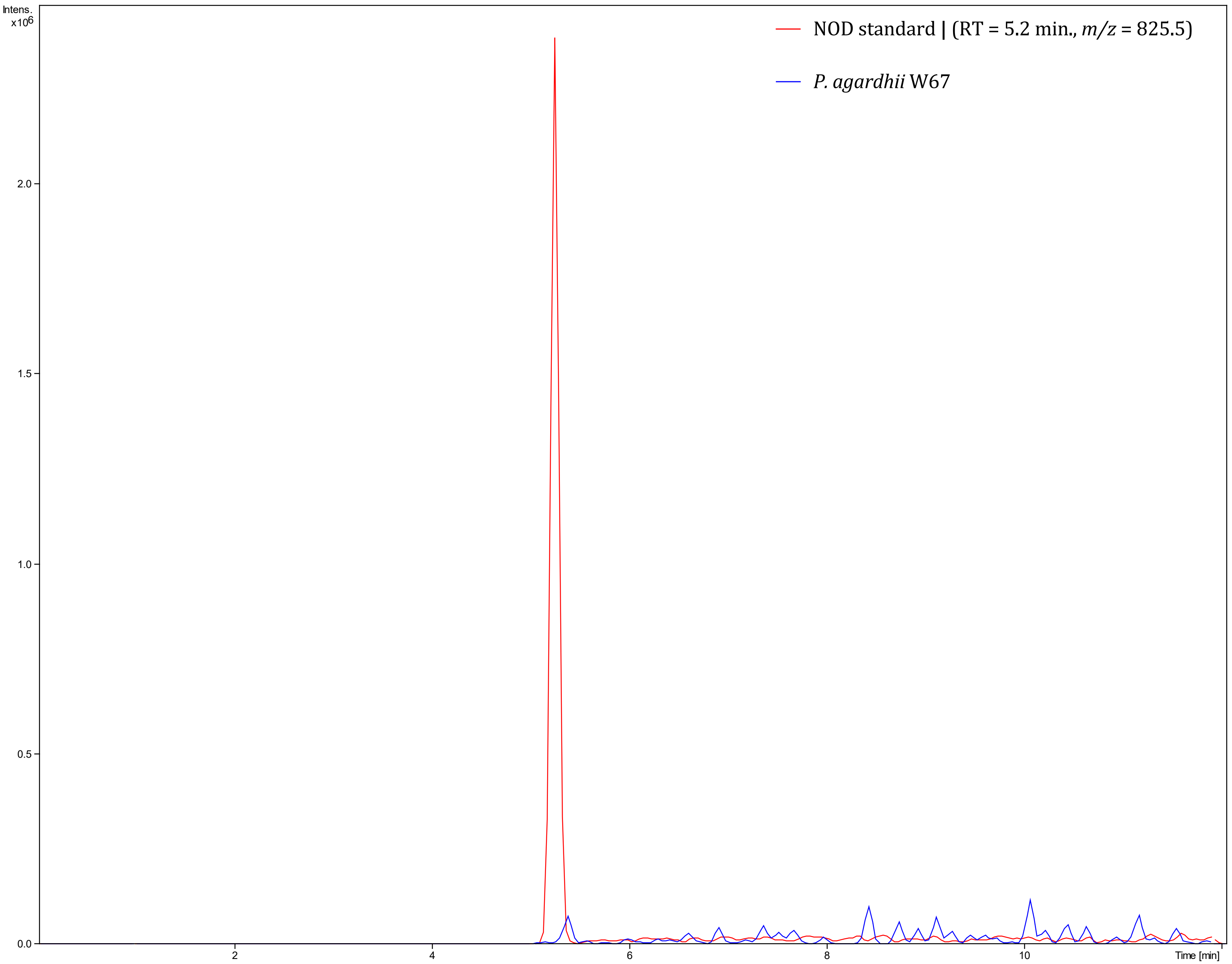

LC-MS analysis | extracted ion chromatogram (*m/z* 825.5) of NOD standard and *P. agardhii* W49, and mass spectrum scan of the compound in *P. agardhii* W49 of RT = 5.4 min. (mass spectrum scan of the compound in W49 of RT similar to RT of the NOD in the standard did not match the mass spectrum scan of the NOD in the standard)

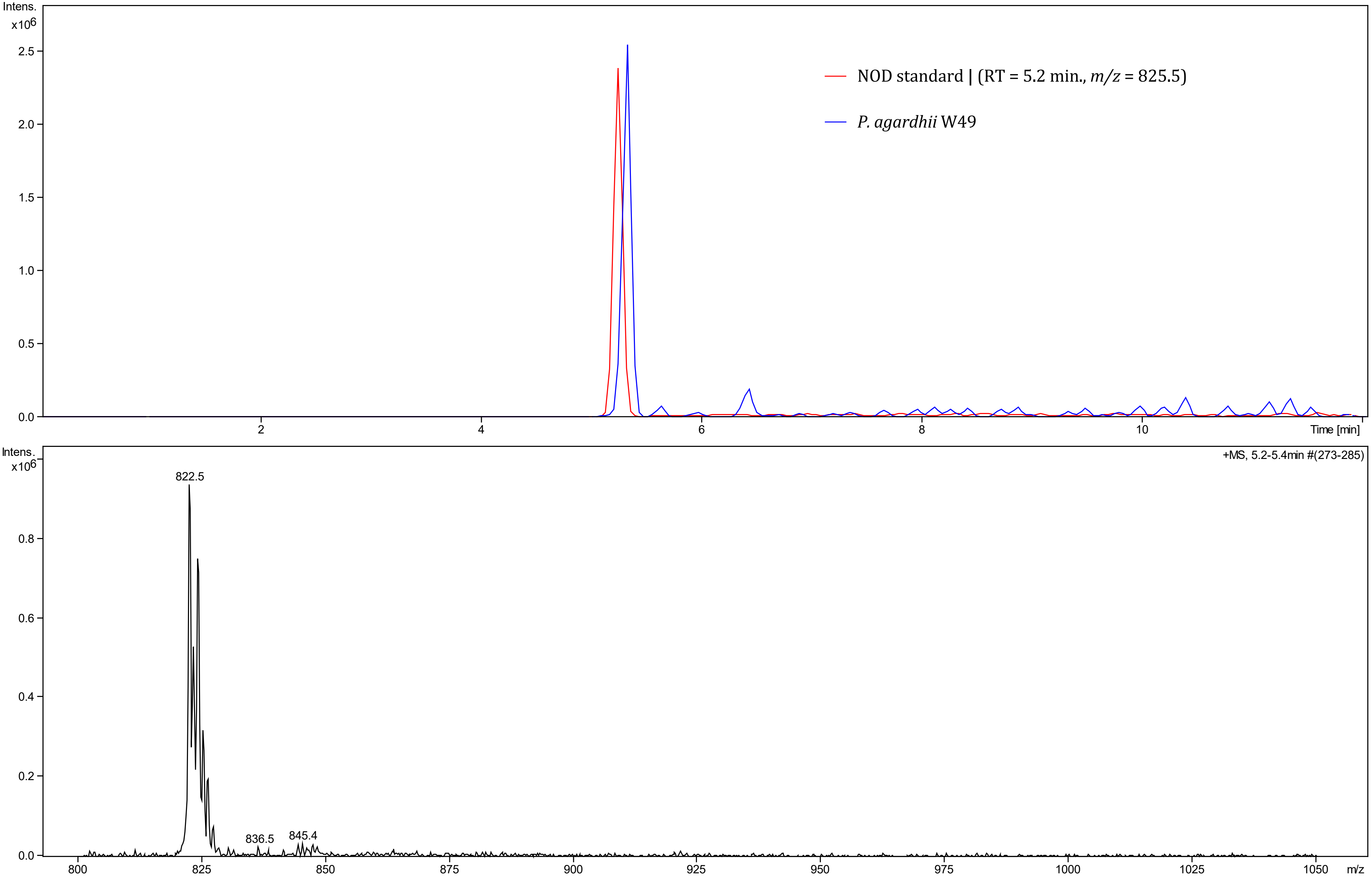

LC-MS analysis | extracted ion chromatogram (*m/z* 825.5) of NOD standard and *R. raciborskii* W88

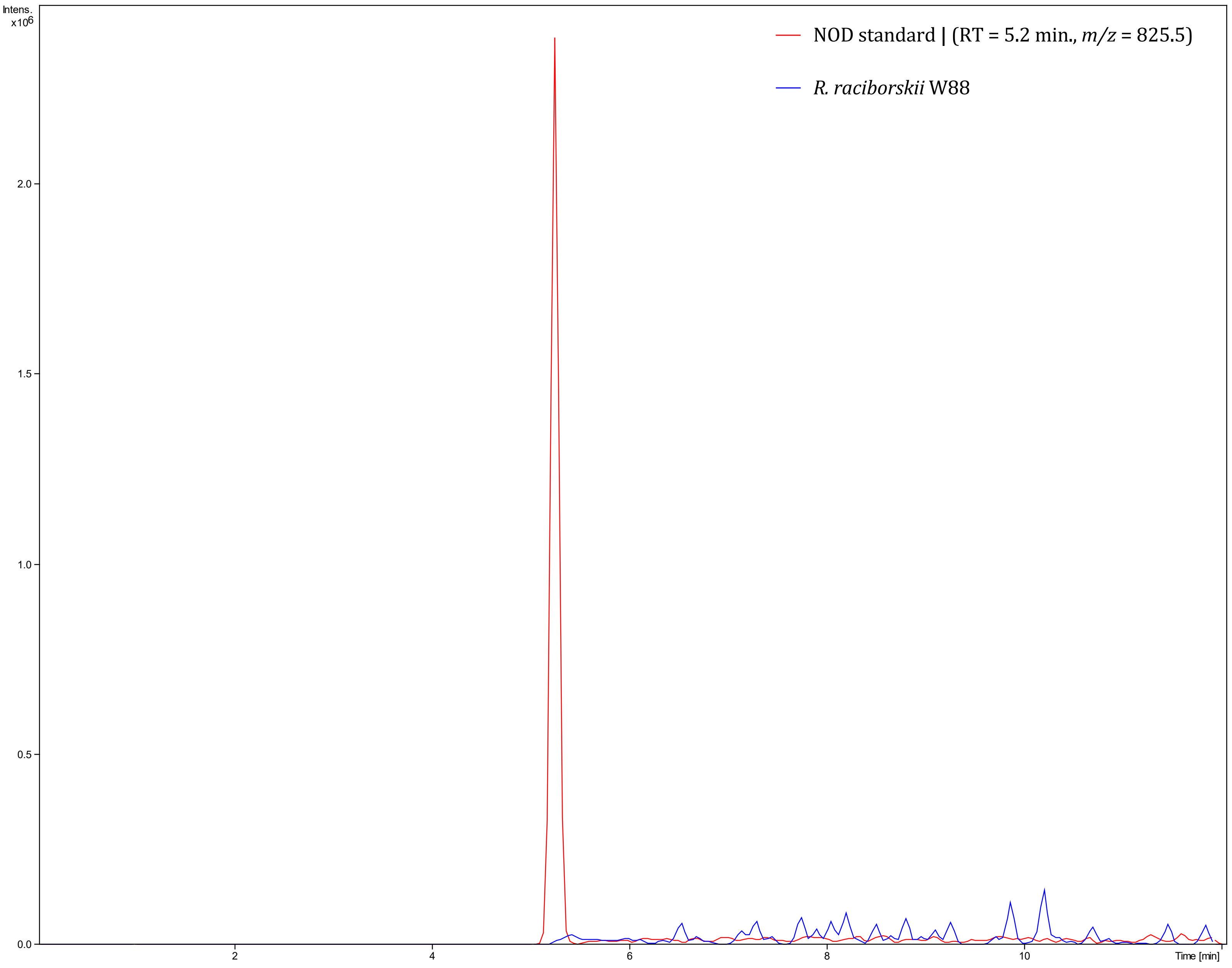

LC-MS analysis | extracted ion chromatogram (*m/z* 825.5) of NOD standard and *R. raciborskii* W73

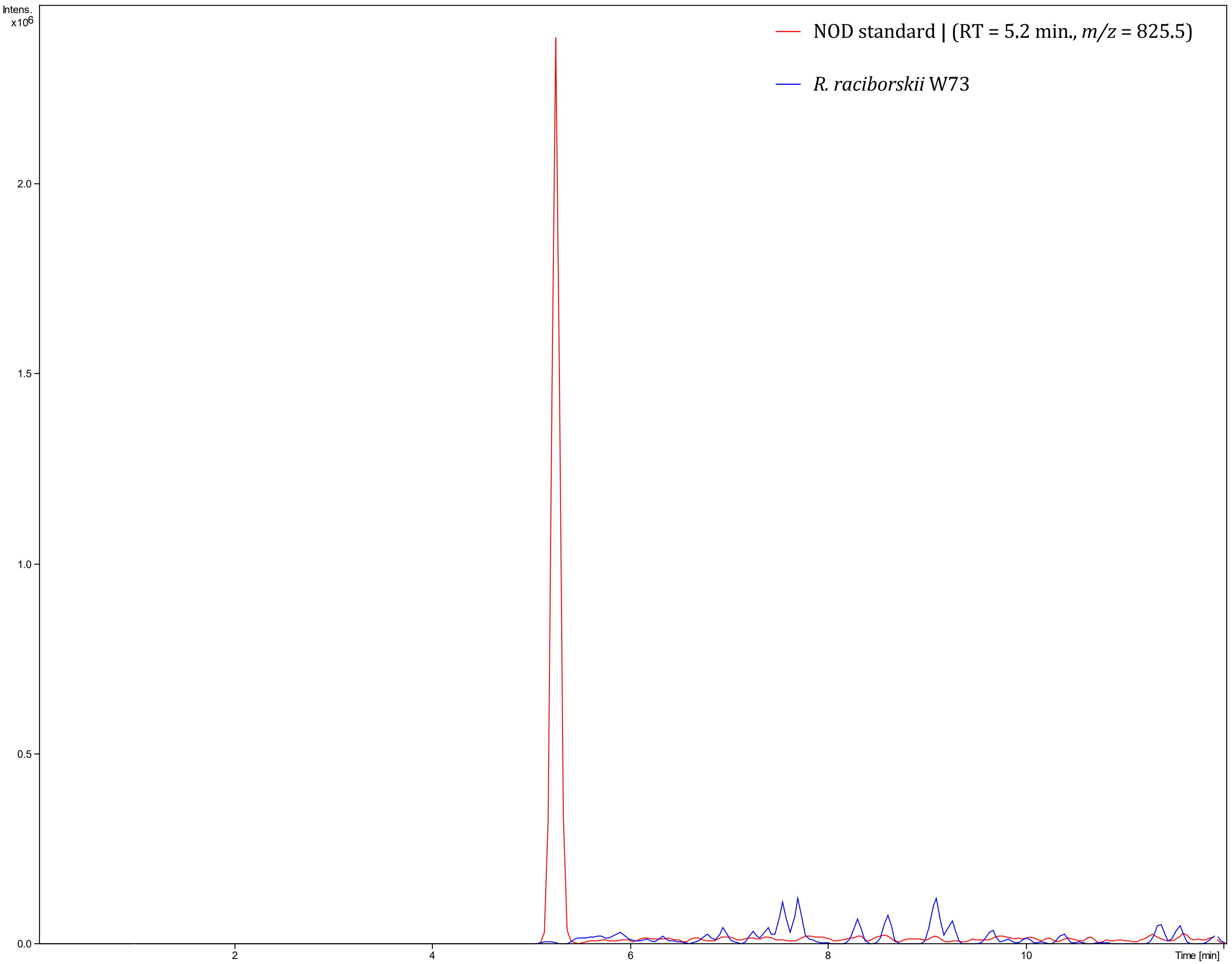

LC-MS analysis | extracted ion chromatogram (*m/z* 825.5) of NOD standard and *A. gracile* W71

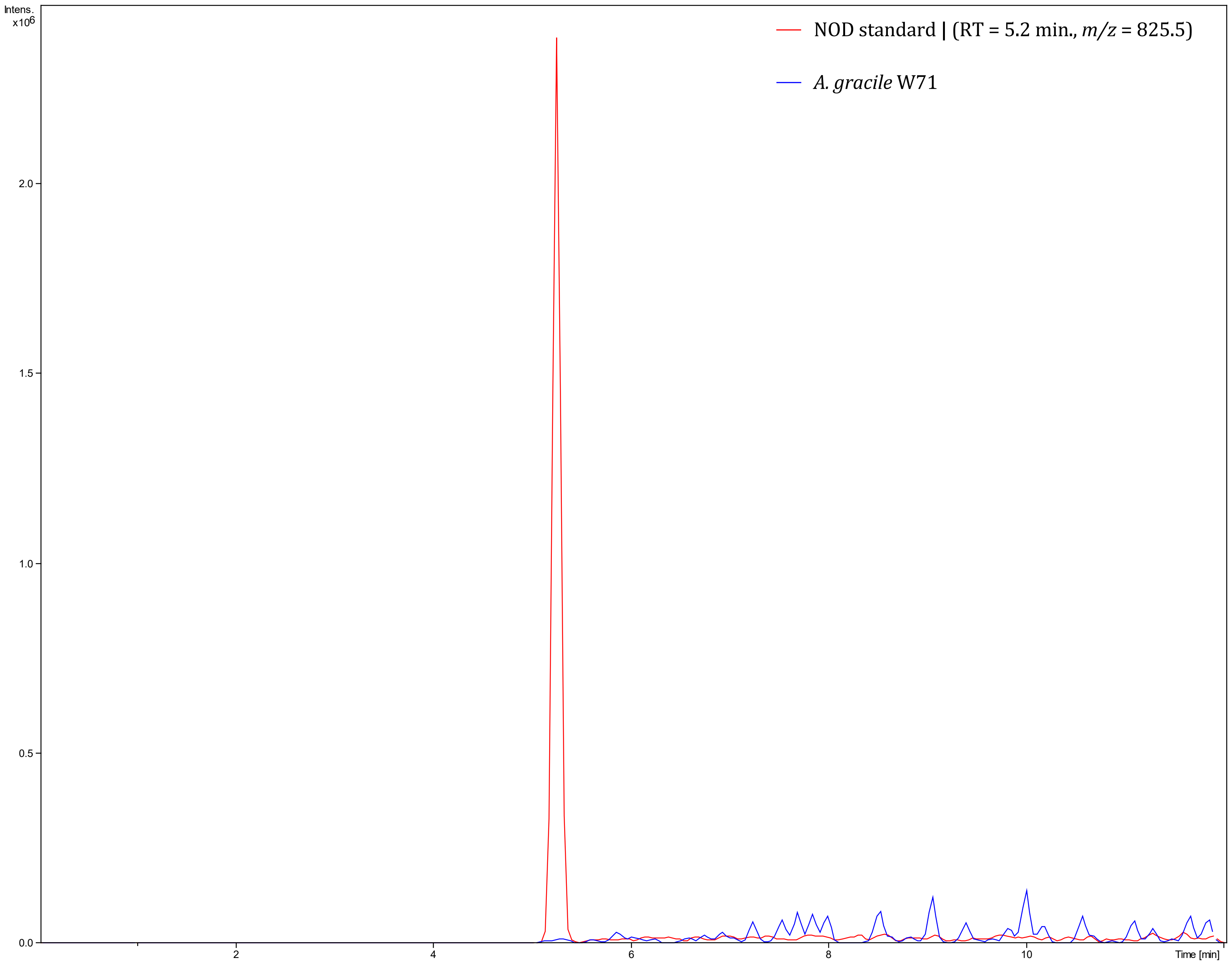

LC-MS analysis | extracted ion chromatogram (*m/z* 825.5) of NOD standard and *A. gracile* W4

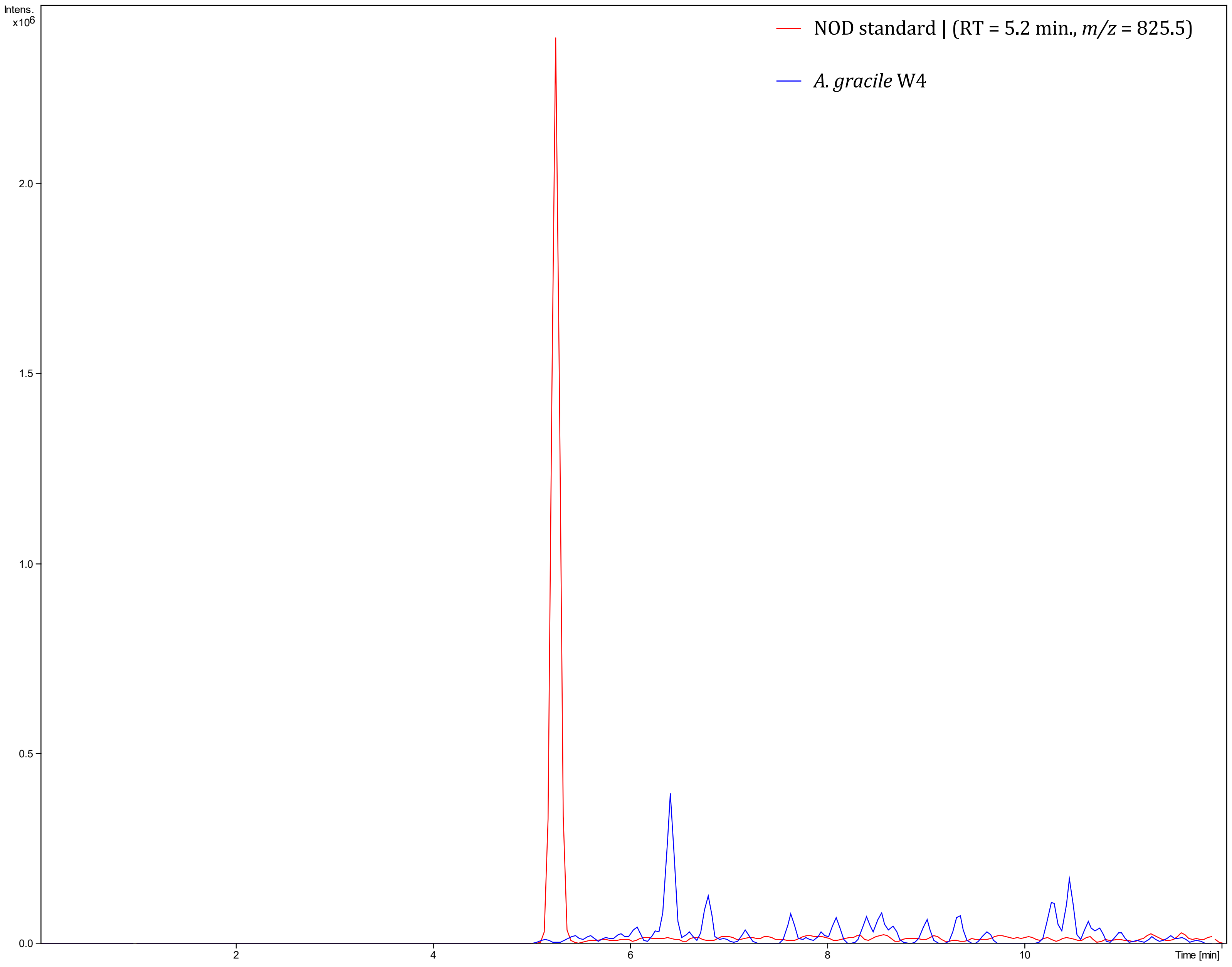

LC-MS analysis | extracted ion chromatogram (*m/z* 825.5) of NOD standard and *A. gracile* W89

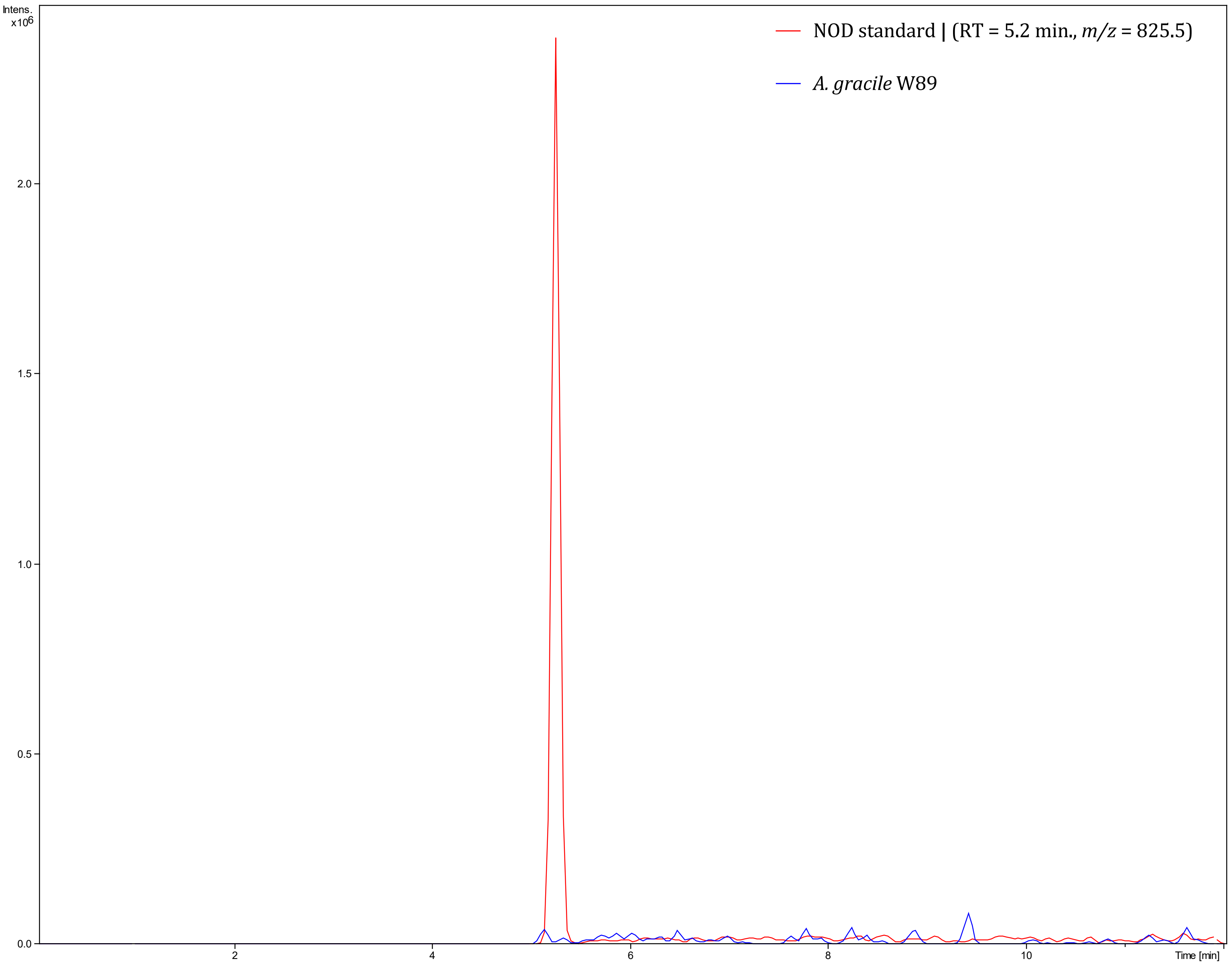

LC-MS analysis | extracted ion chromatogram (*m/z* 825.5) of NOD standard and *P. agardhii* W70

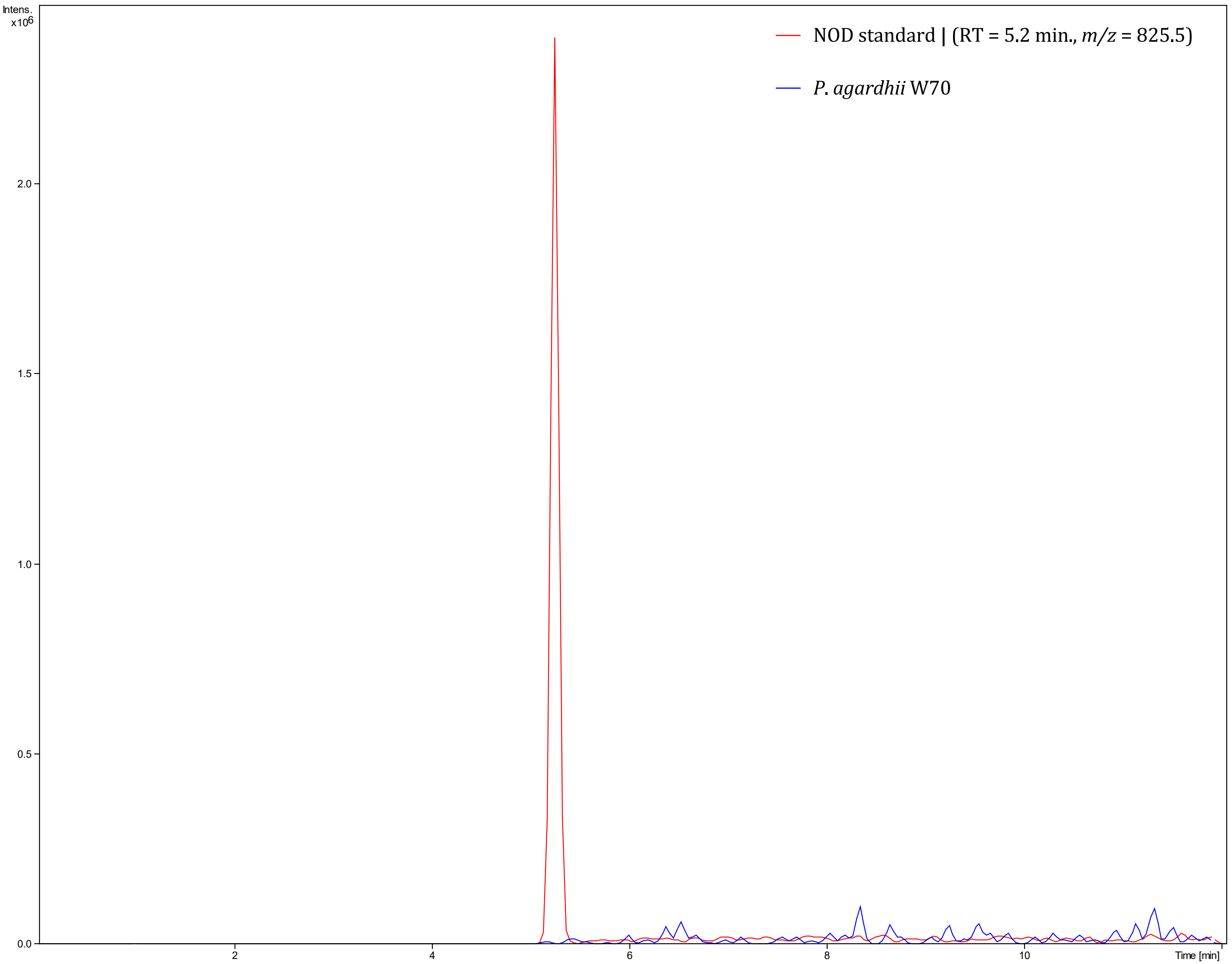

Supplement: Supplementary file 1 [file toxins-16-00357-s001.zip › S7.pdf]
