# Supplementary material for: Community Structure and Toxicity Potential of Cyanobacteria during Summer and Winter in a Temperate-Zone Lake Susceptible to Phytoplankton Blooms
Source: Toxins (Basel). 2024 Aug 14;16(8):357. doi: 10.3390/toxins16080357 (PMC11359657; doi:10.3390/toxins16080357)
Supplement: Supplementary file 1 [file toxins-16-00357-s001.zip › S1.pdf]

Community Structure and Toxicity Potential of Cyanobacteria during Summer and Winter in a Temperate-Zone Lake Susceptible to Phytoplankton Blooms

Łukasz Wejnerowski[1*], Tamara Dulić[2], Sultana Akter[3], Arnoldo Font-Nájera[4], Michał Rybak[5], Oskar Kamiński[1], Anna Czerepska[1], Marcin Krzysztof Dziuba[6], Tomasz Jurczak[7], Jussi Meriluoto[2*], Joanna Mankiewicz-Boczek[7], Mikołaj Kokociński[1]

[1] Department of Hydrobiology, Institute of Environmental Biology, Faculty of Biology, Adam Mickiewicz University, Uniwersytetu Poznańskiego 6, 61-614 Poznań, Poland;

[2] Biochemistry and Cell Biology, Faculty of Science and Engineering, Åbo Akademi University, Tykistökatu 6A, 20520 Turku, Finland;

[3] Biotechnology, Department of Life Technologies, Faculty of Technology, University of Turku, 20520 Turku, Finland;

[4] European Regional Centre for Ecohydrology of the Polish Academy of Sciences, Tylna 3, 90-364 Łódź, Poland;

[5] Department of Water Protection, Institute of Environmental Biology; Faculty of Biology; Adam Mickiewicz University; Uniwersytetu Poznańskiego 6, 61-614 Poznań, Poland;

[6] Department of Ecology and Evolutionary Biology, University of Michigan; MI 48109 Ann Arbor, USA;

[7] University of Lodz, Faculty of Biology and Environmental Protection, UNESCO Chair on Ecohydrology and Applied Ecology; Banacha 12/16, 90-237 Łódź, Poland;

Correspondence: wejner@amu.edu.pl (Ł.W.); Jussi.Meriluoto@abo.fi (J.M.)

Supplementary Information S1

Biomasses of phytoplankton taxa in Lubosińskie Lake during summer (2 September 2019) and winter (11 February 2020)

Group	Taxon<br>Authority	Summary biomass [mg L^{-1}]	Winter biomass [mg L^{-1}]
Cyanobacteria	*Aphanizomenon gracile*<br>Lemmermann 1907	1.394064	0.464688
	Raphidiopsis raciborskii<br>(Woloszynska) Aguilera & al. 2018	2.578944	0
	Jaaginema subtilissimum<br>(Kützing ex Forti) Anagnostidis & Komárek 1988	2.69568	0.08424
	Limnothrix obliqueacuminata<br>(Skuja) Meffert 1988	0.083664	0.035856
	Limnothrix planctonica<br>(Woloszynska) Meffert 1988	0	3.2736
	Limnothrix redekei<br>(Goor) Meffert 1988	1.797336	2.419056
	Limnothrix sp.<br>M.-E.Meffert 1988	7.58784	0
	Merismopedia tenuissima<br>Lemmermann 1898	0.007872	0
	Planktothrix agardhii<br>(Gomont) Anagnostidis & Komárek 1988	10.390176	0.161088
	Pseudanabaena limnetica<br>(Lemmermann) Komárek 1974	0.246384	0.16992
	Raphidiopsis mediterranea<br>Skuja 1937	0.066096	0
	Sphaerospermopsis aphanizomenoides<br>(Forti) Zapomelová, & al. 2010	0.7296	0
	Synechocystis sp.<br>Sauvageau 1892	0.011232	0
Bacillariophyceae	*Fragilaria capucina*<br>Desmazières 1830	0	0.0468
	Nitzschia acicularis<br>(Kützing) W.Smith 1853	0.012048	0
	Ulnaria acus<br>(Kützing) Aboal 2003	0	1.86
	Ulnaria ulna<br>(Nitzsch) Compère 2001	0.078	0
Chlorophyta	*Coelastrum microporum*<br>Nägeli 1855	0.007536	0
	Crucigenia tetrapedia<br>(Kirchner) Kuntze 1898	0.00504	0
	Desmodesmus communis<br>(E.Hegewald) E.Hegewald 2000	0.009	0
	Monoraphidium griffithii<br>(Berkeley) Komárková-Legnerová 1969	0	0.025728
	Schroederia setigera<br>(Schröder) Lemmermann 1898	0.006624	0
	Tetraëdron caudatum<br>(Corda) Hansgirg 1888	0.01776	0
Cryptophyceae	*Cryptomonas erosa*<br>Ehrenberg 1832	0.100224	0.267264
	Cryptomonas ovata<br>Ehrenberg 1832	0.49824	0.298944
	Cryptomonas sp.<br>Ehrenberg 1831	0.3672	0
	Plagioselmis nannoplanctica<br>(Skuja) G.Novarino, I.A.N.Lucas & Morrall 1994	0.05184	0.004704

Group	Taxon<br>Authority	Summer biomass [mg L^{-1}]	Winter biomass [mg L^{-1}]
Dinophyceae	*Ceratium hirundinella*<br>(O.F.Müller) Dujardin 1841	0.608352	0
	Gymnodinium sp.<br>F.Stein 1878	0	0.09096
	Peridiniopsis cunningtonii<br>Lemmermann 1907	0	0.234816
Haptophyceae	*Chrysochromulina parva*<br>Lackey 1939	0.063048	0
